# Genus-level revision of the Alycaeidae (Gastropoda, Cyclophoroidea), with an annotated species catalogue

**DOI:** 10.3897/zookeys.981.53583

**Published:** 2020-10-29

**Authors:** Barna Páll-Gergely, Sheikh Sajan, Basudev Tripathy, Kaibaryer Meng, Takahiro Asami, Jonathan D. Ablett

**Affiliations:** 1 Plant Protection Institute, Centre for Agricultural Research, Herman Ottó út 15, Budapest, H-1022, Hungary Plant Protection Institute, Centre for Agricultural Research of the Hungarian Academy of Sciences (MTA) Budapest Hungary; 2 Zoological Survey of India, Prani Vigyan Bhawan, M Block, New Alipore, Kolkata 700053, West Bengal, India Zoological Survey of India Kolkata India; 3 Wildlife Institute of India, Chandrabani, Dehradun 248 002, Uttarakhand, India Wildlife Institute of India Dehradun India; 4 National Zoological Museum of China, Institute of Zoology, Chinese Academy of Sciences, Beijing, China Institute of Zoology, Chinese Academy of Sciences Beijing China; 5 Department of Biology, Shinshu University, Matsumoto 390-8621, Japan Shinshu University Matsumoto Japan; 6 Mollusca Section, Invertebrates Division, Department of Life Sciences, The Natural History Museums, London SW7 5BD, United Kingdom Natural History Museum London United Kingdom

**Keywords:** land snail, museum collections, systematics, taxonomy

## Abstract

412 species-group names (including 11 replacement names), and 14 genus-group names of the Alycaeidae have been introduced to date. Type materials of 85% (336) of the known species and subspecies were examined, a further 5% (19) of the taxa were studied using available non-type material, and for another 6% (22) the original descriptions were sufficiently detailed to evaluate their taxonomic status. Only 3% of the taxa (12) could not be examined. Special attention was paid to the sculpture of the embryonic whorls and the sutural tube-microtunnel system in order to provide a novel classification for this group.

In this study 363 taxa (320 species or 43 subspecies) are accepted within the family Alycaeidae. Of these, 22 have been described by the lead author and his coauthors in previous publications. In addition, there are 18 species that were formerly classified in *Cycloryx* and now belong to *Pincerna* due to its synonymy with *Cycloryx*. Among the remaining 323 species, 209 (65%) are transferred here to another genus, whilst 114 (35%) have remained in their original genus.

Seven genera are accepted. While some questions (e.g., the distinction between *Pincerna* and *Alycaeus*) remained unanswered, this revision made three main achievements: (1) The *Dicharax* species were identified based on the absence of spiral striation on the entire shell; (2) the *Metalycaeus* species were identified based on the spiral striation of the protoconch; (3) and *Stomacosmethis* was separated from *Alycaeus* based on the extremely short sutural tube.

Five nominal species are being synonymised with other species, and eight species are now treated as subspecies. The following replacement names are proposed: *Dioryx urnula niosiensis* Páll-Gergely, **nom. nov.** for Alycaeus urnula var. daflaensis Godwin-Austen, 1914; *Dioryx urnula rotundus* Páll-Gergely, **nom. nov.** for Alycaeus urnula var. globosus Godwin-Austen, 1914; *Pincerna crenilabris juttingae* Páll-Gergely, **nom. nov.** for *Alycaeus crenilabris laevis* van Benthem Jutting, 1959; *Pincerna crenilabris korintjiensis* Páll-Gergely, **nom. nov.** for *Alycaeus crenilabris latecostatus* van Benthem Jutting, 1959; *Dicharax conicus jatingaensis* Páll-Gergely, **nom. nov.** for Alycaeus conicus var. nanus Godwin-Austen, 1914; *Metalycaeus godwinausteni* Páll-Gergely, **nom. nov.** for *Alycaeus neglectus* Godwin-Austen, 1914; and finally *Metalycaeus suhajdai* Páll-Gergely, **nom. nov.** for *Alycaeus varius* Godwin-Austen, 1914.

## Introduction

The Alycaeidae are operculate land snails in the superfamily Cyclophoroidea. Approximately 360 Asian species and subspecies have been described so far, and classified into 14 genera or subgenera. Alycaeids inhabit a vast area that stretches from the Western Ghats (India) through the Himalaya to Japan in the east, the Chinese Gansu and Shaanxi provinces in the north and Indonesia to the south (Godwin-Austen 1882–1920; Gude 1921; van Benthem Jutting 1948, 1959; Minato 1988; Gittenberger et al. 2017; Aravind & Páll-Gergely 2019). The alycaeid shell is characterised by a tube, which is closed at its outer end, and opens into the inside of the shell just behind the operculum. This tube is in contact with numerous, extremely narrow tunnels, which are formed by the outermost shell layer (Páll-Gergely et al. 2016).

Some terrestrial caenogastropod genera lacking such a sutural tube have provisionally been assigned to the Alycaeidae. *Laotia* Saurin, 1953, which includes two species, has been included either in the Diplommatinidae, because of its similarity with *Helicomorpha*, or in the Alycaeidae, because of its resemblance to the alycaeid *Chamalycaeus* (Saurin 1953; Páll-Gergely 2014). The latest publication on *Laotia* placed this genus in Alycaeidae (Do et al. 2015). The Madagascan endemic *Boucardicus* Fischer-Piette & Bedoucha, 1965 (*Madecataulus* Fischer-Piette & Bedoucha, 1965 is a synonym, see Emberton and Pearce 1999) has also been placed in Alycaeidae due to a similar shell shape and radula (Emberton 2002; Egorov 2019). Following recent extensive surveys, there are now approximately 200 accepted *Boucardicus* species (Fischer-Piette et al. 1993; Emberton and Pearce 1999; Emberton 2002; Emberton et al. 2010; Balashov and Griffiths 2015).

Our study covers the systematics of the Alycaeidae sensu stricto, a group that is characterised by the possession of an external tube that runs along the suture (see above). Although anatomical and radular characters are known for some species (Godwin-Austen 1882–1920; Tielecke 1940; Venmans 1956; Emberton and Pearce 1999; Emberton 2001), those can only be used as supplementary information to hypothesise about the relatedness of the genera, and cannot be used for the appropriate generic placement of species at the current time. Thus, the classification presented here is primarily based on morphological characters of the shell.

The current generic subdivision of the Alycaeidae was established over a century ago and no genus-level revision has been proposed since the publication of Kobelt’s (1902) monograph. Arguably, most authors did not examine the type species of genera, especially those of *Alycaeus*, *Chamalycaeus* and *Dicharax*, when attributing new alycaeid species to any of these genera. Moreover, some allegedly diagnostic characters of the genera and subgenera may not reflect evolutionary relationships because these character states have probably evolved in convergence. For example, the low spire was regarded as the key trait of *Chamalycaeus* (Kobelt 1902). However, most *Chamalycaeus*, *Metalycaeus* and *Dicharax* species are low-spired, and even *Alycaeus jousseaumei* has a depressed shell, while, there are *Dicharax* and *Metalycaeus* species that are high spired (Páll-Gergely et al. 2017). Similarly, *Dicharax* was defined on the basis of a swelling behind the peristome (Kobelt 1902). This trait, however, occurs in several species of the genera *Metalycaeus* and *Dicharax*, and the strength of the swelling is very variable across *Dicharax* and *Metalycaeus* species (Páll-Gergely et al. 2017). Lastly, the outer surface traits of the operculum, which defined the genera *Pincerna*, *Stomacosmethis* and *Metalycaeus*, are also variable within species, and, on the other hand, show similar morphology between species not closely related.

The aim of this study is to provide an updated generic classification of the Alycaeidae based on the principles of our former paper (Páll-Gergely et al. 2017) focusing on two key traits largely neglected in previous publications, because these are presumed to be more useful to distinguish natural groups. Firstly, the sculpture on the outer shell surface and secondly, the length and sculpture of the area where the sutural tube is situated and the surface is differently ribbed from the other whorl range. Microtunnels functioning as complex gas exchange device are present with the sutural tube, which could provide useful traits for alycaeid systematics, at least in some of the groups (Páll-Gergely et al. 2016).

## Taxonomic history of Alycaeidae

Pfeiffer (1858) divided the fourteen species of the genus *Alycaeus* (equivalent to present-day Alycaeidae) known at the time into two informal groups, namely a species with subturbinate shells (“Subturbinati”) and those with depressed shells (“Depressi”). Benson (1859) named three sections within *Alycaeus* as follows. (1) *Alycaeus*: “the last whorl constricted somewhat remotely from the aperture, tumid on both sides of the constriction”; (2) *Charax*: “constriction broad, contiguous to the aperture, and divided more or less remotely from it, across the whorl, by a ridge which is hollow internally”; (3) *Dioryx*: “constriction narrow, and immediately behind the aperture; the sutural tube arising proportionally nearer to the peristome than in *Alycaeus* and *Charax*”. In Benson’s (1859) system, all the three groups were further sub-divided into unnamed subgroups on the basis of shell shape (*Alycaeus* and *Dioryx*) and the morphology of the swelling between the constriction and the aperture (*Charax*). Pfeiffer (1876) introduced the name *Orthalycaeus* as a subgenus of *Alycaeus* and divided it into four subgroups. The name was established without description, but contained 26 species, which made it available. Pfeiffer seemingly intended this name to be used as what we would call today a nominotypical subgenus. He did not select a type species, which was subsequently done by Kobelt (1879: 191), who selected *Alycaeus gibbus* as the type species of *Orthalycaeus*. Because this species is also the type species of *Alycaeus* (also by subsequent designation), these two genus names are objective synonyms. Kobelt and Möllendorff (1897) recognised two genera within the family Alycaeidae: *Dioryx* and *Alycaeus* (with the subgenera *Orthalycaeus*, *Chamalycaeus*, *Charax*). Kobelt’s (1902) monograph was based on the same system as the one by Kobelt and Möllendorff (1897), but he treated the Alycaeinae as a subfamily of the Cyclophoridae. Kobelt (1902) recognised *Dioryx* as a distinct genus and subdivided *Alycaeus* into four ‘sections’: *Alycaeus*, *Chamalycaeus* Kobelt & Möllendorff, 1897 (incorrect attribution of authors, see under *Chamalycaeus*), *Dicharax* Kobelt & Möllendorff, 1900 (replacement name for *Charax* Benson, 1859, non *Charax* Scopoli, 1777 [Pisces]) and *Metalycaeus* Pilsbry, 1900. *Metalycaeus* was described for two Japanese species (*A. melanopoma* and *A. hirasei*). Later, *A. tsushimanus* was also included in *Metalycaeus* (Pilsbry & Y. Hirase, 1909a). *Metalycaeus* has only been reported from Japan, and was diagnosed on the basis of a thickened ring on the outer side of the operculum. In later publications it was not accepted as a distinct taxon, but treated as a junior synonym of *Chamalycaeus* (see Minato 1988). Preston (1907) described the subgenus Pincerna for Alycaeus (Pincerna) liratula Preston, 1907. According to Preston *Pincerna* has an alycaeiform shell, which is higher than wide, and the operculum with a “circular cup” on its outer surface. The subgenera *Cycloryx* and *Raptomphalus* were described by Godwin-Austen (1914). The former is characterised by the ovate-conoid shell and an extremely short tube, which is often pear or club-shaped, whereas the latter has a conspicuous keel on the umbilical margin. *Stomacosmethis* Bollinger, 1918 was defined on the basis of a pipe, tongue or cup-shaped structure on the outer side of the operculum and included two species from southern Celebes and eastern Borneo. Three genus-group taxa (*Sigmacharax* Kuroda, 1943, *Cipangocharax* Kuroda, 1943, *Awalycaeus* Kuroda, 1951) were described from Japan. These have been used either as genera or as subgenera of *Chamalycaeus* and can be distinguished from each other as follows: *Sigmacharax* has a peculiar, sigmoid last whorl with an ovate aperture having an interior ridge, *Cipangocharax* has a thick, “shelly” operculum with closely imbedded spiral cuticular lamellae on its outer surface, and *Awalycaeus* has a very short distance between the starting point of the tube and the peristome, its operculum is situated at the aperture, not deeper as in other groups.

In our own works we have defined *Metalycaeus* by the presence of a spirally striated protoconch, and several species from China, Vietnam, Laos, and Japan have been placed in this genus (Páll-Gergely and Asami 2017; Páll-Gergely et al. 2017). We also found that *Chamalycaeus* possesses a protoconch without spiral striae, and a teleoconch with spiral striation. Consequently, most species previously classified as *Chamalycaeus* have been transferred to *Metalycaeus* or *Dicharax*. The latter is characterised by the absence of spiral striation on the entire shell (Páll-Gergely et al. 2017). We further synonymised *Cycloryx* with *Pincerna*, which is accepted as a distinct genus (Páll-Gergely 2017).

## Materials and methods

Specimens (shells and radulae) were examined using a low vacuum SEM (Miniscope TM-1000, Hitachi High-Technologies, Tokyo) directly without coating. The ethanol-preserved specimens were dissected under a Zeiss stereomicroscope, and photographs were taken using a Keyence LHX5000 digital microscope.

Photographs of shells were taken using various photographic equipment in our laboratories and in museum collections. Photographs of types deposited in the IZCAS, ZSI, SMF are published here. In cases of the other museums the photographs of types are mostly available online, or they will be published by us in separate papers.

Locality data cited as verbatim from the specimen labels, and no English translations are provided in most cases.

Differences in size are indicated in the generic diagnosis using the following terms: very small (smaller than 3 mm), small (3–4 mm), medium-sized (4–6 mm), large (6–8 mm), very large (larger than 8 mm). We distinguish three regions of the teleoconch, following Páll-Gergely et al. (2017): Region 1 (R1) ranging from the beginning of the teleoconch to the beginning of the differently ribbed region where the sutural tube lies, Region 2 (R2) extending from the end of R1 to the constriction (i.e., the length of R2 usually corresponds with the length of the sutural tube, see Páll-Gergely et al. 2016; Páll-Gergely and Asami 2017), and Region 3 (R3) ranging from the constriction up to the peristome.

In order to maintain consistency with the editorial conventions of MolluscaBase (2020), initials of first names of authors are indicated in all cases where a given author shares the same family name with another malacologist (i.e., Y. Hirase, L. Pfeiffer).

### Specimens used for anatomical study

*Alycaeus eydouxi* Venmans, 1956: Vietnam, Marble Mountain, Da Nang, coll. No. V142, NHM 2008 VN expedition, 26.05.2008, NHMUK 20160702.

*Alycaeus gibbosulus* Stoliczka, 1872: ALY03, Malaysia, Malay Peninsula, Baling, after a large bridge of the Baling River, 05°40.950'N, 100°54.883'E, 100 m, leg. Fatley, R., Juhász, A., Majoros, G., Motochin, R., Páll-Gergely, B., 22.07.2016, HNHM 104424.

*Dioryx messageri* (Bavay & Dautzenberg, 1900): Vietnam, Ninh Bình, Cuc Phuong National Park (site 3), 20°18.141'N, 105°39.240'E, NHM 2013 VN expedition, 09.09.2013, NHMUK 20140343.

*Dicharax tokunoshimanus principalis* (Pilsbry & Y. Hirase, 1909): Japan, Kagoshima Prefecture, Amamioshima, Akina, southern edge of the village, 28°26.623'N, 129°33.381'E, 15 m, leg. Hunyadi, A., Miyai, T., Nakahara, Y., Otani, J.U., Páll-Gergely, B. & Yano, Sh., 01.10.2016, 2016.10.01B, spec2, HNHM 104428.

*Metalycaeus minatoi* Páll-Gergely, 2017: 20151214A, Japan, Kagoshima Pref., Tanegashima, Kumage-gun, Minamitane-chō, Kukinaga hōmanjinja, 30°23.051'N, 130°56.108'E, leg. Nakahara, Y., Otani, J.U. & Páll-Gergely, B., 14.12.2015, HNHM 104427.

*Stomacosmethis dohrni* (O. Boettger, 1893): Indonesia, Kalimantan Selatan, Beramban, leg. Yansen Chen, Apr 2012, HNHM 104426.

*Stomacosmethis balingensis* (Tomlin, 1948): Malaysia, Malay Peninsula, Baling, after a large bridge of the Baling River, 05°40.950'N, 100°54.883'E, 100 m, leg. Fatley R., Juhász A., Majoros G., Motochin R., Páll-Gergely B., 22.07.2016., HNHM 104425.

### Specimens used for examining the radula

*Alycaeus eydouxi*: Vietnam, Marble Mountain, Da Nang, coll. No. V142, NHM 2008 VN expedition, 26.05.2008, NHMUK 20160702.

*Alycaeus gibbosulus* Stoliczka, 1872: Malaysia, Penang, Penang National Park, Around Monkey beach, 5°28.457'N, 100°11.165'E, 81 m a.s.l. (ALY30 in molecular study), leg. Hirano, T., 21.07.2016, HNHM 104857.

*Dioryx messageri* (Bavay & Dautzenberg, 1900): Vietnam, Hòa Bình Province, 20°21.329'N, 105°38.005'E, NHM 2013 VN expedition, NHMUK 20140281.

*Chamalycaeus* sp.: Indonesia, Tukik River, Central Aceh, GPS n.a., leg. Yansen Chen, (specimen1, ALY17 in molecular study), HNHM 104858.

*Chamalycaeus everetti* (Godwin-Austen, 1889): Indonesia, Kalimantan Selatan, Beramban, leg. Yansen Chen, Apr 2012, HNHM 104859.

*Dicharax itonis* (Kuroda, 1943): Japan, Hiroshima, Mihai city, Kui-cho, ex coll. K. Ohara, 24.10.2015, HNHM 104860.

Dicharax (?) okinawaensis (Uozumi, Yamamoto & Habe, 1979): Japan, Okinawa, Ogimi, Mt. Nekumachiji, 26°40.977'N, 128°8.332'E, 304 m, leg. Hirano, T. 09.09.2015, HNHM 104431.

*Stomacosmethis balingensis* (Tomlin, 1948): Malaysia, Malay Peninsula, Baling, after a large bridge of the Baling River 05°40.950'N, 100°54.883'E, leg. Fatley, R., Harl, J., Juhász, A., Majoros, G., Motochin, R., Páll-Gergely, B., 22.07.2016. (2016.07.22A, specimen1), HNHM 104861.

*Stomacosmethis perakensis* (Crosse, 1879): 2016.07.22A, Malaysia, Malay Peninsula, Baling, after a large bridge of the Baling River, 05°40.950'N, 100°54.883'E, leg. Fatley, R., Harl, J., Juhász, A., Majoros, G., Motochin, R., Páll-Gergely, B., 22.07.2016, HNHM 104430.

### Abbreviations

**AMNH**American Museum of Natural History (New York, USA);

**ANSP**Academy of Natural Sciences (Philadelphia, USA);

**D** shell diameter;

**HA** Collection András Hunyadi (Budapest, Hungary);

**HBUMM** Mollusc collection of the Museum of Hebei University (Baoding, China);

**HNHM**Hungarian Natural History Musem (Budapest, Hungary);

**IZCAS**National Zoological Museum of China, Institute of Zoology, Chinese Academy of Sciences (Beijing, China);

**MCZ**Museum of Comparative Zoology (Massachusetts, USA);

**MNHN**Muséum National d’Histoire Naturelle (Paris, France);

**MZB**Museum Zoologicum Bogoriense (Bogor, Indonesia);

**NC** Nishinomiya Shell Museum (Hyogo, Japan);

**NHMB**Naturhistorisches Museum, Basel (Basel, Switzerland);

**NHM** The Natural History Museum (London, UK);

**NHMUK** When citing lots deposited in the NHM;

**NHMW**Museum of Natural History of Vienna (Vienna, Austria);

**NZSI** National Zoological Collection of the Zoological Survey of India (when cited specimens deposited in the ZSI);

**NSMT**National Museum of Nature and Science, Tsukuba, Japan;

**PGB** Collection Barna Páll-Gergely (Mosonmagyaróvár, Hungary);

**RBINS**Royal Belgian Institute of Natural Sciences (Brussels, Belgium);

**RMNH**National Museum of Natural History Naturalis (Leiden, The Netherlands);

**SMF**Senckenberg Forschungsinstitut und Naturmuseum (Frankfurt am Main, Germany);

**UF**Florida Museum of Natural History, University of Florida (USA);

**UMMZ**University of Michigan, Museum of Zoology (Ann Arbor, USA);

**UMZC** University Museum of Zoology (Cambridge, United Kingdom);

**USNM**Smithsonian National Museum of Natural History (Washington, USA);

**ZMB**Museum für Naturkunde (Berlin, Germany);

**ZSI**Zoological Survey of India (Kolkata, India).

## Description and assessment of morphological characters

### Shell morphology

#### Protoconch sculpture (Fig. 1)

**Figure 1.**
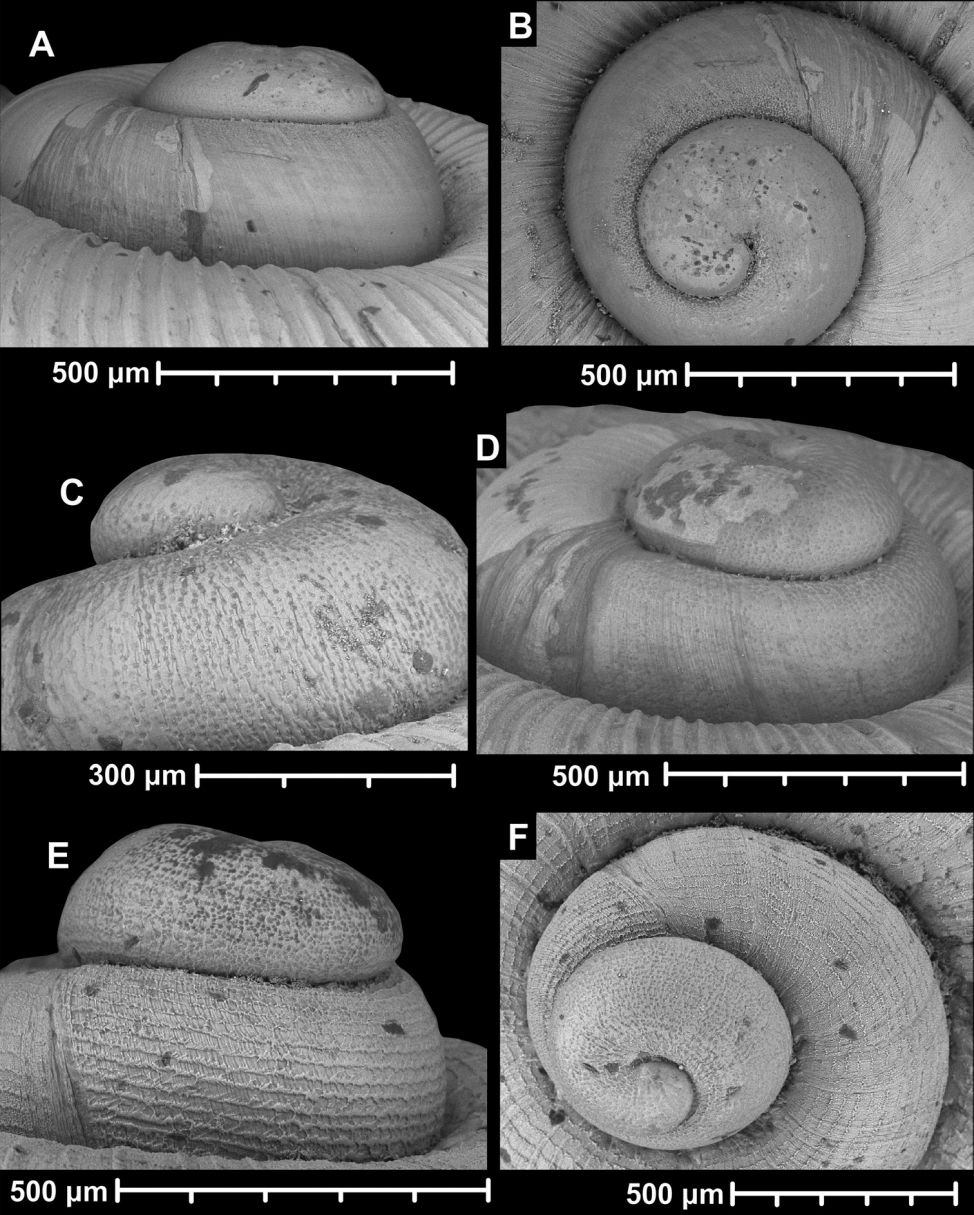
Traits of alycaeid protoconch **A, B** smooth: *Dicharax cristatus* (Möllendorff, 1886) (2010.05.08A, coll. PGB) **C, D** pitted. C: *Chamalycaeus sculptilis* (Benson, 1856) (NHMW 71770/R/17); D: *Dicharax itonis* (Kuroda, 1943) (NSMT 78866) **E, F** spirally striated: *Metalycaeus muciferus* (Heude, 1885) (2013/7, coll. PGB). All images: Barna Páll-Gergely.

**Figure 2.**
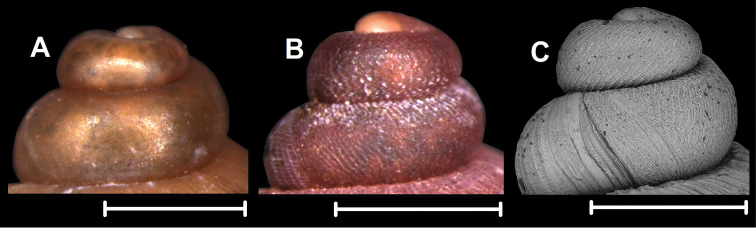
Protoconch sculpture of *Alycaeus* Gray, 1850 species **A***Alycaeus eydouxi* Venmans, 1956 (Cochinchina, coll. V.W. MacAndrew, NHMUK) **B***Alycaeus rolfbrandti* Maassen, 2006 (MNHN-IM-2012-27321) **C***Alycaeus conformis* Fulton, 1902 (HNHM 99714, spec3). Scale bars: 1 mm. All images: Barna Páll-Gergely.

The protoconch is spirally striated in *Metalycaeus* (Fig. 1E, F) (rarely unstriated, see *M. laevis*), and “smooth” (glossy, finely granular or very finely pitted) in all the other genera (Fig. 1A–D). Two species (*Alycaeus conformis* and *A. gibbosulus*) exhibit oblique striae on the protoconch (Fig. 2C; Foon & Liew, 2017). They are placed in *Alycaeus* because the protoconch of *A. rolfbrandti* (a species otherwise similar to the type species, *A. eydouxi*) is finely scaly/tuberculated in oblique lines at the end of the protoconch (Fig. 2B). This sculpture is seemingly an intermediate character state between the smooth (Fig. 2A) and obliquely striated types (Fig. 2C).

#### Protoconch shape

Among the genera with usually depressed shell shape (*Dicharax*, *Chamalycaeus*, *Metalycaeus*), the protoconch is also low (depressed) in the majority of *Dicharax* species. It is, however, often more elevated in the other two genera than what we would expect from the overall low spire. This was not the case for every single species, but generally *Chamalycaeus* and *Metalycaeus* possess more elevated protoconch than *Dicharax*. When the protoconch sculpture is not clearly visible due to corrosion, general differences in the protoconch shape between *Dicharax* and *Chamalycaeus*/*Metalycaeus* may help in generic classification. In the genera with generally higher spire (*Alycaeus*, *Dioryx*, *Pincerna*, *Stomacosmethis*) the protoconch is as elevated as what we would expect from the high spire.

#### R1 sculpture

Spiral striation is absent only in *Dicharax*. All the other genera possess some spiral striae of varying development. These spiral striae consist of microscopic elevated ridges arranged in clearly visible spiral lines. Some spiral striae visible in a few *Dicharax* species (e.g., *D. candrakirana*, *D. depressus*), however, is appear to be a part of the inner shell layers and may not be homologous with those of *Chamalycaeus* and *Metalycaeus*. The strength of radial ribbing is also informative (usually strong in *Chamalycaeus* and *Pincerna*, weak in *Alycaeus* and *Stomacosmethis*). *Dioryx* has overall weak sculpture, whereas it is highly variable in *Metalycaeus*.

#### Length of R2

*Stomacosmethis* is characterised by very short R1 (with a short, tumid, sometimes pear-shaped tube), whereas *Alycaeus* possesses very long R1 (ca. 0.5 whorl). Most *Pincerna* species, especially the ones classified in *Cycloryx* previously, possess a short tube, but some species have a longer tube than usual for that genus. Distinction between longer-tubed *Pincerna* and *Alycaeus* is the most problematic part of the current classification. The remaining genera (*Chamalycaeus*, *Dicharax*, *Dioryx*, *Metalycaeus*) exhibit high variability in terms of the tube length.

#### Sculpture of R2

Highly variable within each genus with the exception of *Dioryx*, which has no elevated R2 ribs. Typically, *Chamalycaeus* and *Metalycaeus* species possess widely spaced, sharp ribs. However, *Metalycaeus vinctus* has widely spaced, sharp ribs, but its putative sister species *M. minatoi* has a smooth outer surface in R2 without any elevated ribs. This seems to indicate that this trait may differ substantially even between closely related species. Similar examples are found among some Himalayan *Metalycaeus* species, and also among the Chinese *Dicharax moellendorffi* vs. other *Dicharax* species. Typical *Dicharax* species possess R2 ribs that curve towards the aperture.

#### Development of R3

In most alycaeid genera, except for *Dioryx*, R3 is strongly developed. The commonly weak shell sculpture may suggest the monophyly of *Dioryx*. The R3 area is occasionally reduced in other genera also, such as in *Chamalycaeus microconus*, *C. mixtus*, *Dicharax akioi*, and *Alycaeus conformis*, which are classified in their respective genera based on other characters.

#### Operculum

The inner side is with or without central nipple. When present, its extent and height may vary between or within species (Páll-Gergely et al. 2017). The outer surface is usually smooth, but can have a closely coiled spiral lamella (in *Dicharax* and *Metalycaeus*, see Páll-Gergely et al. 2017), which may result in a circular ring (*Dicharax bison*, *Metalycaeus nipponensis*). It is unknown whether the pipe, tongue or cup-shaped structure in some *Pincerna* and *Stomacosmethis* species is homologous with the similarly circular structure of *Dicharax* and *Metalycaeus*. *Metalycaeus*, *Pincerna* and *Stomacosmethis* were originally defined on the basis of opercular characters. The outer surface of the operculum can also be finely granulated and flaky with short calcareous spikes or scaffold-like calcareous deposits. These traits are generally (but not always) consistent within each species, making them useful for species recognition (Foon and Liew 2017; Páll-Gergely et al. 2017). Although in some cases opercular characters may suggest relatedness, it does not appear to be useful for subdividing the Alycaeidae into genera. Thus, we do not use opercular traits in our system.

### Anatomy

Females of seven species belonging to seven genera were examined. See corresponding locality data under Materials and methods.

**Figure 3.**
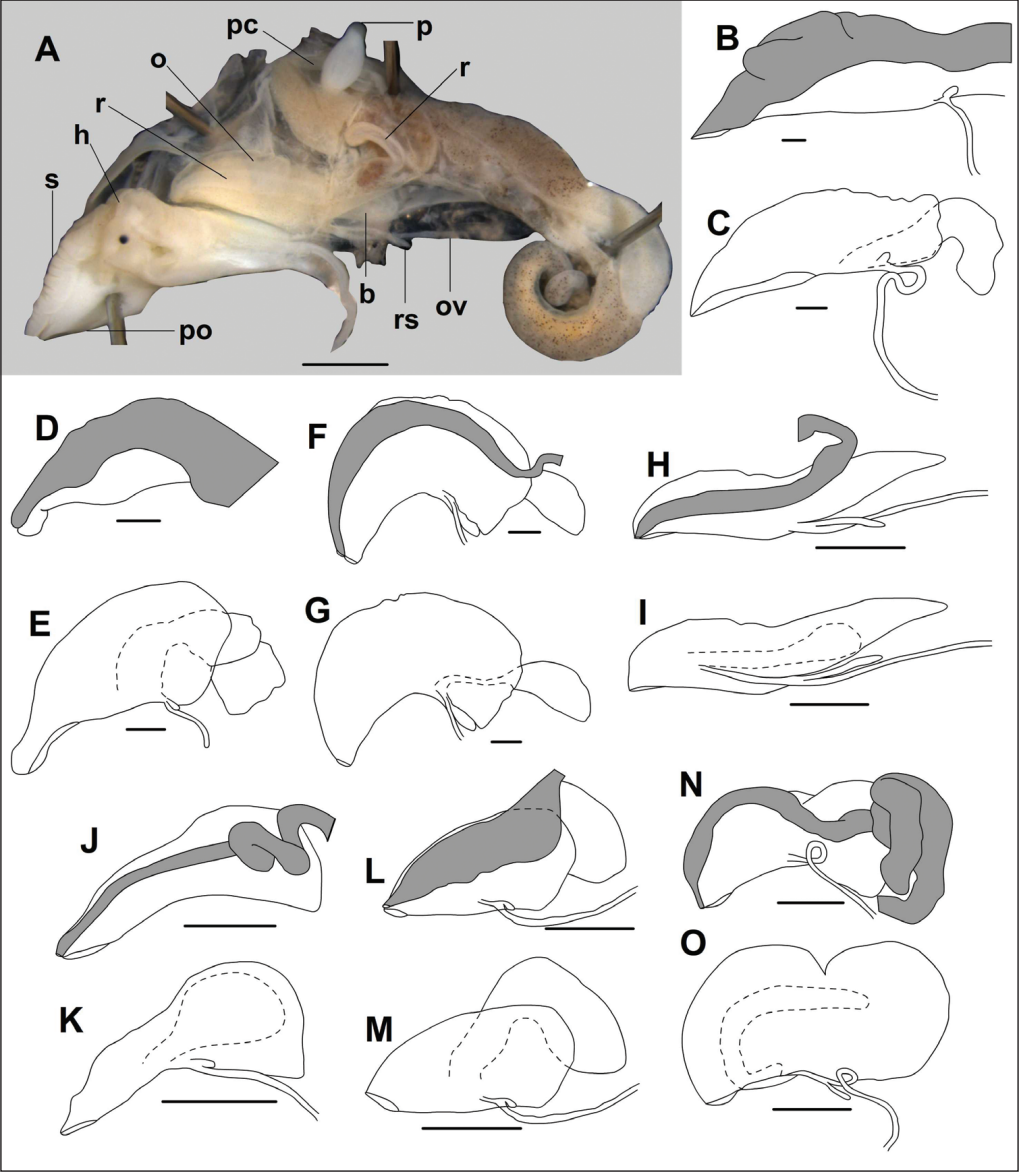
Female genital anatomy of Alycaeidae**A** positioning of females during anatomical examination **B, C***Alycaeus eydouxi* Venmans, 1956 (NHMUK 20160702, V142, specimen5) **D, E***Alycaeus gibbosulus* Stoliczka, 1872 (ALY03) **F, G***Dioryx messageri* (Bavay & Dautzenberg, 1900), NHMUK 20140343 **H, I***Dicharax tokunoshimanus principalis* (Pilsbry & Y. Hirase, 1909) HNHM 104428 (2016.10.01B, spec2) **J, K***Metalycaeus minatoi* Páll-Gergely, 2017 HNHM 104427 (2015.12.14A, female28) **L, M***Stomacosmethis dohrni* (O. Boettger, 1893) HNHM 104426 **N, O***Stomacosmethis balingensis* (Tomlin, 1948), HNHM 104425 (2016.07.22A, sp2). Abbreviations: B: bursa copulatrix; H: head; O: ovarium; OV: oviduct; P: trematode parasite (found in the pericardium); PC: pericardium; PO: position of the operculum (operculum removed); R: rectum; RS: receptaculum seminis; S: sole. Upper images of each pair (**B, D, F, H, J, L, N**) shows the genitalia before removing the rectum (with grey shading). Scale bars: 1 mm. Photograph and drawings: Barna Páll-Gergely.

*Alycaeus eydouxi* Venmans, 1956: ovarium elongated, spindle-shaped, bursa copulatrix curved, relatively slender, opens near centre of ovarium, strongly extends beyond ovarium posteriorly, receptaculum seminis small, rounded (Fig. 3B, C).

*Alycaeus gibbosulus* Stoliczka, 1872: ovarium wide with pointed anterior and blunt posterior end, bursa copulatrix large, extends beyond ovarium posteriorly, opens at middle part of ovarium near its base, bursa has a thickened posterior portion; receptaculum seminis small, oval (Fig. 3D, E).

*Dioryx messageri* (Bavay & Dautzenberg, 1900): ovarium oval, anterior end pointed, posterior end blunt, bursa copulatrix relatively small, oval, strongly extends beyond ovarium posteriorly, its stalk slender, opens posterior to centre of ovarium, receptaculum seminis elongate (Fig. 3F, G).

*Dicharax tokunoshimanus principalis* (Pilsbry & Y. Hirase, 1909): Ovarium elongated, pointed posteriorly, bursa copulatrix relatively slender with blunt bursa, does not extend beyond ovarium, receptaculum seminis strongly elongated (Fig. 3H, I).

*Metalycaeus minatoi* Páll-Gergely, 2017: ovarium slender, pointed anteriorly and rounded posteriorly, bursa copulatrix, rounded, does not extend beyond ovarium, opens near opening of ovarium, receptaculum seminis small, rounded (Fig. 3J, K).

*Stomacosmethis dohrni* (O. Boettger, 1893): shape of ovarium could not be examined due to its decayed condition, but it is probably oval, bursa copulatrix large, elongate, strongly extends beyond ovarium posteriorly, opens at centre of ovarium, receptaculum seminis small, oval (Fig. 3L, M).

*Stomacosmethis balingensis* (Tomlin, 1948): ovarium peanut-shaped, bursa copulatrix was damaged, its posterior part could not be dissected out, opens at anterior part of ovarium, near ovarium opening, receptaculum seminis elongated, a complicated spermoviduct was found in bursa copulatrix: its head is pointed drop-shaped, both ends of the head connected to a slender stalk that forms a flattened loop, the entire length of the stalk is continuous, forming a ring (Fig. 3N, O).

Our knowledge of genital anatomy of terrestrial operculate snails is far more limited than that of pulmonates, probably because dissection of the soft body is more difficult. Firstly, the reproductive organs are not so clearly separated as in pulmonates, but are attached to neighbouring tissues and organs. Secondly, tissues of ethanol-preserved animals are far more fragile. Therefore, it is much more difficult to see the boundaries and junctions of certain organs. For the current study much of the ethanol-preserved material was not in a suitable condition for reproductive anatomy. More than half of the available material was not used for this reason.

So far, the reproductive anatomy of the Alycaeidae is little known. Tielecke (1940) published a few notes without figures on two alycaeid taxa. Although we have dissected a few female specimens, our observations of reproductive anatomy are insufficient to feed into our classification between genera. Considerable differences could be observed in the relative size of the bursa copulatrix, thickness and origin of the bursa’s stalk, shape of the bursa and receptaculum seminis. The taxonomic value of these traits must be clarified by further observations. Nevertheless, the bursa copulatrix originates from the lateral side of the ovarium, which may probably be a synapomorphic character of the Alycaeidae. In contrast, the bursa starts from the terminal (distal) end of the ovarium in all the anatomically examined specimens of Cyclophoridae (Tielecke 1940).

### Radula

**Figure 4.**
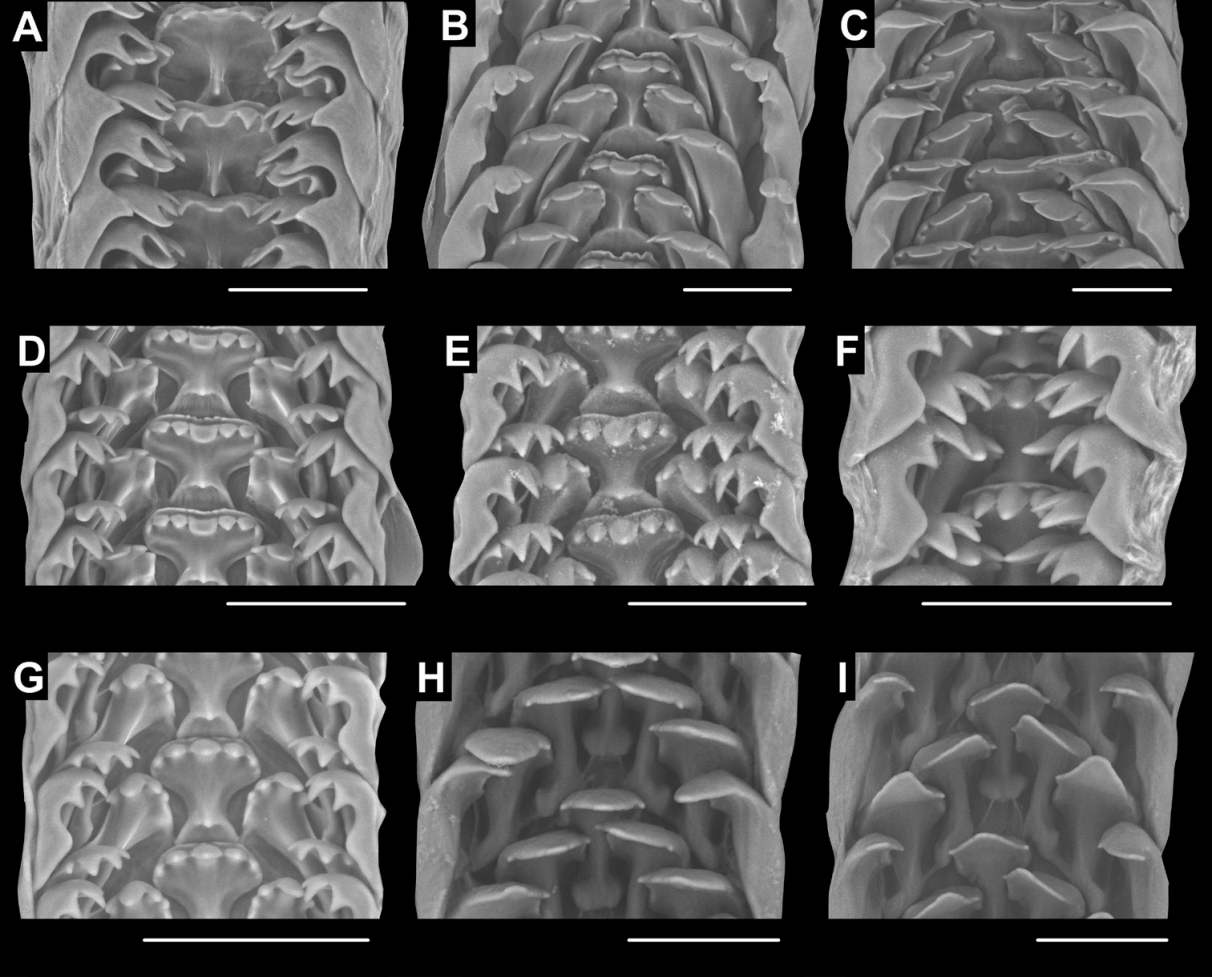
Radulae of Alycaeidae**A***Alycaeus eydouxi* Venmans, 1956 NHMUK 20160702 **B***Alycaeus gibbosulus* Stoliczka, 1872, HNHM 104857 **C***Dioryx messageri* (Bavay & Dautzenberg, 1900) NHMUK 2014.02.81 **D***Chamalycaeus* sp., HNHM 104858 **E***Chamalycaeus everetti* (Godwin-Austen, 1889), HNHM 104859 **F***Dicharax itonis* (Kuroda, 1943) HNHM 104860 **G***Dicharax okinawaensis* (Uozumi, Yamamoto & Habe, 1979), HNHM 104431 **H***Stomacosmethis balingensis* (Tomlin, 1948) HNHM 104861 **I***Stomacosmethis perakensis* (Crosse, 1879) HNHM 104430. Scale bars: 50 μm. All images: Barna Páll-Gergely.

Radulae of nine species belonging to five genera were examined: *Alycaeus eydouxi* Venmans, 1956 (Fig. 4A), *Alycaeus gibbosulus* Stoliczka, 1872 (Fig. 4B), *Dioryx messageri* (Bavay & Dautzenberg, 1900) (Fig. 4C), *Chamalycaeus* sp. (Fig. 4D, Suppl. material 1: Fig. S1), *Chamalycaeus everetti* (Godwin-Austen, 1889) (Fig. 4E), *Dicharax itonis* (Kuroda, 1943) (Fig. 4F), Dicharax (?) okinawaensis (Uozumi, Yamamoto & Habe, 1979) (Fig. 4G), *Stomacosmethis balingensis* (Tomlin, 1948) (Fig. 4H), *Stomacosmethis perakensis* (Crosse, 1879) (Fig. 4I). See corresponding locality data under Materials and methods. For descriptive note on the radular traits, see Table 1 and Fig. 4.

**Table 1.** Radula traits of Alycaeidae. For the species examined by Venmans (1956) see Radula under “Description and assessment of morphological characters” (page 14).

Taxon	Morphology of central tooth	Reference
*Alycaeus conformis*	see remarks	Venmans 1956
*Alycaeus eydouxi*	see remarks	Venmans 1956
*Alycaeus eydouxi*	5 cusps, broad, central cusp blunt	this study
*Alycaeus gibbosulus*	5 cusps, broad, central cusp blunt	this study
*Chamalycaeus everetti*	5 cusps, broad, central cusp pointed	this study
*Dicharax alticola*	5 cusps, broad, central cusp pointed	Páll-Gergely et al. 2017
*Dicharax ananensis*	5 cusps, broad, central cusp pointed	Yano et al. 2013
*Dicharax bicrenatus*	5 cusps, broad, central cusp pointed	Godwin-Austen 1884 (1882–1920)
*Dicharax cristatus*	5 cusps, broad, central cusp pointed	Páll-Gergely et al. 2017
*Dicharax depressus*	5 cusps, broad, central cusp pointed	Páll-Gergely et al. 2017
*Dicharax fimbriatus*	5 cusps, broad, central cusp pointed	Páll-Gergely et al. 2017
*Dicharax immaculatus*	5 cusps, broad, central cusp pointed	Páll-Gergely et al. 2017
*Dicharax itonis*	5 cusps, broad, central cusp pointed	this study
*Dicharax longituba*	5 cusps, broad, central cusp pointed	Benthem-Jutting 1948
*Dicharax okinawaensis*	5 cusps, broad, central cusp pointed	this study
*Dicharax planorbulus*	5 cusps, broad, central cusp pointed	Heude 1882–1890
*Pincerna maolanensis*	7 cusps, broad, central cusp pointed	Luo et al. 2009
*Dioryx messageri*	7 cusps, broad, central cusp blunt	this study
*Dioryx setchuanensis*	5 cusps, broad, central cusp blunt	Heude 1882–1890
*Metalycaeus minatoi*	5 cusps, broad, central cusp pointed	Páll-Gergely & Asami, 2017
*Metalycaeus vinctus*	5 cusps, broad, central cusp pointed	Páll-Gergely & Asami, 2017
*Metalycaeus zayuensis*	5 cusps, broad, central cusp pointed	Zhang et al. 2008
*Pincerna thieroti*	see remarks	Venmans 1956
*Pincerna yanseni*	5 cusps, broad, central cusp pointed	Páll-Gergely 2017
*Stomacosmethis balingensis*	3 cusps, elongated, central cusp pointed	this study
*Stomacosmethis hochstetteri* (synonym of *jagori*)	3 cusps, elongated, central cusp pointed	Bollinger 1918
*Stomacosmethis jagori*	3 cusps, elongated, central cusp pointed	Thiele 1929
*Stomacosmethis jagori*	1 cusp, elongated, central cusp pointed	Sarasin & Sarasin, 1899
*Stomacosmethis kapayanensis selangoriensis*	see remarks	Venmans 1956
*Stomacosmethis kuekenthali*	1 cusp, elongated, central cusp pointed	Sarasin & Sarasin, 1899
*Stomacosmethis perakensis*	3 cusps elongated, central cusp pointed	this study
*Stomacosmethis porcilliferus*	3 cusps elongated, central cusp pointed	Bollinger 1918
*Stomacosmethis sarasinorum*	5 cusps, elongated, central cusp pointed	Bollinger 1918
Dicharax (?) panshiensis	6 cusps ?, broad, central cusp pointed	Chen 1989

Venmans (1956) described the radular morphology of four alycaeid species. The structure of the radula in *Alycaeus eydouxi* and *S. kapayanensis* (specimens were collected as Batu Caves, which is the type locality of *S. kapayanensis selangoriensis*, see Foon and Liew 2017) are especially interesting. Venmans (1956) published drawings of the radula of a specimen of *Alycaeus eydouxi* which had an elongated, spatula-like central tooth without any side cusps. Our observations of the same species from the same locality were, however, strikingly different. The specimens we examined had a blunt central tooth with a blunt, central cusp, and four pointed side-cusps. In order to confirm the identification of Venmans, we examined his specimens. We confirmed that they were indeed *A. eydouxi* shells. Furthermore, so far, the only *Stomacosmethis* radula with a blunt central tooth with cusps has been that of *S. kapayanensis* figured by Venmans (1956). It is highly likely that Venmans mixed the radular drawings or radulae of those two species. Therefore, in our analysis of alycaeid radulae we ignore the data of Venmans (1956).

The radula morphology of 28 species are known from the available literature and this study (excluding the results of Venmans 1956). In every species the radular teeth are arranged in v-shaped rows, each transverse row with seven teeth (2-1-1-1-2). The central tooth is strongly constricted in its middle part. The lateral and two marginal teeth have slighter median constriction of the plates and are seemingly longer and slenderer than the central tooth, except for *Stomacosmethis*, where they are of comparable width to the central teeth. The lateral and marginal teeth are similar in terms of shape of the cusps to the central teeth in all examined specimens.

There are generally two types of central teeth. One type has one round tooth with 5–7 cusps, and the other is elongated with 1–5 (usually 1–3) cusps. The former type is common among all the genera (*Alycaeus*, *Chamalycaeus*, *Dicharax*, *Dioryx*, *Metalycaeus*, *Pincerna*), whereas the latter, elongated type has only been observed in *Stomacosmethis* species. The placement of *S. balingensis* in *Stomacosmethis* is mainly based on its radular morphology, which is similar to that of sympatric *S. perakensis*. The first type of central teeth can be further subdivided into groups with a blunt (*Alycaeus*, *Dioryx*) or pointed (*Chamalycaeus*, *Dicharax*, *Metalycaeus*, *Pincerna*) central cusp. However, a *Chamalycaeus* species we examined had a blunt central cusp. The type with a pointed central cusp is a probably plesiomorphic character, which is visible in many terrestrial caenogastropods (e.g., *Cyclophorus*, *Cyclotus*, *Japonia*, see Egorov 2009). Together with conchological, anatomical and molecular phylogenetic information the radular traits may provide insights about relationships of alycaeid genera (see Concluding remarks).

## Genus-level diversity

Of the 14 nominal genus-group taxa that have been described (Table 2), we accept seven. The classification proposed in this study is based on unique character states (*Dicharax*, *Dioryx*, *Metalycaeus*) and on unique combinations of character states (other genera) (Table 3). The number of accepted species-level taxa is: *Alycaeus*: 7, *Chamalycaeus*: 26, *Dicharax*: 164, *Dioryx*: 30, *Metalycaeus*: 61, *Pincerna*: 37, *Stomacosmethis*: 37.

**Table 2.** Genus-group taxa of the Alycaeidae. Abbreviations: M: monotypy, OD: original designation, SD: subsequent designation. Valid genera are marked with an asterisk.

Genus	Type species	Mode of designation	Remarks
*Alycaeus Gray, 1850	*Cyclostoma gibbum* Eydoux, 1838 (= *Alycaeus eydouxi* Venmans, 1956)	SD	accepted
*Awalycaeus* Kuroda, 1951	*Awalycaeus abei* Kuroda, 1951	M	synonym of *Dicharax*
*Chamalycaeus Möllendorff 1897	Alycaeus (Chamalycaeus) fruhstorferi Möllendorff, 1897	M	accepted
*Charax* Benson, 1859	*Alycaeus hebes* Benson, 1857	SD	accepted name is *Dicharax*
*Cipangocharax* Kuroda, 1943	*Alycaeus biexcisus* Pilsbry, 1902	M	synonym of *Dicharax*
*Cycloryx* Godwin-Austen, 1914	*Cyclostoma constrictum* Benson, 1851	OD	synonym of *Pincerna*
*Dicharax Kobelt & Möllendorff, 1900	*Alycaeus hebes* Benson, 1857	SD	accepted
*Dioryx Benson, 1859	*Alycaeus amphora* Benson, 1856	SD	accepted
*Metalycaeus Pilsbry, 1900	Alycaeus (Metalycaeus) melanopoma Pilsbry, 1900	SD	accepted
*Orthalycaeus* L. Pfeiffer, 1876	*Cyclostoma gibbum* Eydoux, 1838 (= *Alycaeus eydouxi* Venmans, 1956)	SD	synonym of *Alycaeus*
*Pincerna Preston, 1907	Alycaeus (Pincerna) liratula Preston, 1907	M	accepted
*Raptomphalus* Godwin-Austen, 1914	Alycaeus (Raptomphalus) magnificus Godwin-Austen, 1914	M	synonym of *Metalycaeus*
*Sigmacharax* Kuroda, 1943	Chamalycaeus (Sigmacharax) itonis Kuroda, 1943	M	synonym of *Dicharax*
*Stomacosmethis Bollinger, 1918	Alycaeus (Stomacosmethis) sarasinorum Bollinger, 1918	SD	accepted

**Table 3.** Important traits of Alycaeid genera.

**Genus**	**Protoconch sculpture**	**Tube (R2) length**	**Shell diameter (mm)**	**Central tooth**	**Key trait**	**Unclear relationship with**
* Alycaeus *	smooth to obliquely striated	very long (ca. 1/2 whorl)	8–15	5 cusps, broad, central cusp blunt	shell very large, R2 ca. half whorl long	* Pincerna *
* Chamalycaeus *	smooth, usually elevated	variable	2–5	5 cusps, broad, central cusp pointed	shell very small to medium sized, usually depressed, R2 of variable length	* Pincerna *
* Dicharax *	smooth, usually low	variable	1–11	5–7 cusps, broad, central cusp pointed	protoconch + teleoconch without spiral striae	
* Dioryx *	smooth	variable	3.5–9	5–7 cusps, broad, central cusp blunt	shell globular or high-spired, sculpture reduced, R3 absent	
* Metalycaeus *	spirally striated, elevated	variable	3–10	5 cusps, broad, central cusp pointed	protoconch spirally striated	
* Pincerna *	smooth	very short to short	2.5–6	5 cusps, broad, central cusp pointed	shell very small to medium sized, high spired, R2 short	*Alycaeus*, *Chamalycaeus*, *Stomacosmethis*
* Stomacosmethis *	smooth	very short	3–13	elongated, usually with 1 central cusp only, or central cusps with 1–2 small cusp at each side	tiangular, colourful shell, R2 very short	* Pincerna *

**Figure 5.**
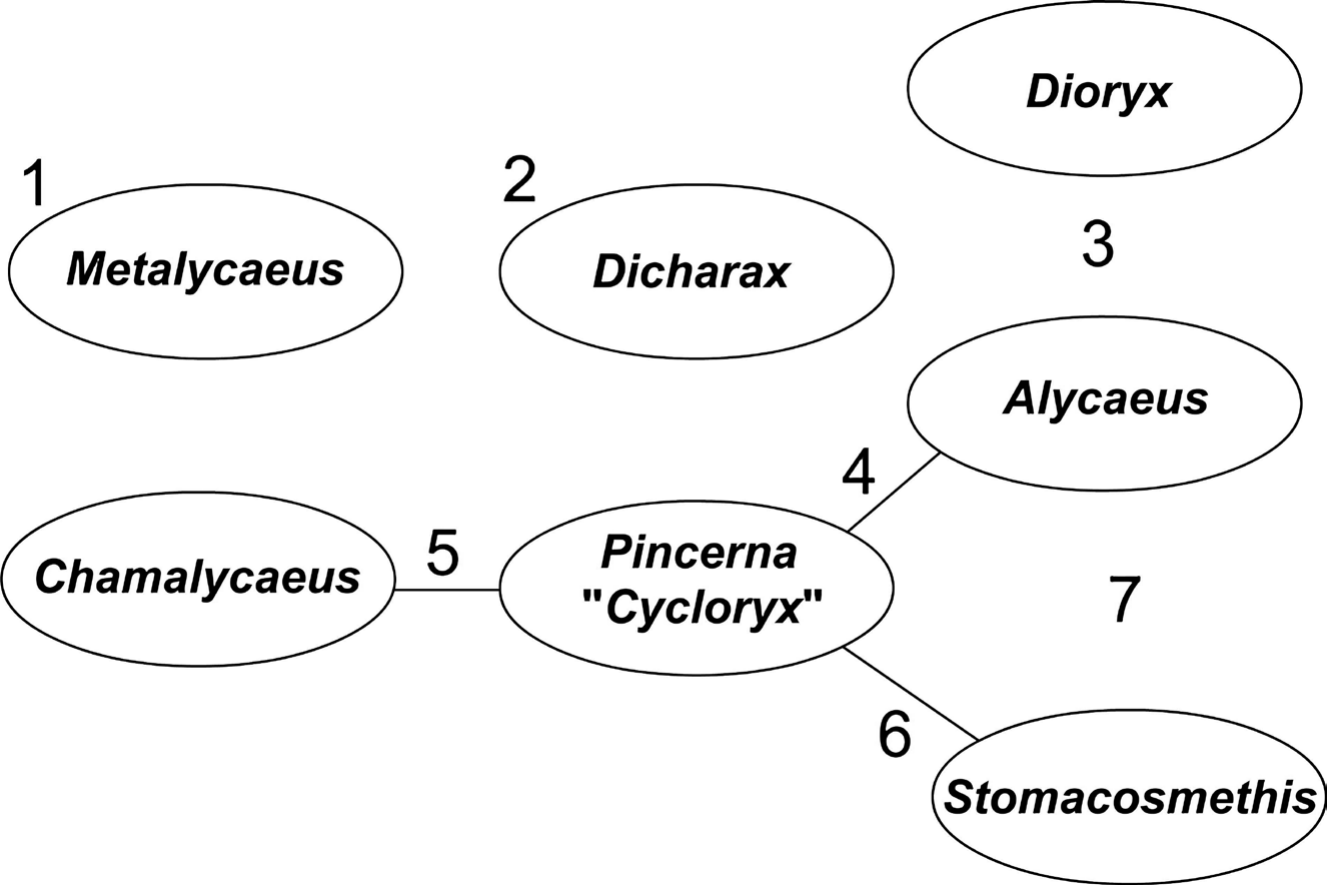
Relationships between alycaeid genera. Solid lines indicate partly unclear generic borders. See explanation in the text.

Besides taxonomic problems at the species level (see under Annotated list of species group taxa), some aspects of grouping species into genera turned out to be especially challenging. As a result, the generic boundaries are not completely clear. This may be due to repeated evolution of morphological traits and the presence of the large numbers of species in the genera. In the absence of phylogenetic analyses, the current classification is tentative. We anticipate that some adjustments are inevitable as our understanding of the evolutionary history of this family improves. Some remarks about the species boundaries (numbers correspond with Fig. 5):

(1) *Metalycaeus* is characterised by the presence of spirally striated protoconch. However, in rare cases the striation is strongly reduced (see *M. laevis*).

(2) *Dicharax* is characterised by the absence of spiral striation on both the protoconch and teleoconch. However, striation is present in a few *Dicharax* species (see remarks under *D. candrakirana*, and *D. depressus*). Striae are not elevated threads but are probably a part of the inner shell layers; this structure would not be homologous with the striae present in the other genera. *Dicharax* is a diverse genus containing 166 species and subspecies. However, meaningful subdivision is not possible at present.

(3) *Dioryx* and *Alycaeus* are clearly separated based on the presence of R3, globular shape and weak sculpture of *Dioryx*. Although the R3 in *Alycaeus conformis* renders its shell somewhat similar to the shell of *Dioryx* species, we consider *Dioryx* to be a recognisable group, which would be monophyletic.

(4) Distinction between *Alycaeus* and *Pincerna* is probably the most problematic issue in the system presented here. The genus *Cycloryx* (treated as a synonym of *Pincerna*) originally contained species with a very small shell, which has elevated spire usually with strong ribs and very short tube, and taxa from the Himalaya region and northern Myanmar. However, similar species are disjunctly found from Sumatra (*Pincerna yanseni*), northern Vietnam (*A. costulosus*) and Borneo (*A. globosus*). For example, the shell of *P. yanseni* is hardly distinguishable from that of Hymalayan taxa. The shells of *Pincerna liratula* and *P. thieroti* in the Malay Peninsula may be larger than typical *Cycloryx*, and their sutural tubes may be longer than the extremely short tube of *Cycloryx*. These two species, however, seem to belong to the same group as *Cycloryx* according to their strong ribbing, generally short tube and high spire. Furthermore, *A. vanbuensis* and *A. costulosus* both of which occur in northern Vietnam, are only distinguishable in tube length. The former is similar to the type species of *Pincerna*, and the latter to Himalayan species of *Cycloryx*. Recognition of *Pincerna* and *Cycloryx* as different genera requires to classify *A. vanbuensis* and *A. costulosus* into those genera on the basis of the tube length, which would not be acceptable. For these reasons, *Cycloryx* has been treated as a synonym of *Pincerna* (see Páll-Gergely 2017). *Alycaeus mouhoti* has a smoother and larger shell with the simple (not double) peristome than otherwise very similar *A. vanbuensis* and is also similar to *Alycaeus eydouxi* (type species of *Alycaeus*) possessing commonly large and similarly shaped shell with a rather long tube. *Alycaeus mouhoti* has shell characteristics that connect *Alycaeus* and *Pincerna*. Thus, a morphological continuum is present from *A. eydouxi*, *A. mouhoti* and *A. vanbuensis* to *A. costulosus*, which looks like a typical *Cycloryx* species. Generic subdivision on this basis would inflate the genus *Alycaeus* enormously, and *A. eydouxi* was separated from *Pincerna* and *Stomacosmethis* in our molecular phylogeny. Therefore, in this revision by a conservative approach, we included only those species in *Alycaeus* that are similar to the type species in terms of the very large shell, extremely long sutural tube (and R2) and strongly inflated body whorl.

(5) *Chamalycaeus* species have a depressed shell with reticulated sculpture and a sutural tube and R2 which vary in length. *Pincerna* species have higher spire, sculpture dominated by radial ribs and sometimes a short tutural tube. *Pincerna crenilabris* seemingly connect the two genera by its rather globular shell and medium-sized tube.

(6) The genus *Stomacosmethis* is characterised by colorful triangular shell mostly with flat whorls and a very short tube. *Pincerna* species from the distributional range of *Stomacosmethis* differ by having round whorls and a short tube. All *Stomacosmethis* species that were examined for radular traits possess unique, simple teeth (Fig. 4H, I). However, a similar type of radula has been found in *A. balingensis*, which could be classified as *Pincerna* based on the shell shape (round whorls). For this reason, *A. balingensis* is here moved to *Stomacosmethis*. This example suggests that *Pincerna* and *Stomacosmethis* might not be mutually monophyletic. Further examinations are necessary to verify the generic position of *Pincerna* species.

(7) One of the most important results of this study is discovery of distinct differences between *Alycaeus* and *Stomacosmethis*, which form two groups without taxa that exhibit overlapping traits of morphology.

## Annotated list of species-group taxa

In this study we list 412 species-group names including eleven replacement names (seven of them proposed in this study) and five *nomina nuda* (Table 4). Types of 336 species and subspecies (85%) were examined, and of 19 taxa non-type specimens were examined (5%), whilst we relied on the sufficiently detailed original descriptions of the 22 taxa (6%). For 17 taxa (4%) no material was available to be examined in this study.

**Table 4.** List of all alycaeid species-group names. Subgenera are treated at the same level as genera in the “previous classification”. Previous classification does not include our papers (Páll-Gergely 2017, Páll-Gergely & Asami 2017, Páll-Gergely et al. 2017, Páll-Gergely & Auffenberg 2019). Abbreviations for species examined: DOD: detailed original description was sufficient for generic placement; NE: not examined; NT: non-type material; T: types.

Taxon	Previous classification	This study	Remarks	Rank (species or subspecies)	Specimens examined
* abdoui *	* Dicharax *	* Dicharax *		sp	T
* abei *	* Awalycaeus *	* Dicharax *		sp	NT
* aborensis *	* Chamalycaeus *	* Metalycaeus *		sp	T
* akhaensis *	* Raptomphalus *	* Dicharax *		sp	T
* akioi *	* Cipangocharax *	* Dicharax *		sp	T
* akiratadai *	* Awalycaeus *	* Dicharax *		sp	T
* akyabensis *	* Alycaeus *	* Dicharax *		ssp	T
*alticola* Foon & Liew, 2017	* Alycaeus *	* Stomacosmethis *		ssp	DOD
*alticola* Páll-Gergely & Hunyadi, 2017	* Dicharax *	* Dicharax *		sp	T
* altispirus *	* Alycaeus *	* Stomacosmethis *		sp	T
* amphora *	* Dioryx *	* Dioryx *		sp	T
* ananensis *	* Cipangocharax *	* Dicharax *		sp	DOD
* anapetes *			nomen nudum		
* anceyi *	* Alycaeus *	* Pincerna *		sp	T
* andamaniae *	* Chamalycaeus *	* Chamalycaeus *		sp	T
* anghamiensis *	* Dioryx *	* Dioryx *		ssp	T
* anonymus *	* Alycaeus *	* Dicharax *		sp	T
* anthostoma *	* Dicharax *	* Dicharax *		sp	T
* armillatus *	* Dicharax *	* Chamalycaeus *		sp	T
* asaluensis *	* Dicharax *	* Dicharax *		sp	T
* ataranensis *	* Dicharax *	* Dicharax *		sp	T
* avae *	* Dicharax *	* Dicharax *		sp	T
* awaensis *	* Chamalycaeus *	* Metalycaeus *		sp	T
* awalycaeoides *	* Metalycaeus *	* Metalycaeus *		sp	T
* bacca *	* Dioryx *	* Dioryx *		sp	T
* balingensis *	* Alycaeus *	* Stomacosmethis *		sp	T
* barowliensis *	* Alycaeus *	* Dicharax *		sp	T
* bawai *	* Dicharax *	* Dicharax *		sp	T
* beddomei *	* Alycaeus *	* Metalycaeus *		sp	T
* bembex *	* Cycloryx *	* Pincerna *		sp	T
* bhutanensis *	* Chamalycaeus *	* Metalycaeus *		sp	T
* bicrenatus *	* Dicharax *	* Dicharax *		sp	T
* biexcisus *	* Cipangocharax *	* Dicharax *		sp	T
* bifrons *	* Dicharax *	* Dicharax *		sp	T
* birugosus *	* Dicharax *	* Dicharax *		sp	T
* bison *	* Dicharax *	* Dicharax *		sp	T
* blanfordi *	* Alycaeus *	* Dicharax *		sp	T
* brahma *	* Chamalycaeus *	* Metalycaeus *		sp	T
* broti *			nomen nudum		
* burrailensis *	* Cycloryx *	* Pincerna *		sp	T
* burroiensis *	* Cycloryx *	* Dicharax *		sp	T
* burtii *	* Alycaeus *	* Metalycaeus *		sp	T
* busbyi *	* Chamalycaeus *	* Chamalycaeus *		sp	T
* calopoma *	* Alycaeus *	* Stomacosmethis *		sp	DOD: detailed original description was sufficient for generic placement; NE
* canaliculatus *	* Chamalycaeus *	* Chamalycaeus *		sp	T
* canaliculus *	* Chamalycaeus *	* Dicharax *	junior synonym of *birugosus*	sp	T
* candrakirana *	* Dicharax *	* Dicharax *		sp	DOD
* carinatus *	* Alycaeus *	* Stomacosmethis *		sp	NT
* cariniger *	* Dioryx *	* Dioryx *		sp	T
* caroli *	* Chamalycaeus *	* Metalycaeus *		sp	T
* caudapiscis *	* Dicharax *	* Dicharax *		sp	T
* celebensis *	* Chamalycaeus *	* Chamalycaeus *		sp	T
* chanjukensis *	* Alycaeus *	* Metalycaeus *	junior synonym of *brahma*	sp	T
* chaperi *	* Alycaeus *	* Alycaeus *	junior synonym of *gibbosulus*	sp	DOD: detailed original description was sufficient for generic placement; NE
* charasensis *	* Alycaeus *	* Stomacosmethis *		ssp	DOD
* chennelli *	* Dicharax *	* Dicharax *		sp	T
* christae *	* Alycaeus *	* Stomacosmethis *		sp	T
* clementsi *	* Alycaeus *	* Stomacosmethis *		ssp	DOD
* cochinensis *	* Dioryx *	* Dioryx *		sp	T
* commutatus *	* Raptomphalus *	* Metalycaeus *	junior synonym of *brahma*	sp	T
* compactus *	* Dioryx *	* Dioryx *		sp	T
* compressicosta *	* Dicharax *	* Metalycaeus *	junior synonym of *heudei*	syn	T
* conformis *	* Alycaeus *	* Alycaeus *		sp	T
* congener *	* Alycaeus *	* Stomacosmethis *		sp	T
* conicus *	* Alycaeus *	* Dicharax *		sp	T
* constrictus *	* Cycloryx *	* Pincerna *		sp	T
* costacrassa *	* Alycaeus *	* Stomacosmethis *		ssp	DOD
* costata *	* Cycloryx *	* Pincerna *		sp	T
* costulosa *	* Alycaeus *	* Pincerna *		sp	T
* crassicollis *	* Chamalycaeus *	* Dicharax *		sp	DOD: detailed original description was sufficient for generic placement; NE
* crassus *	* Dicharax *	* Dicharax *		ssp	T
* crenatus *	* Dicharax *	* Dicharax *		sp	T
* crenilabris *	* Alycaeus *	* Pincerna *		sp	T
* crenulatus *	* Dicharax *	* Metalycaeus *		sp	T
* crispatus *	* Dicharax *	* Dicharax *		sp	T
* cristatus *	* Dicharax *	* Dicharax *		sp	T
* cucullatus *	* Dicharax *	* Dicharax *		sp	T
* cyclophoroides *	* Chamalycaeus *	* Dicharax *		sp	T
* cyphogyrus *	* Chamalycaeus *	* Metalycaeus *		sp	T
*daflaensis* Godwin-Austen, 1876	* Dicharax *	* Dicharax *		sp	T
*daflaensis* Godwin-Austen, 1914	* Dioryx *	* Dioryx *	replaced by *Dioryx urnula niosiensis* nom. nov.	ssp	T
* dalingensis *	* Dicharax *	* Dicharax *		sp	T
* damsangensis *	* Dicharax *	* Dicharax *		sp	T
* dautzenbergi *	* Dioryx *	* Dioryx *	replacement name for *major* Bavay & Dautzenberg, 1900	sp	T
* davisi *	* Chamalycaeus *	* Dicharax *		sp	T
* degenerans *	* Alycaeus *	* Stomacosmethis *		ssp	T
* depressus *	* Alycaeus *	* Dicharax *		sp	T
* diagonius *	* Dicharax *	* Dicharax *		sp	T
* difficilis *	* Cycloryx *	* Pincerna *		sp	T
* digitatus *	* Dicharax *	* Dicharax *		sp	T
* dihingensis *	* Cycloryx *	* Pincerna *		ssp	T
* dikrangensis *	* Alycaeus *	* Metalycaeus *		sp	T
* diminutus *	* Chamalycaeus *	* Dicharax *		sp	T
* diplochilus *	* Chamalycaeus *	* Dicharax *		sp	T
* distinctus *	* Chamalycaeus *	* Metalycaeus *		sp	T
* distortus *	* Dioryx *	* Dioryx *		sp	T
* ditaceus *	* Sigmacharax *	* Dicharax *		ssp	T
* diyungensis *	* Dicharax *	* Dicharax *		ssp	T
* dohertyi *	* Dicharax *	* Dicharax *		sp	DOD: detailed original description was sufficient for generic placement; NE
* dohrni *	* Alycaeus *	* Stomacosmethis *		sp	T
* dolichodeiros *	* Chamalycaeus *	* Dicharax *		sp	DOD: detailed original description was sufficient for generic placement; NE
* dolomiticus *	* Alycaeus *	* Metalycaeus *	junior synonym of *rathouisianus*	syn	T
* dongiensis *	* Dioryx *	* Dioryx *		sp	T
* draco *	* Dicharax *	* Dicharax *		sp	T
* duoculmen *	* Raptomphalus *	* Dicharax *		sp	T
* duorugosus *	* Dicharax *	* Dicharax *		sp	T
* duplicatus *	* Chamalycaeus *	* Dicharax *		sp	T
* edei *	* Chamalycaeus *	* Dicharax *		sp	T
* elegans *	* Cycloryx *	* Pincerna *		sp	T
* elevatus *	* Alycaeus *	* Dicharax *		sp	T
* ellipticus *	* Dicharax *	* Dicharax *		sp	T
* everetti *	* Dicharax *	* Chamalycaeus *		sp	T
* excisus *	* Chamalycaeus *	* Chamalycaeus *		sp	T
* expanstoma *	* Chamalycaeus *	* Dicharax *		sp	T
*expansus* Foon & Liew, 2017	* Alycaeus *	* Stomacosmethis *		ssp	DOD
*expansus* Heude, 1890	* Alycaeus *	* Metalycaeus *	junior synonym of *muciferus*	syn	T
* expatriatus *	* Chamalycaeus *	* Dicharax *		sp	T
* eydouxi *	* Alycaeus *	* Alycaeus *	replacement name for *C. gibbum* Draparnaud, 1805	sp	NT
* fargesianus *	* Chamalycaeus *	* Dicharax *		sp	T
* feddenianus *	* Dioryx *	* Dioryx *		sp	NT
* fimbriatus *	* Dicharax *	* Dicharax *		sp	T
* footei *	* Dicharax *	* Dicharax *		sp	T
* fractus *	* Dicharax *	* Metalycaeus *	junior synonym of *heudei*	syn	T
* fraterculus *	* Dicharax *	* Dicharax *		sp	T
* fruhstorferi *	* Chamalycaeus *	* Chamalycaeus *		sp	T
* fultoni *	* Alycaeus *	* Stomacosmethis *		sp	T
* galbanus *	* Alycaeus *	* Stomacosmethis *		sp	T
* gemma *	* Dicharax *	* Dicharax *		sp	T
* gemmula *	* Dicharax *	* Dicharax *		sp	T
* generosus *	* Cycloryx *	* Dicharax *		sp	T
* gibbosulus *	* Alycaeus *	* Alycaeus *		sp	T
* gibbus *	* Alycaeus *	* Alycaeus *	replaced by *eydouxi*	sp	NT
* glaber *	* Dicharax *	* Dicharax *		sp	T
*globosus* Godwin-Austen, 1914	* Dioryx *	* Dioryx *	replaced by *rotundus* nom. nov.	ssp	T
*globosus* H. Adams, 1870	* Alycaeus *	* Pincerna *		sp	T
* globuloides *	* Dioryx *	* Dioryx *		sp	T
* globulosus *	* Dioryx *	* Dioryx *		sp	T
* globulus *	* Dicharax *	* Dicharax *		sp	T
*godwinausteni* nom. nov.	* Alycaeus *	* Metalycaeus *	nom. nov. pro *A. neglectus* Godwin-Austen, 1914	sp	T
* granum *	* Cycloryx *	* Pincerna *		sp	T
* graphiaria *	* Cycloryx *	* Pincerna *		sp	T
* graphica *	* Cycloryx *	* Pincerna *		sp	T
* habiangensis *	* Dicharax *	* Dicharax *		sp	T
* harimensis *	* Chamalycaeus *	* Dicharax *	junior synonym of *japonicus*	syn	T
* hebes *	* Dicharax *	* Dicharax *		sp	NT
* helicodes *	* Alycaeus *	* Metalycaeus *	junior synonym of *muciferus*	syn	T
* heudei *	* Dicharax *	* Metalycaeus *		sp	T
* hirasei *	* Chamalycaeus *	* Metalycaeus *		sp	T
* hochstetteri *	* Alycaeus *	* Stomacosmethis *	junior synonym of *jagori*	syn	DOD: detailed original description was sufficient for generic placement; NE
* hosei *	* Alycaeus *	* Stomacosmethis *		sp	NT
* huberi *	* Alycaeus *	* Stomacosmethis *	junior synonym of *somnueki*	syn	T
* humilis *	* Dicharax *	* Dicharax *		sp	NT
* hungerfordianus *	* Chamalycaeus *	* Metalycaeus *		sp	T
* ibex *	* Metalycaeus *	* Metalycaeus *		sp	T
* ikanensis *	* Alycaeus *	* Stomacosmethis *		ssp	DOD
* imitator *	* Dicharax *	* Dicharax *		sp	T
* immaculatus *	* Dicharax *	* Dicharax *		sp	T
*inflatus* Godwin-Austen, 1874	* Chamalycaeus *	* Metalycaeus *		sp	T
*inflatus* Möllendorff, 1886	* Chamalycaeus *	* Dicharax *	replaced by *moellendorffi*	sp	T
* ingrami *	* Chamalycaeus *	* Dicharax *		sp	T
* itonis *	* Sigmacharax *	* Dicharax *		sp	NT
* jagori *	* Alycaeus *	* Stomacosmethis *		sp	T
* japonicus *	* Chamalycaeus *	* Dicharax *		sp	NT
*jatingaensis* nom. nov.	* Alycaeus *	* Dicharax *	nom. nov. pro *A. nanus* Godwin-Austen, 1914	ssp	T
* jaintiacus *	* Dicharax *	* Dicharax *		sp	T
* jousseaumei *	* Chamalycaeus *	* Alycaeus *		sp	T
*juttingae* nom. nov.	* Alycaeus *	* Pincerna *	nom. nov. pro *laevis* van Benthem Jutting, 1959	ssp	DOD: detailed original description was sufficient for generic placement; NE
* kamakiaensis *	* Alycaeus *	* Metalycaeus *		sp	T
* kapayanensis *	* Alycaeus *	* Stomacosmethis *		sp	T
* kelantanensis *	* Alycaeus *	* Stomacosmethis *		sp	T
* kengtungensis *	* Raptomphalus *	* Metalycaeus *		sp	T
* kessneri *	* Chamalycaeus *	* Chamalycaeus *		sp	T
* kezamaensis *	* Dicharax *	* Dicharax *		sp	T
* khasiacus *	* Dicharax *	* Dicharax *		sp	T
* khunhoensis *	* Cycloryx *	* Pincerna *		sp	T
* kinabaluana *	* Alycaeus *	* Pincerna *		ssp	T
* kiuchii *	* Cipangocharax *	* Dicharax *		sp	T
* kobeltianus *	* Dioryx *	* Dioryx *		sp	T
*korintjiensis* nom. nov.	* Alycaeus *	* Pincerna *	nom. nov. pro *latecostata* van Benthem Jutting, 1959	ssp	DOD: detailed original description was sufficient for generic placement; NE
* koshuensis *	* Chamalycaeus *	* Dicharax *		ssp	NT
* kuekenthali *	* Chamalycaeus *	* Stomacosmethis *		sp	T
* kurauensis *	* Alycaeus *	* Stomacosmethis *		ssp	DOD
* kurodai *	* Metalycaeus *	* Dicharax *	junior synonym of *spiracellum*	syn	T
* kurodatokubeii *	* Chamalycaeus *	* Dicharax *		sp	T
* kurzianus *	* Dicharax *	* Dicharax *		sp	T
* labrirubidum *	* Dioryx *	* Dioryx *		sp	T
* laevicervix *	* Chamalycaeus *	* Metalycaeus *		ssp	T
*laevis* Pilsbry & Y. Hirase, 1909	* Chamalycaeus *	* Metalycaeus *		sp	T
*laevis* van Benthem Jutting, 1959	* Alycaeus *	* Pincerna *	replaced by *juttingae* nom. nov.	ssp	DOD: detailed original description was sufficient for generic placement; NE
* lahupaensis *	* Raptomphalus *	* Dicharax *		sp	T
* laosensis *	* Metalycaeus *	* Metalycaeus *		sp	T
*Pincerna latecostata* van Benthem Jutting, 1959	* Alycaeus *	* Pincerna *	replaced by *korintjiensis* nom. nov.	ssp	DOD: detailed original description was sufficient for generic placement; NE
*latecostatus* Möllendorff, 1882	* Chamalycaeus *	* Metalycaeus *		sp	T
* latestriata *			nomen nudum		
* lectus *	* Dicharax *	* Dicharax *		sp	T
* lenticulus *	* Dicharax *	* Dicharax *		sp	T
* levis *	* Alycaeus *	* Dicharax *		sp	T
* libonensis *	* Chamalycaeus *	* Metalycaeus *		sp	T
* liratula *	* Pincerna *	* Pincerna *		sp	T
* logtakensis *	* Alycaeus *	* Dicharax *		sp	T
* lohitensis *	* Alycaeus *	* Metalycaeus *		sp	T
* longituba *	* Alycaeus *	* Dicharax *		sp	NT
* luyorensis *	* Raptomphalus *	* Metalycaeus *		sp	T
* macgregori *	* Chamalycaeus *	* Metalycaeus *		sp	T
* magnificus *	* Raptomphalus *	* Metalycaeus *		sp	T
* magnus *	* Alycaeus *	* Dicharax *		sp	T
* major *	* Dioryx *	* Dioryx *	replaced by *dautzenbergi*	sp	T
*major* (*granum* var.)	* Cycloryx *	* Pincerna *	senior synonym of *A. mangutensis* Godwin-Austen, 1914	sp	T
* makarsae *	* Dicharax *	* Dicharax *		ssp	T
* mangutensis *	* Cycloryx *	* Pincerna *	junior synonym of A. granum var. major Godwin-Austen, 1893	sp	T
* maolanensis *	* Dioryx *	* Pincerna *		sp	T
* maosmaiensis *	* Alycaeus *	* Dicharax *		sp	T
* margarita *	* Cycloryx *	* Pincerna *		sp	T
* matchacheepiorum *	* Alycaeus *	* Stomacosmethis *		sp	DOD
* mediocris *	* Alycaeus *	* Dicharax *		ssp	T
* melanopoma *	* Metalycaeus *	* Metalycaeus *	junior synonym of *nipponensis*	syn	T
* menglunensis *	* Dioryx *	* Dioryx *		sp	T
* messageri *	* Dioryx *	* Dioryx *		sp	T
* microconus *	* Chamalycaeus *	* Chamalycaeus *		sp	T
* microcostatus *	* Dicharax *	* Dicharax *		sp	T
* microdiscus *	* Chamalycaeus *	* Dicharax *		sp	T
* micropolitus *	* Dicharax *	* Dicharax *		sp	T
* microstoma *	* Alycaeus *	* Alycaeus *	junior synonyom of *sculptilis*	syn	DOD: detailed original description was sufficient for generic placement; NE
* minatoi *	* Metalycaeus *	* Metalycaeus *		sp	T
* minimus *	* Dicharax *	* Dicharax *		ssp	T
*minor* (*birugosus* var.)	* Dicharax *	* Dicharax *	junior synonym of *birugosus*	syn	T
*minor* (*constrictus* var.)	* Cycloryx *	* Pincerna *	junior synonym of *constrictus*	syn	DOD: detailed original description was sufficient for generic placement; NE
*minor* (*graphicus* var.)	* Cycloryx *	* Pincerna *	junior synonym of *graphicus*	syn	DOD: detailed original description was sufficient for generic placement; NE
*minor* (*jagori* var.)	* Alycaeus *	* Stomacosmethis *	junior synonym of *jagori*	syn	DOD: detailed original description was sufficient for generic placement; NE
*minor* (*paviei* var.)	* Alycaeus *	* Metalycaeus *	junior synonym of *heudei*	syn	T
*minor* (*pilula* var.)	* Dioryx *	* Dioryx *	junior synonym of *pilula*	syn	DOD: detailed original description was sufficient for generic placement; NE
*minor* (*vestitus* var.)	* Alycaeus *	* Dicharax *	junior synonym of *vestitus*	syn	DOD: detailed original description was sufficient for generic placement; NE
* mixtus *	* Chamalycaeus *	* Chamalycaeus *		sp	T
* miyazakii *	* Chamalycaeus *	* Dicharax *		sp	NT
* moellendorffi *	* Chamalycaeus *	* Dicharax *	replacement name for *inflatus* Möllendorff, 1886	sp	T
* monadicus *	* Dioryx *	* Dioryx *		sp	T
* montanus *	* Chamalycaeus *	* Dicharax *		sp	T
* mouhoti *	* Alycaeus *	* Pincerna *		sp	T
* muciferus *	* Chamalycaeus *	* Metalycaeus *		sp	T
* multicostulata *	* Cycloryx *	* Pincerna *		sp	T
* multidentatus *			junior synonym of *fimbriatus*	syn	T
* multirugosus *	* Dicharax *	* Dicharax *		sp	T
* muluana *	* Alycaeus *	* Pincerna *		ssp	T
* mundulus *	* Alycaeus *	* Metalycaeus *		sp	T
* muroharai *	* Chamalycaeus *	* Dicharax *		ssp	T
* muspratti *	* Raptomphalus *	* Dicharax *		sp	T
* mutatus *	* Dicharax *	* Dicharax *		sp	T
* nagaensis *	* Chamalycaeus *	* Dicharax *		sp	T
* nakashimai *	* Sigmacharax *	* Dicharax *		sp	T
*nanus* Godwin-Austen, 1914	* Alycaeus *	* Dicharax *	replaced by *jatingaensis* nom. nov.	ssp	T
*nanus* Möllendorff, 1886	* Chamalycaeus *	* Dicharax *	junior synonym of *diminutus*	syn	T
* nattoungensis *	* Alycaeus *	* Dicharax *		ssp	T
*neglectus* Godwin-Austen, 1914	* Alycaeus *	* Metalycaeus *	replaced by *godwinausteni* nom. nov.	sp	T
*neglectus* Heude, 1885	* Chamalycaeus *	* Metalycaeus *	junior synonym of *rathouisianus*	syn	T
* nicobaricus *	* Alycaeus *	* Alycaeus *	junior synonym of *reinhardti*	syn	DOD: detailed original description was sufficient for generic placement; NE
*niosiensis* nom. nov.	* Dioryx *	* Dioryx *	nom. nov. pro *Alycaeus daflaensis* Godwin-Austen, 1914	ssp	T
* nipponensis *	* Chamalycaeus *	* Metalycaeus *		sp	T
* nishii *	* Chamalycaeus *	* Dicharax *		sp	T
* nitidus *	* Chamalycaeus *	* Dicharax *		sp	T
* nongtungensis *	* Dicharax *	* Dicharax *		sp	T
* notatus *	* Dicharax *	* Dicharax *		sp	T
* notus *	* Dicharax *	* Dicharax *		sp	T
* nowgongensis *	* Alycaeus *	* Dicharax *		sp	T
* oakesi *	* Raptomphalus *	* Metalycaeus *		sp	T
* obscurus *	* Dicharax *	* Dicharax *		sp	T
* ochraceus *	* Dicharax *	* Dicharax *		sp	T
* oglei *	* Alycaeus *	* Chamalycaeus *		sp	T
* oharai *	* Metalycaeus *	* Metalycaeus *		sp	T
* okamurai *	* Cipangocharax *	* Dicharax *		sp	NT
* okinawaensis *	* Chamalycaeus *	* Dicharax *		sp	NT
* okuboi *	* Metalycaeus *	* Metalycaeus *		sp	T
* oligopleuris *	* Chamalycaeus *	* Dicharax *		sp	T
* omissus *	* Chamalycaeus *	* Dicharax *		sp	T
* oshimanus *	* Chamalycaeus *	* Dicharax *		sp	T
* otiphorus *	* Cycloryx *	* Pincerna *		sp	NT
* pachitaensis *	* Dicharax *	* Dicharax *		sp	T
* panggianus *	* Alycaeus *	* Metalycaeus *		sp	T
* panshiensis *	* Chamalycaeus *	* Dicharax *		sp	DOD: detailed original description was sufficient for generic placement; NE
* parvulus *	* Chamalycaeus *	* Dicharax *		sp	T
* paucicostata *	* Cycloryx *	* Pincerna *		sp	T
* paviei *	* Alycaeus *	* Metalycaeus *	junior synonym of *heudei*	syn	T
* peilei *	* Dicharax *	* Dicharax *		sp	T
* pentagonus *	* Alycaeus *	* Dicharax *	junior synonym of *anthostoma*	syn	T
* perakensis *	* Alycaeus *	* Stomacosmethis *		sp	T
* perplexus *	* Alycaeus *	* Chamalycaeus *		sp	T
* physis *	* Alycaeus *	* Metalycaeus *		sp	NT
* pilsbryi *	* Chamalycaeus *	* Chamalycaeus *	synonym of *japonicus*	sp	T
* pilula *	* Dioryx *	* Dioryx *		sp	NT
* pingoungensis *	* Dioryx *	* Dioryx *		sp	T
* pisum *	* Dioryx *	* Dioryx *		ssp	T
* placenovitas *	* Cipangocharax *	* Dicharax *		sp	T
* planorbulus *	* Chamalycaeus *	* Dicharax *		sp	T
* plectocheilus *	* Dicharax *	* Dicharax *		sp	T
* plicilabris *	* Chamalycaeus *	* Dicharax *		sp	T
* pocsi *	* Dioryx *	* Dioryx *		sp	T
* politus *	* Alycaeus *	* Dicharax *		sp	T
* polygonoma *	* Dicharax *	* Metalycaeus *		sp	T
* porcilliferus *	* Alycaeus *	* Stomacosmethis *		sp	T
* praetextus *	* Alycaeus *	* Stomacosmethis *		sp	T
* pratatensis *	* Alycaeus *	* Dicharax *		sp	T
* principalis *	* Chamalycaeus *	* Dicharax *		ssp	T
* prosectus *	* Dicharax *	* Metalycaeus *		sp	T
* purus *	* Chamalycaeus *	* Dicharax *		sp	T
* pusillus *	* Alycaeus *	* Dicharax *		sp	T
* pygmaea *	* Alycaeus *	* Pincerna *		ssp	T
* pyramidalis *	* Alycaeus *	* Alycaeus *		sp	T
* quadrasi *	* Chamalycaeus *	* Metalycaeus *		sp	T
* rabongensis *	* Alycaeus *	* Pincerna *		ssp	T
* rarus *	* Chamalycaeus *	* Chamalycaeus *		sp	T
* rathouisianus *	* Chamalycaeus *	* Metalycaeus *		sp	T
* rechilaensis *	* Dicharax *	* Dicharax *		sp	T
* regalis *	* Alycaeus *	* Stomacosmethis *		sp	DOD
*reinhardti* Mörch, 1872	* Alycaeus *	* Chamalycaeus *		sp	T
*reinhardti* Pilsbry, 1900	* Alycaeus *	* Dicharax *	replaced by *pilsbryi*Kobelt 1902	sp	T
* requiescens *	* Alycaeus *	* Dioryx *		sp	T
* reticulatus *	* Alycaeus *	* Chamalycaeus *		sp	T
* richthofeni *	* Dicharax *	* Chamalycaeus *		sp	T
* rimatus *	* Alycaeus *	* Stomacosmethis *		sp	T
* robustus *	* Dicharax *	* Dicharax *		sp	T
* roebeleni *	* Alycaeus *	* Stomacosmethis *		sp	T
* rolfbrandti *	* Alycaeus *	* Alycaeus *		sp	T
* rosea *	* Dioryx *	* Dioryx *		sp	T
* rotundatus *	* Alycaeus *	* Metalycaeus *		sp	T
* rotundus *	* Dioryx *	* Dioryx *	nom. nov. pro *globosus* Godwin-Austen, 1914	ssp	T
* rubinus *	* Alycaeus *	* Metalycaeus *		sp	T
* rugosus *	* Dicharax *	* Metalycaeus *		sp	T
* ruyangensis *	* Dioryx *	* Dioryx *		sp	DOD
* rywukensis *	* Dicharax *	* Dicharax *		ssp	T
* sabangensis *	* Alycaeus *	* Chamalycaeus *		ssp	T
* sadoensis *	* Chamalycaeus *	* Dicharax *		ssp	T
* sadongensis *	* Alycaeus *	* Stomacosmethis *		sp	T
* sandowayensis *	* Chamalycaeus *	* Dicharax *		sp	T
* sarasinorum *	* Stomacosmethis *	* Stomacosmethis *		sp	T
* satsumanus *	* Chamalycaeus *	* Metalycaeus *		sp	T
* scepticus *			nomen nudum		
* sculptilis *	* Chamalycaeus *	* Chamalycaeus *		sp	T
* sculpturus *	* Alycaeus *	* Dicharax *		sp	T
* selangoriensis *	* Alycaeus *	* Stomacosmethis *		ssp	DOD
* semperi *	* Metalycaeus *	* Metalycaeus *		sp	T
* senyumensis *	* Alycaeus *	* Stomacosmethis *		sp	DOD
* serratus *	* Alycaeus *	* Dicharax *		sp	T
* setchuanensis *	* Dioryx *	* Dioryx *		sp	T
* shiibaensis *	* Chamalycaeus *	* Dicharax *		sp	T
* shiosakimasahiroi *	* Awalycaeus *	* Dicharax *		sp	DOD
* shiotai *	* Sigmacharax *	* Dicharax *		ssp	T
* sibbumensis *	* Alycaeus *	* Metalycaeus *		sp	T
* simplicilabris *	* Dicharax *	* Dicharax *	junior synonym of *cristatus*	syn	T
* sinensis *	* Chamalycaeus *	* Metalycaeus *		sp	T
* smithi *	* Chamalycaeus *	* Dicharax *	junior synonym of *cristatus*	syn	T
* solidus *	* Dicharax *	* Dicharax *		ssp	T
* somnueki *	* Alycaeus *	* Stomacosmethis *		sp	DOD
* somwangi *	* Alycaeus *	* Alycaeus *		sp	DOD
* sonlaensis *	* Alycaeus *	* Dicharax *		sp	DOD
* specus *	* Alycaeus *	* Chamalycaeus *		sp	T
* spiracellum *	* Dicharax *	* Dicharax *		sp	T
* spratti *	* Cycloryx *	* Stomacosmethis *		sp	T
* stoliczkii *	* Chamalycaeus *	* Dicharax *		sp	T
* strangulatus *	* Dicharax *	* Dicharax *		sp	NT
* strigatus *	* Chamalycaeus *	* Dicharax *		sp	DOD: detailed original description was sufficient for generic placement; NE
* stuparum *	* Dicharax *	* Dicharax *		sp	T
* stylifer *	* Dicharax *	* Metalycaeus *		sp	T
* subculmen *	* Dicharax *	* Dicharax *		sp	T
* subdigitata *	* Dicharax *	* Dicharax *		ssp	T
* subfossilis *	* Chamalycaeus *	* Chamalycaeus *		sp	T
* subhumilis *	* Dicharax *	* Dicharax *		sp	T
* subinflatus *	* Chamalycaeus *	* Metalycaeus *		sp	T
* sublimus *	* Chamalycaeus *	* Chamalycaeus *		ssp	T
* succineus *	* Dicharax *	* Dicharax *		sp	T
*suhajdai* nom. nov.	* Dioryx *	* Metalycaeus *	nom. nov. pro *Alycaeus varius* Godwin-Austen, 1914	sp	T
* sumatranus *	* Alycaeus *	* Chamalycaeus *		sp	T
* summus *	* Cycloryx *	* Pincerna *		sp	T
* swinhoei *	* Dioryx *	* Dioryx *		sp	T
* sylheticus *	* Alycaeus *	* Dicharax *		sp	T
* tadai *	* Chamalycaeus *	* Dicharax *		sp	NT
* takahashii *	* Chamalycaeus *	* Dicharax *		sp	T
* tanegashimae *	* Chamalycaeus *	* Dicharax *		sp	T
* tanghali *	* Alycaeus *	* Chamalycaeus *		sp	T
* tangmaiensis *	* Chamalycaeus *	* Dicharax *		sp	T
* tangmaiensis *	* Dioryx *	* Dioryx *		sp	T
* tenellus *	* Cycloryx *	* Pincerna *		sp	T
* teriaensis *	* Dicharax *	* Metalycaeus *		sp	T
* theobaldi *	* Dicharax *	* Dicharax *		sp	T
* thieroti *	* Alycaeus *	* Pincerna *		sp	T
* thompsoni *	* Cycloryx *	* Pincerna *		sp	T
* tokunoshimanus *	* Chamalycaeus *	* Dicharax *		sp	T
* tomotrema *	* Chamalycaeus *	* Metalycaeus *		sp	T
* toruputuensis *	* Dicharax *	* Metalycaeus *		sp	T
* trigonostoma *			nomen nudum		
* troglodytes *	* Chamalycaeus *	* Chamalycaeus *		sp	T
* tsushimanus *	* Chamalycaeus *	* Dicharax *		sp	T
* umbonalis *	* Chamalycaeus *	* Dicharax *		sp	T
* urceolus *	* Dioryx *	* Dioryx *		sp	T
* urnula *	* Dioryx *	* Dioryx *		sp	T
* vallis *	* Pincerna *	* Pincerna *		sp	DOD
* vanbuensis *	* Dioryx *	* Pincerna *		sp	T
* variabilis *	* Cycloryx *	* Pincerna *		ssp	T
*varius* Godwin-Austen, 1914	* Dioryx *	* Metalycaeus *	replaced by *suhajdai* nom. nov.	sp	T
*varius* Pilsbry & Y. Hirase 1905	* Chamalycaeus *	* Metalycaeus *		sp	T
* vesica *	* Alycaeus *	* Metalycaeus *		sp	T
* vestitus *	* Alycaeus *	* Dicharax *		sp	T
* vinctus *	* Chamalycaeus *	* Metalycaeus *		sp	T
* virgogravida *	* Alycaeus *	* Stomacosmethis *		ssp	DOD
* vulcani *	* Alycaeus *	* Chamalycaeus *		sp	T
* wilhelminae *	* Alycaeus *	* Stomacosmethis *		sp	DOD
* woodthorpi *	* Dicharax *	* Dicharax *		sp	T
* yamneyensis *	* Alycaeus *	* Metalycaeus *		sp	T
* yanoshigehumii *	* Chamalycaeus *	* Dicharax *		sp	T
* yanoshokoae *	* Awalycaeus *	* Dicharax *		sp	DOD
* yanseni *	* Pincerna *	* Pincerna *		sp	T
* yetayensis *	* Alycaeus *	* Metalycaeus *		ssp	T
* zayuensis *	* Chamalycaeus *	* Metalycaeus *		sp	see remarks
* zhuangiyucuii *	* Alycaeus *	* Metalycaeus *	junior synonym of *heudei*	syn	T

Of the 395 taxa, 32 are considered synonyms, although no recent revision has been undertaken for some more specific geographic areas, such as the Himalaya region. Consequently, 362 species-group taxa (320 species and 43 subspecies) of the Alycaeidae are currently accepted. Twenty-two were described by us in previous publications, and there are 18 species, that were formerly classified in *Cycloryx* that now belong to *Pincerna* due to its synonymy with *Cycloryx*. Of the 323 remaining species (excluding our taxa and *Cycloryx*), 209 (65%) are here classified in a new genus, whilst 114 (35%) remain in their previously classified genus. Most of these changes in generic placement resulted from two reasons. Firstly, morphological traits for generic definitions in the preceding studies were not able to classify the currently recognised taxa in morphologically distinct groups, and thus, probably the genera did not reflect evolutionary relationships. Secondly, the type species of each genus were not examined adequately before assigning a new species in these genera.

Alycaeids possess complex shell morphology compared to many other land snail groups, and exhibit a considerable magnitude of variation between populations, which could be interpreted as intraspecific or interspecific variation. Thus, a lumping approach would recognize much fewer taxa than a splitting approach. We employed the former approach, which would be most appropriate when examining widespread and variable taxa such as *Dicharax cristatus*, *D. fimbriatus* or *Metalycaeus muciferus* (see Páll-Gergely et al. 2017). This would be most practical for systematic handling of enormous morphological variability among the currently available specimens in the Alycaeidae. The ‘splitting’ approach to these groups would have resulted in recognition of twice as many or even more species. For example, Foon and Liew (2017) have described several species from Peninsular Malaysia based on small quantitative differences in shell sculpture and size, which would be appropriate for subspecific distinction. Although the present study did not include revision of northeastern Indian Alycaeidae, we found that some of Godwin-Austen’s species exhibit rather minor differences (*M. brahma* and its two new synonyms, and *D. birugosus*, and its new synonym, *D. canaliculus* are examples). Accordingly, geographic variation of the currently recognized alycaeid species diversity largely stem from difference between the splitting and lumping approaches employed by the authors.

### Superfamily Cyclophoroidea Gray, 1847

Cyclophoridae Gray, 1847: 181.

#### Family Alycaeidae

Taxon classificationAnimaliaGastropodaCyclophoridae

W. T. Blanford, 1864

BFA3C5CC-80A1-546E-8834-C0CCAC32234B

Alycaeinae W.T. Blanford, 1864: 465.
Alycaeinae – Godwin-Austen, 1886: 186. (subfamily of Cyclophoridae); Bouchet and Rocroi 2005: 23, 248; Bouchet et al. 2017: 28, 340. (subfamily of Cyclophoridae)
Alycaeidae – Kobelt & Möllendorff, 1897: 146; Egorov 2013: 33.

##### Diagnosis.

Shell with a complex gas exchange system consisting of a common external sutural tube and several extremely narrow, perpendicularly running microtunnels, formed by the outermost shell layer (Páll-Gergely et al. 2016). Bursa copulatrix connecting to lateral side of ovarium.

##### Remarks.

In the past this group has been treated as a subfamily of the Cyclophoridae, and as a family of its own right. The complex gas exchange system, combined with the unique position of the bursa copulatrix (both are important synapomorphic characters), seems to justify the distinction of this group as an independent family.

#### Genus Alycaeus

Taxon classificationAnimaliaGastropodaCyclophoridae

Gray, 1850

FFC26E6C-5C07-5A99-A4E5-4018F7155DE8

Alycaeus Gray, 1850: 27.
Orthalycaeus L. Pfeiffer, 1876: 57 (partim).
Alycaeus (Alycaeus) – Thiele 1929: 108; Wenz 1938: 478; Egorov 2013: 33.

##### Type species.

**Figure 6.**
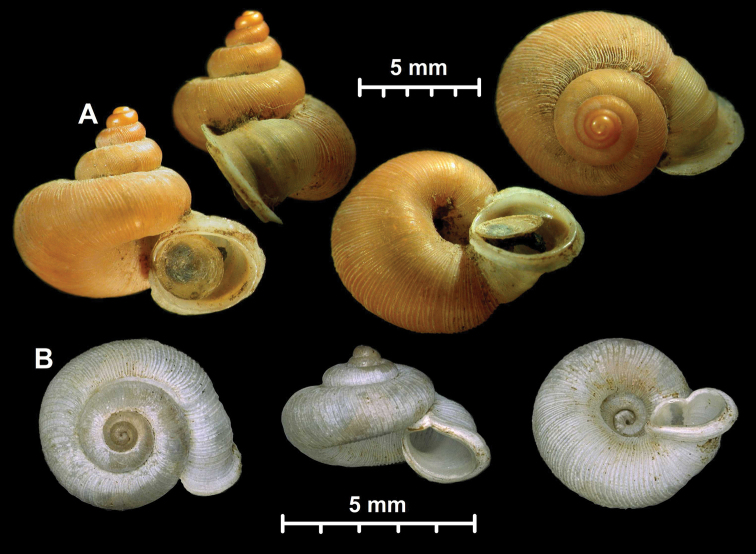
Type species of alycaeid genus-group taxa **A***Alycaeus eydouxi* Venmans, 1956 (SMF 109290; type species of *Alycaeus*) **B***Chamalycaeus fruhstorferi* (Möllendorff, 1897), lectotype (SMF 109481; type species of *Chamalycaeus*). Photographs: Barna Páll-Gergely (**A**) and Frank Walther (**B**).

*Cyclostoma gibbum* Eydoux, 1838 (= *Alycaeus eydouxi* Venmans, 1956)

(Fig. 6A), SD Nevill (1878: 290). *Cyclostoma gibbum* Eydoux, 1838, is a junior homonym of *Cyclostoma gibbum* Draparnaud, 1805. Thus, Venmans (1856) proposed *Alycaeus eydouxi* Venmans, 1956 as a replacement name.

Gray (1850) originally included two species within *Alycaeus* (*A. gibbus* Eydoux, 1838 and *A. strangulatus* L. Pfeiffer, 1846) without selecting either of them to be the type species.

##### Diagnosis.

Shell very large (D: 8–15 mm), triangular, with body whorl being dominant due to very long R2; protoconch smooth, obliquely striated, or transitional character state of the two; R1 usually finely reticulated, due to fine radial ribs and fine spiral striation; R2 long or very long (usually almost reaching 0.5 whorl), smooth or with lamella-like, straight, dense ribs; umbilicus narrow. Operculum thin or relatively thickened (can have both calcareous and proteinaceous layers, Foon and Liew 2017), without elevated outer structure (although scaffold-like calcareous structure and appressed radially spiral lamellae can be present, see Foon and Liew 2017). Central tooth with five cusps, broad, central cusp blunt.

##### Differential diagnosis.

The sculpture of *Alycaeus* and *Chamalycaeus* (smooth protoconch, spirally striated, weakly ribbed teleoconch) are identical, although *Chamalycaeus* tend to have stronger ribs. The distinction is based on the narrow (*Alycaeus*) and wide (*Chamalycaeus*) umbilicus. Furthermore, *Alycaeus* shells are larger, more colourful (reddish or yellowish) and the very long (i.e., ca. 0.5 whorl-long) R2 in *Chamalycaeus* is very rare.

Typical *Pincerna* has a relatively short tube and a strongly ribbed teleoconch, whereas typical *Alycaeus* possesses a long tube and its teleoconch is weakly ribbed. Some species (*P. anceyi*, *P. mouhoti*) form connections between the two genera. However, we prefer to maintain the distinction between *Pincerna* and *Alycaeus* due to the many species characteristic to both respective genera.

*Alycaeus* is easily distinguished differs from *Stomacosmethis* which has a yellowish-orange, triangular shell, and a very short tube.

##### Distribution.

**Figure 7.**
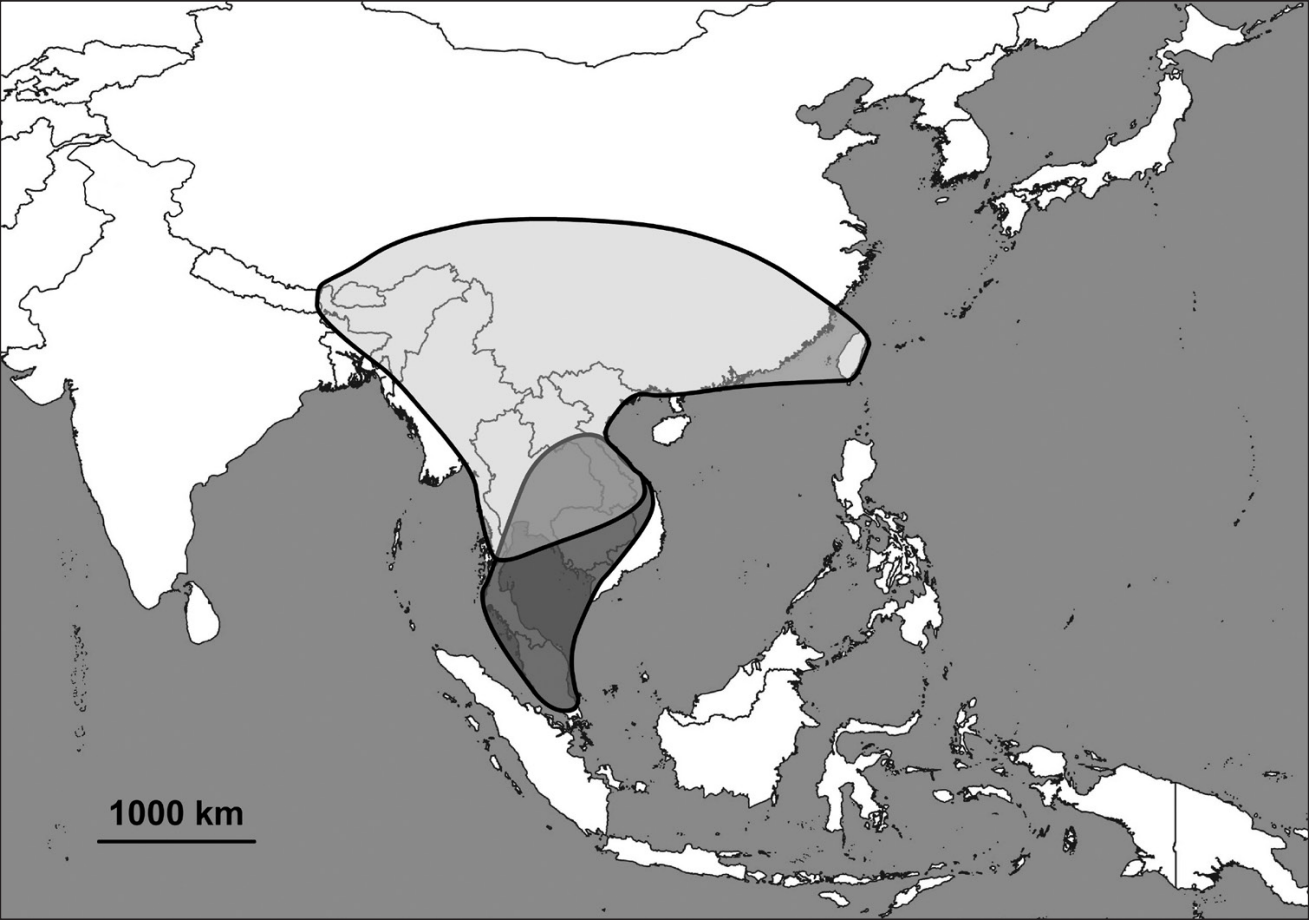
Distribution of *Alycaeus* Gray, 1850 (dark shaded area) and *Dioryx* Benson, 1859 (light shaded area).

This genus is known from northern Laos and northern Vietnam until the southern end of the Malay Peninsula (Fig. 7).

##### Remarks.

Regarding the authorsip of *Alycaeus* (i.e., Baird vs. Gray), we follow Petit (2012: 24–25).

#### Alycaeus conformis

Taxon classificationAnimaliaGastropodaCyclophoridae

Fulton, 1902

837DB68C-0DD0-5883-9C14-30B400006183

Fig. 2C

Alycaeus conformis Fulton, 1902: 68–69.
Alycaeus conformis – Venmans 1956: 81–82, figs 1, 2 (radula, see Results on radula); Páll-Gergely et al. 2016: fig. 2.; Foon and Liew 2017: 30–33, figs 7E, 14, 31D.

##### Type locality.

“Perak”.

##### Material examined.

Perak, NHMUK 1902.5.28.22-23 (2 syntypes); Thailand, Phuket Island, Khao Phra Thaeo Non-hunting Area, Bangpae waterfall, 8°2'6.09"N, 98°23'12.68"E, leg. B. Páll-Gergely & G. Majoros, July 2010, HNHM 99714 (6 shells examined by Páll-Gergely et al. 2016); NHMW 111541 (10 shells, ex NHMW 36649).

##### Remarks.

Protoconch with oblique ribs; R1 densely, finely, regularly ribbed with some very weak spiral striation; R2 very long, with dark and light stripes, the lighter being slightly narrower and more elevated from the surface.

*Alycaeus conformis* and *A. gibbosulus* have a characteristic, oblique striation on the protoconch (Fig. 2). However, the protoconch of *A. rolfbrandti* is also strongly sculptured (mamillated), and at the end of the protoconch oblique striae can be seen. Therefore, there is a continuous transition from the smooth *Alycaeus*-type protoconch sculpture to that of *A. conformis* and *A. gibbosulus*. Due to the similarity in protoconch sculpture and the geographic proximity (they are also found in mixed museum samples), *A. conformis* and *A. gibbosulus* are presumably closely related.

#### Alycaeus eydouxi

Taxon classificationAnimaliaGastropodaCyclophoridae

Venmans, 1956

3C5BDE98-12C5-5B70-BE2E-A6701ED8F079

Figs 2A , 6A , 8

Cyclostoma gibbum Eydoux, 1838: 6, pl. 117, fig 1. (non Cyclostoma gibbum Draparnaud, 1805)
Alycaeus gibbus – Reeve 1878: pl. 1, species 3.
Alycaeus (Alycaeus) gibbus – Kobelt 1902: 344–345.
Alycaeus (Orthalycaeus) gibbus – Godwin-Austen 1914: 427, pl. 156, figs 5, 5a.
Alycaeus eydouxi Venmans, 1956: 87, figs 6, 7 (radula). (nom. nov. pro Cyclostoma gibbum Eydoux, 1838, non Cyclostoma gibbum Draparnaud, 1805)
Alycaeus eydouxi – Egorov 2013: fig. 58a; Páll-Gergely et al. 2017: 9–10, fig. 3A.

##### Type locality.

“que dans les grottes formées dans l’intérieur des montagnes de marbre qui s’élèvent au milieu de la plaine oú est bâtie la ville de Turanne, en Cochinchine”.

##### Material examined.

Annam, Touranne, leg. Frühstorfer, coll. Möllendorff, SMF 109290 (5 shells); Same data, NHMW 43182 (4 shells); Cochinchina, coll. V.W. MacAndrew, NHMUK.

##### Remarks.

Protoconch matte, without spiral lines; R1 fine, dense, rather regular ribs with weak spiral striation; R2 long, with dense, lamellae-like, elevated ribs, which are most elevated closer to the suture. Below the ribs, the microtunnels are visible as narrow light bands between the darker, thicker stripes (visible where there are weathered areas of the shell).

Habe (1965) reported “*Dioryx gibbus* (Reeve)” from “Kao Phlong, north or Sara Buri, Central Thailand”, without publishing a picture. This almost certainly refers to a different species.

**Figure 8.**
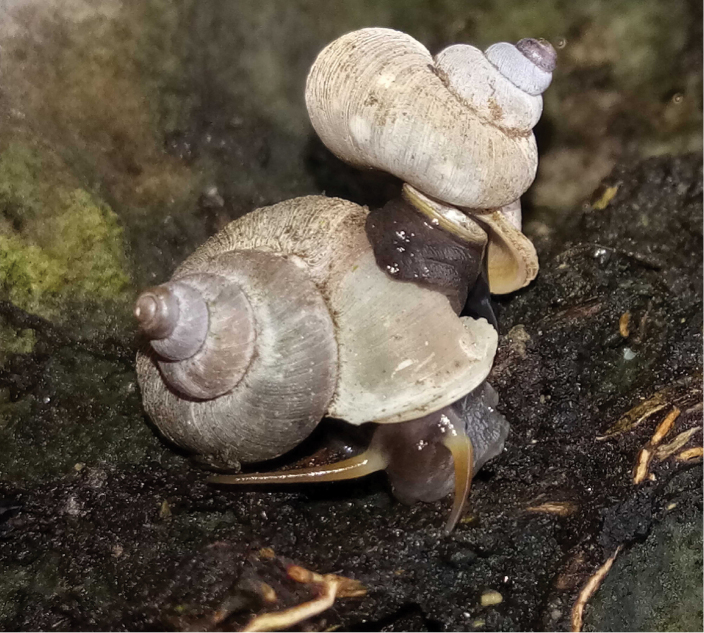
Living specimens of *Alycaeus eydouxi* Venmans, 1856. Thủy Sơn (Water Mountain), Ngũ Hành Sơn (Five Elements or Marble Mountains), Da Nang. 16°0.254’N, 108°15.756’E. Photograph: Junn Kitt Foon.

#### Alycaeus gibbosulus

Taxon classificationAnimaliaGastropodaCyclophoridae

Stoliczka, 1872

6888960D-60CF-5CE0-9FB8-34749CE890D2

Alycaeus gibbosulus Stoliczka, 1872: 268–269, pl. 10, fig. 14.
Alycaeus chaperi de Morgan, 1885a: 70.
Alycaeus (Orthalycaeus) gibbosulus – Möllendorff 1891: 342.
Alycaeus (Alycaeus) gibbosulus – Kobelt 1902: 344.
Alycaeus gibbosulus – Berry 1963: 17, pl. 4, fig. 25; Foon and Liew 2017: 38–41, figs 7F, G, 17, 31E.
Dioryx pyramidalis – Habe 1965: 111–112, pl. 2, figs 3, 4.

##### Type locality.

“Penang island” (from the title).

##### Material examined.

Penang, coll. Dr. Stoliczka, NZSI M.24998 (1 syntype); Perak, Kwala Kangsar ex coll. Grübauer, NHMW 36649 (7 shells, other 10 shells are *A. conformis*: NHMW 111541).

##### Remarks.

Shell sculpture as in *A. conformis*. The types of *A. chaperi* were not examined by us. We follow Möllendorff (1886, 1891) and Foon and Liew (2017) in treating it as a synonym of *A. gibbosulus*.

Habe’s (1965) record of this species from “Khao Chong, Trang Province, peninsular Thailand” almost certainly refers to *Alycaeus gibbosulus*.

#### Alycaeus jousseaumei

Taxon classificationAnimaliaGastropodaCyclophoridae

de Morgan, 1885

AB74F79B-5352-5036-AE42-33546C527181

Alycaeus jousseaumei de Morgan, 1885b: 402, pl. 8, fig. 4.
Alycaeus jousseaumi [sic] – Möllendorff 1891: 343.
Alycaeus (Chamalycaeus) jousseaumei – Kobelt 1902: 357.
Chamalycaeus jousseaumei – Berry 1963: 17, pl. 4, fig. 25.
Alycaeus jousseaumei – Egorov 2013: 33, fig. 58d; Foon and Liew 2017: 43–46, figs 7H, I, 19, 31F.

##### Type locality.

“sur le mont Lano, pres de Campong Kapayan”.

##### Material examined.

Mont Lano, Perak, MNHN-IM-2000-31800 (1 syntype), MNHN-IM-2000-31800 (4 syntypes); Perak, NHMW 41001 (1 shell).

##### Remarks.

Spire low, but upper whorls (not only the protoconch) elevated; Protoconch glossy, R1 with very weak, irregular growth lines and even weaker, fine spiral striation; R2 very long, with wider darker stripes and lighter, narrower channels between, the channels are somewhat elevated from the surface.

#### Alycaeus pyramidalis

Taxon classificationAnimaliaGastropodaCyclophoridae

Benson, 1856

652C7B44-50B6-553A-A4BE-0E24E621F6EC

Alycaeus pyramidalis Benson, 1856: 225.
Alycaeus pyramidalis – Reeve 1878: pl. 1, species 6; Godwin-Austen 1914: 427, pl. 156, figs 6, 6a; Gude 1921: 216.
Alycaeus (Alycaeus) pyramidalis – Kobelt 1902: 348–349.

##### Type locality.

“ad collem Therabuin, vallis Tenasserim”.

##### Material examined.

Therabuin of Therapen (?) hill in Tenasserim, NHMUK 1888.12.4.937–938 (2 possible syntypes); No locality data, UMZC I.102830 (2 possible syntypes).

##### Remarks.

The two syntypes in the NHM and one of the shells from Cambridge were weathered. The third shell from Cambridge is in a good state, and its sculpture could be examined. Protoconch without particular sculpture, rather matte; R1 with low, irregular growth ridges; R2 relatively short, but much longer than typical in *Stomacosmethis*, the surface is irregularly wrinkled, and possibly ribbed near the suture.

Habe’s (1965) record of this species from “Khao Chong, Trang Province, peninsular Thailand” refers to *Alycaeus gibbosulus*.

#### Alycaeus rolfbrandti

Taxon classificationAnimaliaGastropodaCyclophoridae

Maassen, 2006

A8258F43-D119-560F-80E5-48AAF61749EE

Fig. 2B

Alycaeus rolfbrandti Maassen, 2006: 136–137, figs 6–9.
Alycaeus rolfbrandti – Páll-Gergely et al. 2017: 10, fig. 3B; Inkhavilay et al. 2019: 13, fig. 3F.

##### Type locality.

“Laos, limestone Hills 20 km E of Takek”.

##### Material examined.

Laos, Kalkberge ca. 20 km östl. Takek, leg. Brandt 08.09.1963, SMF 262541 (1 shell; labelled as the holotype of “*Alycaeus carinatus* Brandt”, but not mentioned by Maassen 2006); locality data as above, SMF 262541 (5 shells, labelled as paratypes of “*Alycaeus carinatus* Brandt”, but not mentioned in Maassen 2006); South-Central Laos, Khammouan Province, ca. 9 km NE of Thakhek (Muang Khammouan), NW exposition cliff, limestone, clay, black soil in limestone pockets, on and under rocks in dry secondary forest on and under, alt. 190 m, 17°26.757'N, 104°52.937'E, leg. Abdou, A. & Muratov, I.V., 27.11.2007., MNHN-IM-2012-27321 (19 complete shells + some shell fragments).

##### Remarks.

Protoconch irregularly ribbed, squamous, the last ca. 0.25 whorl with oblique ribs similar to those of *A. conformis* and *A. gibbosulus*; R1 with regular, fine, low ribs without spiral striae; R2 long with dense, lamella-like ribs (very similar to those of *A. eydouxi*).

The shells in the Senckenberg Museum are part of the original series of the species collected by Brandt, but since Maassen did not state that he examined them, they are not part of the type series.

#### Alycaeus somwangi

Taxon classificationAnimaliaGastropodaCyclophoridae

Dumrongrojwattana & Maassen, 2008

2C9D2535-B82E-5339-B606-C22008EC5E82

Alycaeus somwangi Dumrongrojwattana & Maassen, 2008: 1–3, figs 1–6.

##### Type locality.

“Thailand, Lub Lae Cave, an isolated limestone hill in Chonburi Province at 13°07'16"N, 101°36'05"E”.

##### Remarks.

We were unable to examine shells of *Alycaeus somwangi*, but the original description provides enough information to allow for generic placement. Protoconch without spiral striae, R2 very long, with regular, low ribs.

#### Genus Chamalycaeus

Taxon classificationAnimaliaGastropodaCyclophoridae

Möllendorff, 1897

6BF61B61-D6F3-59F3-863D-E41A069DC49A

Alycaeus (Chamalycaeus) Möllendorff, 1897b: 93.
Chamalycaeus – Kobelt and Möllendorff 1897: 148; Páll-Gergely et al. 2017: 5–7.
Chamalycaeus (Chamalycaeus) – Thiele 1929: 107–108; Wenz 1938: 477–478; Egorov 2013: 35.

##### Type species.

Alycaeus (Chamalycaeus) fruhstorferi (Fig. 6B) by monotypy, see also Remarks.

##### Diagnosis.

Shell very small to medium sized (D: 2–5 mm), usually flattened, discoid or low triangular, protoconch smooth (or very finely pitted), elevated even if the spire is low; R1 usually roughly reticulated due to spiral striation and radial ribs (sometimes prominent); R2 from short to very long, with widely spaced, sharp, elevated ribs; R3 normally developed. Operculum usually thin, without notable outer structures. Radula is known for a single species (central tooth with five cusps, broad, central cusp pointed).

##### Differential diagnosis.

See under *Alycaeus* and Table 3. *Metalycaeus* species are identical, with the exception of the spirally striated protoconch.

##### Distribution.

**Figure 9.**
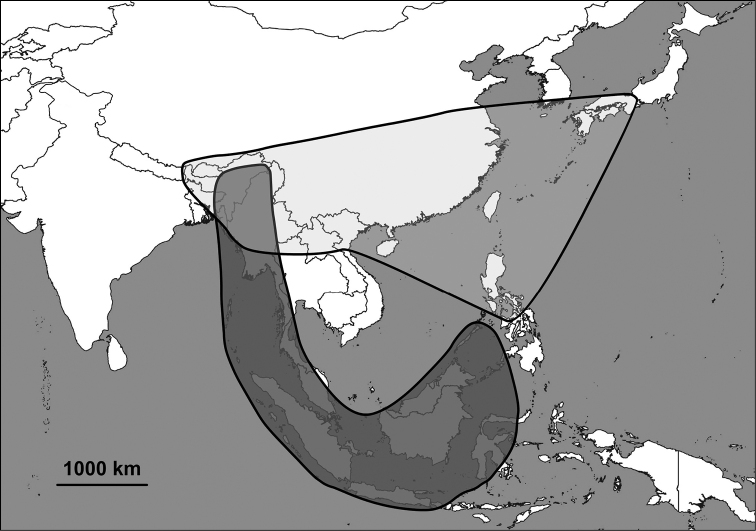
Distribution of *Chamalycaeus* Möllendorff, 1897 (dark shaded area) and *Metalycaeus* Pilsbry, 1900 (light shaded area).

*Chamalycaeus* is distributed from the southeastern Himalaya Region, the Malay Peninsula, Sumatra, Java, Borneo, Sulawesi, and the Philippine Palawan Island (Fig. 9).

##### Remarks.

Kobelt and Möllendorff (1897) listed the species of “Pneumonopoma”, which included all members of the genus *Alycaeus*. They introduced “Subgenus *Chamalycaeus* n.” (Kobelt and Möllendorff 1897: 148), indicating it as a new subgenus, and included 44 species within their new group. The subgenus Chamalycaeus, however, was previously mentioned in another paper in the same volume of the Nachrichtsblatt der Deutschen Malakozoologischen Gesellschaft, in which Möllendorff (1897b: 93) described Alycaeus (Chamalycaeus) fruhstorferi. Möllendorff’s (1897b) paper was published in July-August, whereas that of Kobelt and Möllendorff (1897) was published in September-October. Accordingly, the genus *Chamalycaeus* was described by Möllendorff (1897) and its type species is Alycaeus (Chamalycaeus) fruhstorferi by monotypy. On the other hand, Alycaeus (Chamalycaeus) fruhstorferi was not listed in Kobelt’s and Möllendorff’s (1897) paper, which indicates that they aimed to describe *A. fruhstorferi* after their revision of *Alycaeus*. Moreover, Kobelt’s (1902) monograph referred to *Chamalycaeus* as it was introduced by Kobelt and Möllendorff (1897). Almost all subsequent treatments erroneously attributed the name *Chamalycaeus* to Kobelt & Möllendorff, 1897 (Kobelt 1902; Gude 1921; Yen 1939; Zilch 1957; Azuma 1980; Minato 1982b, 1987a, 2005; Minato and Yano 1988; Egorov 2005; Zhang et al. 2008) and referred to *Alycaeus andamaniae* Benson, 1861 as the type species as subsequent designated by Gude (1921). The ICZN Code 70.2 states the following: “If it is found that an earlier type species fixation has been overlooked, the overlooked fixation is to be accepted and any later fixations are invalid. If this is considered to cause instability or confusion the case is to be referred to the Commission for a ruling”. Therefore, we must examine whether the correction of the type fixation would cause instability. In our view, confusion or instability would be caused only if the majority of authors who have described species within *Chamalycaeus* were unaware of the shell morphology of *Alycaeus andamaniae* (incorrectly selected as the type species for *Chamalycaeus*). No detailed description of *Alycaeus andamaniae* has ever been published, and our revision suggests that most authors who described *Chamalycaeus* species did not examine samples of *Alycaeus andamaniae*. Thus, we find no reason to present this issue to the Commission. Instead, we follow Egorov (2013) in accepting Möllendorff (1897b) as the author of *Chamalycaeus*. Thus, in accordance with Art. 70.2 of the Code we clarify that Alycaeus (Chamalycaeus) fruhstorferi is the type species of *Chamalycaeus* Möllendorff, 1897 by monotypy.

#### Chamalycaeus andamaniae

Taxon classificationAnimaliaGastropodaCyclophoridae

(Benson, 1861)

FEFD001D-63D2-5CCA-A735-31508704D77A

Alycaeus andamaniae Benson, 1861: 28–29.
Alycaeus andamaniae – Reeve 1878: pl. 2, species 10; Godwin-Austen, 1914: 430–431.
Alycaeus (Chamalycaeus) andamaniae – Kobelt 1902: 352–353; Gude 1921: 223–224.
Chamalycaeus (Chamalycaeus) andamaniae – Ramakrishna et al. 2010: 52.

##### Type locality.

“ad portum Blair Insulæ Andamanicæ”.

##### Material examined.

Andaman Islands, UMZC I.103175 (holotype [single specimen mentioned in the original description], photographs examined); Camorta, leg. De Roepstorff, NHMUK 1903.7.1.2708 (1 specimen).

##### Remarks.

Protoconch elevated, finely granulated, no signs of spiral lines; R1 with equally strong spiral lines and irregular ribs; R2 short, with sharp, widely spaced, lamella-like ribs.

#### Chamalycaeus (?) armillatus

Taxon classificationAnimaliaGastropodaCyclophoridae

(Benson, 1856)

8F791FD6-A80D-53C1-8E27-3EDF3A867692

Alycaeus armillatus Benson, 1856: 227.
Alycaeus armillatus – Reeve 1878: pl. 5, species 38; Godwin-Austen 1914: 406, pl. 151, figs 3, 3a.
Alycaeus (Dicharax) armillatus – Kobelt 1902: 365; Gude 1921: 236–237.

##### Type locality.

“ad Thyet-Mio cum præcedente (= *A. sculptilis*)”.

##### Material examined.

UMZC 102995 (holotype [single specimen mentioned in the original description]).

##### Remarks.

Protoconch low, no spiral striation visible.

The specimen largely matches Benson’s original description, and therefore we consider it to be the holotype. The photographs of that specimen show some signs of spiral striation. However, those striae may be part of the lower shell layers, and not raised threads as in other *Chamalycaeus* species. Consequently, the spiral striae on the holotype of *A. armillatus* may not be homologous with the ones in *Chamalycaeus* species; we would need fresh shells to confirm this. For the time being, we refer to this species as Chamalycaeus (?) armillatus.

The shells labelled as *A. armillatus* in the NHM (Thayet-myo, Pegu, coll. Blanford, NHMUK 1906.4.4.71, 6 shells) belong to another (probably undescribed) *Chamalycaeus* species based on the shorter R3, the shallower constriction between R2 and R3, and the smaller distance between the inner and outer peristomes.

#### Chamalycaeus busbyi

Taxon classificationAnimaliaGastropodaCyclophoridae

(Godwin-Austen, 1893)

965427FD-9AB2-5FB2-BE13-6BC2CBFDB457

Alycaeus busbyi Godwin-Austen, 1893: 595.
Alycaeus busbyi – Godwin-Austen 1897: 5, pl. 63, figs 1, 1a, b; Godwin-Austen 1914: 431.
Alycaeus (Chamalycaeus) busbyi – Kobelt 1902: 353; Gude 1921: 225.
Chamalycaeus busbyi – Subba Rao and Mitra 1991: 26–27, pl. 3, fig. 3.
Chamalycaeus (Chamalycaeus) busbyi – Ramakrishna et al. 2010: 52.

##### Type locality.

“Nicobars”.

##### Material examined.

Nicobars, NHMUK 1894.5.23.2 (1 syntype).

##### Remarks.

Protoconch elevated, with very finely pitted surface, no signs of spiral striae; R1 with irregular ribs and spiral striae of the same strength; R2 short, with regular, straight, sharp ribs.

#### Chmamalycaeus canaliculatus

Taxon classificationAnimaliaGastropodaCyclophoridae

(Möllendorff, 1894)

4C5F0964-B52B-5AD6-8388-9400BCE6CC44

Alycaeus canaliculatus Möllendorff, 1894: 154–155, pl. 16, figs 22, 23.
Alycaeus (Chamalycaeus) canaliculatus – Kobelt 1902: 353.
Chamalycaeus (Chamalycaeus) canaliculatus – Zilch 1957: 142, pl. 5, fig. 4.
Dicharax canaliculatus – Páll-Gergely 2017: 25, fig. 15C.

##### Type locality.

“Samui Islands, Gulf of Siam” (from the title).

##### Material examined.

Golf von Siam: Koh-Samui, coll. Möllendorff, SMF 109468 (lectotype, designated by Zilch 1957); Same data, SMF 109469 (4 paralectotypes).

##### Remarks.

Protoconch low, rather matte, very finely granulated, without spiral lines; R1 densely, rather regularly ribbed, the ribs are quite sharp, there is a hardly visible spiral striation between each of the ribs; R2 short, with ribs curved towards the aperture.

#### Chamalycaeus celebensis

Taxon classificationAnimaliaGastropodaCyclophoridae

(E. von Martens, 1891)

DCBDDFC2-A13C-5F3A-BFA4-D8D8C2DDCBA5

Fig. 10A

Alycaeus celebensis E. von Martens, In: Weber, 1891: 217–218.
Alycaeus (Chamalycaeus) celebensis – Kobelt 1902: 354.

##### Type locality.

“Celebes: Luwu”.

##### Material examined.

Luwu, Celebes, M. Weber, ZMB/MOLL 44738 (photographs of a shell [possible syntype] were examined).

##### Remarks.

Protoconch elevated, no spiral lines visible, although the suture is filled with dirt and the photographs are not of high quality. R1 with strong widely spaced, ribs with a fine spiral striation; R2 with denser, straighter ribs, although this part of the shell was somewhat corroded. This species is placed in *Chamalycaeus* due to the colourless shell and biogeographic location. A closer examination of the protoconch would be important to rule out its affinity with *Metalycaeus*, although the occurrence of that genus in Celebes would be surprising.

**Figure 10.**
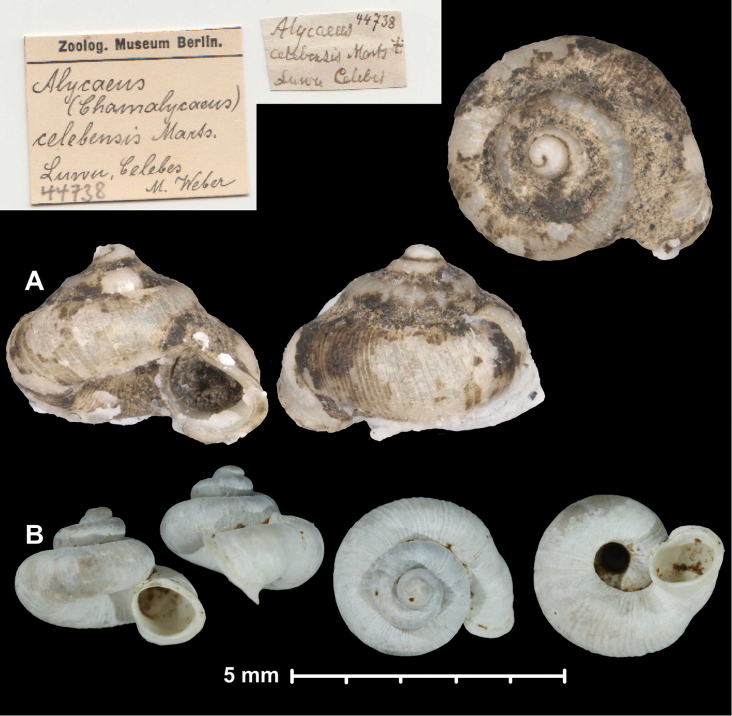
Shells of *Chamalycaeus* Möllendorff, 1897 species **A***Chamalycaeus celebensis* (E. von Martens, 1891), possible syntype (ZMB/MOLL 44738) **B***Chamalycaeus kessneri* Vermeulen, 1996, paratype (SMF 311351). Photographs: Barna Páll-Gergely (**B**) and Christine Zorn (**A**).

#### Chamalycaeus everetti

Taxon classificationAnimaliaGastropodaCyclophoridae

(Godwin-Austen, 1889)

5908F974-537A-55AF-AF81-A4B9820C501D

Alycaeus everetti Godwin-Austen, 1889: 347, pl. 37, figs 5, 5a.
Alycaeus n. sp. – Aldrich 1889: 25, pl. 3, figs 2, 2a, 2b (later mentioned A. broti, but this name was not made available).
Alycaeus everetti – E. A. Smith 1895: 116.
Alycaeus (Dicharax) everetti – Kobelt 1902: 369.
Chamalycaeus (Dicharax) everetti – Zilch 1957: 145.

##### Type locality.

“Niah Hills”

##### Material examined.

Niah Hills, Borneo, NHMUK 1889.12.7.33 (holotype [single specimen mentioned in the original description]).

##### Remarks.

Aldrich (1889) did not give a name for the “*Alycaeus* sp.”, species he figured and described, because he thought it might be *A. spiracellum*, which he was unable examine for comparison. He mentioned that “if new, I propose the name *Alycaeus broti* for it”. This action does not make the name available, because under ICZN Art. 11.5 “To be available, a name must be used as valid for a taxon when proposed”, which was not the case for *A. broti*; therefore, the name *Alycaeus broti* is not available. Smith (1895) mentioned that he compared *A. everetti* specimens with his “*A. broti*”, and they were identical.

Protoconch elevated, no spiral lines visible; R1 with very fine, irregular ribs and spiral lines; R2 short, with sharp, straight, widely spaced ribs.

#### Chamalycaeus excisus

Taxon classificationAnimaliaGastropodaCyclophoridae

(Möllendorff, 1887)

2433B888-0F95-5FBC-9E48-6EA6BDA84A40

Alycaeus excisus Möllendorff, 1887b: 287.
Alycaeus (Chamalycaeus) excisus – Kobelt 1902: 355.
Chamalycaeus (Chamalycaeus) excisus – Zilch 1957: 142, pl. 6, fig. 20.
Chamalycaeus excisus excisus – Páll-Gergely and Auffenberg 2019: 378, figs 2B, 3, 4C, D.

##### Type locality.

“Insel Bongao zwischen Sulu und Borneo” (from the title).

##### Material examined.

Sulu-Inseln, Insel Bongao (Tawi-Tawi-Gr.), leg. Möllendorff 1890, coll. O. Boettger, SMF 109479 (holotype [single adult specimen mentioned in the original description]); Same data, SMF 109480 (4 paratypes).

##### Remarks.

Protoconch elevated without spiral lines; R1 with weak, widely spaced, irregular ribs and somewhat stronger spiral striation; R2 relatively short, with widely spaced, elevated, sharp ribs.

#### Chamalycaeus excisus sublimus

Taxon classificationAnimaliaGastropodaCyclophoridae

Páll-Gergely & Auffenberg, 2019

949F8E23-3F27-5E0A-ACC5-1C0EC0396E8D

Chamalycaeus excisus sublimus Páll-Gergely & Auffenberg, 2019: 381, figs 4A, B, E, F, 6A, B.

##### Type locality.

“Philippine Islands, Palawan Prov., 50 km SW of Quezon, along trail from Ransang to Tau’t Batu Caves, 90–390 m a.s.l., 8°53'N, 117°35'E.”

##### Material examined.

Holotype (UF 115862) and paratypes, see Páll-Gergely and Auffenberg (2019).

##### Remarks.

Same as the nominotypical subspecies.

#### Chamalycaeus fruhstorferi

Taxon classificationAnimaliaGastropodaCyclophoridae

(Möllendorff, 1897)

29BD3774-1C8B-536A-9816-482862D002E8

Fig. 6B

Alycaeus (Chamalycaeus) fruhstorferi Möllendorff, 1897b: 93–94.
Alycaeus (Chamalycaeus) fruhstorferi – Kobelt 1902: 356.
Chamalycaeus fruhstorferi – van Benthem Jutting 1948: 571–572, fig. 26; Páll-Gergely et al. 2017: 7, fig. 46D–F.
Chamalycaeus (Chamalycaeus) fruhstorferi – Zilch 1957: 142, pl. 6, fig. 21.

##### Type locality.

“Java” (from the title).

##### Material examined.

Java, leg. Fruhstorfer, coll. Möllendorff, SMF 109481 (lectotype, designated by Zilch 1957); Same data, SMF 109482 (5 paralectotypes); Mons Gede, 4000’, W. Java, leg. Fruhstorfer, Aug. 1892, E.R. Sykes colln. 1954, NHMUK 20150361 (4 specimens).

##### Remarks.

Protoconch elevated, no spiral lines visible; R1 rather regularly ribbed with sharp ribs, and with somewhat weaker spiral striation; R2 relatively long, with widely spaced, sharp ribs.

#### Chamalycaeus kessneri

Taxon classificationAnimaliaGastropodaCyclophoridae

Vermeulen, 1996

A69E11B1-B069-5504-9170-E515768F7EC5

Fig. 10B

Chamalycaeus kessneri Vermeulen, 1996: 150, fig. 2a–c.
Chamalycaeus kessneri – Vermeulen and Whitten 1998: 46, fig. 23.

##### Type locality.

“Nusa Penida”.

##### Material examined.

Tengasa Monkey Temple, Nusa Penida, Indonesia, 8°45'S, 115°31'E, leg. A.J. Witten, 1993, NHMUK 20000248 (paratype); Indonesia, Nusa Penida, Tengasa Monkey Temple, 8°45'S, 115°31'E, Secondary forest, leg. A.J. Whitten, 1993, ex coll. J.J. Vermeulen 4080, SMF 311351 (1 paratype). Indonesia, South Kalimantan, Nateh, leg. Yansen Chen, April 2012 (6 shells).

##### Remarks.

The examined paratype was badly weathered, only the elevated protoconch with some spiral lines on R1 and the short tube were visible. Based on these, *C. kessneri* remains classified in the genus *Chamalycaeus*.

The shells from Nateh were considerably smaller than typical *C. kessneri*, but agreed with that species in terms of shell shape, the short tube, and the spiral striation. Although these six shells were also weathered, one of them was in a relatively good condition. None of the shells showed signs of spiral striation on the protoconch, therefore the placement of this species in *Chamalycaeus* seems to be justified.

#### Chamalycaeus microconus

Taxon classificationAnimaliaGastropodaCyclophoridae

(Möllendorff, 1887)

E2C6E00C-029E-5FB8-97B5-7DB8BA27D520

Fig. 11A

Alycaeus microconus Möllendorff, 1887a: 311–312.
Alycaeus microconus – Möllendorff 1891: 343, pl. 30, figs 12, 12a, 12b.
Alycaeus (Chamalycaeus) microconus – Kobelt 1902: 358.
Chamalycaeus (Chamalycaeus) microconus – Zilch 1957: 143, pl. 5, fig. 6.

##### Type locality.

“Ad Buket Pondong”.

##### Material examined.

Malakka: Bukit Pondong (Perak), SMF 109493 (lectotype, designated by Zilch 1957); Same data, SMF 109494 (2 paralectotypes).

##### Diagnosis.

Protoconch rather low, without obvious spiral lines, the granules following a near spiralling arrangement, but not at all similar to the multiple, narrow spiral striae typical to most *Metalycaeus* species; R1 with rather regular ribs and strong spiral lines; R2 extremely short, with only ca. five ribs which are blunt (probably bent?). Operculum unknown.

**Figure 11.**
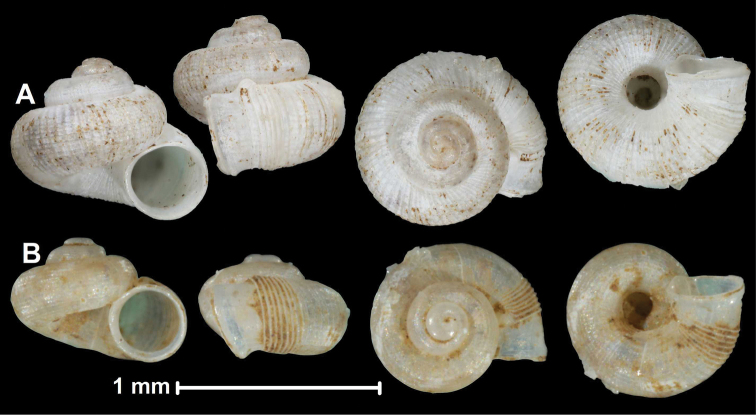
Shells of *Chamalycaeus* Möllendorff, 1897 species **A***Chamalycaeus microconus* (Möllendorff, 1887), lectotype (SMF 109493) **B***C. mixtus* Zilch, 1957, holotype (SMF 109510). All images: Barna Páll-Gergely, courtesy Ronald Janssen.

#### Chamalycaeus mixtus

Taxon classificationAnimaliaGastropodaCyclophoridae

Zilch, 1957

C3A658E0-7946-5572-A995-974470AA895A

Fig. 11B

Chamalycaeus (Chamalycaeus) mixtus Zilch, 1957: 143–144, pl. 5, fig. 7.

##### Type locality.

“Malakka, Bukit Pondong, (Perak)”.

##### Material examined.

Malakka: Bukit Pondong (Perak), SMF 109510 (holotype); Same data, SMF 109511 (4 paratypes).

##### Remarks.

Protoconch as in *C. microconus*; R1 with rather irregular, widely spaced, low ribs, with somewhat stronger spiral striae; R2 extremely short, consists of ca. eight ribs which are bent in the direction of their anterior neighbours.

#### Chamalycaeus oglei

Taxon classificationAnimaliaGastropodaCyclophoridae

(Godwin-Austen, 1914)

F37C80C4-C0C1-5004-B37F-4C24AE888E1E

Alycaeus oglei Godwin-Austen, 1914: 362, pl. 148, fig. 2.
Alycaeus oglei – Gude 1921: 213.
Alycaeus (Alycaeus) oglei – Ramakrishna et al. 2010: 49.

##### Type locality.

“Sadia”; “Dihing, 500 ft”.

##### Material examined.

Noa Dihing, 500 f. (2 shells in the vial) & Sadia, 350 f. (1 shell in the vial), leg. M. Ogle, NHMUK 1903.07.01.2491 (syntypes). The box labelled *A. oglei* contained two glass vials.

##### Remarks.

Protoconch elevated, no spiral lines visible; R1 with rather irregular, low ribs, and weaker spiral striation; R2 very long with widely spaced, sharp ribs.

#### Chamalycaeus perplexus

Taxon classificationAnimaliaGastropodaCyclophoridae

(Godwin-Austen, 1914)

15F2F26B-5C08-5934-88BE-596C8A22DCD8

Alycaeus perplexus Godwin-Austen, 1914: 380, pl. 155, fig. 11.
Alycaeus perplexus – Gude 1921: 214.
Alycaeus (Alycaeus) perplexus – Ramakrishna et al. 2010: 50.

##### Type locality.

“Khasi Hills”.

##### Material examined.

Khasi Hills, coll. Godwin-Austen, NHMUK 1903.7.1.2756. (3 syntypes).

##### Remarks.

Protoconch elevated, no spiral lines visible; R1 irregularly, weakly wrinkled and as strongly spirally striated; R2 moderately long, with wider darker and narrower lighter stripes alternating, the overall surface is nearly smooth, rather irregularly wavy.

#### Chamalycaeus rarus

Taxon classificationAnimaliaGastropodaCyclophoridae

Páll-Gergely & Auffenberg, 2019

94122B88-DEA0-5753-9738-0ACCC004E1E7

Chamalycaeus rarus Páll-Gergely & Auffenberg, 2019: 382, fig. 6C.

##### Type locality.

“Philippine Islands, Palawan Prov., 50 km SW of Quezon, along trail from Ransang to Tau’t Batu Caves, 90–390 m a.s.l., 8°53'N, 117°35'E”.

##### Material examined.

Only the holotype (UF 525657) is known.

##### Remarks.

R1 rather strongly and irregularly ribbed with weaker spiral striation; R2 + R3 short, less than 90° combined; R2 shorter than R3; ribs on R2 lamella-like; spiral striation also visible on R2; R3 with spiral striation and weaker ribs than those on R1.

The placement of this species into the genus *Chamalycaeus* is based on biogeographic information alone, since the protoconch, which is necessary for generic allocation, is absent in the only available shell (Páll-Gergely and Auffenberg 2019).

#### Chamalycaeus reinhardti

Taxon classificationAnimaliaGastropodaCyclophoridae

(Mörch, 1872)

4DD86E08-CAF5-50E8-ADCD-4932F38E7564

Alycaeus (Charax) reinhardi (sic) Mörch, 1872a: 22.
Alycaeus (Charax) reinhardi (sic) – Mörch 1872b: 315.
Alycaeus nicobaricus Reeve, 1878: pl. 4, species 29.
Alycaeus reinhardti – Godwin-Austen 1895: 455; Godwin-Austen 1914: 431; Gude 1921: 216–217.
Alycaeus (Alycaeus) reinhardti – Kobelt 1902: 349; Subba Rao and Mitra 1991: 26, pl. 3, fig. 4.; Ramakrishna et al. 2010: 50.

##### Type locality.

“Bords de la rivière Galathea, sur la terre, sous les feuilles mortes” and “Kar Nicobar”.

##### Material examined.

Great Nicobar, coll. Godwin-Austen, NHMUK 1903.7.1.2711.

##### Remarks.

Spire elevated, shell slightly wider than high; protoconch elevated, no spiral lines visible; R1 with irregular ribs, and somewhat weaker spiral lines; R2 relatively long, with widely spaced, regular, sharp ribs.

The original spelling of the species was *reinhardi*, which was corrected to *reinhardti* by Kobelt (1902). This was a justified emendation under the Article 32.5. of the ICZN Code, because it was obvious that Mörch (1872a) named the new species after the collector Reinhardt.

Godwin-Austen (1895) mentioned that the type of this species is from Great Nicobar Island, and the form from Camorta is named *f. minor* by Mörch. However, we have not found the publication in which Mörch introduced that name.

Reeve’s *A. nicobaricus* was not examined by us, but it was considered to be a junior synonym of *A. reinhardti* by Gude (1921).

#### Chamalycaeus reinhardti sabangensis

Taxon classificationAnimaliaGastropodaCyclophoridae

(B. Rensch, 1933)

348CB263-21DE-5CDA-AC92-AA7966A235A2

Alycaeus reinhardti sabangensis B. Rensch, 1933: 200–201.
Alycaeus (Alycaeus) reinhardti sabangensis – Zilch 1957: 147.

##### Type locality.

“aus dem Walde bei Sabang”.

##### Material examined.

Sumatra: Wald b. Sabang, Pulu Weh., exp. Rensch, 1927, SMF 6241 (1 paratype).

##### Remarks.

Spire elevated, shell slightly wider than high; protoconch elevated, no spiral lines visible; R1 with irregular ribs, and somewhat weaker spiral lines; R2 relatively long, with widely spaced, regular, sharp ribs.

#### Chamalycaeus reticulatus

Taxon classificationAnimaliaGastropodaCyclophoridae

(Möllendorff, 1897)

B0DF1317-D4CB-5E4B-8406-BEA912769CBA

Alycaeus (Orthalycaeus) reticulatus Möllendorff, 1897b: 93.
Alycaeus (Alycaeus) reticulatus – Kobelt 1902: 349; Zilch 1957: 147, pl. 6, fig. 26.
Alycaeus reticulatus – van Benthem Jutting 1948: 570–571.

##### Type locality.

“Java” (from the title).

##### Material examined.

W-Java, Djampang, 2000’, leg. H. Fruhstorfer, 1895, coll. O. Boettger, SMF 57196 (syntype, labelled as holotype, photographs examined).

##### Remarks.

The original description does not mention the number of examined specimens. Thus, we consider the specimen labelled holotype (SMF 57196) as a syntype.

Spire quite elevated, shell approximately as high as it is wide; protoconch very finely granulated, no spiral lines visible; R1 with strong, rather irregular ribs and somewhat weaker spiral striation; R2 short, with widely spaced, sharp ribs.

#### Chamalycaeus richthofeni

Taxon classificationAnimaliaGastropodaCyclophoridae

(W. T. Blanford, 1863)

0A37AD22-1337-51B9-A4AB-96DD473FB75C

Alycaeus richthofeni W.T. Blanford, 1863: 324.
Alycaeus richthofeni – Reeve 1878: pl. 3, species 23; Godwin-Austen 1914: 428, pl. 151, fig. 9.
Alycaeus (Dicharax) richthofeni – Kobelt 1902: 376; Gude 1921: 268.

##### Type locality.

“Molmain”.

##### Material examined.

Tenasserim, Moulmein, NHMUK 1906.5.5.24 (holotype [single specimen mentioned in the original description]).

##### Remarks.

The holotype is strongly weathered. Protoconch strongly elevated, no spiral lines visible; R1 rather regularly ribbed with some weak signs of spiral striation; R2 long, with widely spaced ribs, which were probably sharp in the fresh shell.

#### Chamalycaeus sculptilis

Taxon classificationAnimaliaGastropodaCyclophoridae

(Benson, 1856)

45FAE379-94D6-5EEA-84EF-2AB1E9150911

Alycaeus sculptilis Benson, 1856: 226–227.
Alycaeus sculptilis – Reeve 1878: pl. 4, species 32, figs a, b; Godwin-Austen 1914: 398, 412, pl. 139, figs 7, 7a; pl. 155, fig. 8.
Alycaeus margarita Theobald in Hanley & Theobald, 1874: pl. 97, fig. 7 (renamed A. microstoma by Reeve 1878)
Alycaeus microstoma Reeve, 1878: pl. 4, species 28.
Alycaeus (Chamalycaeus) sculptilis – Kobelt 1902: 362; Gude 1921: 233.
Chamalycaeus (Chamalycaeus) sculptilis – Ramakrishna et al. 2010: 55.

##### Type locality.

“ad Thyet-Mio prope fluvium Irawadi, non procul a finibus provinciæ Burmanicæ Britannicæ”.

##### Material examined.

Bens. col., Thyet Myo”, UMZC I.102845 (3 shells, type status uncertain); Pegu, Thayet-myo, “typical”, “aperture figured”, NHMUK 1906.4.4.70.

##### Remarks.

Protoconch elevated, no spiral lines visible; R1 with rather regular, widely spaced ribs, and somewhat weaker spiral lines; R2 long, with widely spaced ribs; there is a lamella on each rib which is slightly bent in the direction of the anterior neighbour.

Reeve (1878) named the shell figured by Hanley and Theobald (1874) on pl. 97, fig. 7, as *Alycaeus microstoma*, and published drawings (pl. 4, species 28). The type specimens were not examined by us, but that species was considered to be a synonym of *Alycaeus sculptilis* Benson, 1856 by Godwin-Austen (1914).

#### Chamalycaeus specus

Taxon classificationAnimaliaGastropodaCyclophoridae

(Godwin-Austen, 1889)

15D1A6C8-5D65-575A-8192-4113C6BF04C4

Alycaeus specus Godwin-Austen, 1889: 347, pl. 37, figs 4, 4a.
Alycaeus (Alycaeus) specus – Kobelt 1902: 351.

##### Type locality.

“In limestone caves at Jambusan”.

##### Material examined.

Caves, Borneo, leg. A. Everett, coll. Godwin-Austen, NHMUK 1889.12.7.26 (1 syntype).

##### Remarks.

The syntype is weathered and was glued to a piece of black paper by its R2 area, therefore limited information could be gained during its examination. The shell is depressed and conical; protoconch strongly weathered, but there were no signs of spiral striation near the suture; R1 regularly and strongly ribbed with very weak spiral striation; length of R2 could not be fully seen, but has low, dense riblets and fine spiral lines. We received photographs and good quality drawings of newly collected shells from Thor-Seng Liew and Jaap Vermeulen (pers. comm. August 2019), and those confirmed that this species is a *Chamalycaeus* due to the colourless shell, long R2, and relatively strong ribs.

#### Chamalycaeus subfossilis

Taxon classificationAnimaliaGastropodaCyclophoridae

(P. Sarasin & F. Sarasin, 1899)

134C8378-B11B-5AA8-A89C-764CD42CEE3E

Alycaeus subfossilis P. Sarasin & F. Sarasin, 1899: 63–64, pl. 4, figs 46, 46a, pl. 5, fig. 66, pl. 8, fig. 91.
Alycaeus (Chamalycaeus) subfossilis – Kobelt 1902: 363–364.
Chamalycaeus subfossilis – Páll-Gergely and Auffenberg 2019: 378, fig. 2A.

##### Type locality.

“Geröllbank am Limbotto-See”.

##### Material examined.

Limbotto See, NHMB 2265a (lectotype, designated herein), NHMB 2265a' (3 paralectotypes).

##### Remarks.

Protoconch elevated, finely pitted, no spiral lines visible; R1 with dense riblets and some weak spiral striation; R2 short, with somewhat elevated ribs that are similar to those on R1.

Lothar Forcart selected a specimen (NHMB 2265a) and labelled it as the lectotype, but never published this action (Ambros Hänggi, pers. comm. 2020 June). We previously referred to that specimen as a lectotype (Páll-Gergely and Auffenberg 2019); however, that was not a valid lectotype selection. Thus, here we designate the same specimen selected by Locard (Páll-Gergely and Auffenberg 2019: fig. 2a) as the lectotype.

#### Chamalycaeus sumatranus

Taxon classificationAnimaliaGastropodaCyclophoridae

(E. von Martens, 1900)

681691B1-A8D6-5F2B-83DF-FEB6B0878447

Fig. 12A

Alycaeus (Orthalycaeus) sumatranus E. von Martens, 1900: 6–7.
Alycaeus (Alycaeus) sumatranus – Kobelt 1902: 351.
Alycaeus sumatranus – van Benthem Jutting 1959: 77–78, fig. 5.

##### Type locality.

“Unter-Lankat”.

##### Material examined.

Unter-Lankat, coll. Schneider, ZMB/MOLL 51748 (1 syntype, labelled as holotype; photographs examined).

##### Remarks.

The original description does not mention the number of examined specimens. Thus, we consider the specimen labelled holotype (ZMB/MOLL, 51748) as a syntype.

Protoconch elevated, no spiral striation visible; R1 with rather irregular, low ribs and spiral striation roughly of the same strength; R2 long, with widely spaced, sharp ribs.

**Figure 12.**
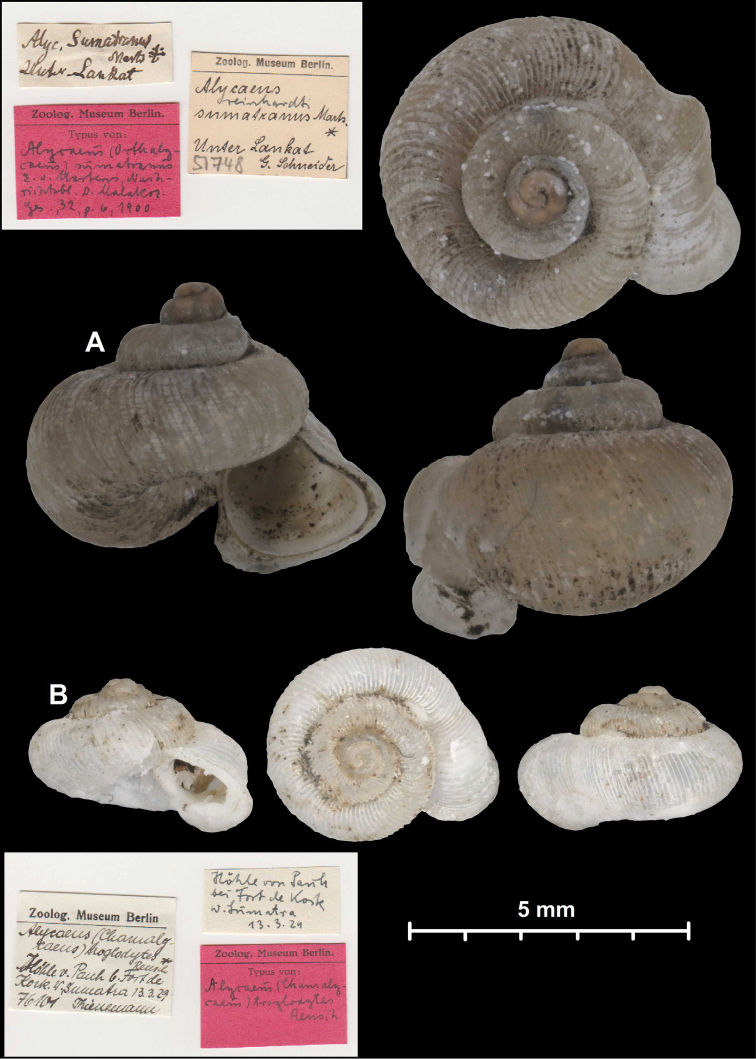
Shells of *Chamalycaeus* Möllendorff, 1897 species **A***Chamalycaeus sumatranus* (Martens, 1900), syntype (ZMB/MOLL 51748) **B***Chamalycaeus troglodytes* (Rensch, 1934), syntype (ZMB/MOLL 76101). Photographs: Christine Zorn.

#### Chamalycaeus tanghali

Taxon classificationAnimaliaGastropodaCyclophoridae

(Godwin-Austen, 1914)

FF367284-9734-50B2-999D-9E3475D0DF4B

Alycaeus tanghali Godwin-Austen, 1914: 401, pl. 137, figs 3, 3a, 3b.

##### Type locality.

“Munipur. Exact locality not recorded; somewhere on the northern side of the valley”.

##### Material examined.

Munipur, figured by Godwin-Austen, NHMUK 1903.7.1.2671 (6 syntypes).

##### Remarks.

Protoconch elevated, but no spiral lines are visible; R1 with widely spaced, regular ribs and fine spiral striation; R2 long, with widely spaced, regular, sharp ribs.

#### Chamalycaeus troglodytes

Taxon classificationAnimaliaGastropodaCyclophoridae

(B. Rensch, 1934)

293535AE-AD86-5931-8A21-AF83364F6C83

Fig. 12B

Alycaeus (Chamalycaeus) troglodytes B. Rensch, 1934: 743–744, fig. 3.

##### Type locality.

“Mittel-Sumatra: Höhle von Pauh bei Fort de Kock”.

##### Material examined.

Mittel-Sumatra: Höhle von Pauh bei Fort de Kock, leg. Thienemann, 13.03.29., ZMB/MOLL 76101 (1 syntype; photographs examined).

##### Remarks.

Protoconch low, no spiral lines visible; R1 with widely spaced, sharp regular ribs and very weak spiral striation; R2 very short, with ribs which are similar to those on R1.

#### Chamalycaeus vulcani

Taxon classificationAnimaliaGastropodaCyclophoridae

(W. T. Blanford, 1863)

FA3E1E03-B3E6-5F0A-A6CE-EE805F22C1AC

Alycaeus vulcani W.T. Blanford, 1863: 323.
Alycaeus vulcani – Reeve 1878: pl. 2, species 17; Godwin-Austen 1914: 413–414, pl. 151, figs 5, 5a; Gude 1921: 221–222.
Alycaeus (Alycaeus) vulcani – Kobelt 1902: 352.

##### Type locality.

“on the upper portion of the isolated peak of Puppa, an extinct volcano lying ca. 40 miles E. S. E. of the town of Pu-gán in the territories of the king of Ava”.

##### Material examined.

Ava, Burma, MCZ 135705 (1 shell, labelled as syntype); Puppadoung, ex coll. Theobald, NHMUK 1888.12.4.939–942 (4 shells, possible syntypes); Puppa, Ava, Burma, coll. H.F. Blanford, ex coll. auctoris, NHMUK (8 shells, possible syntypes); Puppa Hill, Ava, leg. Blanford, Crosse coll. 1899, Sykes coll. 1954, NHMUK (2 shells, possible syntypes).

##### Remarks.

Protoconch rather elevated, without spiral striation; R1 with elevated, regular, sharp ribs and without spiral striation; R2 long with widely spaced, sharp ribs.

The absence of spiral striation on the entire shell is unusual for *Chamalycaeus*, and characteristic for *Dicharax*. However, the general shell shape, the strong, equidistant ribs, and the elevated protoconch suggests that this species belongs to *Chamalycaeus*.

#### Genus Dicharax

Taxon classificationAnimaliaGastropodaCyclophoridae

Kobelt & Möllendorff, 1900

92ADF073-0314-52A2-B1D9-3D1072C3B8D2

Charax Benson, 1859: 177.
Dicharax Kobelt & Möllendorff, 1900: 186 (new replacement name for Charax Benson, 1859, non Charax Scopoli, 1777 [Pisces]).
Chamalycaeus (Dicharax) – Thiele 1929: 108; Wenz, 1938: 478; Egorov 2013: 37.
Chamalycaeus (Sigmacharax) Kuroda, 1943: 8.
Chamalycaeus (Cipangocharax) Kuroda, 1943: 11.
Chamalycaeus (Awalycaeus) Kuroda, 1951: 73–74.
Chamalycaeus (Awalycaeus) – Egorov 2013: 35–36.
Chamalycaeus (Cipangocharax) – Egorov 2013: 36.
Chamalycaeus (Sigmacharax) – Egorov 2013: 37–38.
Dicharax – Páll-Gergely et al. 2017: 10; Páll-Gergely and Asami 2017: 14 (Awalycaeus, Cipangocharax and Sigmacharax are synonyms).

##### Type species.

**Figure 13.**
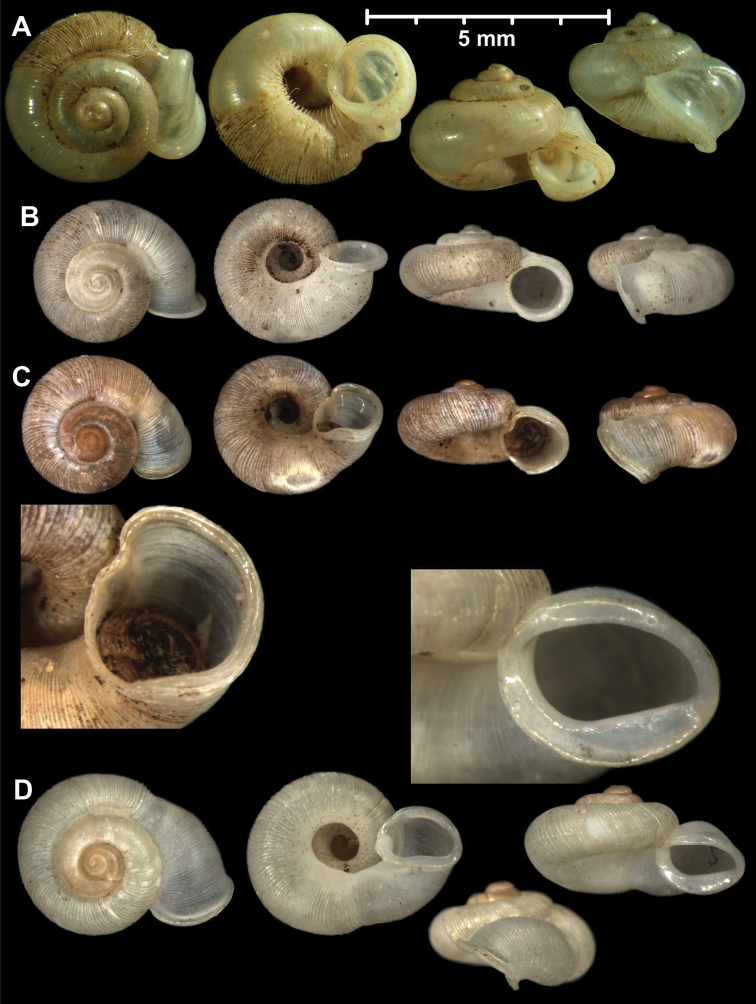
Type species of alycaeid genus-group taxa **A***Dicharax hebes* (Benson, 1857) (SMF 109244; type species of *Dicharax*) **B**D. (?) abei (Kuroda, 1951) (NSMT 50125; type species of *Awalycaeus*) **C**D. (?) biexcisus (Pilsbry, 1902) (NSMT 263; type species of *Cipangocharax*) **D**D. (?) itonis (Kuroda, 1943) (NSMT 78866; type species of *Sigmacharax*). Close-up images of the aperture are not to scale. All photographs: Barna Páll-Gergely.

*Alycaeus hebes* Benson, 1857 (Fig. 13A), SD Gude (1921: 236); *Awalycaeus abei* Kuroda, 1951 (Fig. 13B), by monotypy (*Awalycaeus*); *Alycaeus biexcisus* Pilsbry, 1902 (Fig. 13C), by monotypy (*Cipangocharax*); Chamalycaeus (Sigmacharax) itonis Kuroda, 1943 (Fig. 13D), by monotypy (*Sigmacharax*).

##### Diagnosis.

Shell very small to very large (D: 1–11 mm), in most cases the spire low (dorsal side flattened), spire rarely elevated (shell globular); protoconch low in nearly all species, smooth or finely pitted, not spirally striated; R1 usually glossy, sometimes ribbed (ribs can vary from weak to strong), but spiral lines almost always absent; R2 of variable length, typically with prominent ribs which are bent in an anterior direction, but many species have smooth R2 or straight ribs; R3 well developed, often with blunt or sharp swelling, in some taxa reduced (mostly ‘*Awalycaeus*’). Operculum thin or with various outer funnel-like structure resulting from modifications of the multispiral lamina. Central tooth typical for the family: 5–7 cusps, broad, central cusp pointed.

##### Differential diagnosis.

This genus can be recognised by the absence of spiral striation on the entire shell (protoconch and teleoconch). Very few species with spiral striation are classified in this genus.

##### Distribution.

**Figure 14.**
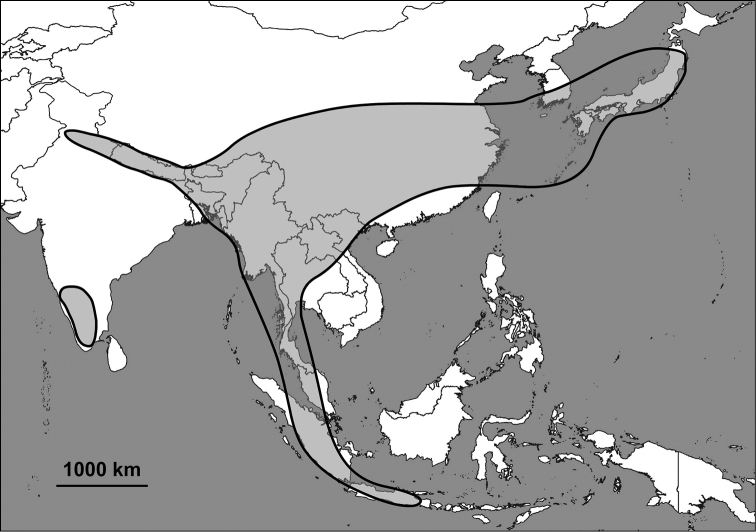
Distribution of *Dicharax* Kobelt & Möllendorff, 1900.

*Dicharax* inhabits a large geographic area from the southeastern Himalayan region to Japan, and through the Malay Peninsula to the southern arc of the Malay Archipelago up to Sumatra and Java. There are also isolated occurrences in the Western Ghats of India and in the southwestern Himalaya (see Fig. 14).

##### Remarks.

**Figure 15.**
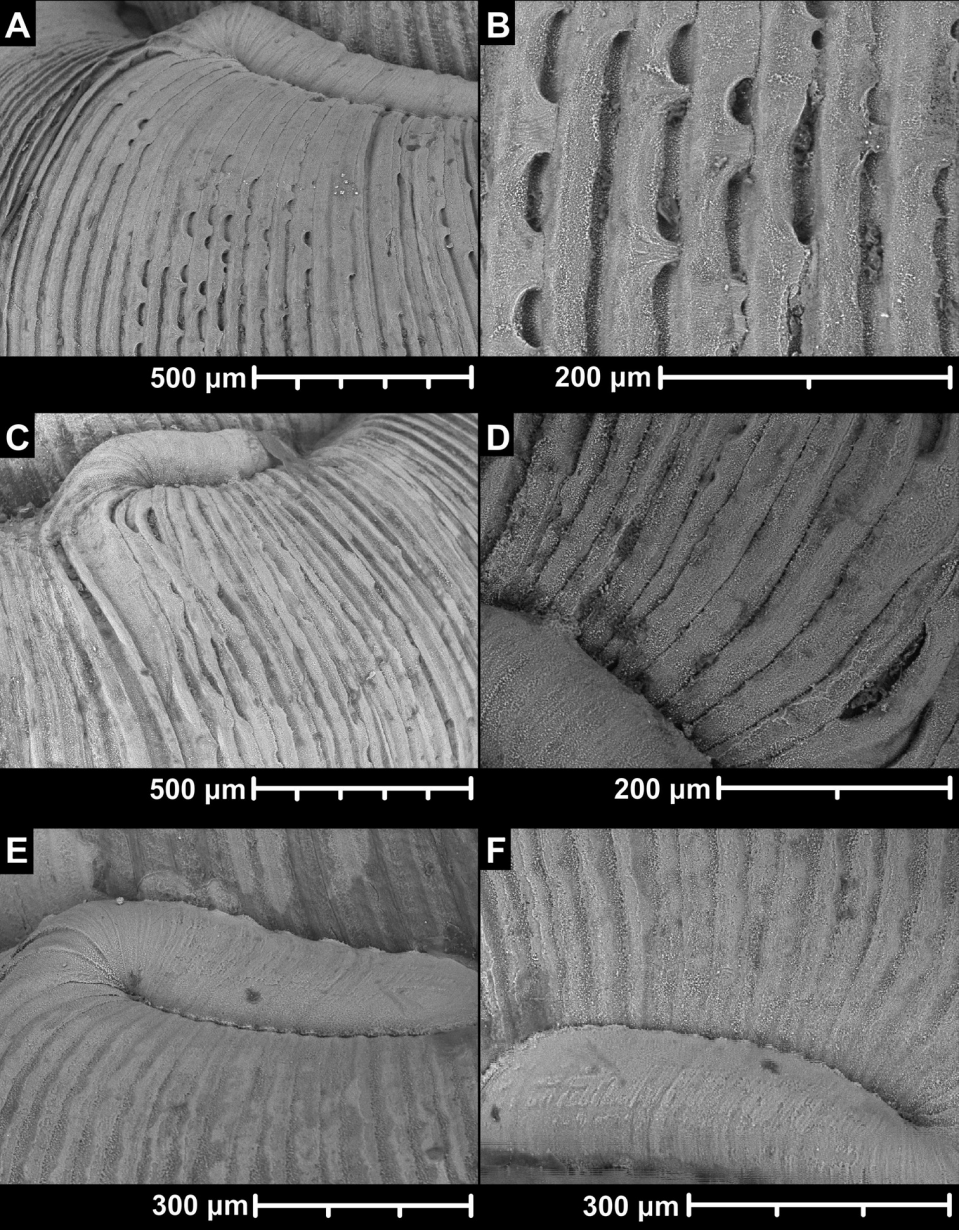
R2 ribs of Japanese *Dicharax* Kobelt & Möllendorff, 1900 species **A, B**Dicharax (?) abei (Kuroda, 1951), NSMT 50125 **C, D***Dicharax* (?) *biexcisus* (Pilsbry, 1902), NSMT 263 **E, F**Dicharax (?) itonis (Kuroda, 1943), NSMT 78866. All images: Barna Páll-Gergely.

*Cipangocharax*, introduced as the subgenus of *Chamalycaeus*, was described for a single species, *Alycaeus biexcisus*. The diagnosis of *Cipangocharax* was in fact the abbreviated description of *Alycaeus biexcisus*. Kuroda (1943) indicated some features in italics, emphasising the importance of these characters to distinguish *Cipangocharax* from other members of *Chamalycaeus*. These characters were the extraordinary thickness of the operculum, and the closely coiled outer belt on the outer surface of the operculum. The Japanese *Chamalycaeus* species described since Kuroda’s (1943) paper showed that there are transitional character states between the thick and belted operculum of *A. biexcisus* and the thin and unbelted opercula of most Japanese *Chamalycaeus* species (e.g., Minato 1993). For example, the operculum of *Cipangocharax kiuchii* is relatively slim, whereas that of “*Chamalycaeus*” *miyazakii* is exceptionally thickened. Consequently, the thickness of the operculum is not a distinguishing feature between *Cipangocharax* and other Japanese species assigned to *Chamalycaeus*. The outer opercular belt is missing in *C. placenovitas* (a species being otherwise very similar to *A. biexcisus*), therefore this character is also not stable within the genus. Moreover, the outer belt is known to be present and absent within the same species, or even population (see under *Chamalycaeus nipponensis* and *Dicharax simplicilabris*, see Páll-Gergely et al. 2017). The other distinctive character mentioned by Kuroda (1943) is the sinuated columellar margin. This region is not sinuated either in *C. placenovitas*, or in *C. okamurai*. Therefore, this character is also not stable within the genus. Moreover, Japanese *Chamalycaeus* species with unstriated protoconchs show an extraordinary diversity in terms of the formation of the aperture (*C. expanstoma*, *C. okamurai*, *C. yanoshigehumii*), indicating that the morphological variation is very high between species. Consequently, among the Japanese species with unstriated protoconch, it would not be legitimate to classify certain species into separate (sub)genera from the others. Furthermore, the species classified into the genus *Sigmacharax* also do not differ considerably from the rest of Japanese species with a smooth protoconch. Therefore, based on the absence of the spiral striation on the entire shell, these species are classified in the genus *Dicharax*. The overlapping ribs near the tube (Fig. 15) may a synapomorphic character of Japanese and Korean *Dicharax*, but this character was also found in the Chinese species *D. alticola* (Páll-Gergely et al. 2017), which is, due to the geographic distance, probably only distantly related. The morphological variation within the genus *Dicharax* (especially in northeastern India and in the Malay Archipelago) is so large, that at the current time we do not find it meaningful to separate the Japanese and Korean species into a separate subgenus within *Dicharax*.

*Awalycaeus* is a peculiar group of alycaeids due to the reduced (short, un-swollen) R3. However, in *Awalycaeus yanoshokoae* there is a moderately developed R3, which can be interpreted as an intermediate form between *Awalycaeus* and the rest of Japanese alycaeids which have a smooth protoconch. Given that the other shell characters (absence of spiral striation, merged R2 ribs) are similar to the other Japanese species, we also treat *Awalycaeus* as a synonym of *Dicharax*.

Such ‘over spitting’ of generic taxa inhabiting Japan has also been documented in the pulmonate family Clausiliidae, which is a character-rich family such as the Alycaeidae (Páll-Gergely et al. 2019). Nordsieck (1998) stated that the Japanese clausiliid genera and subgenera correspond only to subgenera and species groups of Western Palaearctic clausiliids. This claim was confirmed by recent molecular phylogeny (Motochin et al. 2017).

For the sake of simplicity, this genus is divided into three sections: typical (with curved R2 ribs), atypical (without the typical R2 sculpture), and those species from Japanese and Korean localities (including species formerly classified into *Awalycaeus*, *Cipangocharax*, and *Sigmacharax*).

#### Typical *Dicharax*

##### Dicharax anonymus

Taxon classificationAnimaliaGastropodaCyclophoridae

(Godwin-Austen, 1914)

7AE853D2-F110-57AB-96BE-C76153729DD8

Alycaeus anonymus Godwin-Austen, 1914: 405–406, pl. 139, figs 1, 1a.
Alycaeus anonymus – Gude 1921: 205.

###### Type locality.

“Akouk-toung, Pegu: Type; also Thoudaung and Yenandoung, Pegu”.

###### Material examined.

Akouktoung, Pegu, NHMUK 1906.4.4.67a (2 syntypes).

###### Remarks.

Protoconch slightly elevated, no spiral lines visible; R1 with rather regular, moderately elevated ribs without spiral lines inbetween; R2 with ribs being lamella-like.

##### Dicharax anthostoma

Taxon classificationAnimaliaGastropodaCyclophoridae

(Möllendorff, 1885)

8AB9E131-5CD8-544E-A4A7-4A807B043B31

Alycaeus anthostoma Möllendorff, 1885: 162.
Alycaeus pentagonus Heude, 1886: 211.
Alycaeus (Charax) anthostoma – Möllendorff 1886: 166, pl. 5, fig. 4.
Alycaeus anthostoma – Gredler 1891: 79. (considered A. pentagonus as synonym)
Alycaeus (Charax) anthostoma – Kobelt and Möllendorff 1897: 149.
Alycaeus (Dicharax) anthostoma – Kobelt 1902: 364.
Chamalycaeus (Dicharax) anthostoma – Zilch 1957: 145, pl. 6, fig. 22.
Dicharax anthostoma – Páll-Gergely et al. 2017: 30–32, figs 19A, B.

###### Type locality.

“in regione Badung provinciae sinensis Hubei”.

###### Material examined.

Patung, Hubei: China, coll. Boettger ex coll. Möllendorff, SMF 39225 (lectotype of *A. anthostoma*, designated by Yen 1939); same data, SMF 39226 (12 paralectotypes of *anthostoma*); China, Hupé, Coll. Möllendorff, ex Oberwimmer, ex David D. Thaanum Jan. 1947, MCZ 180902 (3 paralectotypes of *anthostoma*).

###### Remarks.

In our earlier paper (Páll-Gergely et al. 2017) we overlooked that Gredler (1891) already synonymised *A. pentagonus* with *A. anthostoma*.

Protoconch low, glossy, without spiral lines; R1 rather regularly ribbed, ribs low, no spiral striation visible; R2 relatively long, with ribs curved towards the aperture, forming a smooth surface.

##### Dicharax asaluensis

Taxon classificationAnimaliaGastropodaCyclophoridae

(Godwin-Austen, 1914)

0D3FF3CB-0898-5BD5-BE85-15B3247CDF55

Alycaeus crispatus var. – Godwin-Austen 1874: 93, pl. 4, fig. 2.
Alycaeus asaluensis Godwin-Austen, 1914: 385–386, pl. 145, figs 2, 2a, 2b.
Alycaeus (Dicharax) asaluensis – Gude 1921: 237.
Chamalycaeus (Dicharax) asaluensis – Ramakrishna et al. 2010: 56.

###### Type locality.

“Neuglo,” “Phulong” and “Dihung River, N. Cachar, north of Asalu”.

###### Material examined.

Dihung, N. Cachar, coll. Godwin-Austen, NHMUK 1903.7.1.2636. (2 syntypes); Asalu, North Cachar, NHMUK 1903.7.1.2761 (probably figured sample, with images on the sides of the box). See under *D. crispatus*.

###### Remarks.

Syntypes: protoconch low, spiral lines not visible; R1 irregularly ribbed, without spiral lines; R2 moderately long, with lamella-like, straight ribs.

###### Other sample.

The specimens are conspicuously variable in term of shell size (smallest: D = 2.7 mm, H = 2.1 mm; largest shell: D = 4.0 mm, H = 2.9 mm), the sculpture of R1 (nearly smooth to strongly, regularly ribbed) and the sculpture of R2. Despite the large variability, we consider all shells to belong to the same species since the variation is continuous between the extreme morphological forms. Protoconch low without spiral striation. R2 of some specimens typical *Dicharax*-like (ribs are curved towards the aperture), whereas those of other specimens are more lamella-like and less curved. Note that the shells with straight, lamella-like ribs on R2 are not weathered, which demonstrates that the two types of ribbing are a part of the intraspecific variation.

##### Dicharax avae

Taxon classificationAnimaliaGastropodaCyclophoridae

(W. T. Blanford, 1863)

E849669B-A1B7-57A5-A2D0-5BE06CA92FE8

Alycaeus avae W. T. Blanford, 1863: 323–324.
Alycaeus avae – Reeve 1878: pl. 3, species 20; Godwin-Austen 1914: 406–407, pl. 151, fig. 6.
Alycaeus (Dicharax) avae – Kobelt 1902: 365; Gude 1921: 238.

###### Type locality.

“The hills east of Mandalay and Ava”.

###### Material examined.

Shan Hills, E of Ava, Burma, coll. Blanford, NHMUK 1906.4.4.61 (6 syntypes).

###### Remarks.

Protoconch low without spiral lines; R1 irregularly, densely ribbed, no spiral lines visible; R2 relatively short; ribs lamella-like, curved towards the aperture, but they are not in contact.

##### Dicharax bison

Taxon classificationAnimaliaGastropodaCyclophoridae

Páll-Gergely & Hunyadi, 2017

FACAD3D0-4574-5406-920B-F715297DAE01

Dicharax bison Páll-Gergely & Hunyadi in Páll-Gergely et al., 2017: 32–34, figs 21A, 22, 23.

###### Type locality.

“China, Sichuan, Dujiangyan Shi, Taianzhen, Qingchenghoushan, Sanlongshuijing Rongdong, 942 m, 30°55.15418'N, 103°29.72375'E”.

###### Material examined.

Holotype (HNHM 99703) and several paratypes, see the original description for further details.

###### Remarks.

Protoconch low, rather matte; R1 regularly, densely ribbed, ribs low, without spiral lines; ribs becoming slightly more widely spaced towards end of R1; R2 very densely ribbed, ribs curved towards the aperture; for more details see the original description.

##### Dicharax caudapiscis

Taxon classificationAnimaliaGastropodaCyclophoridae

Páll-Gergely & Hunyadi, 2018

43648BE4-B6F1-56E9-88A1-FDE52E1422C9

Dicharax caudapiscis Páll-Gergely & Hunyadi, 2018: 60, fig. 1A–E.

###### Type locality.

“Thailand, Chiang Rai Province, approx. 9 km south-southwest from Mae Sai, Wat Tham Pla, 400 m a.s.l., 20°19.723'N, 99°51.817'E”.

###### Material examined.

HNHM 100177 (holotype).

###### Remarks.

Protoconch glossy; R1 also glossy, with very fine, irregular growth lines; R2 with dense, curved ribs (ca. 46 altogether), for more details see the original description.

##### Dicharax chennelli

Taxon classificationAnimaliaGastropodaCyclophoridae

(Godwin-Austen, 1886)

32CA960F-0FFB-556E-861E-BA5AA58CF0C7

Alycaeus chennelli Godwin-Austen, 1886: 192–193, pl. 48, fig. 2.
Alycaeus chennelli and chennelli var. – Godwin-Austen 1914: 387.
Alycaeus (Dicharax) chennelli – Kobelt 1902: 366; Gude 1921: 240–241.
Chamalycaeus (Dicharax) chennelli – Ramakrishna et al. 2010: 57.

###### Type locality.

“Piknúi, Naga Hills”; “Lhota Naga Hills” (*chennelli* var.).

###### Material examined.

Piknui, Naga Hills, leg. A. Chennell, NHMUK 1903.7.1.2612 (8 syntypes); Lhota Naga Hills, leg. Chennell, NHMUK 1903.7.1.2613 (10 shells = “*chennelli* var.”).

###### Remarks.

Protoconch rather glossy, low, no spiral lines visible; R1 glossy, with widely spaced sharp ribs which are present only near the suture, and without spiral lines; R2 with curved ribs, typical to *Dicharax*.

The only stable character which distinguishes *D. chennelli* from *D. diagonius* is the presence of a lower apertural bay in the former, whilst it is absent in the latter.

##### Dicharax conicus

Taxon classificationAnimaliaGastropodaCyclophoridae

(Godwin-Austen, 1871)

B471B14C-295D-590A-9905-BD8287837E42

Alycaeus conicus Godwin-Austen, 1871: 87–88, pl. 3, fig. 1.
Alycaeus conicus – Reeve 1878: pl. 1, species 9; Godwin-Austen 1914: 387–388, pl. 143, figs 4, 4a, 4b; Gude 1921: 208.
Alycaeus (Alycaeus) conicus – Kobelt 1902: 342; Ramakrishna et al. 2010: 47.

###### Type locality.

“Was abundant on the Limestone Hill east of Kopili river, North Cachar District, and was occasionally also found in other places, but rare”.

###### Material examined.

Samiamri, E of the Kopili R., leg. Godwin-Austen, NHMUK 1903.7.1.2674 (12 syntypes).

###### Remarks.

Protoconch low, no spiral lines visible; R1 is similar to protoconch by being moderately glossy and sculptureless; R2 short, with regular ribs curved towards the aperture, forming a relatively wide, flat area, when viewed from above.

##### Dicharax conicus jatingaensis

Taxon classificationAnimaliaGastropodaCyclophoridae

Páll-Gergely nom. nov.

FCC8DA7B-2FB7-522B-9C63-CFD641B1C2C7

Alycaeus conicus var. nanus Godwin-Austen, 1914: 388, pl. 138, figs 6, 6a, 6b. (non Alycaeus nanus Möllendorff, 1886)
Alycaeus conicus var. nana – Gude 1921: 208.

###### Type locality.

“Jatinga Valley, North Cachar Hills”.

###### Material examined.

Jatinga valley, N. Cachar, NHMUK 1903.7.1.2675 (12 syntypes); Khasi Hills, NHMUK 1903.7.1.2565 (1 shell, included with type lot, but not mentioned in the original description and not considered as part of the type series).

###### Etymology.

The replacement name (*jatingaensis*) refers to the type locality (Jatinga Valley).

###### Remarks.

Protoconch low, glossy, no spiral lines visible; R1 also glossy without spiral lines; R2 relatively short, with regular ribs; each rib has a lamella-like horizontal projection towards their anterior neighbours.

Alycaeus conicus var. nanus Godwin-Austen, 1914 is a primary homonym of *Alycaeus nanus* Möllendorff, 1886 (treated as a synonym of *A. diminutus*). Both taxa have been used as valid with this combination after 1899, thus, a replacement name is given to the junior homonym.

##### Dicharax crenatus

Taxon classificationAnimaliaGastropodaCyclophoridae

(Godwin-Austen, 1871)

50659405-971A-5059-BF1A-099F3CB67458

Alycaeus crenatus Godwin-Austen, 1871: 90–91, pl. 3, fig. 5.
Alycaeus crenatus – Reeve 1878: pl. 1, species 1, figs a, b; Godwin-Austen 1914: 388–389, pl. 143, figs 8, 8a, 8b.
Alycaeus (Dicharax) crenatus – Kobelt 1902: 366; Gude 1921: 241.
Chamalycaeus (Dicharax) crenatus – Ramakrishna et al. 2010: 57.

###### Type locality.

“On Burrail Range, N. Cachar, at ca. 5000 feet”.

###### Material examined.

Mokarsa, Khasi Hills, coll. Godwin-Austen, NHMUK 1903.7.1.2642 (2 syntypes). Note that the type locality does not match with the locality of the type sample, but the type locality was clarified in Godwin-Austen 1914 in 1897–1914: 389, and the drawing in the original description is identical with the two syntypes.

###### Remarks.

Protoconch rather glossy, low, without spiral lines; R1 rather regularly, finely ribbed without spiral striation; R2 relatively short, with regular ribs curved towards the aperture.

##### Dicharax cristatus

Taxon classificationAnimaliaGastropodaCyclophoridae

(Möllendorff, 1886)

DF8EDCE3-CB6C-50AE-AECA-C5FE3A42FB01

Alycaeus cristatus Möllendorff, 1886: 168, pl. 5, fig. 6.
Alycaeus (Charax) cristatus – Kobelt and Möllendorff 1897: 150.
Alycaeus (Dicharax) cristatus – Kobelt 1902: 367.
Alycaeus (Chamalycaeus) smithi Fulton, 1907: 157, pl. 10, fig. 5.
Alycaeus (Charax) fimbriatus var. simplicilabris Bavay & Dautzenberg, 1912: 53–54, pl. 6, fig. 18.
Chamalycaeus (Dicharax) cristatus – Zilch 1957: 146, pl. 6, fig. 23.
Dicharax cristatus – Páll-Gergely et al. 2017: 34–43, figs 12C, D, 13C, 24–27, 28A–D, 29C, D, 30 (smithi Fulton, 1907 and simplicilabris Bavay & Dautzenberg, 1912 are synonyms).

###### Type locality.

“in provinciae sinensis Hunan parte meridionali”

###### Material examined.

Süd-Hunan: China, coll. Möllendorff 1886, SMF 39231 (lectotype, designated by Yen 1939); Same data, SMF 39232 (11 paralectotypes); Same data, SMF 39233 (2 paralectotypes); for types of the synonymised names see Páll-Gergely et al. (2017).

###### Remarks.

Protoconch low, glossy, without spiral lines; R1 with regular, low ribs and without spiral striation; R2 relatively long, with ribs curved towards the aperture and reaching each other.

##### Dicharax cucullatus

Taxon classificationAnimaliaGastropodaCyclophoridae

(Theobald, 1870)

6D3E8243-24A5-5F13-B1B4-21DE74A7CA51

Alycaeus cucullatus Theobald, 1870: 396–397, pl. 18, fig. 2.
Alycaeus cucullatus – Reeve 1878: pl. 2, species 12; Godwin-Austen 1914, Vol. II: 407, pl. 155, fig. 5.
Alycaeus (Dicharax) cucullatus – Kobelt 1902: 367–368; Gude 1921: 244–245.

###### Type locality.

“Shan States”.

###### Material examined.

Shan States, NHMUK 1888.12.4.951–952 (2 syntypes).

###### Remarks.

Protoconch low, rather matte, no spiral lines visible; R1 regularly, strongly ribbed without spiral striation; R2 Relatively long, with all ribs curved towards the aperture, and they are almost in contact.

##### Dicharax damsangensis

Taxon classificationAnimaliaGastropodaCyclophoridae

(Godwin-Austen, 1886)

CA7BB933-1DF9-56A6-843A-294770043FEE

Alycaeus damsangensis Godwin-Austen, 1886: 192, pl. 43, figs 3, 3a–c.
Alycaeus (Dicharax) damsangensis – Kobelt 1902: 368; Gude 1921: 246–247.
Alycaeus (Charax) damsangensis – Godwin-Austen 1914: 339.

###### Type locality.

“Damsang Peak, Western Bhutan Hills”.

###### Material examined.

Damsang, W. Bhutan, leg. Robert, NHMUK 1903.7.1.2677 (12 syntypes).

###### Remarks.

Protoconch moderately glossy, low, no spiral lines visible; R1 with regular ribs and without spiral striae; R2 is moderately long, the upper part of the ribs are horizontal (in cross-sectional view the ribs are T-shaped); in most cases the ribs do not reach each other.

##### Dicharax davisi

Taxon classificationAnimaliaGastropodaCyclophoridae

(Godwin-Austen, 1914)

CF0AFD57-A2FC-53C3-911A-05AE63309F91

Alycaeus davisi Godwin-Austen, 1914: 408, pl. 148, figs 9, 9a.
Alycaeus (Chamalycaeus) davisi – Gude 1921: 226.

###### Type locality.

“Siam and Shan boundary”.

###### Material examined.

Shan States, leg. Woodthorpe, NHMUK 1903.7.1.1630 (4 syntypes).

###### Remarks.

Protoconch low, glossy, very finely granulated, no spiral lines visible; R1 finely, regularly ribbed without spiral lines; R2 long, with strong signs of Byne’s disease; the ribs are curved towards the aperture and reach each other (typical *Dicharax* structure), forming a glossy, nearly smooth surface.

##### Dicharax depressus

Taxon classificationAnimaliaGastropodaCyclophoridae

(Bavay & Dautzenberg, 1912)

91474D22-6199-5657-8587-595EE68F0169

Alycaeus depressus Bavay & Dautzenberg, 1912: 51–52, pl. 4, figs 10–13.
Dicharax depressus – Páll-Gergely et al. 2017: 43–45, figs 12E, F, 13D, 28E–H, 29E, F, 31A–C; Inkhavilay et al. 2019: 14, fig. 5A.

###### Type locality.

“Pac-Kha, Tonkin”

###### Material examined.

Pac-Kha, leg. Messager, MNHN-IM-2000-27165 (1 syntype); for additional specimens see Páll-Gergely et al. (2017).

###### Remarks.

Protoconch low; R1 glossy with regular, dense ribs, which gradually transform to an irregularly ribbed section having widely spaced ribs at end of R1; ribs low and blunt on whole shell; R2 very densely ribbed, ribs curved towards aperture.

Shells of a single sample had some spiral striation, which is highly unusual in this genus (Páll-Gergely et al. 2017)

##### Dicharax diagonius

Taxon classificationAnimaliaGastropodaCyclophoridae

(Godwin-Austen, 1871)

0C950916-5FA8-5ADD-9946-B6DDDE4066DF

Alycaeus diagonius Godwin-Austen, 1871: 88–89, pl. 3, fig. 2.
Alycaeus diagonus [sic] – Reeve 1878: pl. 1, species 2.
Alycaeus (Dicharax) diagonius – Kobelt 1902: 368–369; Gude 1921: 247–248.
Alycaeus diagonius – Godwin-Austen 1914: 389–390, pl. 143, figs 5, 5a, 5b.
Chamalycaeus (Dicharax) diagonius – Ramakrishna et al. 2010: 58.

###### Type locality.

“The Diyung valley, north of Asálú, in Cachar District”.

###### Material examined.

Diyung valley, N. of Asalu, N. Cachar, coll. Godwin-Austen, NHMUK 1903.7.1.2678 (10 syntypes).

###### Remarks.

Protoconch low, no spiral lines visible; R1 also without spiral lines, its sculpture is similar to that of the protoconch; R2 short, with regular ribs curved towards the aperture.

##### Dicharax digitatus

Taxon classificationAnimaliaGastropodaCyclophoridae

(H. F. Blanford, 1871)

54D2665E-8C43-5B06-B93C-B2C8AB393EFC

Alycaeus digitatus H. F. Blanford, 1871: 41–42, pl. 2, fig. 4.
Alycaeus (Dicharax) digitatus – Kobelt 1902: 369; Gude 1921: 248.
Alycaeus digitatus – Godwin-Austen 1914: 339–340, pl. 134, figs 5, 5a.
Chamalycaeus (Dicharax) digitatus – Ramakrishna et al. 2010: 59.

###### Type locality.

“apud Darjeeling in vallo Rungno fluminis Himalayæ Sikkimensis”.

###### Material examined.

Rechila Pk., Sikkim, leg. W. Robert, NHMUK 1903.7.1.1253 (1 shell, probably not syntype, but figured by Godwin-Austen 1914).

###### Remarks.

Protoconch low, without spiral lines; R1 with very fine ribs, no spiral lines visible; R2 moderately long, ribs curved towards the aperture.

##### Dicharax diminutus

Taxon classificationAnimaliaGastropodaCyclophoridae

(Heude, 1885)

4F07CCC8-E90E-5E84-AF00-66847D2B7059

Alycoeus [sic] diminutus Heude, 1885: 96, pl. 24, figs 5, 5a.
Alycaeus diminutus – Heude 1886: 210.
Alycaeus (Orthalycaeus) diminutus – Möllendorff 1886: 170.
Alycaeus (Orthalycaeus) nanus Möllendorff 1886: 170, pl. 5, fig. 8.
Alycaeus (Chamalycaeus) diminutus – Kobelt and Möllendorff 1897: 148; Kobelt 1902: 354.
Alycaeus nanus – Tarruella and Domènech 2011: 72.
Dicharax diminutus – Páll-Gergely et al. 2017: 50–52, figs 33A–C (nanus Möllendorff, 1886 is a synonym).

###### Type locality.

“in ditione Tchen-k’eou”.

###### Material examined.

Hunan, China, coll. Möllendorff ex coll. Heude, SMF 39255 (1 syntype of *A. diminutus* [“*minutus*” on the label]); for additional specimens see Páll-Gergely et al. (2017).

###### Remarks.

Protoconch low, glossy, without spiral lines, R1 with regular, dense, low ribs, no spiral striation visible; R2 short, with dense ribs curved towards the aperture.

##### Dicharax diplochilus

Taxon classificationAnimaliaGastropodaCyclophoridae

(Möllendorff, 1887)

8A038F0B-9AAB-5CED-A1D8-6B76666BA887

Alycaeus diplochilus Möllendorff, 1887a: 310.
Alycaeus diplochilus – Möllendorff 1891: 342, pl. 30, figs 8, 8a, 8b.
Alycaeus (Chamalycaeus) diplochilus – Kobelt 1902: 354–355.
Chamalycaeus (Chamalycaeus) diplochilus – Zilch 1957: 142, pl. 5, fig. 5.
Dicharax diplochilus – Páll-Gergely et al. 2017: 45, fig. 31D.

###### Type locality.

“Ad Buket Pondong”.

###### Material examined.

Malakka: Bukit Pondong (Perak), coll. Möllendorff, SMF 109476 (lectotype, designated by Zilch 1957); Same data, SMF 109477 (5 paralectotypes); Perak, leg. Hungerford, NHMUK 1891.3.17.779–782 (4 possible syntypes, these are labelled as types, but this is questionable).

###### Remarks.

Protoconch low, rather matte, without spiral lines; R1 with similar sculpture to that of the protoconch; R2 very short, with ca. 20 regular ribs, ribs curved towards the aperture.

##### Dicharax draco

Taxon classificationAnimaliaGastropodaCyclophoridae

Páll-Gergely & Hunyadi, 2017

EBEDD32F-8C21-5C33-B9A3-1BF9B1CD2E35

Dicharax draco Páll-Gergely & Hunyadi in Páll-Gergely et al., 2017: 52–54, fig. 34A.

###### Type locality.

“China, Yunnan, Wenshanzhuang Zumiaozu Zizhizho, Guangnan Xian, Liji, 1611 m, 23°45.54175'N, 104°59.55567'E”.

###### Material examined.

Holotype (HNHM 99705) and a few paratypes (see the original description).

###### Remarks.

Protoconch low, rather glossy; R1 with low, regular ribs, ribbing weaker at beginning of R1, but stronger at end of R1; R2 with ribs curved towards aperture.

##### Dicharax elevatus

Taxon classificationAnimaliaGastropodaCyclophoridae

(Heude, 1886)

DFC7B62D-7CFE-5A40-8CA7-486682E2034B

Fig. 16

Alycaeus elevatus Heude, 1886: 210.
Alycaeus elevatus – Heude 1890: 129, pl. 36, fig. 19.
Alycaeus (Chamalycaeus) elevatus – Kobelt and Möllendorff 1897: 148; Kobelt 1902: 355.
Metalycaeus (?) elevatus – Páll-Gergely et al. 2017: 104–105, fig. 69I.

###### Type locality.

“Tchen K’eou”.

###### Material examined.

Cheng-Kou County, Chong-qing, China, HMT-218a, deposited in IZCAS (syntype: labelled as lectotype, but probably there was no valid lectotype designation). No type specimens deposited in American museums were reported by Johnson (1973).

###### Remarks.

This species could be examined for the first time, since it was not examined in our previous paper (Páll-Gergely et al. 2017). Protoconch low, smooth; R1 without spiral striation, its beginning is densely, finely ribbed, which gradually changes to a more widely-spaced, strongly ribbed surface. R2 and R3 are of comparable length, R2 ribs dense, low, blunt, not elevated. The most similar species is *D. fargesianus*, which has denser ribs on R1 and R3, and more marked swelling on R3.

The examined specimen has a shorter R3 than the one illustrated by Heude (1890) (see also Páll-Gergely et al. 2017: fig. 69I). This raises some doubts about the identity of this type.

**Figure 16.**
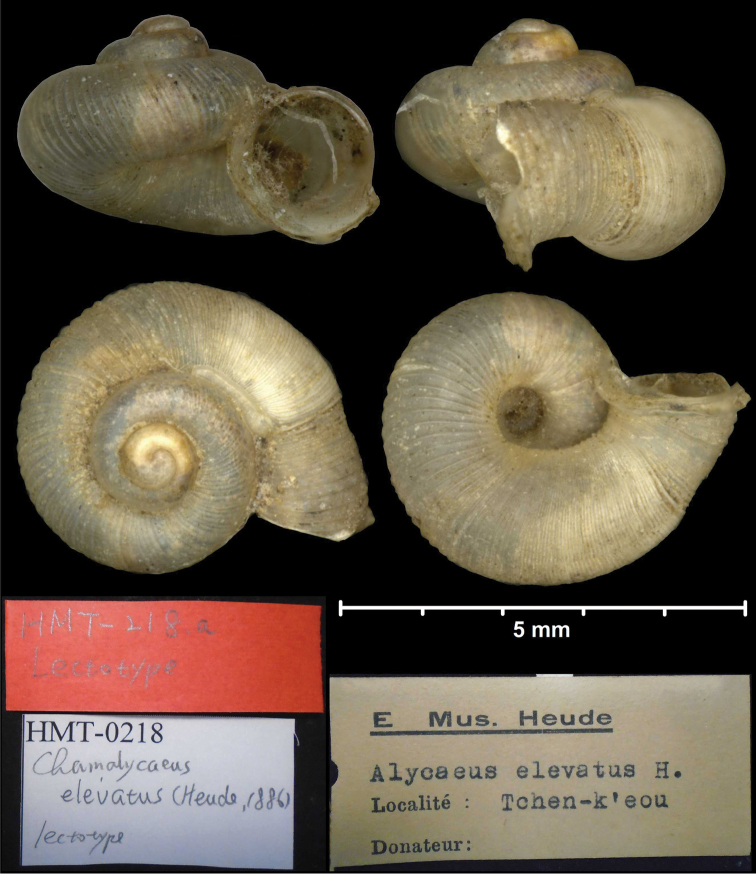
Dicharax (?) elevatus (Heude, 1886), syntype (HMT-218a). Photographs: Kaibaryer Meng.

##### Dicharax fimbriatus

Taxon classificationAnimaliaGastropodaCyclophoridae

(Bavay & Dautzenberg, 1912)

7ABF13F3-94F8-57A7-819D-C4804156A485

Alycaeus (Charax) fimbriatus Bavay & Dautzenberg, 1912: 52–53, pl. 6, figs 14–17.
Chamalycaeus plicilabris multidentatus Yen, 1939: 29, pl. 2, fig. 33.
Dicharax fimbriatus – Páll-Gergely et al. 2017: 54–61, figs 13E, 35–37, 38A–D, 39 (multidentatus Yen, 1939 is a synonym); Inkhavilay et al. 2019: 14, fig. 5B.

###### Type locality.

“Pac-Kha”.

###### Material examined.

Pac-Kha, leg. Messager, MNHN-IM-2000-27166 (1 syntype); for additional specimens see Páll-Gergely et al. (2017).

###### Remarks.

Protoconch low, rather matte; R1 rather regularly ribbed with blunt, but strong ribs; rib density decreases towards the end of region; R2 extremely densely ribbed; ribs curved towards aperture, forming a nearly smooth surface.

##### Dicharax fraterculus

Taxon classificationAnimaliaGastropodaCyclophoridae

(Bavay & Dautzenberg, 1900)

BE9C5A8F-FD98-52E2-9FB3-3F65C1DF0D12

Alycaeus (Charax) fraterculus Bavay & Dautzenberg, 1900a: 120.
Alycaeus (Charax) fraterculus – Bavay and Dautzenberg 1900b: 457–458, pl. 11, figs 11–14.
Alycaeus (Dicharax) fraterculus – Kobelt 1902: 370.
Dicharax fraterculus – Páll-Gergely et al. 2017: 62–64, fig. 41.

###### Type locality.

“Haut-Tonkin”.

###### Material examined.

Haut Tonkin, leg. Messager, MNHN-IM-2000-27168 (1 syntype), for additional specimens see Páll-Gergely et al. (2017).

###### Remarks.

Protoconch low, glossy; R1 coarsely, rather irregularly ribbed, ribs weaker on edge of body whorl; R2 very finely and densely ribbed, ribs low, curved towards the aperture.

##### Dicharax generosus

Taxon classificationAnimaliaGastropodaCyclophoridae

(Godwin-Austen, 1914)

E7838172-84A3-5D9C-A82A-28A42266A91F

Alycaeus generosus Godwin-Austen, 1914: 374, pl. 138, figs 8, 8a, 8b.
Alycaeus (Cycloryx) generosus – Gude 1921: 279.
Cycloryx generosus – Ramakrishna et al. 2010: 71.

###### Type locality.

“Khasi Hills”.

###### Material examined.

Khasi Hills, coll. Godwin-Austen, NHMUK 1903.7.1.2566 (2 syntypes).

###### Remarks.

Protoconch low, no spiral lines visible; R1 glossy, no ribs or spiral striation visible; R2 very short, with only ca. 14 ribs; ribs curved towards the aperture, which do not reach each other.

##### Dicharax globulus

Taxon classificationAnimaliaGastropodaCyclophoridae

(Godwin-Austen, 1874)

C602F2D2-7B30-593A-9928-7371DD65DD4D

Alycaeus globulus Godwin-Austen, 1874: 147–148, pl. 3, fig. 4.
Alycaeus (Dicharax) globulus – Kobelt 1902: 371; Gude 1921: 254.
Alycaeus globulus – Godwin-Austen 1914: 392, pl. 144, figs 4, 4a, 4b.
Chamalycaeus (Dicharax) globulus – Ramakrishna et al. 2010: 60.

###### Type locality.

“Phunggum, a Naga village at head of the Lanier valley, at 5,000 feet”.

###### Material examined.

Phunggum, Lahupa Naga Hills, Munipur, NHMUK 1903.7.1.2486 (13 syntypes).

###### Remarks.

Protoconch low, matte, no spiral lines visible; R1 with irregular, rough wrinkles, especially near the suture, but no spiral lines are visible; R2 relatively long, with regular ribs curved towards the aperture; the ribs are bent, nearly reach each other forming a nearly smooth surface.

##### Dicharax habiangensis

Taxon classificationAnimaliaGastropodaCyclophoridae

(Godwin-Austen, 1914)

275C7527-4A36-59CA-B32F-8330BDFBC8A7

Alycaeus habiangensis Godwin-Austen, 1914: 374, pl. 138, figs 2, 2a, 2b.
Alycaeus (Dicharax) habiangensis – Gude 1921: 254.
Chamalycaeus (Dicharax) habiangensis – Ramakrishna et al. 2010: 61.

###### Type locality.

“Habiang Garo, on the West Khasi border”.

###### Material examined.

Habiang Garo, W. Khasi, leg. Godwin-Austen, NHMUK 1903.7.1.2649 (1 syntype).

###### Remarks.

Protoconch low, glossy, no spiral lines visible; R1 glossy, without ribs and spiral lines; R2 very short, only ca. 13 ribs are present; the ribs are slightly curved towards the aperture at their tops, and do not reach each other (typical *Dicharax*).

##### Dicharax hebes

Taxon classificationAnimaliaGastropodaCyclophoridae

(Benson, 1857)

8E82FB8F-578B-55F0-9F76-B6A3209407BC

Alycaeus hebes Benson, 1857: 204–205.
Alycaeus hebes – Reeve 1878: pl. 6, species 52; Godwin-Austen 1886: 191, pl. 43, figs 1, 1a–c.
Alycaeus (Dicharax) hebes – Kobelt 1902: 371; Gude 1921: 255.
Alycaeus hebes – Godwin-Austen 1914: 374–375, pl. 145, figs 5, 5a, 5b.
Chamalycaeus (Dicharax) hebes – Ramakrishna et al. 2010: 61.
Dicharax hebes – Páll-Gergely et al. 2017: 10, fig. 5.

###### Type locality.

“ad Teria Ghát”.

###### Material examined.

Khasi Hills, Teria Ghat, leg. Godwin-Austen, NHMUK 1903.7.1.2658 (17 specimens); Vorder-Indien, Khasi Berge, coll. Möllendorff, SMF 109244; NHMUK 1888.12.4.908-910, leg. Theobald, possible syntypes. The shell, which was believed to be a possible type specimen (No locality, UMZC I.102635), belongs to another *Dicharax* species.

###### Remarks.

Protoconch low, glossy, no spiral lines visible; R1 with low, irregular growth ridges, but otherwise glossy without spiral lines; R2 moderately long, with regular ribs nearly reaching each other; the ribs near the beginning of R2 are bent in an anterior direction, the ribs near the end of R2 are bent in a posterior direction, and the ribs in the middle section of R2 are T-shaped in cross sectional view.

##### Dicharax humilis

Taxon classificationAnimaliaGastropodaCyclophoridae

(W. T. Blanford, 1862)

E823CE0E-73E8-5CAA-A07D-E44A7BB1F1EA

Alycaeus humilis W. T. Blanford, 1862: 136–137.
Alycaeus humilis – Reeve 1878: pl. 5, species 40; Godwin-Austen 1914: 408–409, pl. 151, fig. 8.
Alycaeus (Dicharax) humilis – Kobelt 1902: 372; Gude 1921: 255–256.

###### Type locality.

“ad Akouktoung, ad ripas fluminis Irawaddi, in provincia Burmana Pegu”.

###### Material examined.

River Bank, Myanoung, Pegu, NHMUK 1906.4.4.69 (1 shell); Pegu, coll. C. Bosch ex coll. H. Rolle, SMF 192340 (4 shells).

###### Remarks.

The only available specimen housed in the NHM was weathered; Protoconch low, with any recognisable sculpture; R1 with irregular, fine ribbing which turns into a widely spaced, strongly ribbed area at the end of the region, no spiral lines visible; R2 relatively short, weathered. SMF sample: protoconch low, rather glossy; R1 also glossy, with widely spaced, strongly ribs near the end of the region, no spiral lines visible; R2 relatively short, ribs curved towards the aperture.

##### Dicharax imitator

Taxon classificationAnimaliaGastropodaCyclophoridae

Páll-Gergely & Hunyadi, 2017

2C1CA3A4-190C-550B-A34C-5F89DBDCB46D

Dicharax imitator Páll-Gergely & Hunyadi in Páll-Gergely et al., 2017: 64–65, figs 34B, 38E, F, 42A, B.

###### Type locality.

“China, Guangxi, Bose Shi, Leye Xian, Moli Cun, cliffs S of the village on the left side of the Buliu River, 540 m, 24°39.436'N, 106°43.245'E”.

###### Material examined.

Holotype (HNHM 99706) and a few paratypes, see Páll-Gergely et al. (2017).

###### Remarks.

Protoconch without any recognisable sculpture, although it was weathered in examined shells; R1 smooth, glossy, with sharp, widely spaced, regular ribs near suture and inside umbilicus; R2 finely, densely ribbed, ribs are curved towards aperture at end of R2, but in curved in posterior direction at beginning of R2.

##### Dicharax jaintiacus

Taxon classificationAnimaliaGastropodaCyclophoridae

(Godwin-Austen, 1871)

761E4923-19E4-565A-B013-EDDBF16299ED

Alycaeus Jaintiacus Godwin-Austen, 1871: 92–93, pl. 5, fig. 3.
Alycaeus (Dicharax) jaintiacus – Kobelt 1902: 372; Gude 1921: 256.
Alycaeus jaintiacus – Godwin-Austen 1914: 375, pl. 143, figs 3, 3a, 3b.
Chamalycaeus (Dicharax) jaintiacus – Ramakrishna et al. 2010: 61.

###### Type locality.

“in Nongjinghi, Jiantia”.

###### Material examined.

Nongjinghi, Jiantia Hills, leg. Godwin-Austen, NHMUK 1903.7.1.2686 (14 syntypes).

###### Remarks.

Protoconch low, without spiral lines; R1 smooth except for some rough wrinkles near the suture, no spiral striation visible; R2 moderately long, with regular ribs, which are curved towards the aperture.

##### Dicharax jaintiacus crassus

Taxon classificationAnimaliaGastropodaCyclophoridae

(Godwin-Austen, 1914)

16A6B849-497B-525A-9F36-71228BFD54C4

Alycaeus jaintiacus var. crassus Godwin-Austen, 1914: 375, pl. 137, figs 5, 5a.
Alycaeus (Dicharax) jaintiacus Var. crassa – Gude 1921: 256–257.

###### Type locality.

“in Nongjinghi, Jiantia, 4563 feet”.

###### Material examined.

Nonjinghi, Jiantia, coll. Godwin-Austen, NHMUK 1903.7.1.2752 (4 syntypes in 2 vials).

###### Remarks.

Protoconch matte, R1 smooth, no spiral lines visible (although the entire shell is somewhat weathered); R2 of normal length, the ribs are overall low, they are slightly curved towards the aperture.

##### Dicharax kezamaensis

Taxon classificationAnimaliaGastropodaCyclophoridae

(Godwin-Austen, 1914)

083B51EE-E17A-57D5-998A-5D3F2FC62F1C

Alycaeus kezamaensis Godwin-Austen, 1914: 393, pl. 149, fig. 1.
Alycaeus (Dicharax) kezamaensis – Gude 1921: 258.
Chamalycaeus (Dicharax) kezamaensis – Ramakrishna et al. 2010: 62.

###### Type locality.

“Kezama, Aughami-Naga Hills”.

###### Material examined.

Kezama, Naga Hills, coll. Godwin-Austen, NHMUK 1903.7.1.2556 (1 syntype).

###### Remarks.

Protoconch low, glossy, no spiral lines visible; R1 with strong ribs and without spiral striation; R2 moderately long, ribs curved towards the aperture (typical *Dicharax* structure).

##### Dicharax lahupaensis

Taxon classificationAnimaliaGastropodaCyclophoridae

(Godwin-Austen, 1914)

AFD91033-054D-5E61-9CC5-D7BC7F30775A

Alycaeus lahupaensis Godwin-Austen, 1914: 394, pl. 141, figs 3, 3a.
Alycaeus (Raptomphalus) lahupaensis – Gude 1921: 287–288.
Chamalycaeus (Raptomphalus) lahupaensis – Ramakrishna et al. 2010: 68.

###### Type locality.

“Gaziphimi, Lahupa Naga Hills, Munipur”.

###### Material examined.

Gaziphimih, N.E. Munipur, NHMUK 1903.7.1.2655 (10 syntypes).

###### Remarks.

Protoconch low, glossy, no spiral lines visible; R1 glossy, with some spiral lines, which are, however, not present on the surface but are found on parts of the inner layers of the shell and visible through the semi-transparent upper layer (thus, not homologous with the spiral striation of other genera); R2 short, with lamella-like, sharp ribs, which are slightly curved towards the aperture; there is quite large gap between the ribs.

##### Dicharax longituba

Taxon classificationAnimaliaGastropodaCyclophoridae

(E. von Martens, 1864)

7B13D96B-6D2A-59A1-931B-4564FEFF7C30

Alycaeus longituba E. von Martens, 1864: 1 20.
Alycaeus longituba – E. von Martens 1867: 151, pl. 4, fig. 8.
Alycaeus (Dicharax) longituba – Kobelt 1902: 373.
Chamalycaeus longituba – van Benthem Jutting 1948: 573–575, fig. 29.

###### Type locality.

“Sumatra bei Kepahiang”. Later (Martens 1867) more precisely: “Sumatra, am Ostabhang der mittleren Bergkette bei Kepahiang”.

###### Material examined.

Mt Gede, West Java, 4000 ft., H. Fruhstorfer, 1898, E. R. Sykes Collection, Acc. no. 1825, NHMUK 20150127 (3 shells).

###### Remarks.

Protoconch low, no spiral lines visible; R1 rather regularly, finely ribbed without spiral lines; R2 very long, with regular ribs, which are curved towards the aperture, and reach each other (typical *Dicharax*).

##### Dicharax maosmaiensis

Taxon classificationAnimaliaGastropodaCyclophoridae

(Godwin-Austen, 1922)

1EE219F4-6179-52FB-B82C-C4E51AC5310E

Alycaeus maosmaiensis Godwin-Austen, 1922: 365, text figs.

###### Type locality.

“Khasi Hills, near Cherrapunji, at the mouth of the Maosmai cave”.

###### Material examined.

Maosmai, nr Cherrapoonjee, Khasi, NHMUK 20191067 (1 syntype separated in a vial with pink wool + 4 additional syntypes).

###### Remarks.

Protoconch low, rather matte, no spiral lines visible; R1 with very widely spaced wrinkles without spiral striation; R2 moderately long, the ribs are bent and do not reach each other (typical *Dicharax* structure).

##### Dicharax microcostatus

Taxon classificationAnimaliaGastropodaCyclophoridae

Páll-Gergely, 2017

EF211232-F41C-5A09-80D0-BB7EAC0F8B55

Dicharax microcostatus Páll-Gergely in Páll-Gergely et al., 2017: 66, fig. 34C.

###### Type locality.

“China, Sichuan, Taian Zhen, Qingchenghoushan, Dujiangyan Shi, Cuiyinghu to upper station of Jinli cable station, 1273 m, 30°56.27110'N, 103°28.75198'E”.

###### Material examined.

Holotype (HNHM 99708) and a few paratypes, see the original description.

###### Remarks.

Protoconch low, we only had weathered material available to study and therefore the sculpture could not be examined; R1 regularly, finely ribbed; R2 very densely ribbed, ribs curved towards aperture.

##### Dicharax microdiscus

Taxon classificationAnimaliaGastropodaCyclophoridae

(Möllendorff, 1887)

7B0ECCDB-7842-5AB1-98E9-2ED0C0A83B97

Alycaeus microdiscus Möllendorff, 1887a: 311.
Alycaeus microdiscus – Möllendorff 1891: 343.
Alycaeus (Chamalycaeus) microdiscus – Kobelt 1902: 358.
Chamalycaeus (Chamalycaeus) microdiscus – Zilch 1957: 143, pl. 5, fig. 8; Egorov 2013: 35, fig. 62b.
Dicharax microdiscus – Páll-Gergely 2017: 25, fig. 15D.

###### Type locality.

“Ad Buket Pondong”.

###### Material examined.

Malakka, Bukit Pondong (Perak), coll. Möllendorff, SMF 109496 (lectotype, designated by Zilch 1957); Same data, SMF 109497 (3 paralectotypes); Perak, leg. Hungerford, NHMUK 1891.3.17.794–796 (3 possible paralectotypes).

###### Remarks.

Protoconch low, glossy, no spiral lines visible; R1 irregularly, finely ribbed without spiral lines; R2 with ribs curved towards the aperture (typical *Dicharax*).

##### Dicharax micropolitus

Taxon classificationAnimaliaGastropodaCyclophoridae

Páll-Gergely & Hunyadi, 2017

2D0C614C-AA34-55F2-B643-7759A79F83D1

Dicharax micropolitus Páll-Gergely & Hunyadi in Páll-Gergely et al., 2017: 66–68, figs 34D, 42C, D, 43.

###### Type locality.

“China, Sichuan, Taian Zhen, Qingchenghoushan, Dujiangyan Shi, Cuiyinghu to upper station of Jinli cable station, 1273 m, 30°56.27110'N, 103°28.75198'E”.

###### Material examined.

Holotype (HNHM 99709) and a few paratypes, see the original description.

###### Remarks.

Protoconch low, glossy; R1 almost smooth, with only very inconspicuous, irregular growth lines; R2 very densely ribbed, ribs curved towards the aperture.

##### Dicharax nitidus

Taxon classificationAnimaliaGastropodaCyclophoridae

(W. T. Blanford, 1862)

D4B249CC-5EF9-51F1-BA17-4CBD52DF76B4

Alycaeus nitidus W. T. Blanford, 1862: 141.
Alycaeus nitidus – Reeve 1878: pl 3, species 25; Godwin-Austen 1914: 421–422, pl. 151, figs 4, 4a.
Alycaeus (Chamalycaeus) nitidus – Kobelt 1902: 360; Gude 1921: 230–231.
Chamalycaeus (Chamalycaeus) nitidus – Ramakrishna et al. 2010: 54.

###### Type locality.

“prope Tongoop in Arakan”.

###### Material examined.

Manya Khyoung, Arakan, coll. Blanford, NHMUK 1906.4.4.54 (3 possible syntypes).

###### Remarks.

Protoconch low, glossy, no spiral lines visible; R1 glossy, without spiral lines; R2 short, with a few ribs; each rib lamella-like ribs, which is slightly curved towards the aperture.

##### Dicharax notatus

Taxon classificationAnimaliaGastropodaCyclophoridae

(Godwin-Austen, 1876)

D108A9B7-ED2F-598A-AB8A-56A089BA6418

Alycaeus notatus Godwin-Austen, 1876: 176, pl. 7, figs 9, 9a, 9b.
Alycaeus notatus – Godwin-Austen 1886: 191–192, pl. 43, figs 2, 2a–c; Godwin-Austen 1914: 358–359, pl. 145, figs 8, 8a.
Alycaeus (Dicharax) notatus – Kobelt 1902: 374; Gude 1921: 262.
Chamalycaeus (Dicharax) notatus – Ramakrishna et al. 2010: 64; Tripathy et al. 2018: 789.

###### Type locality.

“On the slopes of Torúpútú Peak at 3000 feet”.

###### Material examined.

Toruputu Peak, Dafla Hills, 3000, NHMUK 1903.7.1.2672 (4 syntypes); Dafla Hills, coll. Godwin-Austen, NHMUK 1903.7.1.2544 (2 syntypes). Both samples are in the same box, but in different vials.

###### Remarks.

Protoconch low without spiral lines; R1 irregularly, strongly ribbed without spiral striation; R2 long, with dense ribs which are curved towards the aperture, but do not usually reach each other.

##### Dicharax notus

Taxon classificationAnimaliaGastropodaCyclophoridae

(Godwin-Austen, 1914)

7DDE847B-D822-591C-AF06-8444EC2F176D

Alycaeus notus Godwin-Austen, 1914: 411, pl. 155, fig. 12.
Alycaeus (Dicharax) notus – Gude 1921: 262.

###### Type locality.

“Fort Stedman, Burma”.

###### Material examined.

Fort Stedman, Burma, coll. Woodthorpe, NHMUK 1903.7.1.3065 (15 syntypes).

###### Remarks.

Protoconch low, without spiral lines; R1 rather regularly, weakly ribbed without spiral striae; R2 relatively short, with ribs curved towards the aperture that reach each other.

##### Dicharax nowgongensis

Taxon classificationAnimaliaGastropodaCyclophoridae

(Godwin-Austen, 1914)

56464E44-5B9C-5AA2-9815-0EA1A5B3EB35

Alycaeus nowgongensis Godwin-Austen, 1914: 397, pl. 137, figs 4, 4a, 4b.
Alycaeus nowgongensis – Gude 1921: 213.
Alycaeus (Alycaeus) nowgongensis – Ramakrishna et al. 2010: 49.

###### Type locality.

“Koliaghur or Koliahur, Nowgoug District, Assam”.

###### Material examined.

Koliaghur nr. Tezpur, Assam, NHMUK 1903.7.1.2682 (holotype [single specimen mentioned in the original description]).

###### Remarks.

Protoconch low, without spiral lines; R1 nearly smooth, there are some rough wrinkles near the suture; R2 short, with ribs, which are curved towards the aperture that reach each other.

##### Dicharax ochraceus

Taxon classificationAnimaliaGastropodaCyclophoridae

(Godwin-Austen, 1893)

712AC62B-612C-5F2C-93B2-729C8CCD117E

Alycaeus ochraceus Godwin-Austen, 1893: 594–595.
Alycaeus ochraceus – Godwin-Austen 1897: 3, pl. 63, figs 7, 7a, 7b; Godwin-Austen 1914: 411.
Alycaeus (Dicharax) ochraceus – Kobelt 1902: 374; Gude 1921: 263.

###### Type locality.

“Ruby Mines District, Upper Burmah”.

###### Material examined.

Ruby mine Disr., Up. Burma, leg. Doherty, NHMUK 1903.7.1.2684 (2 syntypes).

###### Remarks.

Protoconch low, moderately glossy, without spiral lines; R1 finely, regularly ribbed, without spiral striae; R2 moderately long, with regular ribs; ribs curved towards the aperture (typical *Dicharax*).

##### Dicharax oligopleuris

Taxon classificationAnimaliaGastropodaCyclophoridae

(Möllendorff, 1887)

63B9FCBA-5FFF-508B-9E36-32BBC066BC94

Alycaeus oligopleuris Möllendorff, 1887a: 310–311.
Alycaeus oligopleuris – Möllendorff 1891: 342, pl. 30, figs 9, 9b.
Alycaeus (Chamalycaeus) oligopleuris – Kobelt 1902: 360–361.
Chamalycaeus (Chamalycaeus) oligopleuris – Zilch 1957: 144, pl. 5, fig. 9.
Dicharax oligopleuris – Páll-Gergely et al. 2017: 45, fig. 31E.

###### Type locality.

“Ad Buket Pondong”.

###### Material examined.

Malakka: Perak, coll. Möllendorff, SMF 109226 (lectotype, designated by Zilch 1957); Same data, SMF 109227 (2 paralectotypes).

###### Remarks.

Protoconch low, without spiral striae; R1 with widely spaced, strong ribs, which are the most prominent near the suture and become lower away from it; R2 very short, consists of ca. 15 ribs, which are curved towards the aperture.

##### Dicharax parvulus

Taxon classificationAnimaliaGastropodaCyclophoridae

(Möllendorff, 1887)

27568E74-0DAB-586A-ABF3-0DB026764733

Alycaeus parvulus Möllendorff, 1887a: 311.
Alycaeus parvulus – Möllendorff 1891: 343, pl. 30, figs 11, 11b.
Alycaeus (Chamalycaeus) parvulus – Kobelt 1902: 361.
Chamalycaeus (Chamalycaeus) parvulus – Zilch 1957: 144, pl. 5, fig. 10; Egorov 2013: 35, fig. 62a.
Dicharax parvulus – Páll-Gergely 2017: 25, fig. 15E.

###### Type locality.

“Ad Buket Pondong”.

###### Material examined.

Malakka: Bukit Pondong (Perak), coll. Möllendorff, SMF 109507 (lectotype, designated by Zilch 1957); Same data, SMF 109508 (4 paralectotypes).

###### Remarks.

Protoconch low, without spiral striae; R1 finely, regularly ribbed, without spiral striae; R2 extremely short, with ca. six ribs, which are curved towards the aperture.

##### Dicharax planorbulus

Taxon classificationAnimaliaGastropodaCyclophoridae

(Heude, 1885)

2520DF4F-1B13-531D-A689-46E1C0653B1D

Alycoeus [sic] planorbulus Heude, 1885: 96, pl. 24, figs 2, 2a–c.
Alycaeus (Chamalycaeus) planorbulus – Kobelt and Möllendorff 1897: 149; Kobelt 1902: 361.
Dicharax planorbulus – Páll-Gergely et al. 2017: 68, figs 21B, C, 42E, F, 44.

###### Type locality.

“in ditione Tchen-k’eou”.

###### Material examined.

China, Tchen-K’eou, MCZ 167136 (10 syntypes).

###### Remarks.

Protoconch low, rather matte; R1 regularly, densely ribbed, ribs low; ribs becoming slightly more widely spaced towards end of R1; R2 very densely ribbed, ribs curved towards the aperture.

##### Dicharax plicilabris

Taxon classificationAnimaliaGastropodaCyclophoridae

(Möllendorff, 1886)

B547A7EE-155F-5485-89A2-487B8AF83FFA

Alycaeus plicilabris Möllendorff, 1886: 167, pl. 5, fig. 5.
Alycaeus (Chamalycaeus) plicilabris – Kobelt and Möllendorff 1897: 149; Kobelt 1902: 361.
Chamalycaeus plicilabris plicilabris – Yen 1939: 29, pl. 2, fig. 32.
Chamalycaeus (Chamalycaeus) plicilabris plicilabris – Zilch 1957: 145, pl. 5, fig. 14.
Dicharax plicilabris – Páll-Gergely et al. 2017: 68–70, fig. 33D.

###### Type locality.

“in provincia sinensi Hunan”.

###### Material examined.

China, Prov. Hunan, coll. O. Boettger ex coll. Möllendorff, SMF 39229 (lectotype, designated by Yen 1939); same data, SMF 39229 (4 paralectotypes).

###### Remarks.

Protoconch low, rather glossy; R1 regularly, densely ribbed; R2 with ribs curving towards the aperture.

##### Dicharax politus

Taxon classificationAnimaliaGastropodaCyclophoridae

(W. T. Blanford, 1865)

2F27C70E-FFA0-5F20-9133-2F653082C751

Alycaeus politus W. T. Blanford, 1865: 83–84.
Alycaeus politus – Reeve 1878: pl. 5, species 39; Godwin-Austen 1914: 422, pl. 139, figs 5, 5a; Gude, 1921: 214–215.
Alycaeus (Alycaeus) politus – Kobelt 1902: 348.

###### Type locality.

“Phuong do, near Cape Negrais, Arakan”.

###### Material examined.

Phungdo, Arakan, coll. Blanford, NHMUK 1906.4.4.178 (3 probable syntypes).

###### Remarks.

Protoconch low, glossy, no spiral lines visible; R1 glossy, without notable sculpture; R2 short, with regular ribs, which are curved towards the aperture.

##### Dicharax pratatensis

Taxon classificationAnimaliaGastropodaCyclophoridae

(Panha & Burch, 1997)

BCF6FA72-FECB-5B43-B916-C7D938845396

Alycaeus pratatensis Panha & Burch, 1997: 119–122, figs 2a–c.

###### Type locality.

“Pratat cave, Erawan Natural Park, Karnchanaburi Province at 14°27'58"N, 99°49'49"E, 230 meters elevation.”

###### Material examined.

Pratat cave, Erawan N. P., Karnchanaburi Province, Thailand, 26.10.1996, ex coll. S. Panha, 2008, SMF 331452 (2 paratypes).

###### Remarks.

Protoconch low, without spiral lines; R1 irregularly wrinkled, without spiral lines; R2 short, with ribs, which are curved towards the aperture.

##### Dicharax robustus

Taxon classificationAnimaliaGastropodaCyclophoridae

Páll-Gergely & Hunyadi, 2017

E0701A85-D9D2-5294-88A4-3CA8056B238C

Dicharax micropolitus Páll-Gergely & Hunyadi in Páll-Gergely et al., 2017: 70–71, figs 19C, 29A, B, 45.

###### Type locality.

“China, Yunnan, Kunming Shi, Yuqiqu, Bijianshan, Guanyinsi (temple), approximate GPS data: 24°16.271'N, 102°49.726'E”.

###### Material examined.

HNHM 99704 (holotype) and a few paratypes, see original description.

###### Remarks.

Protoconch, normally elevated (not higher or lower than what would be expected from the overall shell shape), it is matte, without any notable sculpture; R1 regularly ribbed; in fresh shells ribs sharp and strongly curved towards aperture; R2 very densely ribbed, ribs with T-shaped cross sectional view.

##### Dicharax stuparum

Taxon classificationAnimaliaGastropodaCyclophoridae

Páll-Gergely & Hunyadi, 2018

3EB07984-8B34-5F02-819F-2CE8654B06A1

Dicharax stuparum Páll-Gergely & Hunyadi, 2018: 62, figs 1F–K.

###### Type locality.

“Thailand, Chiang Rai Province, Doi Tung, 50 m before Wat Phra That Doi Tung, around the car park, 1350 m a.s.l., 20°19.540'N, 99°49.987'E”.

###### Material examined.

HNHM 100178 (holotype).

###### Remarks.

Protoconch glossy; R1 also glossy, with irregular growth lines; R2 bears dense, curved ribs (ca. 46–48 in total), for more details see the original description.

##### Dicharax sylheticus

Taxon classificationAnimaliaGastropodaCyclophoridae

(Godwin-Austen, 1914)

4F1B066F-117B-57B2-B5DE-113AD16A3D25

Alycaeus sylheticus Godwin-Austen, 1914: 382, pl. 154, figs 4, 4a.
Alycaeus sylheticus – Gude 1921: 220.

###### Type locality.

“South Sylhet Hills”.

###### Material examined.

S. Sylhet Hills, leg. W. Channel, NHMUK 1903.7.1.55 (holotype [single specimen mentioned in the original description]).

###### Remarks.

Protoconch low, no spiral lines visible; R1 with widely spaced ribs with the very slight indication of spiral lines; R2 relatively short, with some blunt (weathered), regular ribs, which have lamella-like horizontal projections reaching to the neighbouring ribs (typical *Dicharax*).

##### Dicharax tangmaiensis

Taxon classificationAnimaliaGastropodaCyclophoridae

(Chen & Zhang, 2001)

5BEA3506-4B61-5101-9B49-2D46333F7272

Fig. 17

Chamalycaeus tangmaiensis Chen & Zhang, 2001: 184–185, 188–189, figs 1–4.
Dicharax (?) tangmaiensis – Páll-Gergely et al. 2017: 107–108.

###### Type locality.

“Tongmai Town, (30°01'N, 95°E), Bomi County, Tibet Autonomous Region, China”.

###### Material examined.

CASIZ TM 0010054 (holotype) deposited in IZCAS: Tong-Mai Town, Bo-Mi County, Tibet Autonomous Region, China, leg. Chen De-niu, 1980.6.20; CASIZ TM 0010056 (paratype): same as holotype.

###### Remarks.

Protoconch low, without notable sculpture; R1 rather dense, low ribs, no spiral striae visible; R2 + R3 90° combined; R2 with low ribs, ribs similar to those on R1; R3 with a prominent, blunt swelling.

The shell of *Dicharax tangmaiensis* is similar to some other northeastern Indian *Dicharax* species with fringed peristome (e.g., *D. cucullatus*). Future investigation should reveal whether this species is really distinct from other Himalayan species, since no comparisons were made in the original description.

**Figure 17.**
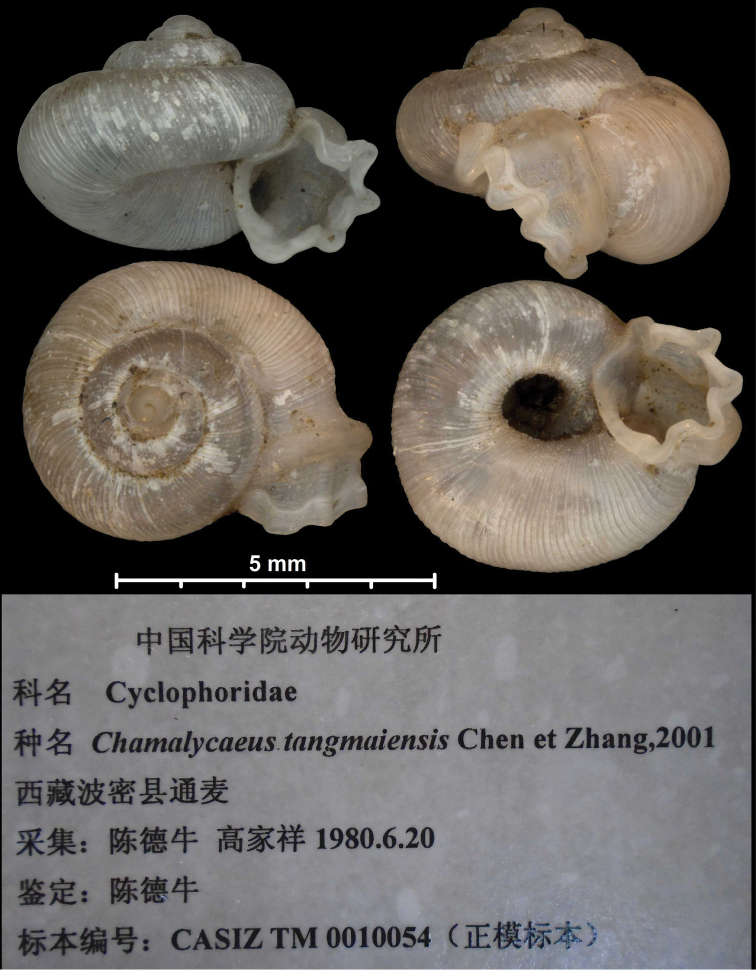
*Dicharax tangmaiensis* (Chen & Zhang, 2001), holotype (CASIZ TM 0010054). Photographs: Kaibaryer Meng.

##### Dicharax theobaldi

Taxon classificationAnimaliaGastropodaCyclophoridae

(W. T. Blanford, 1862)

838693A7-69A7-5590-825F-BEEB773C3E30

Alycaeus Theobaldi W. T. Blanford, 1862: 142–143.
Alycaeus theobaldi – Reeve 1878: pl. 5, species 44; Godwin-Austen 1914: 359–360, pl. 149, figs 3, 3a, 3b; Godwin-Austen 1914: 382–383, pl. 145, figs 4, 4a.
Alycaeus (Dicharax) theobaldi – Kobelt 1902: 377–378; Gude 1921: 272–273.
Chamalycaeus (Dicharax) theobaldi – Ramakrishna et al. 2010: 67.

###### Type locality.

“in montibus Khasi”.

###### Material examined.

Khasi Hills, coll. W. T. Blanford, NHMUK 1906.4.4.60 (2 possible syntypes).

###### Remarks.

Protoconch low, lacks any signs of spiral striation; R1 with widely spaced ribs but no spiral lines; R2 short, with ribs which are curved towards the aperture.

##### Dicharax theobaldi diyungensis

Taxon classificationAnimaliaGastropodaCyclophoridae

(Godwin-Austen, 1914)

B317C79A-75C6-5949-ACEE-8D85492FEFD7

Alycaeus theobaldi var. diyungensis Godwin-Austen, 1914: 401–402, pl. 138, fig. 4.
Alycaeus (Dicharax) theobaldi Var. diyungensis – Gude 1921: 274.

###### Type locality.

“ad Darjiling”.

###### Material examined.

Diyung Valley, N of Asalu, NHMUK 1903.7.1.2546 (12 syntypes).

###### Remarks.

Same as in *theobaldi solidus*, but R2 is longer.

##### Dicharax theobaldi solidus

Taxon classificationAnimaliaGastropodaCyclophoridae

(Godwin-Austen, 1914)

0C972A00-B0D7-5529-9DBD-6D3D0E14F908

Alycaeus theobaldi var. solidus Godwin-Austen, 1914: 383–384, pl. 155, fig. 10.
Alycaeus (Dicharax) theobaldi Var. solida – Gude 1921: 273–274.

###### Type locality.

“Garo Hills”.

###### Material examined.

Garo Hills, NHMUK 1903.7.1.2560 (4 syntypes).

###### Remarks.

Protoconch somewhat elevated but lacks any signs of spiral striation; R1 with widely spaced ribs but no spiral lines; R2 short, with ribs which are curved towards the aperture.

##### Dicharax vestitus

Taxon classificationAnimaliaGastropodaCyclophoridae

(W. T. Blanford, 1862)

4ADADD11-73EC-5E23-8B45-1DDB3B0E9E21

Alycaeus vestitus W. T. Blanford, 1862: 138–139.
Alycaeus vestitus var. minor W. T. Blanford, 1862: 138.
Alycaeus vestitus – Reeve 1878: pl. 1, species 3; Godwin-Austen 1914: 424–425, pl. 139, figs 2, 2a; Gude 1921: 220–221.
Alycaeus (Alycaeus) vestitus – Kobelt 1902: 352.
Dicharax vestitus – Páll-Gergely et al. 2017: 71.

###### Type locality.

“in montibus Arakanensibus”.

###### Material examined.

Moditoung, NHMUK 1906.4.4.53 (holotype [single specimen of both the nominotypical form and var. minor were mentioned in the original description]), and two additional non-type specimens in the same lot from Alori Khyoung and Mamya Khyoung.

###### Remarks.

All three specimens are strongly weathered; therefore, their sculpture could not be fully distinguished. Protoconch low, without recognisable sculpture; R1 seemingly smooth; R2 with dense ribs, which were all broken.

##### Dicharax vestitus akyabensis

Taxon classificationAnimaliaGastropodaCyclophoridae

(Godwin-Austen, 1914)

8982AFBD-DDCE-55F6-867B-4932BDD6CF73

Alycaeus vestitus var. akyabensis Godwin-Austen, 1914: 425–426, pl. 155, fig. 7.
Alycaeus vestitus var. akyabensis – Gude 1921: 221.

###### Type locality.

“Baumi, Akyab”.

###### Material examined.

Baumi, Akyab, NHMUK 1888.12.4.251–252 (2 syntypes).

###### Remarks.

Protoconch low, matte; R1 with very low, but rather regular ribbing near the suture (ribs nearly absent at the edge of the body whorl); R2 of normal length, the ribs are curved towards the aperture, nearly reaching each other.

#### Atypical or questionable *Dicharax* species

##### Dicharax (?) abdoui

Taxon classificationAnimaliaGastropodaCyclophoridae

Páll-Gergely, 2017

91825F23-4F44-50BC-9341-01B2BD3E0E36

Dicharax abdoui Páll-Gergely in Páll-Gergely et al., 2017: 14, fig. 6.
Dicharax abdoui – Inkhavilay et al. 2019: 14, fig. 4F.

###### Type locality.

“Laos, Khammouane Province, approx. 9 km NE of Thakhek (Muang Khammouan), 190 m, 17°26.757'N, 104°52.937'E, on and under rocks in dry secondary forest on and under NW exposed cliffs”.

###### Material examined.

MNHN IM-2012-27329 (holotype) and 2 paratypes (MNHN-IM-2012-27328).

###### Remarks.

Protoconch low, nearly smooth, with extremely fine pits arranged in spiral rows (not homologous with the spiral striation of *Metalycaeus* species); R1 nearly smooth, with low, widely spaced ribs near suture and in umbilicus; R2 very short, with low, dense regular ribs (ca. 20).

##### Dicharax (?) akhaensis

Taxon classificationAnimaliaGastropodaCyclophoridae

(Godwin-Austen, 1914)

A5964DB0-07CB-5E07-BA82-C33B1A11B8D1

Alycaeus akhaensis Godwin-Austen, 1914: 352, pl. 141, figs 1, 1a, 1b.
Alycaeus (Raptomphalus) akhaensis – Gude 1921: 286.
Chamalycaeus (Raptomphalus) akhaensis – Ramakrishna et al. 2010: 68.

###### Type locality.

“Barowli Gorge, Durrang District, Assam, foot of the Akha Hills”.

###### Material examined.

Akha Hills, Barowli River, coll. Godwin-Austen, NHMUK 1903.7.1.2683 (holotype [single specimen mentioned in the original description]).

###### Remarks.

The entire shell is quite weathered, but the following observations could be made: protoconch low, without spiral striae; R1 glossy, with widely spaced, strong ribs (present only near the suture) and without spiral lines; R2 short, with dense, low ribs; R2 of fresh shells is probably smooth.

##### Dicharax (?) alticola

Taxon classificationAnimaliaGastropodaCyclophoridae

Páll-Gergely & Hunyadi, 2017

0F5F409C-311A-5AF2-ABEB-2D0726781C72

Dicharax alticola Páll-Gergely & Hunyadi in Páll-Gergely et al., 2017: 14–20, figs 8A, 9A–D, 10A, B, 11, 12A, B, 13A, B.

###### Type locality.

“China, Sichuan, Liangshan Yizu Zizhizhou, Yanyuan Xian, Bainiao Zhen, Kedeng Rongdong (cave), 2618 m, 27°43.103'N, 101°31.021'E”.

###### Material examined.

Holotype (HNHM 99702) and several paratypes (see Páll-Gergely et al. 2017).

###### Remarks.

Protoconch low, seemingly smooth but rather matte; R1 somewhat regularly ribbed; rib density of R2 higher than that of R1, ribs on R2 low, not curved, rather sharp, connected to each other near tube (similar to Japanese “*Awalycaeus*” and “*Cipangocharax*” species); for more details see the original description.

##### Dicharax (?) ataranensis

Taxon classificationAnimaliaGastropodaCyclophoridae

(Godwin-Austen, 1914)

DE71AA9D-2B5C-5BA9-A167-FDBE28F59C6F

Fig. 18

Alycaeus ataranensis Godwin-Austen, 1914: 426, pl. 148, figs 4, 4a, 4b.
Alycaeus (Dicharax) ataranensis – Gude 1921: 237.

###### Type locality.

“1 Ataran”.

###### Material examined.

Ataran, Burma, ex Dr. F. Stoliczka, NZSI M.8073 (holotype [single specimen mentioned in the original description]).

###### Remarks.

The holotype is in a strongly corroded state; therefore, the sculpture could not be examined in detail. This species is putatively placed in *Dicharax* due to the overall smooth shell and the fringed aperture.

**Figure 18.**
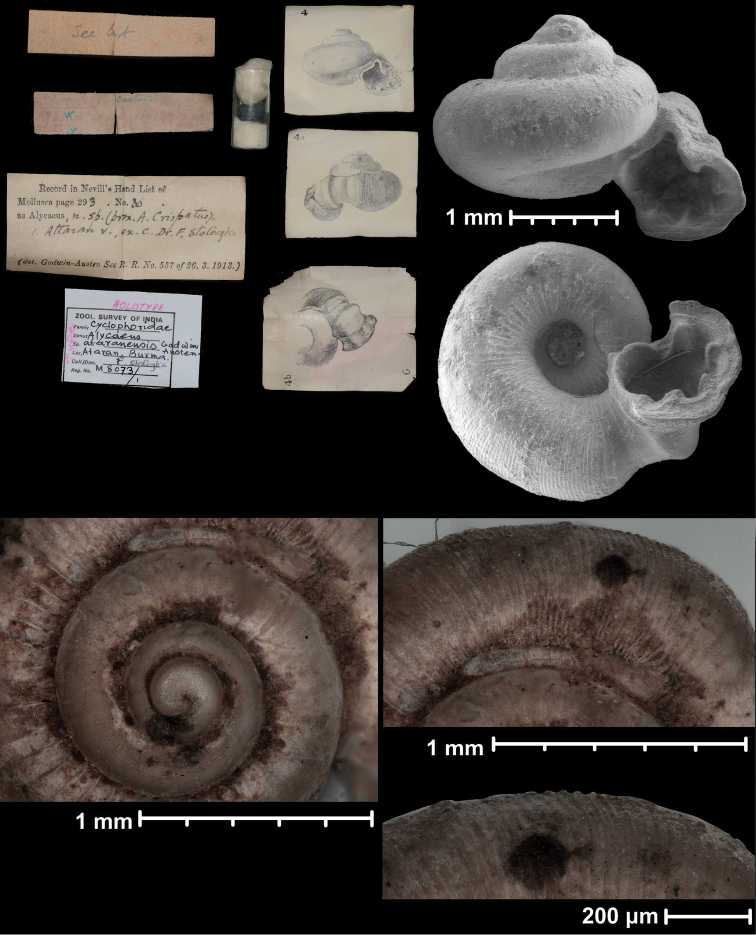
*Dicharax ataranensis* (Godwin-Austen, 1914), holotype (NZSI M.8073). All images: Sheikh Sajan.

##### Dicharax (?) barowliensis

Taxon classificationAnimaliaGastropodaCyclophoridae

(Godwin-Austen, 1914)

4D0B0942-6276-5A10-BF00-6269FCB9DF99

Alycaeus barowliensis Godwin-Austen, 1914: 352, pl. 141, fig. 4.
Alycaeus barowliensis – Gude 1921: 205.
Alycaeus (Alycaeus) burowliensis [sic] – Ramakrishna et al. 2010: 46.

###### Type locality.

“Barowli River, Akha Hills, Durrang, Assam”.

###### Material examined.

Barowli R. Durrang, Assam, coll. Godwin-Austen, NHMUK 1903.7.1.2723 (holotype [single specimen mentioned in the original description]).

###### Remarks.

Only the holotype is known. The outermost shell layer is entirely weathered and the sculpture is not visible. The protoconch is seemingly low. Based on this character, *A. barowliensis* is tentatively classified in the genus *Dicharax*.

##### Dicharax (?) bawai

Taxon classificationAnimaliaGastropodaCyclophoridae

Aravind & Páll-Gergely, 2018

44C342B3-D4BE-5066-BCBA-B790C790C71E

Dicharax (?) bawai Aravind & Páll-Gergely, 2018: 56, figs 1A, 2, 3.

###### Type locality.

“India, Karnataka State, Chamarajanagar District, Malai Mahadeshwara Hills, 1010 m a.s.l., 12.04911°N, 77.56369°E, from the base of a big tree, next to the road near the temple (the habitat has lots of lianas and stones with a good amount of litter in dry deciduous forest)”.

###### Material examined.

ZSI/WGRC/9865 (holotype), for other examined shells see the original description.

###### Remarks.

Protoconch somewhat elevated, rather glossy without notable sculpture; first whorl of R1 irregularly, finely ribbed, with ribs becoming stronger, rarer and more regular towards end of R1; R2 with 24–28 elevated, blunt, regular ribs; for description of cross-sectional view see original description.

##### Dicharax (?) bicrenatus

Taxon classificationAnimaliaGastropodaCyclophoridae

(Godwin-Austen, 1874)

D126CCD0-A814-5759-A5F2-E6D06D481E3D

Alycaeus bicrenatus Godwin-Austen, 1874: 148, pl. 3, fig. 5.
Alycaeus (Dicharax) bicrenatus – Kobelt 1902: 365; Gude 1921: 238–239.
Alycaeus bicrenatus – Godwin-Austen 1884: pl. 51, fig. 4; Godwin-Austen 1914: 386–387, pl. 144, figs 5, 5a, 5b.
Chamalycaeus (Dicharax) bicrenatus – Ramakrishna et al. 2010: 56.

###### Type locality.

“Kopamedza Peak Naga Hill, 8–9,000 feet, in forest”.

###### Material examined.

Kopamedza, Naga Hills, NHMUK 1903.7.1.2490 (7 syntypes in two vials).

###### Remarks.

Protoconch low, glossy, no spiral lines visible; R1 very finely, regularly ribbed without spiral lines; R2 moderately long, with regular ribs, which are curved towards the aperture, however the space between the ribs is much larger than in typical *Dicharax*.

##### Dicharax (?) bifrons

Taxon classificationAnimaliaGastropodaCyclophoridae

(Theobald, 1870)

46A3DFAD-1027-591F-89DB-2D09989F269C

Alycaeus bifrons Theobald, 1870: 396, pl. 18, fig. 1.
Alycaeus bifrons – Reeve 1878: pl. 6, species 48; Godwin-Austen 1914: 407, pl. 139, figs 3, 3a.
Alycaeus (Dicharax) bifrons – Kobelt 1902: 365–366; Gude 1921: 239.

###### Type locality.

“Shan States”.

###### Material examined.

Shan States, NHMUK 1888.12.4.956–958 (3 syntypes).

###### Remarks.

Protoconch low, rather matte, no spiral lines visible; R1 irregularly wrinkled near the suture, this sculpture becomes stronger anteriorly, and near the end of R1 there are widely spaced, strong ribs, which extend not only to the suture area but to the edge of the body whorl; no signs of spiral striae visible on R1; R2 moderately long, with widely spaced, lamella-like, straight, rather low ribs.

##### Dicharax (?) birugosus

Taxon classificationAnimaliaGastropodaCyclophoridae

(Godwin-Austen, 1893)

A766C183-1E28-5503-AD45-DF7712F3DC64

Fig. 19

Alycaeus bi-rugosus Godwin-Austen, 1893: 593.
Alycaeus bi-rugosus – Godwin-Austen 1897: 387, pl. 63, figs 5, 5a.
Alycaeus (Dicharax) birugosus – Kobelt 1902: 366.
Alycaeus birugosus – Godwin-Austen 1914: 370; Godwin-Austen 1914: 387.
Alycaeus canaliculus Godwin-Austen, 1914: 371, pl. 154, fig. 11. syn. nov.
Alycaeus birugosus var. – Godwin-Austen 1914: 370, pl. 154, figs 7, 7a.
Alycaeus birugosus var. minor Godwin-Austen, 1914: 370, pl. 155, figs 9, 9a.
Alycaeus (Chamalycaeus) canaliculus – Gude 1921: 225.
Alycaeus (Dicharax) birugosus – Gude 1921: 239–240.
Alycaeus (Dicharax) birugosus var. minor – Gude 1921: 240.
Chamalycaeus (Chamalycaeus) canaliculus – Ramakrishna et al. 2010: 53.
Chamalycaeus (Dicharax) birugosus – Ramakrishna et al. 2010: 57.

###### Type locality.

“Khasi Hills and Manipur” (*A. birugosus*); “Garo Hills” (A. birugosus var. minor); “Teria Ghat, foot of the Khasi Hills” (*A. canaliculus*).

###### Material examined.

**Figure 19.**
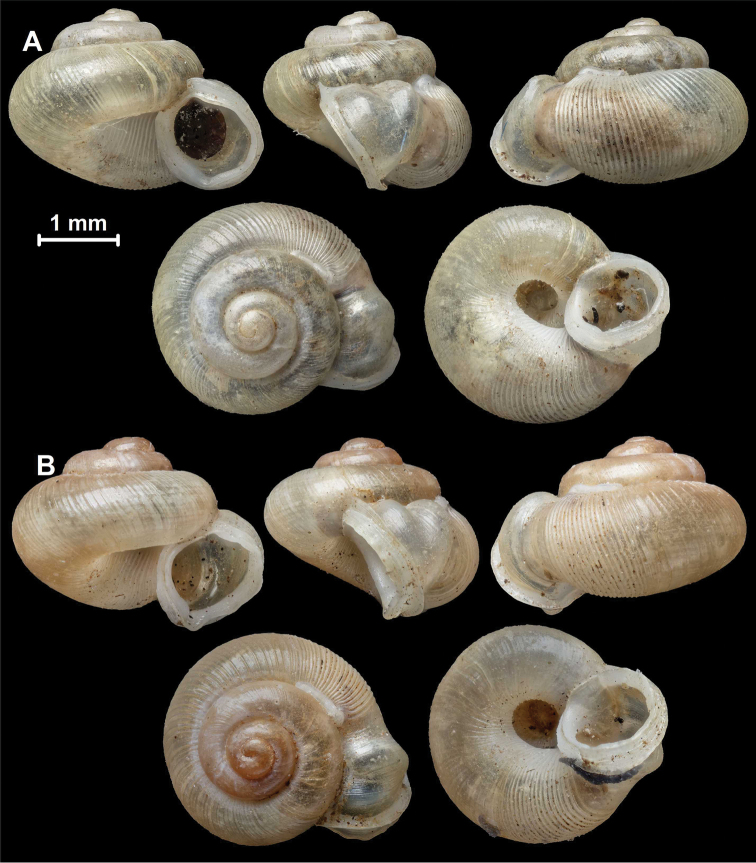
Shells of Dicharax (?) birugosus (Godwin-Austen, 1893) **A***Alycaeus canaliculus* Godwin-Austen, 1914, (NHMUK 1903.7.1.2764) **B***A. birugosus* Godwin-Austen, 1893, syntype (NHMUK 1903.7.1.2628). Photographs: Kevin Webb (NHM).

Khasi Hills, leg. Godwin-Austen, NHMUK 1903.7.1.2628 (2 syntypes of *A. birugosus*, Fig. 19B); Jawai, Jiantia Hills, 282a, leg. Godwin-Austen, NHMUK 1903.7.1.2571 (7 shells, labelled as “*birugosus* var.”); Garo Hills, NHMUK 1903.7.1.2755 (1 syntype of Alycaeus birugosus var. minor, labelled as duorugosus var. minor); Teria Ghat, Khasi, leg. Godwin-Austen, NHMUK 1903.7.1.2764 (1 syntype of *A. canaliculus*, Fig. 19A).

###### Remarks.

*Alycaeus birugosus* and *A. canaliculus* are practically identical and both of them inhabit the Khasi Hills. Thus, the latter is moved to the synonymy of the former.

Protoconch low, rather glossy, without spiral lines; R1 without spiral lines; R2 short, with blunt, straight ribs. Specimens labelled as “*birugosus* var.” are smooth on R1, whereas typical shells are more strongly sculptured.

Comments relating to “var. *minor*”: protoconch low, rather glossy, without spiral lines; R1 glossy, without spiral lines; R2 short, with regular, blunt, not bent ribs.

##### Dicharax (?) blanfordi

Taxon classificationAnimaliaGastropodaCyclophoridae

(Godwin-Austen, 1914)

F69EB354-5EB5-53A7-9C6C-C7A863BF1EC3

Alycaeus blanfordi Godwin-Austen, 1914: 418, pl. 148, fig. 3.
Alycaeus blanfordi – Gude 1921: 206.

###### Type locality.

“Chwegali, Arakan Hills”.

###### Material examined.

Chwegalé, Arakan Hills, NHMUK 1906.4.4.177 (holotype [single specimen mentioned in the original description]).

###### Remarks.

Protoconch low, no spiral lines visible; R1 without spiral lines; R2 long, with widely spaced but blunt ribs, which are curved towards the aperture (especially near the tube, far from the tube the ribs are straighter); curved ribs are situated far apart from each other.

##### Dicharax (?) burroiensis

Taxon classificationAnimaliaGastropodaCyclophoridae

(Godwin-Austen, 1914)

7A12D638-46ED-567C-B2CF-17A20BE990C6

Alycaeus burroiensis Godwin-Austen, 1914: 354, pl. 141, figs 6, 6a.
Alycaeus (Cycloryx) burroiensis – Gude 1921: 277.

###### Type locality.

“Burroi Gorge, Dafla Hills”.

###### Material examined.

Burroi Rr., Dafla, NHMUK 1903.7.1.2653 (1 syntype).

###### Remarks.

The single shell is strongly weathered. Protoconch low, its sculpture is not visible; there are no signs of spiral lines on R1; R2 with dense ribs, their fine structure is not visible. Based on the low protoconch this species is putatively classified in *Dicharax*.

##### Dicharax (?) candrakirana

Taxon classificationAnimaliaGastropodaCyclophoridae

Nurinsiyah & Hausdorf, 2017

918627B8-BB1A-5C04-BCF3-B389709B028D

Dicharax (?) candrakirana Nurinsiyah & Hausdorf, 2017: 589–591, fig. 1.

###### Type locality.

“Indonesia, East Java: Malang, Sempu Island, limestone rocks in lowland rainforest at entrance of Kelabang Cave, 44 m a.s.l., 8°26'58"S 112°41'28"E”.

###### Material examined.

Photographs of the holotype (MZB 19025) were examined.

###### Remarks.

Protoconch low without spiral striae; R1–R3 smooth but spirally striated on the umbilical side. This spiral striation is assumed not to be homologous with that of *Metalycaeus* species (i.e., it is probably part of the lower shell layers, not elevated from the shell surface), and similar to the structure observed in some *D. depressus* shells (see Páll-Gergely et al. 2017). R2 smooth from above, with ca. seven narrow lines, no elevated ribs discernible.

##### Dicharax (?) crassicollis

Taxon classificationAnimaliaGastropodaCyclophoridae

(Benthem-Jutting, 1959)

644CAD4A-9C1C-590F-B077-619AA4FE5183

Chamalycaeus crassicollis van Benthem Jutting, 1959: 76–77, fig. 4.

###### Type locality.

“Sirung Galing, Karo Highlands”.

###### Remarks.

No specimens were examined. The general shape and the sculpture of the species is similar to *Dicharax longituba* according to the original description. Therefore, *Alycaeus crassicollis* is tentatively classified in *Dicharax*.

##### Dicharax (?) crispatus

Taxon classificationAnimaliaGastropodaCyclophoridae

(Godwin-Austen, 1871)

F39A5CAB-EAD3-59E0-A7BB-9CABD571E526

Alycaeus crispatus Godwin-Austen, 1871: 91–92, pl. 4, fig. 1.
Alycaeus crispatus – Godwin-Austen 1875: 8, pl. 4, fig. 3.
Alycaeus (Dicharax) crispatus – Kobelt 1902: 367; Gude 1921: 242–243.
Alycaeus crispatus – Godwin-Austen 1914: 371–372, pl. 145, figs 1, 1a, 1b; Godwin-Austen 1914: 389.
Chamalycaeus (Dicharax) crispatus – Ramakrishna et al. 2010: 58; Tripathy et al. 2018: 789.

###### Type locality.

“Khasia, Jiantia and N. Cachar Hills”.

###### Material examined.

Shibak, Habiang Garo Hills, NHMUK 1903.7.1.2635 (5 syntypes); Same container (probably same locality), NHMUK 1903.7.1.2759 (11 syntypes).

Godwin-Austen (1914: 372) explained that the *Alycaeus crispatus* variety from north Cachar in his previous paper (Godwin-Austen 1871: 93) was renamed *A. asaluensis*. The originally figured sample (Godwin-Austen 1871: pl. 4, fig. 1) is from Shibak, Gabir valley (Godwin-Austen, 1914: 372).

###### Remarks.

Protoconch moderately elevated, matte, no spiral lines visible; R1 rather regularly ribbed, also without spiral lines; R2 relatively short, with regular, widely spaced, sharp ribs.

The placement of the species in the genus *Dicharax* is based on the absence of spiral striation on the entire shell; however, the sharp R2 ribs are characteristic of the genus *Chamalycaeus*. The shape of protoconch shows some variation within species. Namely, typical *crispatus* and typical *cristatus minimus* shells have only slightly elevated protoconchs, whereas it is characteristically *Chamalycaeus*-like (strongly elevated) in *D. crispatus makarsae* specimens.

##### Dicharax (?) crispatus makarsae

Taxon classificationAnimaliaGastropodaCyclophoridae

(Godwin-Austen, 1914)

BCDE5531-3F37-58C2-9C63-4E1EFA0B9A98

Alycaeus crispatus var. makarsae Godwin-Austen, 1914: 372, pl. 158, fig. 13.
Alycaeus (Dicharax) crispatus var. makarsae – Gude 1921: 243.

###### Type locality.

“Makarsa, N. Khasi Hills (or more correct, Maokarsa; the common Khasi prefix “Mao” meaning a stone”.

###### Material examined.

Makarsa, Khasi, leg. Godwin-Austen, NHMUK 1903.7.1.2638 (8 syntypes).

###### Remarks.

See under *Chamalycaeus crispatus*.

##### Dicharax (?) crispatus minimus

Taxon classificationAnimaliaGastropodaCyclophoridae

(Godwin-Austen, 1914)

7582C99D-6ED6-5610-9F9C-121FDD41ACEC

Alycaeus crispatus var. minimus Godwin-Austen, 1914: 373, pl. 148, figs 5, 5a.
Alycaeus (Dicharax) crispatus var. minima – Gude 1921: 243–244.

###### Type locality.

“Habiang Garo Hills, West Khasi”.

###### Material examined.

Habiang Garo Hills, W. Khasi, leg. Godwin-Austen, NHMUK 1906.4.4.176 (holotype [single specimen mentioned in the original description]).

###### Remarks.

Protoconch rather low, R1 with strong, widely spaced ribs which are most prominent near the suture and disappear on the edge of the body whorl; R2 of normal length, ribs blunt, and at the anterior end of the region ribs curved towards aperture.

##### Dicharax (?) crispatus rywukensis

Taxon classificationAnimaliaGastropodaCyclophoridae

(Godwin-Austen, 1914)

4ED65DA8-51F4-5DBC-A769-9AA1A8CF46A7

Alycaeus crispatus var. rywukensis Godwin-Austen, 1914: 373–374, pl. 154, figs 3, 3a.
Alycaeus (Dicharax) crispatus var. rywukensis – Gude 1921: 244.

###### Type locality.

“Rywuk Valley of the Garo Hills”.

###### Material examined.

Rywuk, Garo Hills, South base of, coll. Godwin-Austen, NHMUK 1903.7.1.2637 (2 syntypes).

###### Remarks.

Protoconch moderately elevated, smooth; R1 with strong, widely spaced ribs without spiral striation; R2 of normal length and ribs curved towards aperture.

##### Dicharax (?) daflaensis

Taxon classificationAnimaliaGastropodaCyclophoridae

(Godwin-Austen, 1876)

9A13BCB8-4A4E-55BF-9F1B-02B1EF28E127

Alycaeus daflaensis Godwin-Austen, 1876: 176–177, pl. 7, figs 12, 12a, 12b.
Alycaeus (Dicharax) daflaensis – Kobelt 1902: 368; Gude 1921: 245–246.
Alycaeus daflaensis – Godwin-Austen 1914: 354–355, pl. 145, figs 11, 11a, 11b.
Chamalycaeus (Dicharax) daflaensis – Ramakrishna et al. 2010: 58; Tripathy et al. 2018: 789.

###### Type locality.

“Torúpútú Peak, 7000 feet”.

###### Material examined.

Toruputu Peak, Dafla Hills, coll. Godwin-Austen, NHMUK 1903.7.1.2497 (lectotype: here designated, and 6 paralectotypes). The type sample contained two vials, one with two and the other with four shells. The one with four shells contained three larger shells and one which was conspicuously smaller. That smaller shell differs from the larger ones in terms of other shell characters, such as the spire height (lower than the others), the sculpture of R1 (smoother than the others), the strength of the swelling on R3 (less elevated than that of the others), and the lobes of the peristome (less conspicuous than those of the others). Therefore, one of the larger shells is selected here as lectotype to avoid further confusion.

###### Remarks.

Protoconch low, rather matte, no spiral lines visible; R1 irregularly, finely, densely ribbed, some spiral lines visible but these are probably part of the layer below the outermost one; R2 relatively short, smooth, only lighter, narrow and slightly thicker, darker stripes alternating.

##### Dicharax (?) daflaensis subdigitatus

Taxon classificationAnimaliaGastropodaCyclophoridae

(Godwin-Austen, 1876)

4BCB6E54-DB0E-565D-A5C8-D321CB77045C

Alycaeus sub-digitatus Godwin-Austen, 1876: 177.
Alycaeus (Dicharax) daflaensis var. subdigitata – Kobelt 1902: 368.
Alycaeus (Dicharax) daflaensis var. subdigitatus – Gude 1921: 246.

###### Type locality.

“Shengorh Peak” and “Tánir ridge at 4000 feet”.

###### Material examined.

Shengorh Peak, Dafla Hills, coll. Godwin-Austen, NHMUK 1903.7.1.2498 (3 syntypes).

###### Remarks.

As in the nominotypical subspecies.

##### Dicharax (?) dalingensis

Taxon classificationAnimaliaGastropodaCyclophoridae

(Godwin-Austen, 1914)

950855E5-E957-52F0-A30C-DD1D4F0C51C4

Alycaeus dalingensis Godwin-Austen, 1914: 338–339, pl. 134, figs 3, 3a–c.
Alycaeus (Dicharax) dalingensis – Gude 1921: 246.

###### Type locality.

“Rechila Peak, Daling District, on Sikkim and Bhutan Boundary (10,300 ft.)”.

###### Material examined.

Rechila Pk, Sikkim, leg. W. Robert, NHMUK 1903.7.1.1251 (7 syntypes).

###### Remarks.

Protoconch low, glossy; R1 glossy, no spiral and radial lines visible; R2 short, also glossy, alternating thick/dark and narrow/lighter stripes.

##### Dicharax (?) dohertyi

Taxon classificationAnimaliaGastropodaCyclophoridae

(Godwin-Austen, 1893)

AF222EC3-A712-5E6D-A743-656A80C9CBF4

Alycaeus dohertyi Godwin-Austen, 1893: 595.
Alycaeus dohertyi – Godwin-Austen 1897: 3, pl. 63, figs 3, 3a; Godwin-Austen 1914: 408.
Alycaeus (Dicharax) dohertyi – Kobelt 1902: 369; Gude 1921: 248–249.

###### Type locality.

“Momeit, Burmah”.

###### Remarks.

We could not find the type specimens in the NHM. According to the original description they are in Aldrich’s collection, which is housed in the Michigan Museum (Dance 1986). We contacted the Dr. Taehwan Lee (Michigan Museum) who reported that the type sample of *A. dohertyi* could not be found in the UMMZ. We classify this species in *Dicharax* because the original description did not mention spiral striation, which rules out *Metalycaeus*, and mentions that it has a rather long tube, which rules out *Cycloryx* (= *Pincerna*). The crenulated peristome is characteristic for many *Dicharax* species.

##### Dicharax (?) dolichodeiros

Taxon classificationAnimaliaGastropodaCyclophoridae

(Heude, 1890)

6F7B849E-5579-5A96-B189-8A31A7EA2274

Alycaeus dolichodeiros Heude, 1890: 129, pl. 38, fig. 3.
Alycaeus (Chamalycaeus) dolichodeirus (sic) – Kobelt and Möllendorff 1897: 148.
Alycaeus (Chamalycaeus) dolichodirus (sic) – Kobelt 1902: 355.
Chamalycaeus (Chamalycaeus) dolichodirus (sic) – Zilch 1957: 142.
Dicharax (?) dolichodeiros – Páll-Gergely et al. 2017: 22–23, figs 8D, E, 9E, F, 10E, F.

###### Type locality.

“Tchen k’eou”.

###### Remarks.

No type specimen housed in American museums were reported by Johnson (1973). The non-type specimen figured by Yen (1939: pl. 2, fig. 37), is similar to “*Chamalycaeus*” *helicodes* (= synonym of *Metalycaeus muciferus*, see Páll-Gergely et al. 2017). This species was putatively classified into the genus *Dicharax* by Páll-Gergely et al. (2017).

##### Dicharax (?) duoculmen

Taxon classificationAnimaliaGastropodaCyclophoridae

(Godwin-Austen, 1914)

54C6D55F-C844-5A7C-A126-AF5E793BF587

Alycaeus duoculmen Godwin-Austen, 1914: 365, pl. 157, figs 2, 2a.
Alycaeus (Raptomphalus) duoculmen – Gude 1921: 286–287.
Chamalycaeus (Raptomphalus) duoculmen – Ramakrishna et al. 2010: 68.

###### Type locality.

“Tsanspu Valley”.

###### Material examined.

Tsanspu Valley, leg. Oakes, NHMUK 1903.7.1.3582 (holotype [single specimen mentioned in the original description]).

###### Remarks.

Protoconch low, no spiral lines visible; R1 glossy, with rough wrinkles near the suture and without any spiral lines; R2 very short, with alternating thicker/darker and narrow/lighter stripes; overall surface of R2 smooth.

##### Dicharax (?) duorugosus

Taxon classificationAnimaliaGastropodaCyclophoridae

(Godwin-Austen, 1914)

8B21CF06-5649-5B9B-98DD-5C1DE3D56BAA

Alycaeus duorugosus Godwin-Austen, 1914: 391.
Alycaeus (Dicharax) duorugosus – Gude 1921: 249.
Chamalycaeus (Dicharax) duorugosus – Ramakrishna et al. 2010: 59.

###### Type locality.

“Burrail Range, Naga”, “Also Angaoluo Trigonometrical Station, No. 2572; South Barak, No. 2629, and Munipur, No. 2654 B.M.”.

###### Material examined.

Burrail, coll. Godwin-Austen, NHMUK 1903.7.1.2771 (1 syntype).

###### Remarks.

Protoconch low, no spiral lines visible; R1 glossy, without any notable sculpture; R2 very short, with alternating thicker darker, and narrower lighter stripes; overall surface of the region smooth.

##### Dicharax (?) edei

Taxon classificationAnimaliaGastropodaCyclophoridae

(Godwin-Austen, 1914)

77F774EB-4A4C-563E-99E1-BE566ECA95CB

Alycaeus edei Godwin-Austen, 1914: 391–392, pl. 149, figs 2, 2a.
Alycaeus (Chamalycaeus) edei – Gude 1921: 227.
Chamalycaeus (Chamalycaeus) edei – Ramakrishna et al. 2010: 53.

###### Type locality.

“Naraindhur, Cachar, No. 1665 B.M.”.

###### Material examined.

Naraindhur, Cachar, leg. F. Ede, NHMUK 1903.7.1.1665 (8 syntypes in 2 vials).

###### Remarks.

Protoconch low, glossy, no spiral lines visible; R1 without spiral striation; R2 very long, ribs very slender, relatively sharp, straight; at the edge of the body whorl space between ribs is ca. 3–4 × larger than the ribs themselves.

##### Dicharax (?) ellipticus

Taxon classificationAnimaliaGastropodaCyclophoridae

Páll-Gergely, 2017

97BEF8A6-C8CB-5502-8BA9-EB5E699B0658

Dicharax ellipticus Páll-Gergely in Páll-Gergely et al. 2017: 23–25, figs 15A, B, 16.

###### Type locality.

“Vietnam, Quang Ninh Province, Ha Long Bay area, Tien Ong Cave on Hang Trai Island, collected inside the cave, 20°48.96'N, 107°07.33'E”.

###### Material examined.

RMNH 5004014 (holotype) and some other paratypes, see the original description.

###### Remarks.

Protoconch low, smooth, glossy; R1 also smooth, glossy, R2 also entirely smooth, with alternating narrow light (= microtunnels) and thick dark (= area between the microtunnels) transverse stripes.

##### Dicharax (?) expatriatus

Taxon classificationAnimaliaGastropodaCyclophoridae

(W. T. Blanford & H. F. Blanford, 1860)

AD88A9AC-16F4-597E-B2B1-4930F3936EBC

Alycaeus Expatriatus W. T. Blanford & H. F. Blanford, 1860: 123–124.
Alycaeus expatriatus – Reeve 1878: pl. 5, species 45; Godwin-Austen 1914: 433–434.
Alycaeus (Dicharax) expatriatus – Kobelt 1902: 369–370; Gude 1921: 250–251.
Chamalycaeus (Dicharax) expatriatus – Ramakrishna et al. 2010: 59.
Chamalycaeus expatriatus – Raheem et al. 2014: 46, fig. 25C.
Dicharax (?) expatriatus – Aravind & Páll-Gergely 2018: 59, fig 1B.

###### Type locality.

“Haud raro ad Neddoowuttom ghat, ad latus septentrionale montium “Nilgiri” Indiæ australis et circa 3000–4000 ped. alt.”.

###### Material examined.

Neddiwuttom, Nilgiris, NHMUK 1906.4.4.58 (lectotype, hereby designated and 5 paralectotypes).

###### Remarks.

Protoconch low, glossy, no spiral lines visible; R1 rather glossy, with low, irregular growth ridges and without spiral striae; R2 short with low, widely spaced ribs curved towards aperture.

The lectotype designation of Aravind and Páll-Gergely (2018) was invalid because no explicit statement was made to designate the lectotype. Thus, we here designate that shell to be the lectotype, which was named as such and figured by Aravind & Páll-Gergely (2018: fig. 1B).

##### Dicharax (?) fargesianus

Taxon classificationAnimaliaGastropodaCyclophoridae

(Heude, 1885)

9272FF50-D837-5D88-A271-91060F0E61C7

Alycoeus [sic] fargesianus Heude, 1885: 96, pl. 24, fig. 3, 3a.
Alycaeus (Chamalycaeus) fargesianus – Kobelt and Möllendorff 1897: 148; Kobelt 1902: 355.
Dicharax (?) fargesianus – Páll-Gergely et al. 2017: 26, figs 8B, 10C, D, 17A, B.

###### Type locality.

“in ditione Tchen-k’eou”.

###### Material examined.

China, Tchen-k’eou, MCZ 167229 (4 syntypes). According to Johnson (1973) paratypes are also present in the USNM (inv. number: 472341). These were not examined by us.

###### Remarks.

Protoconch low, rather matte; R1 regularly, densely ribbed; R2 extremely densely ribbed, ribs low, not typical to *Dicharax*.

##### Dicharax (?) footei

Taxon classificationAnimaliaGastropodaCyclophoridae

(W. T. Blanford & H. F. Blanford, 1861)

DFB497E9-31EB-51F4-83F9-6C46EAF949F8

Alycaeus Footei W. T. Blanford & H. F. Blanford, 1861: 348, pl. 1, fig. 3.
Alycaeus footei – Reeve 1878: pl. 4, species 35; Godwin-Austen 1914: 432–433
Alycaeus (Dicharax) footei – Kobelt 1902: 370; Gude 1921: 251–252.
Chamalycaeus (Dicharax) footei – Ramakrishna et al. 2010: 60.
Chamalycaeus footei – Raheem et al. 2014: 46, Fig. 25D.
Dicharax (?) footei – Aravind & Páll-Gergely 2018: 61, figs 1C, D.

###### Type locality.

“Habitat in montibus Kolamulliis dictis”.

###### Material examined.

Kolamalai Hills, nr. Trichinopoly, coll. W.T. Blanford, NHMUK 1906.4.4.57 (lectotype, hereby designated); for additional examined specimens see Aravind & Páll-Gergely (2018).

###### Remarks.

Protoconch low, without spiral structure; R1 smooth, glossy, without spiral striation; R2 long, with straight, low ribs; in some specimens, especially near the beginning of the tube, the ribs look as if they have been “pushed” in an anterior direction (ribs similar to typical *Dicharax*, but lower).

The lectotype designation of Aravind and Páll-Gergely (2018) was invalid, because no explicit statement was made to designate the lectotype. Thus, we here designate that shell to be the lectotype, which was named as such, and figured by Aravind & Páll-Gergely (2018: fig. 1D).

##### Dicharax (?) gemma

Taxon classificationAnimaliaGastropodaCyclophoridae

(Godwin-Austen, 1914)

E2195F0A-241E-5025-884C-6935264FABF3

Alycaeus gemma Godwin-Austen, 1914: 355–356, pl. 149, figs 6, 6a.
Alycaeus (Dicharax) gemma – Gude 1921: 252.
Chamalycaeus (Dicharax) gemma – Ramakrishna et al. 2010: 60; Tripathy et al. 2018: 789.

###### Type locality.

“No .7 Camp, Dikrang Valley, Dafla Hills”.

###### Material examined.

No. 7. camp, Dikrang Valley, Dafla, NHMUK 1903.7.1.2601 (3 syntypes in two vials).

###### Remarks.

All three available shells were weathered, but the following observations could be made: protoconch low, without spiral lines; R1 with rough wrinkles near the suture, but the region is without spiral lines; R2 very short, the fine structure of the ribs could not be observed.

##### Dicharax (?) gemmula

Taxon classificationAnimaliaGastropodaCyclophoridae

 (Benson, 1859)

C894D558-72AD-544E-8432-BA2045ED30ED

Alycaeus gemmula Benson, 1859: 179–180.
Alycaeus gemmula – Reeve 1878: pl. 5, species 37; Godwin-Austen 1886: 190, pl. 48, figs 4, 4a–c.
Alycaeus (Dicharax) gemmula – Kobelt 1902: 370–371; Gude 1921: 252–253.
Alycaeus (Charax) gemmula – Godwin-Austen 1914: 340.
Chamalycaeus (Dicharax) gemmula – Ramakrishna et al. 2010: 60; Tripathy et al. 2018: 789.

###### Type locality.

“in valle Rungun”.

###### Material examined.

Darjling, coll. Blanford, NHMUK 1906.4.4.55 (1 specimen, labelled as “typical”, holotype [single specimen mentioned in the original description]).

###### Remarks.

Protoconch low, glossy, no spiral lines visible; R1 also glossy, R2 moderately long, with thicker darker and very narrow, lighter stripes alternating; overall R2 surface nearly smooth.

##### Dicharax (?) glaber

Taxon classificationAnimaliaGastropodaCyclophoridae

(W. T. Blanford, 1865)

F2786BFE-431E-5837-AAB6-514876F80DF4

Alycaeus glaber W. T. Blanford, 1865: 84.
Alycaeus glaber – Reeve 1878: pl. 4, species 31; Godwin-Austen 1914: 418–419, pl. 151, fig. 1.
Alycaeus (Dicharax) glaber – Kobelt 1902: 371; Gude 1921: 253.

###### Type locality.

“Akyab, Arakan; the hills south of the harbour”.

###### Material examined.

Akyab, coll. W.T. Blanford, NHMUK 1906.4.4.181 (possible syntypes); Akyab, Arakan, NHMUK 20191066 (2 shells).

###### Remarks.

Protoconch low, without spiral striation; R1 without sculpture; R2 long, with regular, low ribs T-shaped in cross section, or curved towards aperture.

##### Dicharax (?) immaculatus

Taxon classificationAnimaliaGastropodaCyclophoridae

Páll-Gergely, 2017

69AE49DC-B310-5263-840B-61A0B70CCC75

Dicharax immaculatus Páll-Gergely in Páll-Gergely et al. 2017: 26–27, figs 8C, 12G, H, 13F, 17C–F.

###### Type locality.

“China, Gansu, Wen Xian, Tielouzangzu Xiang, Sanjiaoyan, 1646 m, 32°53.180'N, 104°24.672'E”.

###### Material examined.

Holotype (HNHM 99707) and a few paratypes, see the original description.

###### Remarks.

Protoconch low, glossy; R1 almost completely smooth, with only very inconspicuous, irregular growth lines; R2 extremely densely ribbed, with low, blunt ribs; for cross-sectional view of R2 see original description.

##### Dicharax (?) ingrami

Taxon classificationAnimaliaGastropodaCyclophoridae

(W. T. Blanford, 1862)

9C897CA8-52F2-54A0-AE55-22255518AAF3

Alycaeus ingrami W. T. Blanford, 1862: 135–136.
Alycaeus ingrami – Reeve 1878: pl. 6, species 54; Godwin-Austen 1886: 193–194, pl. 44, figs 1, 1a–c; Godwin-Austen 1914: 421.
Alycaeus (Chamalycaeus) ingrami – Kobelt 1902: 357; Gude 1921: 228–229.

###### Type locality.

“prope Tongoop in Arakan”.

###### Material examined.

Tongoop, Arakan, coll. Blanford, NHMUK 1906.4.4.68 (7 shells, probable syntypes, figured by Godwin-Austen 1886); Arakan, “authentic”, “type var.”, 1944, No. 450, NHMUK (1 shell).

###### Remarks.

Protoconch low, matte, without spiral striation; R1 with regular, strong, rather dense ribs, near the suture the ribs are sharp, elevated; R2 long, with rather dense, sharp ribs; at the anterior end of R2 the ribs are slightly bent in anterior direction.

##### Dicharax (?) khasiacus

Taxon classificationAnimaliaGastropodaCyclophoridae

(Godwin-Austen, 1871)

2F504B6F-054F-5037-810D-21A4E6D6A887

Alycaeus Khasiacus Godwin-Austen, 1871: 90, pl. 3, fig. 4.
Alycaeus khasiacus – Reeve 1878: pl. 1, species 8; Godwin-Austen 1914: 356, 376–377, 393, pl. 143, figs 7, 7a, 7b.
Alycaeus (Dicharax) khasiacus – Kobelt 1902: 372–373; Gude 1921: 257–258.
Alycaeus khasiacus var. – Godwin-Austen 1914: 356.
Chamalycaeus (Dicharax) khasiacus – Ramakrishna et al. 2010: 62.

###### Type locality.

“On the highest parts of the Khasi and Jiantia Hills”.

###### Material examined.

Lailangkote, Khasi Hills, NHMUK 1903.7.1.2650 (29 syntypes).

###### Remarks.

Protoconch low, glossy, no spiral lines visible; R1 with similar sculpture to that of the protoconch; R2 short, with alternating thicker/darker and narrow/lighter stripes, resulting in a nearly smooth surface.

##### Dicharax (?) kurzianus

Taxon classificationAnimaliaGastropodaCyclophoridae

(Theobald & Stoliczka, 1872)

AACAE32D-DC0F-5612-AE7A-0BD50D83C627

Fig. 20

Alycaeus kurzianus Theobald & Stoliczka, 1872: 330, pl. 11, fig. 2.
Alycaeus kurzianus – Reeve 1878: pl. 3, species 22; Godwin-Austen 1914: 409–410, pl. 151, figs 7, 7a.
Alycaeus (Dicharax) kurzianus – Kobelt 1902: 373; Gude 1921: 258–259.

###### Type locality.

“Nattoung in provincia Barmana, Prome dicta”.

###### Material examined.

Nr. Prome, Pegu, leg. F. Stoliczka, NHMUK 1903.7.1.2700 (3 probable syntypes [labelled as “kurtzianus”; the locality do not match with the original description, but the specimens are identical to the figured one]); Nattoung, W. Prome, coll. Theobald, NZSI M.24974 (1 syntype), 25021 (6 syntypes).

###### Remarks.

Protoconch low, glossy, without spiral lines; R1 also glossy, without any notable sculpture; R2 moderately long, with lamella-like ribs, which are straight in the first half of the region, but are gradually more curved towards the aperture in the second half of R2.

**Figure 20.**
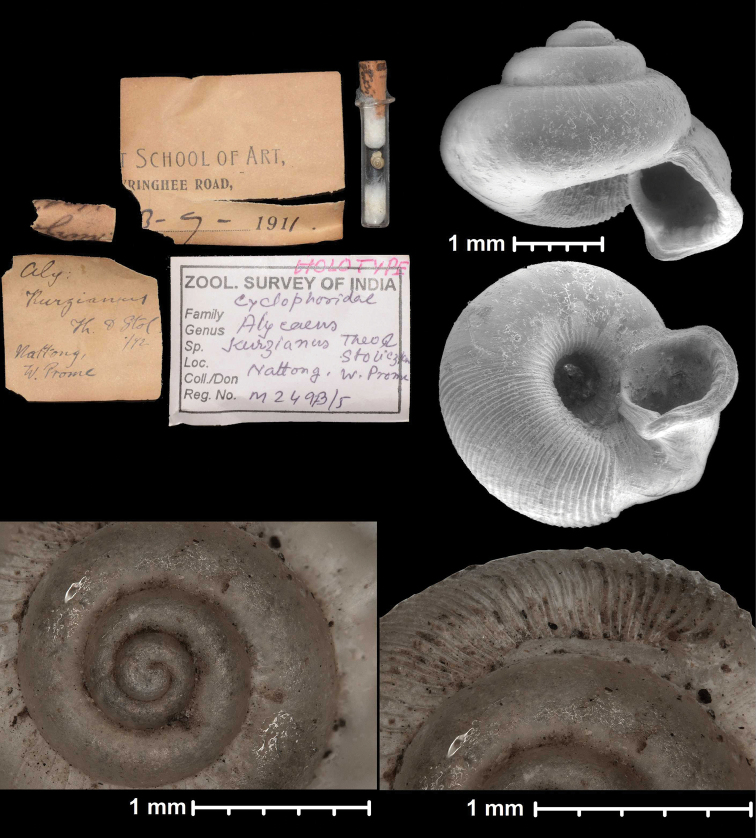
Dicharax (?) kurzianus (Theobald & Stoliczka, 1872) Nattoung, W. Prome, coll. Theobald, syntype (NZSI M.24974). All images: Sheikh Sajan.

##### Dicharax (?) lectus

Taxon classificationAnimaliaGastropodaCyclophoridae

(Godwin-Austen, 1914)

EA66F64F-21A0-5C67-961D-879605E5E556

Fig. 21

Alycaeus lectus Godwin-Austen, 1914: 340, pl. 136, figs 5, 5a, b.
Alycaeus (Dicharax) lectus – Gude 1921: 259.
Chamalycaeus (Dicharax) lectus – Ramakrishna et al. 2010: 62; Tripathy et al. 2018: 789.

###### Type locality.

“in valle Rungun”.

###### Material examined.

Near Chaukkalan, Darjeeling, coll. Dr. F. Stoliczka, NZSI M.8074 (holotype [single specimen mentioned in the original description]).

###### Remarks.

The holotype was strongly weathered, but no spiral striation could be found on the relatively intact protoconch and R1 surface; the protoconch was also low, therefore this species is classified in *Dicharax*. The R2 region was so weathered that its original structure could not be seen.

**Figure 21.**
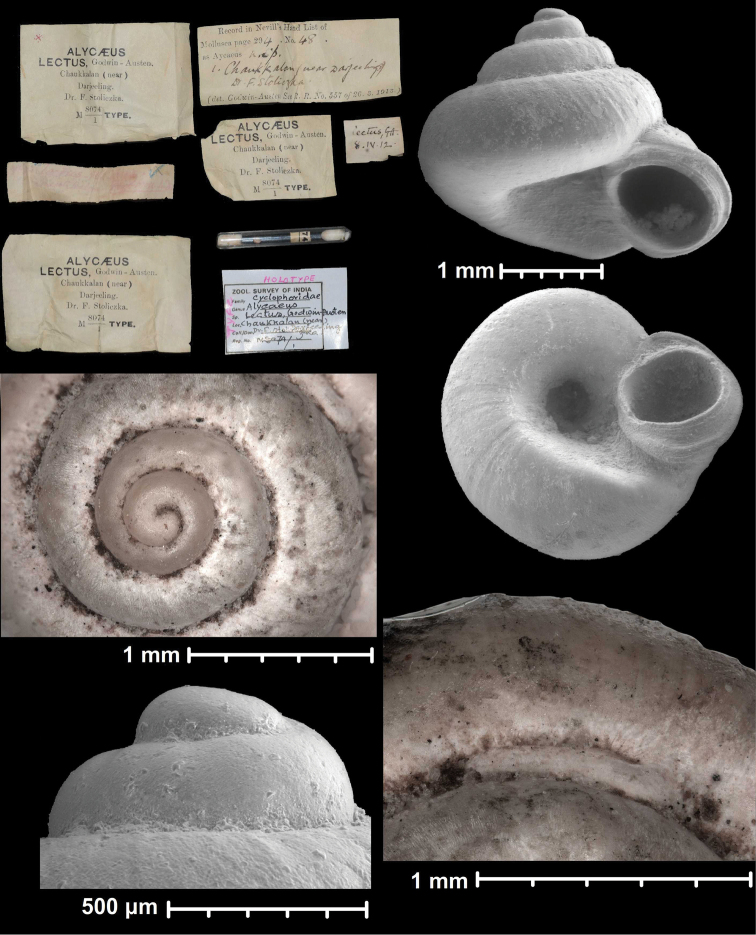
Dicharax (?) lectus (Godwin-Austen, 1914), holotype (NZSI M.8074). All images: Sheikh Sajan.

##### Dicharax (?) lenticulus

Taxon classificationAnimaliaGastropodaCyclophoridae

(Godwin-Austen, 1874)

AFB133FF-A694-549F-A00F-D7817CED0D6D

Fig. 22

Alycaeus lenticulus Godwin-Austen, 1874: 147.
Alycaeus lenticulus – Godwin-Austen 1914: 340–341, pl. 136, figs 2, 2a.
Alycaeus (Dicharax) lenticulus – Gude 1921: 259–260.
Chamalycaeus (Dicharax) lenticulus – Ramakrishna et al. 2010: 63; Tripathy et al. 2018: 789.

###### Type locality.

“Darjeeling”.

###### Material examined.

Darjeeling, coll. Godwin-Austen, NZSI M.8075 (holotype [single specimen mentioned in the original description]).

###### Remarks.

The holotype is strongly corroded. We include this species in *Dicharax* due to the relatively low protoconch.

**Figure 22.**
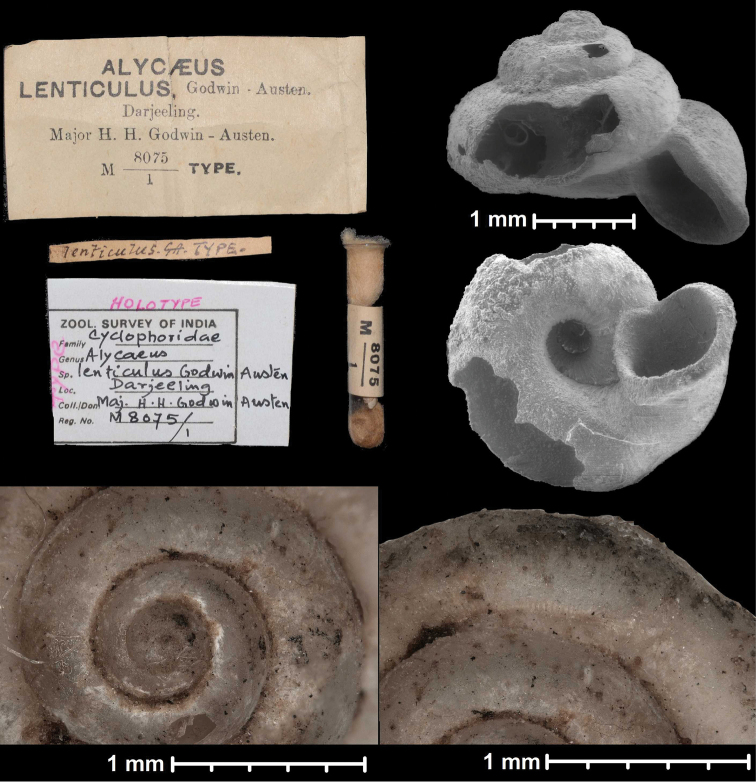
Dicharax (?) lenticulus (Godwin-Austen, 1874), holotype (NZSI M.8075). All images: S. Sajan.

##### Dicharax (?) levis

Taxon classificationAnimaliaGastropodaCyclophoridae

(Godwin-Austen, 1914)

5ABFCD6F-1BAF-586B-A26D-A151E708DC3E

Alycaeus levis Godwin-Austen 1914: 394, pl. 138, figs 3, 3a.
Alycaeus levis – Gude 1921: 209–210.
Alycaeus (Alycaeus) levis – Ramakrishna et al. 2010: 48.

###### Type locality.

“Gaziphimi, Lahupa Naga Hills, Munipur”.

###### Material examined.

Munipur, coll. Godwin-Austen, NHMUK 1903.7.1.2631 (holotype [single specimen mentioned in the original description]).

###### Remarks.

The entire shell was strongly weathered; the fine sculpture could not be fully examined. Protoconch low, no spiral lines visible; R1 without any recognisable sculpture; R2 moderately long, ribs dense and their fine structure could not be examined. The species is placed in the genus *Dicharax* on the basis of its low protoconch.

##### Dicharax (?) logtakensis

Taxon classificationAnimaliaGastropodaCyclophoridae

(Godwin-Austen, 1914)

83FA6444-7421-559F-9623-96ABF3E44197

Alycaeus logtakensis Godwin-Austen, 1914: 394–395, pl. 155, fig. 6.
Alycaeus logtakensis – Gude 1921: 209–210.
Alycaeus (Alycaeus) logtakensis – Ramakrishna et al. 2010: 48.

###### Type locality.

“Logtak Lake, Munipur (...) on a low hill near the northern shore”.

###### Material examined.

On low hill. Logtak Lake, Munipur, coll. Godwin-Austen, “figured”, NHMUK 1903.7.1.2639 (1 syntype).

###### Remarks.

Protoconch elevated, but no spiral lines were visible; R1 with widely spaced ribs without spiral striae; R2 relatively long with widely spaced, sharp ribs.

##### Dicharax (?) magnus

Taxon classificationAnimaliaGastropodaCyclophoridae

(Godwin-Austen, 1893)

AAB9F50F-CDD0-5CC6-8372-A642B9667861

Alycaeus magnus Godwin-Austen, 1893: 594.
Alycaeus (Alycaeus) magnus – Kobelt 1902: 346–347; Ramakrishna et al. 2010: 49.
Alycaeus magnus – Godwin-Austen 1914: 395, pl. 138, figs 1, 1a; Gude 1921: 210–211, fig. 33.

###### Type locality.

“Naga Hills, 150 miles eastward of Kohima”.

###### Material examined.

Naga Hills, fr. Beddome, NHMUK 1903.7.1.1480 (1 syntype).

###### Remarks.

Protoconch low, weathered, no spiral lines visible; R1 also without spiral lines; R2 very long (similar to *Chamalycaeus heudei*), some of the ribs have a horizontal projection in an anterior direction; near the suture these projections sometimes reach the neighbouring ribs; the entire shell is somewhat weathered, therefore it is not possible to decide if all ribs had these horizontal projections or only some of them.

##### Dicharax (?) moellendorffi

Taxon classificationAnimaliaGastropodaCyclophoridae

(Kobelt & Möllendorff, 1887)

4D04347F-2E33-507D-BA1E-B6C1C75366D2

Alycaeus inflatus Möllendorff, 1886: 168, pl. 5, fig. 7.
Alycaeus (Chamalycaeus) moellendorffi Kobelt & Möllendorff, 1897: 149; Kobelt 1902: 359.
Chamalycaeus moellendorffi – Yen 1939: 30, pl. 2, fig. 35.
Chamalycaeus (Chamalycaeus) moellendorffi – Zilch 1957: 144, pl. 5, fig. 12.
Dicharax (?) moellendorffi – Páll-Gergely et al. 2017: 27–30, fig. 18.

###### Type locality.

“Dau-dshou provinciae sinensis Hunan”.

###### Material examined.

China: Dau-dshou (Hunan), coll. Möllendorff 1886, coll. Boettger, SMF 39236 (lectotype, designated by Yen 1939); same data, SMF 39237 (2 paralectotypes); same data, SMF 39238 (8 paralectotypes).

###### Remarks.

Protoconch low, glossy, without spiral striation; R1 with dense, rather regular, low ribs and without spiral striation; R2 relatively long, with darker wider and lighter narrower stripes alternating, the overall surface of R2 is smooth.

##### Dicharax (?) montanus

Taxon classificationAnimaliaGastropodaCyclophoridae

(Nevill, 1881)

6F46A0C0-0617-5EE4-8FB6-1E7210B99C24

Fig. 23

Alycaeus montanus Nevill, 1881: 149, pl. 6, fig. 5.
Alycaeus (Chamalycaeus) montanus – Kobelt 1902: 359; Gude 1921: 229.
Alycaeus montanus – Godwin-Austen 1914: 341–342, pl. 136, figs 3, 3a.
Chamalycaeus (Chamalycaeus) montanus – Ramakrishna et al. 2010: 54.

###### Type locality.

“Sikkim, at 11,000 ft”.

###### Material examined.

Sikkim, at 11,000 ft, coll. Dr. Stoliczka, NZSI M.8082 (syntype, labelled as holotype).

###### Remarks.

The original description does not mention the number of available specimens. Thus, the shell labelled as holotype is considered to be a syntype.

Protoconch low, without spiral striation, R1 with low, rather regular ribbing; surface of R2 wavy, ribs are blunt and only slightly elevated from the surface.

**Figure 23.**
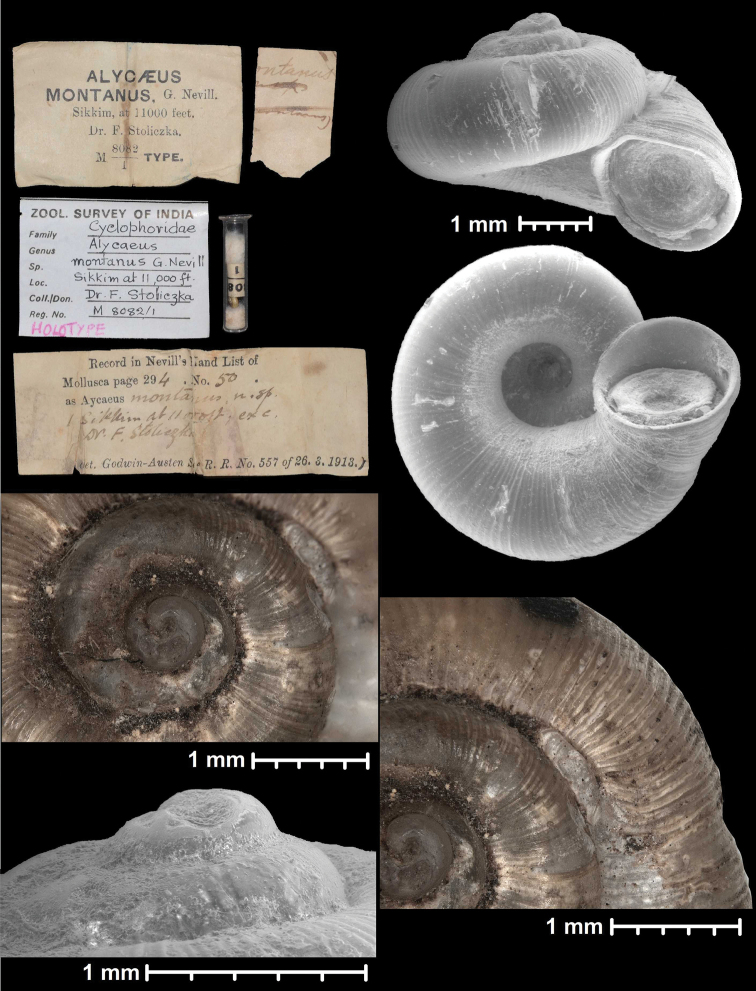
Dicharax (?) montanus (Nevill, 1881), syntype (NZSI M.8082). All images: Sheikh Sajan.

##### Dicharax (?) multirugosus

Taxon classificationAnimaliaGastropodaCyclophoridae

(Godwin-Austen, 1874)

1592380C-7F20-5DED-B94B-EDF558D79C97

Alycaeus multirugosus Godwin-Austen 1874: 149, pl. 3, fig. 7.
Alycaeus (Dicharax) multirugosus – Kobelt 1902: 373; Gude 1921: 260.
Alycaeus multirugosus – Godwin-Austen 1914: 395–396, pl. 144, figs 7, 7a.
Chamalycaeus (Dicharax) multirugosus – Ramakrishna et al. 2010: 63.

###### Type locality.

“Hills at head of the Lanier River, Naga Hills, ca. 5–6,000 feet”.

###### Material examined.

Head of Lanier R.N.E. Munipur, coll. Godwin-Austen, NHMUK 1903.7.1.2485 (2 syntypes).

###### Remarks.

Protoconch low, rather glossy, no spiral lines visible; R1 smooth, without spiral striation, only some irregular, rough wrinkles visible near the suture; R2 moderately long, nearly smooth with alternating darker and lighter stripes.

##### Dicharax (?) muspratti

Taxon classificationAnimaliaGastropodaCyclophoridae

(Godwin-Austen, 1914)

7E19637D-907B-5501-9871-466EAFE94ABC

Alycaeus muspratti Godwin-Austen, 1914: 396, pl. 148, fig. 1.
Alycaeus (Raptomphalus) muspratti – Gude 1921: 289.
Chamalycaeus (Raptomphalus) muspratti – Ramakrishna et al. 2010: 69.

###### Type locality.

“Eastern Naga”.

###### Material examined.

E. Naga, coll. R.H. Beddome, NHMUK 1912.4.16.273 (16 syntypes).

###### Remarks.

Protoconch low, no spiral lines visible; R1 with very widely spaced regular ribs and without spiral striation; R2 long, some ribs slightly curved towards the aperture, but mostly straight, lamella-like.

##### Dicharax (?) mutatus

Taxon classificationAnimaliaGastropodaCyclophoridae

(Godwin-Austen, 1876)

3A94D8CB-DD0E-511D-B0CB-AD59D566DA91

Alycaeus mutatus Godwin-Austen, 1876: 177–178, pl. 7, figs 11, 11a.
Alycaeus (Dicharax) mutatus – Kobelt 1902: 373–374; Gude 1921: 260–261.
Alycaeus mutatus – Godwin-Austen 1914: 357, pl. 145, figs 9, 9a.
Chamalycaeus (Dicharax) mutatus – Ramakrishna et al. 2010: 63; Tripathy et al. 2018: 789.

###### Type locality.

“On Torúpútú, Tánir, and Shengorh Peaks, at 6–7000 feet elevation, in the dead leaves and moss about the roots of the forest”.

###### Material examined.

Toruputu Peak, Dafla Hills, coll. Godwin-Austen, NHMUK 1903.7.1.2495 (9 syntypes).

###### Remarks.

Protoconch low, finely granulated, no spiral lines visible; R1 rather regularly, finely ribbed without spiral lines; R2 relatively short, with narrow, greyish, and somewhat more thickened yellowish corneous alternating stripes; entire R2 surface almost smooth, but narrow greyish stripes slightly elevated from the surface.

##### Dicharax (?) nagaensis

Taxon classificationAnimaliaGastropodaCyclophoridae

(Godwin-Austen, 1871)

2C390697-188D-57CA-BD21-709D8EB99753

Alycaeus ingrami var. nagaensis Godwin-Austen, 1871: 92, pl. 5, fig. 2.
Alycaeus nagaensis – Godwin-Austen 1884: pl. 51, figs 3, 7; Godwin-Austen 1886: 195, pl. 44, figs 3, 3a–c; Godwin-Austen 1914: 396–397, pl. 143, figs 2, 2a, 2b.
Alycaeus (Chamalycaeus) nagaensis – Kobelt 1902: 359; Gude 1921: 230.
Chamalycaeus (Chamalycaeus) nagaensis – Ramakrishna et al. 2010: 54.

###### Type locality.

“Neighbourhood of Asálú, rather local in its distribution, but abundant”.

###### Material examined.

Asalu, N. Cachar, NHMUK 1903.7.1.2615 (7 syntypes in 2 vials).

###### Remarks.

Protoconch rather low without any signs of spiral striae; R1 regularly ribbed without spiral striation; R2 long with widely spaced, sharp ribs (typical for *Metalycaeus*).

##### Dicharax (?) nattoungensis

Taxon classificationAnimaliaGastropodaCyclophoridae

(Godwin-Austen, 1914)

161A199C-16A6-5DB7-ADF9-77667BA94B72

Fig. 24

Alycaeus nattoungensis Godwin-Austen, 1914: 410–411, pl. 155, figs 15, 15a.
Alycaeus nattoungensis – Gude 1921: 212–213.

###### Type locality.

“Nattoung Hills”.

###### Material examined.

Nattoung Hills, Mendon District, Pegu, Burma, coll. Theobald, NZSI M.8036 (holotype, [single specimen mentioned in the original description]).

###### Remarks.

Protoconch low, without spiral striation; beginning of R1 nearly smooth, its end with rather widely spaced, wide, but low ribs; R2 long, with rather sharp, straight ribs.

**Figure 24.**
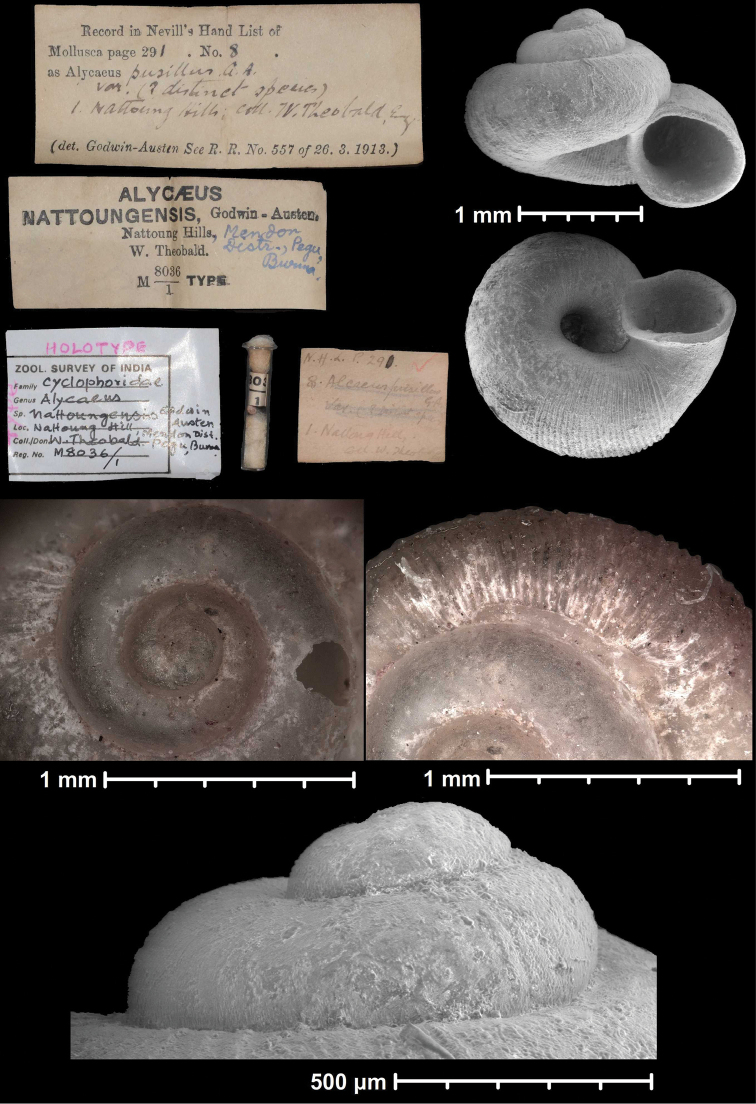
Dicharax (?) nattoungensis (Godwin-Austen, 1914), holotype (NZSI M.8036). All images: Sheikh Sajan.

##### Dicharax (?) nongtungensis

Taxon classificationAnimaliaGastropodaCyclophoridae

(Godwin-Austen, 1914)

E262B596-9814-5166-AD2E-02B6700225C0

Alycaeus nongtungensis Godwin-Austen, 1914: 378, pl. 138, figs 5, 5a.
Alycaeus (Dicharax) nongtungensis – Gude 1921: 261.
Chamalycaeus (Dicharax) nongtungensis – Ramakrishna et al. 2010: 64.

###### Type locality.

“Nongtung, Jaintia Hills”.

###### Material examined.

Nongtung, coll. Godwin-Austen, NHMUK 1903.7.1.2692 (21 syntypes).

###### Remarks.

Protoconch low, without spiral striation; R1 glossy, without any sculpture; R2 short, with low ribs, not bent in any direction.

##### Dicharax (?) obscurus

Taxon classificationAnimaliaGastropodaCyclophoridae

(Godwin-Austen, 1914)

0B935850-05D3-5F79-8547-DE50FA58661B

Alycaeus obscurus Godwin-Austen, 1914: 378–379, pl. 154, figs 9, 9a.
Alycaeus (Dicharax) obscurus – Gude 1921: 263.
Chamalycaeus (Dicharax) obscurus – Ramakrishna et al. 2010: 64.

###### Type locality.

“Cherra Poonje”.

###### Material examined.

Cherra Poonje, leg. Godwin-Austen, NHMUK 1913.3.16.8 (1 syntype).

###### Remarks.

Protoconch slightly elevated, but no spiral lines visible; R1 also without spiral lines; R2 moderately long with relatively dense but spaced, sharp ribs. The slightly elevated protoconch and the morphology of the ribs is similar to that of *Chamalycaeus*, but they are closer to each other than usually occurs in that genus.

##### Dicharax (?) omissus

Taxon classificationAnimaliaGastropodaCyclophoridae

(Godwin-Austen, 1914)

93A0C83C-4AA4-518B-8C03-0261BAE315C2

Alycaeus omissus Godwin-Austen, 1914: 411, pl. 155, fig. 13.
Alycaeus (Chamalycaeus) omissus – Gude 1921: 231.

###### Type locality.

“Siam and Shan boundary”.

###### Material examined.

Siam & Shan boundary, coll. Woodthorpe, NHMUK 1903.7.1.1228 (2 syntypes).

###### Remarks.

Protoconch low, no spiral lines visible; R1 also without spiral striation; R2 short, with dense ribs; the fine morphology of the ribs could not be examined due to corrosion.

##### Dicharax (?) pachitaensis

Taxon classificationAnimaliaGastropodaCyclophoridae

(Godwin-Austen, 1886)

5C18DCD1-5ABC-5389-8161-55C44D4B7751

Alycaeus pachitaensis Godwin-Austen, 1886: 190–191, pl. 48, figs 5, 5a–c.
Alycaeus (Dicharax) pachitaensis – Kobelt 1902: 374; Gude 1921: 264.
Alycaeus pachitaensis – Godwin-Austen 1914: 359.
Chamalycaeus (Dicharax) pachitaensis – Ramakrishna et al. 2010: 65; Tripathy et al. 2018: 789.

###### Type locality.

“Pachita village (Camp no. 7 of the Expeditionary Force, 1874–75), Dafla Hills, Assam”.

###### Material examined.

Pachita village, Dafla Hills, coll. Godwin-Austen, NHMUK 1903.7.1.2614 (3 syntypes).

###### Remarks.

Protoconch low, without spiral striae; R1 also without spiral striation; R2 short, with very low ribs; this region is characterised by alternating thicker/dark, and narrower/white stripes.

##### Dicharax (?) panshiensis

Taxon classificationAnimaliaGastropodaCyclophoridae

(Chen, 1989)

D3B436FA-2774-58BA-B830-5E0743865EE5

Chamalycaeus panshiensis Chen, 1989: 157.
“*Chamalycaeus*” panshiensis – Páll-Gergely et al. 2017: 107.

###### Type locality.

“Hongn-an forestry centre, Panshi County (43°15'N, 126°10'E), Jilin Province, China”.

###### Material examined.

Type specimen presumably lost.

###### Remarks.

The type specimens could not be found in Beijing during a recent search, and they are thus considered lost. The original description is not sufficient for correct generic placement. A *Dicharax* species have been collected near the type locality of *C. panshiensis* (Guoyi Zhang pers. comm.) reminiscent of Korean and Japanese *Dicharax* species, which suggests that *C. panshiensis* belongs to the genus *Dicharax*. However, since no specimens are available and the original description is also useless, collection of topotypic specimens would be necessary.

##### Dicharax (?) peilei

Taxon classificationAnimaliaGastropodaCyclophoridae

(Preston, 1914)

DC9CF92D-FA5A-5DB4-B416-B7FE990FE58C

Alycaeus (Charax) peilei Preston, 1914: 22–23, figure on page 23.
Alycaeus peilei – Godwin-Austen 1914: 397–398.
Alycaeus (Dicharax) peilei – Gude 1921: 264.
Chamalycaeus (Dicharax) peilei – Ramakrishna et al. 2010: 65.

###### Type locality.

“Naga Hills”.

###### Material examined.

Naga Hills, coll. Preston, NHMUK 1915.1.4.1281 (1 syntype).

###### Remarks.

The syntype is strongly weathered, the original sculpture could not be examined. *Alycaeus peilei* is placed in the genus *Dicharax* on the basis of the seemingly low protoconch.

##### Dicharax (?) plectocheilus

Taxon classificationAnimaliaGastropodaCyclophoridae

(Benson, 1859)

F9F34B4C-702C-5ADD-8050-D79D8B738105

Alycaeus plectocheilus Benson, 1859: 180.
Alycaeus plectocheilus – Reeve 1878: pl. 2, species 14; Godwin-Austen 1914: 342–343, pl. 134, figs 4, 4a–c.
Alycaeus (Dicharax) plectochilus [sic] – Kobelt 1902: 375.
Alycaeus plectocheilus , large var. – Godwin-Austen 1914: 342–343, pl. 133, figs 3, 3a–c.
Alycaeus (Dicharax) plectochilus [sic] – Gude 1921: 264–265.
Chamalycaeus (Dicharax) plectochilus – Ramakrishna et al. 2010: 65.
Dicharax plectocheilus – Páll-Gergely et al. 2017: 54.

###### Type locality.

“in valle Rungun”.

###### Material examined.

Darjiling, Rungun Valley, coll. Blanford, NHMUK 1906.4.4.184 (2 syntypes); Vorder Indien, Rungun Valley, Darjiling, leg. Hungerford 1889, leg. O. Boettger, SMF 109254 (2 shells); Damsang, Daling District, NHMUK 1903.7.1.1256 (25 shells of “large var.”).

###### Remarks.

Protoconch low, rather glossy, no spiral lines visible; R1 glossy, very finely ribbed without spiral lines; R2 moderately long, it forms a nearly smooth area with alternating thicker/darker and narrower/lighter stripes.

##### Dicharax (?) pusillus

Taxon classificationAnimaliaGastropodaCyclophoridae

(Godwin-Austen, 1871)

12AF3161-0162-5C2A-9D46-E9AFD384243F

Alycaeus pusillus Godwin-Austen, 1871: 89–90, pl. 3, fig. 3.
Alycaeus pusillus – Reeve 1878: pl. 1, species 7; Godwin-Austen 1914: 379–380, 398, pl. 143, figs 6, 6a, 6b; Gude 1921: 215–216.
Alycaeus (Alycaeus) pusillus – Kobelt 1902: 348; Ramakrishna et al. 2010: 50.

###### Type locality.

“near Jawai”, “on the banks of the Kopili river on the road from Jawai to Asálú, viâ Súfai”.

###### Material examined.

Jawai, Jaintia, NHMUK 1903.7.1.2688 (5 syntypes).

###### Remarks.

Protoconch low, no spiral lines visible; R1 glossy, without notable sculpture; R2 short, with alternating thicker/darker and narrower/lighter stripes; the overall surface of R2 is seemingly smooth.

##### Dicharax (?) rechilaensis

Taxon classificationAnimaliaGastropodaCyclophoridae

(Godwin-Austen, 1914)

5584F81C-7530-59EB-BEAC-AA5E20846CF1

Alycaeus rechilaensis Godwin-Austen, 1914: 343–344, pl. 134, figs 2, 2a.
Alycaeus (Dicharax) rechilaensis – Gude 1921: 267.

###### Type locality.

“Rechila Peak, Daling District, on Sikhim-Bhutan Frontier, 10,300 feet”.

###### Material examined.

Rechila Pk., Sikkim, leg. N. Robert, NHMUK 1903.7.1.1252 (holotype [single specimen mentioned in the original description]).

###### Remarks.

Protoconch low, without spiral striae; R1 also without spiral striae and with low, dense, regular ribs; R2 with low, regular ribs; the entire R2 surface is wavy.

##### Dicharax (?) sandowayensis

Taxon classificationAnimaliaGastropodaCyclophoridae

(Godwin-Austen, 1914)

54715EB4-52F9-5825-B070-EF7A027E086A

Alycaeus sandowayensis Godwin-Austen, 1914: 423–424, pl. 139, figs 4, 4a.
Alycaeus (Chamalycaeus) sandowayensis – Gude 1921: 232.

###### Type locality.

“Mai-i, Sandoway District, Arakan”.

###### Material examined.

Mai-i, Sandoway Dist., Arakan, leg. Stoliczka, NHMUK 1903.7.1.2558 (holotype [single specimen mentioned in the original description]).

###### Remarks.

Protoconch moderately elevated, no signs of spiral striae; R1 regularly ribbed, without spiral striae; R2 very short, ribs lamella-like, straight, seemingly do not differ from the ribs of R1.

##### Dicharax (?) sculpturus

Taxon classificationAnimaliaGastropodaCyclophoridae

(Godwin-Austen, 1875)

8D1DA002-11B8-5077-AFCD-2B9E5AD85659

Alycaeus sculpturus Godwin-Austen 1875: 8, pl. 4, fig. 2.
Alycaeus (Alycaeus) sculpturus – Kobelt 1902: 351; Ramakrishna et al. 2010: 51.
Alycaeus sculpturus – Godwin-Austen 1914: 398–399, pl. 145, figs 6, 6a, 6b; Gude 1921: 218.

###### Type locality.

“on the hill ranges from near Tellizo Peak to the eastward, and on Mungching Hill in Munipur”.

###### Material examined.

Sikhami, N.E. Munipur, NHMUK 1903.7.1.2666 (1 syntype); Mungching, Munipur, NHMUK 1903.7.1.2667 (4 syntypes).

###### Remarks.

The specimen in lot 1903.7.1.2666 is weathered, so the original sculpture could not be fully examined, but a second type lot of *A. sculpturus* contained four shells in relatively good condition (NHMUK 1903.7.1.2667).

Protoconch moderately elevated, no spiral lines visible; R1 with widely spaced, strong ribs and without spiral lines; R2 of normal length, with widely spaced, sharp ribs.

##### Dicharax (?) serratus

Taxon classificationAnimaliaGastropodaCyclophoridae

(Godwin-Austen, 1874)

F1B7E306-CDEF-59D0-AD28-3409FA6471B7

Alycaeus serratus Godwin-Austen, 1874: 148–149, pl. 3, fig. 6.
Alycaeus (Alycaeus) serratus – Kobelt 1902: 351; Ramakrishna et al. 2010: 51.
Alycaeus serratus – Godwin-Austen 1914: 400, pl. 144, figs 6, 6a, 6b; Gude 1921: 219.

###### Type locality.

“Laisen Trigl. station, Munipur Hills”.

###### Material examined.

Laisen Valley, Jiantia Hills, NHMUK 1903.7.1.2487 (1 syntype).

###### Remarks.

The syntype was strongly corroded. Protoconch low without spiral lines; R1 smooth with rough wrinkles near the suture; R2 moderately long, the fine structure of the ribs could not be examined because the ribs have been damaged.

##### Dicharax (?) sonlaensis

Taxon classificationAnimaliaGastropodaCyclophoridae

(Raheem & Schneider, 2017)

A8C73A58-C20D-570A-95DF-2151642E4E91

Alycaeus sonlaensis Raheem & Schneider, 2018: 1305, figs 4A–J.

###### Type locality.

“The earliest Miocene (Aquitanian, 23–21 Ma) Hang Mon Formation at Hang Mon in Northern Vietnam”.

###### Remarks.

This species fits the range of morphological variation of *Dicharax fimbriatus*, which is an extant species from the same geographic area. However, we find it more useful to keep this taxon as a separate species due to its age. The strikingly similar appearance of an early Miocene species (Aquitanian, 23–21 Ma) to species living today indicates the high level of morphological stability of *Dicharax*.

##### Dicharax (?) stoliczkii

Taxon classificationAnimaliaGastropodaCyclophoridae

(Godwin-Austen, 1874)

F6A26778-EEE1-51C5-BA78-8AEE3EE8617D

Alycaeus Stoliczkii Godwin-Austen, 1874: 147, pl. 3, fig. 3.
Alycaeus stoliczkii – Reeve 1878: pl. 6, species 53; Godwin-Austen 1914: 399–400, pl. 144, figs 3, 3a, 3b.
Alycaeus (Chamalycaeus) stoliczkai [sic] – Kobelt 1902: 363; Gude 1921: 233–234.
Chamalycaeus (Chamalycaeus) stoliczkai [sic] – Ramakrishna et al. 2010: 55.

###### Type locality.

“Angaoluo Peak, Nágá Hills at 7,000 feet”; “further to the east at Kezakenomih, and at the head of the Lanier River at ca. 5,000 feet where the specimens are much larger”.

###### Material examined.

Naga Hills, NHMUK 1903.7.1.2622 (3 syntypes).

###### Remarks.

Protoconch low, without spiral structure; R1 rather regularly ribbed without spiral structure, R2 long, with sharp, straight, low ribs. The sharp ribs are characteristic for the genus *Dicharax*, but in case of *A. stoliczkii* the ribs are much lower than in any *Chamalycaeus* species. Therefore, based on the low protoconch without spiral lines, we classify this species in the genus *Dicharax*.

##### Dicharax (?) strangulatus

Taxon classificationAnimaliaGastropodaCyclophoridae

(L. Pfeiffer, 1846)

D234939D-4459-5FFB-B52C-21E5213B63FE

Cyclostoma strangulatum L. Pfeiffer, 1846: 86.
Alycaeus strangulatus – Reeve 1878: pl. 6, species 47; Godwin-Austen 1914: 337, pl. 136, figs 1, 1a.
Alycaeus (Dicharax) strangulatus – Kobelt 1902: 376; Gude 1921: 269.
Chamalycaeus (Dicharax) strangulatus – Ramakrishna et al. 2010: 66.
Dicharax strangulatus – Sajan et al. 2020: 523, Figs 1A, B, 2A–L.

###### Type locality.

“Bengalia”.

###### Material examined.

“possible syntype” NHMUK 1856.9.15.18 (1 shell); Mussoorie, N.W Himalaya,”figd”, NHMUK 1903.7.1.2501 (at least 5 shells); NHMUK 1928.7.28.85–104 (from general collection). See also newly collected specimens examined in Sajan et al. (2020).

###### Remarks.

Protoconch low, without spiral striae; R1 with irregular, low ribs and without spiral striation; R2 short, with alternating wider/darker and narrower/lighter stripes; entire surface nearly smooth.

##### Dicharax (?) strigatus

Taxon classificationAnimaliaGastropodaCyclophoridae

(Godwin-Austen, 1874)

8068CA56-A35E-5105-86DE-6FFAA7484979

Alycaeus strigatus Godwin-Austen, 1874: 146–147, pl. 3, fig. 2.
Alycaeus (Chamalycaeus) strigatus – Kobelt 1902: 363; Gude 1921: 234–235.
Alycaeus strigatus – Godwin-Austen 1914, 381, 401, pl. 144, figs 2, 2a, 2b.
Chamalycaeus (Chamalycaeus) strigatus – Ramakrishna et al. 2010: 56.

###### Type locality.

“Assam”.

###### Material examined.

Type specimen presumably lost.

###### Remarks.

The syntypes should be in the Indian Museum (see Nevill 1878), but they have not been located there. This species is tentatively classified in the genus *Dicharax* based on the flat shell and low protoconch, and its resemblance to Dicharax (?) khasiacus (see the original description).

##### Dicharax (?) subculmen

Taxon classificationAnimaliaGastropodaCyclophoridae

(Godwin-Austen, 1893)

019233E1-A2D1-58A7-9974-774A4A249A50

Alycaeus subculmen Godwin-Austen, 1893: 593.
Alycaeus subculmen – Godwin-Austen 1897: 4, pl. 63, figs 4, 4a.
Alycaeus (Dicharax) subculmen – Kobelt 1902: 377; Gude 1921: 270–271.
Alycaeus subculmen – Godwin-Austen 1914: 398.
Chamalycaeus (Dicharax) subculmen – Ramakrishna et al. 2010: 67.

###### Type locality.

“Naga Hills”.

###### Material examined.

Naga Hills, leg. Doherty, NHMUK 1903.7.1.2687 (3 syntypes).

###### Remarks.

Protoconch low, no spiral lines visible; R1 glossy, without spiral striae; R2 short; ribs slightly bent, although the exact fine sculpture could not be determined due to corrosion of the shell.

##### Dicharax (?) subhumilis

Taxon classificationAnimaliaGastropodaCyclophoridae

(Möllendorff, 1897)

108C48F8-868C-563F-A3F7-C01991A76401

Fig. 25

Alycaeus (Charax) subhumilis Möllendorff, 1897a: 41.
Alycaeus (Dicharax) subhumilis – Kobelt 1902: 377; Gude 1921: 271, pl. 1, figs 1, 2.
Alycaeus (Charax) subhumilis – Godwin-Austen 1914: 344–345, pl. 133, figs 2, 2a–c.
Chamalycaeus (Dicharax) subhumilis – Zilch 1957: 146, pl. 6, fig. 24; Ramakrishna et al. 2010: 67.

###### Type locality.

“in montibus Darjiling Indiae”.

###### Material examined.

Vorderindien: Darjiling, coll. Möllendorff, SMF 109224 (lectotype, designated by Zilch 1957); Same data, SMF 109225 (paralectotype).

###### Remarks.

Protoconch low, without spiral striae; R1 glossy, without spiral lines; R2 moderately long, with alternating wider/darker and narrower/lighter stripes; overall surface smooth.

**Figure 25.**
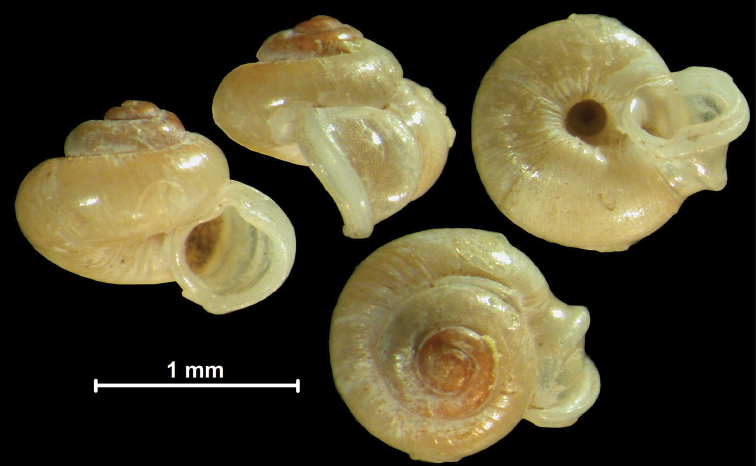
*Dicharax subhumilis* (Möllendorff, 1897), lectotype (SMF 109224). Photographs: Barna Páll-Gergely, courtesy Ronald Janssen.

##### Dicharax (?) succineus

Taxon classificationAnimaliaGastropodaCyclophoridae

(W. T. Blanford, 1862)

110EB0D8-B041-5244-8D56-11509E37CF40

Alycaeus succineus W. T. Blanford, 1862: 139–140.
Alycaeus succineus – Reeve 1878: pl. 2, species 16; Godwin-Austen 1914: 424, pl. 151, fig. 2.
Alycaeus (Dicharax) succineus – Kobelt 1902: 377; Gude 1921: 271–272.

###### Type locality.

“in montibus Arakanensibus”.

###### Material examined.

Tangoop Pass, Arakan Hills, NHMUK 1906.404.52 (1 syntype).

###### Remarks.

Protoconch rather low, without spiral striae; R1 regularly ribbed without spiral striation; R2 long with widely spaced, sharp ribs. *Alycaeus succineus* is classified in *Dicharax* on the basis of the low protoconch and the absence of spiral striation, but the elevated, sharp R2 ribs are characteristic of the genera *Metalycaeus* and *Chamalycaeus*.

##### Dicharax (?) umbonalis

Taxon classificationAnimaliaGastropodaCyclophoridae

(Benson, 1856)

EEB0F0E7-14DE-5345-8932-0ED0B2ABD0B8

Alycaeus umbonalis Benson, 1856: 225–226.
Alycaeus umbonalis – Reeve 1878: pl. 4, species 36; Godwin-Austen 1886: 194–195, pl. 44, figs 2, 2a–c; Godwin-Austen 1914: 413, 424.
Alycaeus (Chamalycaeus) umbonalis – Kobelt 1902: 364; Gude 1921: 235–236.

###### Type locality.

“ad Akaouktong, prope ripas fluvii Irawadi”.

###### Material examined.

Burmah, UMZC I.103005 (3 syntypes); Akoutong, Burma, Blanford coll., NHMUK (5 shells).

###### Remarks.

Protoconch low, no spiral lines visible; R1 with rather regular ribs and without spiral striation; R2 long, with widely spaced, ribs lamella-like, straight, but relatively low (typical *Chamalycaeus* character).

##### Dicharax (?) woodthorpi

Taxon classificationAnimaliaGastropodaCyclophoridae

(Godwin-Austen, 1914)

14CF94A4-EF00-5691-AED4-D968CB946364

Alycaeus woodthorpi Godwin-Austen, 1914: 414, pl. 155, fig. 14.
Alycaeus (Dicharax) woodthorpei [sic] – Gude 1921: 275.

###### Type locality.

“Fort Stedman, Burma”.

###### Material examined.

Fort Stedman, Burma, coll. Woodthorpe, NHMUK 1903.7.1.3064 (22 syntypes in two vials); Burma, E. R. Sykes Collection, Acc. no. 1825, NHMUK 20150126 (2 shells).

###### Remarks.

Protoconch glossy, low, no spiral lines visible; R1 with rather irregular, dense, low ribs without spiral striation; R2 with alternating thicker/darker and narrow/lighter stripes, the overall surface is smooth.

#### Japanese and Korean *Dicharax*

##### Dicharax (?) abei

Taxon classificationAnimaliaGastropodaCyclophoridae

(Kuroda, 1951)

1DE1573B-6048-5196-9808-89D63B1075EF

Fig. 13B

Awalycaeus abei Kuroda, 1951: 73–74, text figs 1–3.
Awalycaeus abei – Kuroda and Abe 1980: 20; Minato 1982a: 121–123, fig. 4; Azuma 1982: 13, pl. 4, fig. 44; Hanshin Shell Club 1986: pl. 2, figs 7–9; Minato 1988: 17; Yano et al. 2016: 57, fig. 4C-a, b.

###### Type locality.

“徳島縣（阿讃境に近く）城壬山々頂附近” (Tokushima, [near the Sakai boundary], near the top of Mt. Jiyauwau).

###### Material examined.

Kito-son, Taira, Tokushima Pref., leg. Y. Koyama, 27.03.1975, NSMT-Mo 50125 (8 shells).

###### Remarks.

Holotype is deposited in the NC-H006 (Kazunori Hasegawa, pers. comm. 2015), and was not examined by us.

Protoconch low, rather matte, very finely granulated; R2 with 30–32 low, blunt, irregular ribs, which are usually in contact with each as they near the tube; at the edge of the body whorl they are distinct, the gap between them being slightly smaller than the width of a rib; no spiral lines visible on R1.

##### Dicharax (?) akioi

Taxon classificationAnimaliaGastropodaCyclophoridae

(Kuroda & Abe, 1980)

7804D59F-A957-5C6F-BA52-AF8595C624E8

Cipangocharax akioi Kuroda & Abe, 1980: 20, pl. 2, figs 5–7.
Cipangocharax akioi Minato and Abe 1982: 200–202, fig. 9; Azuma 1982: 13, pl. 4, fig. 42; Minato 1988: 16; Minato 1993: 1, fig. 5.

###### Type locality.

“高越山” (Koutsu-san = Mt. Kotsu, Yoshinogawa-shi, Tokushima Prefecture, Shikoku Island).

###### Material examined.

徳島縣1山川町高越山 (Tokushima Prefecture, Yamakawa Town, Mt. Koutsu), Sakurai collection, NSMT-Mo 79090 (2 shells); Holotype: NC-H005 (Kazunori Hasegawa, pers. comm. 2015, not examined).

###### Remarks.

Protoconch low, very finely granulated, moderately matte; R2 with ca. 26 blunt, relatively low ribs; near the suture most ribs form connections (projections) with their neighbours or are completely merged; at the edge of the body whorl the space between the ribs is slightly less than the width of the ribs; no spiral lines visible on R1.

##### Dicharax (?) akiratadai

Taxon classificationAnimaliaGastropodaCyclophoridae

(Minato, 1982)

93163604-D0C6-5FB7-A36F-306906DD5FDE

Awalycaeus akiratadai Minato, 1982a: 121–123, figs 1–3.
Awalycaeus akiratadai – Minato 1988: 17, pl. 3, figs 5, 6; Yano et al. 2016: 57, figs 4B-a–d.

###### Type locality.

“Koogejima, Sekizen-mura, Ochi-gun, Ehime-Ken”.

###### Material examined.

NSMT-Mo 60073 (holotype).

###### Remarks.

Protoconch low, very finely granulated, only slightly glossy; R2 with ca. 23 relatively low riblets occasionally fused to each other near the tube; space between the riblets at the edge of the body whorl is roughly as wide as the ribs; no spiral lines visible on R1.

##### Dicharax (?) ananensis

Taxon classificationAnimaliaGastropodaCyclophoridae

(Yano, Tada & Matsuda, 2013)

747D6B03-F703-558C-9235-BAB2E865E8C8

Cipangocharax ananensis Yano, Tada & Matsuda, 2013: 29–37, figs 1–4.

###### Type locality.

“Wakasugidani, Suii-cho, Anan city, Tokushima Prefecture, Shikoku, Japan”.

###### Remarks.

No specimens were examined by us, but the original description provides enough information of the key characters: protoconch low, glossy; R1 with rather dense, strong ribs; R2 short, with very dense, low ribs; R3 longer than R2, without swelling.

##### Dicharax (?) biexcisus

Taxon classificationAnimaliaGastropodaCyclophoridae

(Pilsbry, 1902)

F65D5868-FE7E-5435-8D0D-18D7DDCCDC14

Fig. 13C

Alycaeus biexcisus Pilsbry, 1902d: 26.
Awalycaeus biexcisus – Kuroda and Abe 1980: 20, pl. 2, figs 1–4.
Cipangocharax biexcisus – Minato 1981: 135–137, fig. 4; Minato and Abe 1982: 200–202, figs 10–12; Azuma 1982: 12, pl. 4, fig. 41; Minato 1988: 16, pl. 2, fig. 5; Minato 1993: 1, figs 2, 4.
Chamalycaeus (Cipangocharax) biexcisus – Egorov 2013: 36, fig. 64.

###### Type locality.

“Suimura, Awa”.

###### Material examined.

ANSP 82660a (lectotype, designated by Baker 1964, photographs examined), Nagai-mura, Awa, Tokushima Pref., Japan, coll. Hirase (#263), NSMT-Mo 2050 (3 shells).

###### Remarks.

Protoconch low, finely granulated, rather matte; R2 with ca. 26 low, blunt, irregular ribs, which are connected to each other near the tube, gradually becoming free towards the edge of the body whorl (here the distance between neighbouring ribs is very small, much less than the width of a rib); no spiral lines visible on R1.

##### Dicharax (?) cyclophoroides

Taxon classificationAnimaliaGastropodaCyclophoridae

(Pilsbry & Y. Hirase, 1909)

1DB7B20D-F763-5FC7-96D5-DD1B4CB87A76

Alycaeus cyclophoroides Pilsbry & Y. Hirase, 1909b: 9, pl. 5, figs 1, 2.
Alycaeus cyclophoroides – Pilsbry 1926: 454.
Chamalycaeus (Metalycaeus) cyclophoroides – Kuroda 1936: 170.

###### Type locality.

“Fusan, Korea”.

###### Examined specimens.

ANSP 95741 (1 syntype, photographs examined); 韓国江原道雪岳山 (Kankoku, Kougen-do, Soraku-san (Japanese reading = Republic of Korea, Gangwon Province, Seoraksan, Mt. Seorak), Sakurai coll. NSMT-Mo 79091 (4 shells).

###### Remarks.

Protoconch low, finely granulated, rather matte; R2 with ca. 28 low, blunt riblets, which form connections with each other near the tube; they are distinct at the edge of the body whorl, the space between them is slightly smaller than the width of a rib; no spiral lines visible on R1.

##### Dicharax (?) cyclophoroides koshuensis

Taxon classificationAnimaliaGastropodaCyclophoridae

(Kuroda, 1936)

452F5130-914B-5170-8A2F-305C565D7A4B

Chamalycaeus (Metalycaeus) cyclophoroides koshuensis Kuroda, 1936: 174.
Chamalycaeus cyclophoroides koshuensis – Hanshin Shell Club 1986: pl. 2, figs 3, 4.

###### Type locality.

“全羅南道光州” (Zenranan-do, Kwanju/Koshu = Jeonlanam Province, Gwangju).

###### Material examined.

朝鮮光州 (Chōsen [= Korea], Kāshū), Kawamura coll., NSMT-Mo (4 shells).

###### Remarks.

According to Kwon and Habe (1979)*Chamalycaeus koshuensis* Kuroda, 1936 is a synonym (“local form”) of *Alycaeus cyclophoroides*. The holotype is deposited in the NC (NC-H203) (not examined by us).

Protoconch low or slightly elevated; R2 with ca. 26, relatively elevated and distinct ribs which are not in contact even at near the tube; at the edge of the body whorl the distance between the ribs is more than twice as wide as the width of a rib; there are no spiral lines visible on R1.

##### Dicharax (?) expanstoma

Taxon classificationAnimaliaGastropodaCyclophoridae

(Minato, 1982)

6B395E6D-2EA1-5547-85EB-D54BA6E3064D

Chamalycaeus expanstoma Minato, 1982b: 126–127, pl. 1, figs 1–4.
Chamalycaeus expanstoma – Minato 1988: 15, pl. 3, figs 1, 2.

###### Type locality.

“Iejima, Ujigunto Islets, Kagoshima-ken, Japan”

###### Material examined.

NSMT-Mo 59699 (holotype); 鹿児島縣1宇治群島家島 (Kagoshima-ken, Uji-gunto, Iejima = Kagoshima Prefecture, Uji Archipelago, Iejima), coll. Sakurai, NSMT-Mo 79092 (2 shells).

###### Remarks.

Protoconch low, very finely granulated, moderately glossy, no spiral lines visible; R2 with ca. 14–20 (14 Sakurai coll., 20 on the holotype) low, blunt ribs; ribbing resembles irregular wrinkled surface; near the tube some ribs reach the neighbouring ones, and partly fuse with them; at the edge of the body whorl the ribs are not in contact, the spaces between ribs almost reaches the width of a single rib; no spiral lines on R1.

##### Dicharax (?) itonis

Taxon classificationAnimaliaGastropodaCyclophoridae

(Kuroda, 1943)

A61811EF-C767-5FDD-86CC-DA73256414AD

Fig. 13D

Chamalycaeus (Sigmacharax) itonis Kuroda, 1943: 8–11, figs 1–4.
Chamalycaeus (Sigmacharax) itonis itonis – Minato 1987b: 76–77, figs 1–3; Minato 1988: 16, pl. 2, fig. 2.
Chamalycaeus (Sigmacharax) itonis – Azuma 1982: 12, pl. 4, fig. 40; Hanshin Shell Club 1986: pl. 2, figs 5, 6.

###### Type locality.

“Kami-iti-mura, Bittyū, Okayama Prefecture”.

###### Material examined.

Ura-Hikimi-Kyo, Hikimi-mach, Mino-gun, Shimane Pref., NSMT-Mo 78866 (5 shells). The holotype is deposited in NC-H004 (Kazunori Hasegawa, pers. comm.) (not examined by us).

###### Remarks.

Protoconch low, very finely granulated, moderately glossy; R2 with ca. 24 ribs; they are low and the entire ribbing is not clear; the ribs are not distinct entities, their boundaries are obscure since their edges merge; no spiral lines visible on R1.

##### Dicharax (?) itonis shiotai

Taxon classificationAnimaliaGastropodaCyclophoridae

(Minato & Yano, 1988)

54EFFF25-0F51-536B-837C-D63D2975EF62

Chamalycaeus (Sigmacharax) itonis shiotai Minato & Yano, 1988: 33–36, figs 1–3.

###### Type locality.

“Kasayama, Hagi city, Yamaguchi-ken, Japan”

###### Material examined.

NSMT-Mo 64484 (holotype).

###### Remarks.

Protoconch low, very finely granulated, not glossy; however, the protoconch of the holotype was quite weathered; ca. 16 ribs on R2 (individual ribs are wider than in the nominotypical subspecies); R2 ribbing similar as in the nominotypical subspecies; no spiral lines visible on R1.

##### Dicharax (?) japonicus

Taxon classificationAnimaliaGastropodaCyclophoridae

(E. von Martens, 1865)

65147953-27F4-55EF-A0F4-115CF8197965

Alycaeus japonicus E. von Martens, 1865: 51.
Alycaeus japonicus – Pilsbry 1900: 381.
Alycaeus harimensis Pilsbry, 1900: 381.
Alycaeus reinhardti – Pilsbry 1900: 381.
Alycaeus (Chamalycaeus) harimensis – Kobelt 1902: 356.
Alycaeus (Chamalycaeus) japonicus – Kobelt 1902: 357.
Alycaeus (Chamalycaeus) pilsbryi Kobelt 1902: 361. (nom. nov. pro Alycaeus reinhardti Pilsbry, 1900, non Mörch, 1872)
Chamalycaeus harimensis – Azuma 1982: 10, pl. 3, fig. 29.
Chamalycaeus pilsbryi – Azuma 1982: 11, pl. 4, fig. 36.
Chamalycaeus japonicus japonicus – Minato 1988: 14.
Chamalycaeus japonicus – Yano 2015: 233–236, fig. 2.

###### Type locality.

“Yokohama”.

###### Material examined.

ANSP 78777a (lectotype of *A. harimensis*, designated by Baker 1964, photographs examined); ANSP 78817a (lectotype of *A. reinhardti*, designated by Baker 1964, photographs examined); 紀伊周参見 (Kii Susami), Toru Inaba collection, NSMT-Mo 66474 (5 shells) (“*japonicus harimensis*”).

###### Remarks.

Protoconch low, glossy; R2 with 18–22 low, blunt ribs connected to each other near the tube, but free at the edge of the body whorl (here the distance between two ribs is ca. as wide as the width of a single rib); no spiral lines visible on the teleoconch.

##### Dicharax (?) japonicus sadoensis

Taxon classificationAnimaliaGastropodaCyclophoridae

(Pilsbry & Y. Hirase, 1903)

358BBE74-2906-5097-96DD-CCEC427394B1

Alycaeus harimensis var. sadoensis Pilsbry & Y. Hirase, 1903: 128–130.
Chamalycaeus japonicus sadoensis – Minato 1988: 14.

###### Type locality.

“Aikawa, Sado”.

###### Material examined.

ANSP 83895a (lectotype, designated by Baker 1964, photographs examined); 佐渡長江川左岸北斜面 標高200 m ナラ トチ クリ 雑木林 1 個体 1982-11 矢田政治 (Sado, Nagae-gawa, sa-gan, kita-shamen, hyoko 200 m, nara, tochi, kuri zoki-bayashi, 1 kotai, 1982-11, Yada Masaji = Sado, Nagae river, left bank, north slope, altitude 200 m, mixed forest of oak [nara], Japanese horse-chestnut [tochi], and Japanese chestnut [kuri], 1 specimen, Nov. 1982, Masaji Yada), NSMT-Mo 60464 (1 shell).

###### Remarks.

The shell we examined was weathered, only the shape of protoconch (low) could be seen and its sculpture was not visible; R2 with ca. 22 low, blunt, irregular ribs which are joined to their neighbours at least near the tube; no spiral lines visible on the shell.

##### Dicharax (?) kiuchii

Taxon classificationAnimaliaGastropodaCyclophoridae

(Minato & Abe, 1982)

377B98EF-27AB-5DED-8872-A180FEF459D6

Cipangocharax kiuchii Minato & Abe, 1982: 200–202, figs 1–8.
Cipangocharax kiuchii – Azuma 1982: 13; Minato 1988: 17; Minato 1993: 1, figs 1, 7.

###### Type locality.

“Near Toogen cave, Mt. Hizuka, Takano, Kisawa-son, Naga-gun, Tokushima-ken, Japan”.

###### Material examined.

NSMT-Mo 59545 (holotype); 徳島縣1那賀郡木沢村高野 (Tokushima-ken, Naka-gun, Kisawa-son, Takano = Tokushima Prefecture, Naka County, Kisawa Village, Takano), Sakurai coll., NSMT-Mo 79093 (3 shells).

###### Remarks.

Protoconch similar to that of *biexcisus*; R2 with 16, widely spaced, low, blunt ribs; spaces as wide as or slightly wider (at the edge of the body whorl) than individual ribs; in the case of the three shells in the Sakurai collection some of the ribs on R2 are joined to the neighbouring ones near the tube; no spiral lines visible on R1. Operculum relatively slim with low outer belt and without nipple (see also original description).

##### Dicharax (?) kurodatokubeii

Taxon classificationAnimaliaGastropodaCyclophoridae

(Minato, 1987)

C767DC04-BDDB-5B0B-A323-B23955AEA667

Chamalycaeus kurodatokubeii Minato, 1987a: 222–223, 224–225, figs 1–3.
Chamalycaeus kurodatokubeii – Minato 1988: 15.

###### Type locality.

“Ichihashi (limestone region), Ikeda-cho, Ibi-gun, Gifu-ken, Japan”

###### Material examined.

NSMT-Mo 64217 (holotype); 岐阜縣1揖斐郡池田町 (Gifu-ken, Ibi-gun, Ikeda-cho = Gifu Prefecture, Ibi County, Ikeda Town), coll. Sakurai, NSMT-Mo 79094 (2 shells).

###### Remarks.

The protoconch of the holotype probably has a growth disorder, and its sculpture could not be fully examined. It was low, very finely granulated, and the last 0.25 of the whorl was wrinkled. The specimens in the Sakurai collection have normally developed protoconchs, which were low and finely granulated, without spiral lines; R2 has ca. 30–32 low, blunt ribs; most ribs are completely merged with the neighbouring ribs nearer the tube; ribs free at edge of body whorl; space between ribs ca. 2 × as wide as an individual rib; no spiral lines visible on R1.

##### Dicharax (?) miyazakii

Taxon classificationAnimaliaGastropodaCyclophoridae

(Takahashi & Habe, 1976)

BAC4E2AA-72FB-5315-9EAE-E6CDD233AC04

Chamalycaeus miyazakii Takahashi & Habe, 1976: 27–28, text fig. 1.
Chamalycaeus miyazakii – Minato 1988: 15, pl. 3, figs 7, 8.

###### Type locality.

“Inunaki-yama, Kasuya-gun, Fukuoka Pref., Kyushu”.

###### Material examined.

福岡縣1粕屋郡古賀町薬王寺 (Fukuoka-ken, Kasuya-gun, Koga-machi, Yakuoji = Fukuoka Prefecture, Kasuya County, Koga Town, Yakuoji [=temple], coll. Sakurai, NSMT-Mo 79095 (13 shells).

###### Remarks.

Protoconch low, very finely granulated, but rather glossy; R2 with ca. 20 low, blunt riblets; they are very low near the tube (it is difficult to decide whether they overlap or not); at the edge of the body whorl the space between the ribs is roughly as wide as a single rib; no spiral lines visible on R1; operculum relatively thick, at least as thick as that of *C. kiuchii*.

##### Dicharax (?) nakashimai

Taxon classificationAnimaliaGastropodaCyclophoridae

(Minato, 1987)

1FF8A5B6-9F38-59B0-BC74-C2A58AF46BE7

Chamalycaeus (Sigmacharax) itonis nakashimai Minato, 1987b: 75–77, figs 4–6.
Chamalycaeus (Sigmacharax) nakashimai nakashimai – Minato and Yano 2000: 133, figs 5–7.
Chamalycaeus (Sigmacharax) itonis nakashimai – Minato 1988: 16.

###### Type locality.

“Namariyama, Misasa-cho, Tōhaku-gun, Tottori-ken, Japan”.

###### Material examined.

NSMT-Mo 64355 (holotype).

###### Remarks.

Protoconch low, very finely granulated, moderately glossy (the protoconch of the holotype was partly weathered); R2 with ca. 20 low ribs, clearly separated at edge of body whorl; generally ribs more distinct than in *S. itonis itonis*; here space between ribs is as wide as individual ribs; no visible space between the ribs closer to the tube, except for those situated close to the end of the tube; no spiral lines visible on R1.

##### Dicharax (?) nakashimai ditaceus

Taxon classificationAnimaliaGastropodaCyclophoridae

(Minato & Yano, 2000)

9674D3CF-6575-5644-BA2E-69F50055FFDE

Chamalycaeus (Sigmacharax) nakashimai ditaceus Minato & Yano, 2000: 129–133, figs 1–4.

###### Type locality.

“Ochiiwa (35°24'N, 134°22'E), Kooge-cho, Yazu-gun, Tottori Prefecture, Japan”.

###### Material examined.

NSMT-Mo 71684 (holotype).

###### Remarks.

Protoconch low, relatively glossy, finely granulated with some fine radial growth lines; R2 with ca. 26 low riblets, more distinct at edge of body whorl, but overall very obscure; no spiral lines visible on R1.

##### Dicharax (?) nishii

Taxon classificationAnimaliaGastropodaCyclophoridae

(Minato, 2005)

C9791A29-CDD1-5329-A62B-863FE4DC0101

Chamalycaeus nishii Minato, 2005: 41–44, figs 4–6.

###### Type locality.

“Tsugenotaki-do Cave, (limestone region), 32°38'N, 131°18'E, Kuronitsa, Takachiho-cho, Nishisugi-gun, Miyazaki Prefecture, Japan”.

###### Material examined.

NSMT-Mo 73685 (holotype); NSMT-Mo 73686 & 73687 (paratypes).

###### Remarks.

Protoconch low, moderately glossy, very finely granulated, with fine wrinkles on the last 0.25 of whorl; R2 with ca. 28 low, hardly separable, blunt ribs; close to the tube boundary between ribs hardly visible, they possibly overlap; at the edge of the body whorl there is some distance between ribs, smaller than width of an individual rib; no spiral lines visible on R1.

##### Dicharax (?) okamurai

Taxon classificationAnimaliaGastropodaCyclophoridae

(Azuma, 1980)

1B1AC8FE-DE7A-5198-A8AC-98D7A067148E

Awalycaeus okamurai Azuma, 1980: 140–141, figs 1–5.
Cipangocharax okamurai – Azuma 1982: 13, pl. 4, fig. 43; Minato 1988: 17; Minato 1993: 1, fig. 3.

###### Type locality.

“Mt. Sarumasa (altitude about 500–600 meters), Nitachȏ, Nitagun, Shimane Pref., Japan”.

###### Material examined.

島根縣1猿政山 (Shimane-ken, Sarumasayama = Shimane Prefecture, Mt. Sarumasa), coll. Sakurai, NSMT-Mo 79096 (7 shells); Japan, W-Honshu, Shimane-pref., Mt. Sarumasa-yama, ex coll. M. Azuma 1983, SMF 256326 (2 shells).

###### Remarks.

Protoconch low, relatively glossy, extremely finely granulated; R2 with ca. 24 low, blunt, irregular ribs, which usually overlap with the neighbouring rings nearer the tube; distance between ribs at edge of body whorl ca. half rib width; no spiral lines visible on R1.

This species has a lip that is strongly extended in the direction of the umbilicus. Also, the thickened operculum is very peculiar with a compressed hourglass shape.

##### Dicharax (?) okinawaensis

Taxon classificationAnimaliaGastropodaCyclophoridae

(Uozumi, Yamamoto & Habe, 1979)

2ED22BB9-5E2D-5F8A-BA2D-3B3477004C7B

Chamalycaeus okinawaensis Uozumi, Yamamoto & Habe, 1979: 167–168.
Chamalycaeus okinawaensis – Minato 1988: 12.

###### Type locality.

“Sueyoshi-gu-ato, Naha City, Okinawa Main Island”

###### Remarks.

This species was originally described as a fossil species. However, a living individual was collected on Mount Nekumachiji (26°41'01.18"N, 128°08'17.85"E), Okinawa Island (Hiroshi Fukuda, pers. comm. 2016).

We have not found the holotype in the NSMT-Mo (inventory number 57766 according to the original description). We place the species in this genus because the original description does not mention spiral lines.

##### Dicharax (?) oshimanus

Taxon classificationAnimaliaGastropodaCyclophoridae

(Pilsbry & Y. Hirase, 1904)

B3C1D4BD-F4DA-5F08-AE85-5D2D5A1E15F9

Alycaeus oshimanus Pilsbry & Y. Hirase, 1904b: 7–8.
Chamalycaeus oshimanus – Azuma 1982: 9, pl. 3, fig. 26; Minato 1988: 13.

###### Type locality.

“Oshima, Osumi”.

###### Material examined.

ANSP 83385a (lectotype, designated by Baker 1964, photographs examined); 鹿児島縣1奄美大島 (Kagoshima-ken, Amami Oshima = Kagoshima Prefecture, Amami Oshima), Sakurai coll., NSMT-Mo 79097 (2 shells).

###### Remarks.

Protoconch low, finely granulated, moderately glossy; R2 with ca. 32 low, blunt ribs; in one of the specimens the ribs are free from each other even near the tube; distance between ribs at edge of body whorl larger than a rib width; in the other, weathered specimens (Sakurai coll.) the regular ribs stand close to each other, similarly to that of *C. laevis*; no spiral lines on the teleoconch.

##### Dicharax (?) placenovitas

Taxon classificationAnimaliaGastropodaCyclophoridae

(Minato, 1981)

4FCD98A4-355D-50A1-914C-5C31B6BE8064

Cipangocharax placenovitas Minato, 1981: 135–137, figs 1–3.
Cipangocharax placenovitas – Azuma 1982: 12–13; Minato 1988: 17, pl. 2, figs 3, 4; Minato 1993: 1, fig. 6.

###### Type locality.

“Arakura (Limestone region), Haruno-cho, Agawa-gun, Kochi-ken, Japan”.

###### Material examined.

NSMT-Mo 58918 (holotype).

###### Remarks.

Protoconch low, smooth, but the last 0.5 whorl of the protoconch was slightly weathered; ca. 24 ribs on R2 , well separated and wide near beginning of tube, gradually becoming narrower, and dense towards end of tube; near tube ribs situated very close to each other, some of them overlap with their neighbours; ribs clearly separated at edge of body whorl, but spaces still much narrower than a rib width; no spiral lines visible on R1.

Operculum with a big central nipple on its inner side; however, the outer belt was absent. The columellar margin of the aperture is not sinuated. The aperture forms a connection between the *Cipangocharax*-type and normal-type aperture.

##### Dicharax (?) purus

Taxon classificationAnimaliaGastropodaCyclophoridae

(Pilsbry & Y. Hirase, 1904)

D3169C07-8B0E-5436-8D79-0EBCA7E0A9EF

Alycaeus purus Pilsbry & Y. Hirase, 1904c: 617.
Chamalycaeus purus – Minato 1988: 12.

###### Type locality.

“Tokunoshima, Ōsumi”.

###### Material examined.

ANSP 87683 (1 syntype, photographs examined).

###### Remarks.

Protoconch low, moderately glossy; R1 with low riblets, R2 with denser, blunt ribs, which are connected to each other near the tube.

##### Dicharax (?) shiibaensis

Taxon classificationAnimaliaGastropodaCyclophoridae

(Minato, 2005)

69129F5E-60C9-56E3-BA4C-4084222F8232

Chamalycaeus shiibaensis Minato, 2005: 39–41, figs 1–3.

###### Type locality.

“Entrance of the Matsukiinari-no-ana Cave, (limestone region), 32°30'N, 131°80'E, Shiiba-son, Higashiusuki-gun, Miyazaki Prefecture, Japan”.

###### Material examined.

NSMT-Mo 73682 (holotype), NSMT-Mo 73683 & 73684 (paratypes).

###### Remarks.

Protoconch low, very finely granulated, moderately glossy, no wrinkles present; R2 with ca. 22 low, blunt, but well separated ribs; near tube some ribs merge with neighbouring ribs, but distance between ribs at edge of body whorl ca. as wide as a single rib (or slightly wider); no spiral lines visible on R1.

##### Dicharax (?) shiosakimasahiroi

Taxon classificationAnimaliaGastropodaCyclophoridae

(Yano, Matsuda & Nishi, 2016)

E1A51944-62A1-54C9-8183-22C520CFD4F4

Awalycaeus shiosakimasahiroi Yano, Matsuda & Nishi in Yano et al. 2016: 55–57, figs 2A–G, 3C, D, 4B-a–B-d.

###### Type locality.

“Mt. Konoha, Gyokuto-machi, Tamana-gun, Kumamoto Prefecture, Japan”.

###### Remarks.

No specimens were examined by us, but the photographs of the original description provide sufficient details of the fine sculpture. Protoconch low, rather glossy; R1 with widely spaced, elevated, sharp ribs; R2 with much denser, blunter ribs.

##### Dicharax (?) spiracellum

Taxon classificationAnimaliaGastropodaCyclophoridae

(A. Adams & Reeve, 1850)

58E0D59A-9287-5A08-A1BD-EDD87A6F5353

Cyclostoma spiracellum A. Adams & Reeve, 1850: 56, pl. 14, fig. 1.
Alycaeus spiracellum – E. von Martens 1867: 150–151; Reeve 1878: pl. 4, species 33; Godwin-Austen 1889: 346, pl. 37, fig. 6.
Alycaeus (Dicharax) spiracellum – Kobelt 1902: 376.
Alycaeus kurodai Pilsbry & Y. Hirase 1908: 60, pl. 4, figs 1–4.
Alycaeus kurodae (sic) – Pilsbry 1926: 454.
Chamalycaeus (Metalycaeus) kurodai – Kwon and Habe 1979: 26.
Dicharax spiracellum Páll-Gergely 2019: 192, fig 1A–D. (kurodai Pilsbry & Y. Hirase, 1908 is a synonym)

###### Type locality.

“Borneo, under decayed vegetable matter in the forests” (*spiracellum*), “Cheju (Quelpart) Island” (*kurodai*). Páll-Gergely (2019) emended the type locality of *C. spiracellum* to “probably Cheju Island, South Korea”. See Remarks.

###### Material examined.

Borneo, ‘Samarang pl. 14. F. 1’ (in pencil), coll. Mrs Lombe Taylor, NHMUK 1874.12.11.233 (possible syntype); Borneo, NHMUK 1889.12.7.27 (1 shell); 済州島 (Seishūtō = Jeju Island), coll. Hirase (#275) NSMT-Mo 7593 (4 shells, labelled as *A. kurodai*); ANSP 95742 (1 syntype of *A. kurodai*) (photographs examined).

###### Remarks.

protoconch low, rather matte, with irregular fine wrinkles; R2 with ca. 24 low ribs; boundaries between ribs hardly visible; ribs situated very close to each other near tube; distance between ribs at edge of body whorl roughly a rib width; no spiral lines visible on R1.

*Dicharax spiracellum* (A. Adams & Reeve, 1850) was described from Borneo, based on shells from the expedition of HMS Samarang (1843–1846). *Alycaeus kurodai* Pilsbry & Y. Hirase, 1908, described from the Korean Cheju Island, is conchologically identical to *Dicharax spiracellum* and is a junior synonym of the former species (Páll-Gergely 2019). The type specimens of *Dicharax spiracellum* were probably obtained when the Samarang visited Cheju Island, and the specimens were most likely mislabelled (Páll-Gergely 2019). Therefore, the type locality of *D. spiracellum* was emended to “probably Cheju Island, South Korea” (Páll-Gergely 2019).

##### Dicharax (?) spiracellum duplicatus

Taxon classificationAnimaliaGastropodaCyclophoridae

(Kuroda & Miyanaga, 1943)

A97FFB05-4517-5452-A3D4-8C682F92CAD6

Chamalycaeus kurodai duplicatus Kuroda & Miyanaga, 1943: 130, 136, text fig. 4.
Chamalycaeus (Metalycaeus) kurodai – Kwon and Habe 1979: 26 (treats duplicatus as a synonym = “local form”).
Dicharax spiracellum duplicatus – Páll-Gergely 2019: 192.

###### Type locality.

“Reisui, Zenra-Nandō, southern coast of Tyōsen”.

###### Remarks.

No specimens of this taxon were examined by us. The holotype is deposited in the Nishinomiya Museum (NC-H204; Kazunori Hasegawa, pers. comm. 2015). The drawing in the original description shows a shell with two parallel R3 swellings, which justifies the distinction of that form at least at subspecies level.

##### Dicharax (?) tadai

Taxon classificationAnimaliaGastropodaCyclophoridae

(Kuroda & Kawamoto, 1956)

3192F2BF-C4F3-579C-B002-DD6DED91C3DB

Chamalycaeus tadai Kuroda & Kawamoto in Kawamoto & Tanabe, 1956: 13, 86, figs 11, 12.
Chamalycaeus tadai – Azuma 1982: 11–12, pl. 4, fig. 39; Hanshin Shell Club 1986: pl. 2, figs 1, 2; Minato 1988: 15.

###### Type locality.

“萩市見島, 日崎” (Hisaki, Mishima, Hagishi [Hagi City in Yamaguchi Prefecture]).

###### Material examined.

山口縣1見島日崎 (Yamaguchi-ken, Mishima, Hizaki = Yamaguchi Prefecture, Mishima, Hizaki), Sakurai collection, NSMT-Mo 79098 (7 shells).

###### Remarks.

Protoconch low, very finely granulated; moderately glossy; R2 with 18–24 irregular, low, blunt ribs, connected to each other near tube and sometimes further away from tube (near edge of body whorl) also; distance between ribs at edge of body whorl usually ca. a rib width; no spiral lines visible on R1.

The authors (Kuroda and Kawamoto 1956) wrote the following: “Having lamellar plate in the form of spiral plates on the outer surface is a feature of the *Metalycaeus* group” (translated by J.U. Otani).

##### Dicharax (?) takahashii

Taxon classificationAnimaliaGastropodaCyclophoridae

(Habe, 1976)

569B38FC-C248-597E-B6D9-C33DE8C19991

Chamalycaeus takahashii Habe, 1976: 225, figs 4, 5.
Chamalycaeus takahashii Minato 2005: 42–43, figs 7, 8; Azuma 1982: 10, pl. 3, fig. 33; Minato 1988: 15.

###### Type locality.

“Onagara-dȏ Cave, Honjȏ-mura, Minamimabe-gun, Ȏita Pref., Kyushu”.

###### Material examined.

NSMT-Mo 52661 (holotype), NSMT-Mo 52662 (paratype).

###### Remarks.

Protoconch low, finely granulated, moderately glossy; R2 with 32–36 low, blunt, well separated ribs; nearer the tube there is some distance between most of the ribs, but some of them join with their neighbours; distance between ribs at edge of body whorl 1–2 × as large as a rib width; no spiral lines visible on R1.

##### Dicharax (?) takahashii muroharai

Taxon classificationAnimaliaGastropodaCyclophoridae

(Minato, 2012)

0E0BD67F-F542-51FE-B2A0-90E06FD73DB1

Chamalycaeus takahashii muroharai Minato, 2012: 49–52, figs 1a–e, 2a, b.
Chamalycaeus takahashii muroharai – Yano 2015: 233–236, fig. 1.

###### Type locality.

“Limestone outcrops of the Koumori-do Cave, Honjo-Kazato, Saeki City, Oita Prefecture”.

###### Material examined.

NSMT-Mo 77464 (holotype).

###### Remarks.

Protoconch low, very finely granulated, moderately glossy; with fine wrinkles on the last 0.25 of whorl; R2 with ca. 42 low, blunt ribs, which are situated very close to each other near the tube; near the middle of the tube the ribs reach each other and form connections with their neighbours; space between ribs at edge of body whorl roughly twice as large as a rib width; no spiral lines visible on R1.

##### Dicharax (?) tanegashimae

Taxon classificationAnimaliaGastropodaCyclophoridae

(Pilsbry, 1902)

45ADF286-EF68-5F8D-9F1D-B974F5897780

Alycaeus tanegashimae Pilsbry, 1902c: 562.
Chamalycaeus tanegashimae – Azuma 1982: 10, pl. 3, fig. 32.
Chamalycaeus satsumanus tanegashimae – Minato 1988: 14.
Dicharax (?) tanegashimae – Páll-Gergely and Asami 2017: 14, figs 7C, D.

###### Type locality.

“Tane-ga-shima, Osumi”.

###### Material examined.

ANSP 82480a (lectotype, designated by Baker 1964, photographs examined); 種子ヶ島 = 種子島 (Tanegashima), Toru Inaba coll., NSMT-Mo 66469 (5 shells).

###### Remarks.

Protoconch low, finely granulated, rather matte; R2 with ca. 20 low, blunt, regular ribs which are some distance from each other even near the tube; distance between ribs at edge of body whorl is larger than a rib width; no spiral lines visible on the teleoconch.

Minato (1988) treated *Chamalycaeus tanegashimae* as a subspecies of *Chamalycaeus satsumanus*. According to our classification, *Chamalycaeus satsumanus* and *Sigmacharax tanegashimae* belong to different genera (*Dicharax* and *Metalycaeus*, respectively).

##### Dicharax (?) tokunoshimanus

Taxon classificationAnimaliaGastropodaCyclophoridae

(Pilsbry & Y. Hirase, 1904)

22EE3E22-32C8-5484-A789-058A5C323194

Alycaeus tokunoshimanus Pilsbry & Y. Hirase, 1904c: 617–618.
Chamalycaeus tokunoshimanus – Azuma 1982: 9, pl. 3, fig. 27.
Chamalycaeus tokunoshimanus tokunoshimanus – Minato 1988: 13.

###### Type locality.

“Tokunoshima, Ōsumi”.

###### Material examined.

ANSP 87505 (lectotype, designated by Baker 1964, photographs examined).

###### Remarks.

Protoconch low, finely granulated; R1 with low, irregular wrinkles; R2 with very low, regularly spaced ribs, which are in contact with each other near the tube.

##### Dicharax (?) tokunoshimanus principalis

Taxon classificationAnimaliaGastropodaCyclophoridae

(Pilsbry & Y. Hirase, 1909)

FCD81336-F51D-57BA-84CD-7A9BBBA7736C

Alycaeus tokunoshimanus principalis Pilsbry & Y. Hirase, 1909a: 587.
Chamalycaeus tokunoshimanus – Azuma 1982: 9–10, pl. 3, fig. 28.
Chamalycaeus tokunoshimanus principalis – Minato 1988: 13.

###### Type locality.

“Ōgachi, Ōshima, (Ōsumi)”.

###### Material examined.

ANSP 95830a (lectotype, designated by Baker 1964, photographs examined); 鹿児島縣1奄美大島名瀬市平田町 (= Kagoshima-ken, Amami Oshima, Naze-shi, Hirata-cho = Kagoshima Prefecture, Amami Oshima, Naze City, Hirata Town), Sakurai coll., NSMT-Mo 79099 (3 shells).

###### Remarks.

Protoconch low and finely granulated, the last 0.25 of the protoconch whorl is finely wrinkled; R2 with ca. 34–40 low, blunt ribs; they are situated close to each other near tube, but probably do not form connections with each other; distance between ribs at the edge of body whorl ca. as wide as a rib width; R1 with extremely fine spiral lines between ribs (probably not homologous with the spiral striations in *Metalycaeus* species).

##### Dicharax (?) tokunoshimanus mediocris

Taxon classificationAnimaliaGastropodaCyclophoridae

(Pilsbry & Y. Hirase, 1909)

B2B16438-A7CF-5DBB-92E1-32FFAA0FEE4A

Alycaeus tokunoshimanus mediocris Pilsbry & Y. Hirase, 1909a: 587.

###### Type locality.

“Yorojima (Ōsumi)”.

###### Material examined.

Yorojima (Ōsumi), coll. Hirase, ‘05, ANSP 89926 (lectotype, designated by Baker 1964, photographs examined).

###### Remarks.

Mentioned traits are the same as those of the nominotypical subspecies.

##### Dicharax (?) tsushimanus

Taxon classificationAnimaliaGastropodaCyclophoridae

(Pilsbry & Y. Hirase, 1909)

39131C38-5D09-573B-B11E-28D8ABD16396

Alycaeus (Metalycaeus) tsushimanus Pilsbry & Y. Hirase, 1909a: 586–587.
Chamalycaeus tsushimanus – Azuma 1982: 11, pl. 4, fig. 38; Minato 1988: 15.

###### Type locality.

“Tsushu, Tsushima”.

###### Material examined.

ANSP 95737 (lectotype, designated by Baker 1964, photographs examined); 長崎縣1壹岐神社 (Nagasaki-ken, Iki-jinja = Nagasaki Prefecture, Iki Shrine), Sakura coll., NSMT-Mo 79100 (2 shells).

###### Remarks.

Protoconch relatively low, very finely granulated, moderately glossy; R2 with ca. 22 low, blunt, irregular ribs which are connected to each other near the tube but are free further away from there; distance between ribs at edge of body whorl less than the a rib width; no spiral lines are visible on the teleoconch.

##### Dicharax (?) yanoshigehumii

Taxon classificationAnimaliaGastropodaCyclophoridae

(Minato, 1987)

72E3C5BF-C106-5865-AAF2-8FF74C1C79D8

Chamalycaeus yanoshigehumii Minato, 1987a: 223–223, 225, figs 4–6.
Chamalycaeus yanoshigehumii – Minato 1988: 16.

###### Type locality.

“Tajigawa, Chunan-cho, Nakatado-gun, Kagawa-ken, Japan”.

###### Material examined.

NSMT-Mo 64219 (holotype); 香川縣1仲多度郡仲南町多治川 (Kagawa-ken, Nakatado-gun, Chunan-cho, Taji-gawa = Kagawa Prefecture, Nakatado County, Chunan Town, Taji River), coll. Sakurai, NSMT-Mo 79101 (4 shells).

###### Remarks.

Protoconch low, very finely granulated, moderately glossy, but the protoconch of the holotype was slightly weathered; however, those in the Sakurai collection were not; R2 with ca. 25 low, blunt, but well separated ribs; even near the tube the ribs seem to be free from each other; distance between ribs at edge of body whorl ca. as wide as a rib, or slightly wider; no spiral lines visible on R1.

##### Dicharax (?) yanoshokoae

Taxon classificationAnimaliaGastropodaCyclophoridae

(Yano & Matsuda, 2016)

1761A3EA-F62C-5B07-AB4E-C14BC73E2D6A

Awalycaeus sp. – Kawase et al. 2012: 85–88, fig. 5a.
Awalycaeus yanoshokoae Yano & Matsuda in Yano et al. 2016: 53–55, figs 1A–I, 3A, B, 4A-a–d.

###### Type locality.

“extended region of limestone area of Saruda-do Cave, Okina, Hidaka-mura, Takaoka-gun, Kochi Prefecture”.

###### Remarks.

No specimens were examined by us, but the photographs of the original description provide sufficient details of the fine sculpture. Protoconch low, rather glossy; R1 with relatively dense, strong ribs; R2 with low, very dense ribbing.

#### Genus Dioryx

Taxon classificationAnimaliaGastropodaCyclophoridae

Benson, 1859

D4363F54-3225-5752-818F-836036CA4B3D

Dioryx Benson, 1859: 177. [section (subgenus) of Alycaeus]
Dioryx – Kobelt and Möllendorff 1897: 146; Gude 1921: 198; Thiele 1929: 107; Wenz 1938: 477; Egorov 2013: 34.

##### Type species.

**Figure 26.**
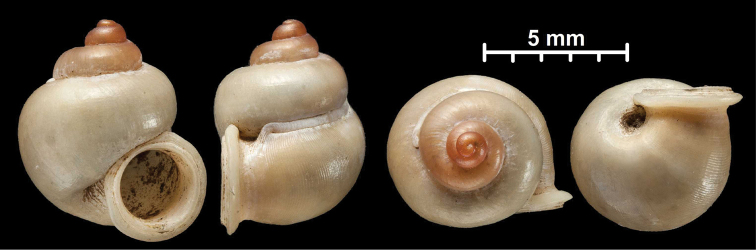
*Dioryx amphora* (Benson, 1856), probable syntype (UMZC I.102850; type species of *Dioryx*). Photographs: Harold Taylor (NHM).

*Alycaeus amphora* Benson, 1856 (Fig. 26), SD Gude (1921: 198).

##### Diagnosis.

Shell small to very large (3.5–9 mm), globose, sometimes the body whorl is angled or keeled; protoconch smooth, not spirally striated; R1 usually very finely reticulated due to fine radial ribs and fine spiral striation, or smooth; R2 short to long, usually without ribs (superficially smooth, with alternating lighter and darker stripes); R3 practically absent (the inner opening of the sutural tube is situated close to the aperture). Operculum thin, usually proteinaceous (“horny”), in some species can be thicker, calcified. Central tooth with 5–7 cusps, broad, central cusp blunt.

##### Differential diagnosis.

The combination of the globular shell, the reduced sculpture (only very fine spiral and radial lines are present), and the absence of the R2 region characteristic for the vast majority of alycaeid species make *Dioryx* species distinguishable from other alycaeid genera.

##### Distribution.

*Dioryx* is distributed from the southeastern Himalayan region to Taiwan in the east, and down to the northern part of the Malay Peninsula and southern Vietnam in the south (Fig. 7).

##### Remarks.

*Dioryx* is primarily classified based on the reduced R3. This character state is found in other genera of the Alycaeidae as well, such as in *Alycaeus conformis*, *Chamalycaeus microconus*, and in *Awalycaeus* (treated as a synonym of *Dicharax*). Even presuming that similar morphology has appeared multiple times in the Alycaeidae, we can reasonably assume the monophyly of *Dioryx* due to the uniform, simple sculpture across all species, and the generally globose shell.

#### Dioryx amphora

Taxon classificationAnimaliaGastropodaCyclophoridae

(Benson, 1856)

5BAC7BEB-F533-5022-9640-AA7077F092F2

Fig. 26

Alycaeus Amphora Benson, 1856: 226.
Alycaeus amphora – Reeve 1878: pl. 2, species 15.
Dioryx amphora – Kobelt and Möllendorff 1897: 146; Kobelt 1902: 336–337; Gude 1921: 198–199, fig. 31.
Alycaeus (Dioryx) amphora – Godwin-Austen 1914: 429–430, pl. 153, figs 11, 11a, 11b.

##### Type locality.

“ad Moulmein, et in valle Tenasserim”.

##### Material examined.

UMZC I.102850 (1 syntype); NHMUK 1906.5.5.23 (figured by Godwin-Austen; this sample contains 2 *Dioryx amphora* shells, and a third shell of another *Dioryx* species).

##### Remarks.

Protoconch matte, without spiral striae; R2 with regular spiral striation, and weaker, irregular radial lines; the overall sculpture is weak; R2 very long, reaches 150°, its surface smooth, with alternating narrow light and slightly thicker dark stripes.

#### Dioryx bacca

Taxon classificationAnimaliaGastropodaCyclophoridae

(L. Pfeiffer, 1862)

2DE6860C-6775-5636-9B80-7F6296C5FEA2

Alycaeus bacca L. Pfeiffer, 1862: 275–276.
Alycaeus bacci [sic] – Reeve 1878: pl. 3, species 26.
Dioryx bacca – Kobelt and Möllendorff 1897: 146; Kobelt 1902: 337; Páll-Gergely et al. 2017: 10; Inkhavilay et al. 2019: 15, fig. 5C, D.

##### Type locality.

“Lao Mountains, Camboja”.

##### Material examined.

Lao Mts., coll. Demon, NHMUK 1903.7.1.2714 (1 shell).

##### Remarks.

The examined shell was weathered. Protoconch without spiral striae; R1 with very fine spiral striation and weaker, irregular growth lines; R2 short, smooth, alternating narrow/lighter and thicker/darker stripes.

#### Dioryx cariniger

Taxon classificationAnimaliaGastropodaCyclophoridae

Möllendorff, 1897

9AC91EAE-4AC4-5552-836E-66255EBC87C8

Dioryx cariniger Möllendorff, 1897a: 41.
Dioryx cariniger – Kobelt and Möllendorff 1897: 146; Kobelt 1902: 337; Zilch 1957: 141, pl. 5, fig. 1; Páll-Gergely et al. 2017: 10; Inkhavilay et al. 2019: 15, fig. 5E, F.

##### Type locality.

“Prope oppidum Luang-Prabangin regione Laos”.

##### Material examined.

Hinter Indien: Lakon (Laos-Gebiet), coll. Möllendorff, SMF 109263 (lectotype, designated by Zilch 1957); Same data, SMF 109264 (paralectotype).

##### Remarks.

Protoconch very finely tuberculated; R1 without radial lines, only very weak growth ridges may be visible, but the region bears visible, closely spaced spiral striae; R2 relatively long, with alternating darker and lighter stipes, lighter ones only slightly narrower than dark ones.

#### Dioryx cochinensis

Taxon classificationAnimaliaGastropodaCyclophoridae

(Godwin-Austen, 1914)

A60241DD-878B-54DF-BBE7-1C4301833693

Alycaeus (Dioryx) cochinensis Godwin-Austen, 1914: 428–429, pl. 156, figs 7, 7a.
Dioryx cochinensis – Páll-Gergely et al. 2017: 10.

##### Type locality.

“Cochin China”.

##### Material examined.

Cochin China, NHMUK 20170011 (holotype [single specimen mentioned in the original description], measurements of the specimen: D = 6.1, H = 7.0). See also Remarks.

##### Remarks.

Protoconch without spiral striae; R1 with very fine spiral striation and weaker, irregular, wavy growth lines; R2 very short, smooth, narrow light and thicker dark stripes alternating.

According to the original description of *A. cochinensis*, only a single shell was found “stuck on the same slab with *Alycaeus gibbus*” and originated from the collection of Hugh Cuming. We were unable to locate the holotype in the type collection of the NHM, but found a shell in the general collection labelled “*Dioryx cochinensis* G.-A. ms., Cochin China (Cuming)”, with a black glue mark on its body whorl indicating that it was previously mounted as indicated in the original description. This specimen is identical with the figures of Godwin-Austen (1914) (characteristic shell shape from apertural view: pl. 156, fig. 7; and the very short tube: pl. 156, fig. 7a). Therefore, we identify this specimen as the holotype of *Dioryx cochinensis*. The measurements provided by Godwin-Austen (1914) are the following: “major diam: 5.5; alt. axis 4.75 mm”. Our measurements of the specimen are, however, somewhat larger, namely D: 6.1 mm, H: 7 mm. This is probably due to the inaccuracy of the original measurements (as usual in Godwin-Austen’s specimens), and we do not question the status of the specimen.

#### Dioryx compactus

Taxon classificationAnimaliaGastropodaCyclophoridae

(Bavay & Dautzenberg, 1900)

7F82E018-DCA1-5A77-A796-6E03EE6DE39C

Alycaeus (Dioryx) compactus Bavay & Dautzenberg, 1900a: 119–120.
Alycaeus (Dioryx) compactus – Bavay and Dautzenberg 1900b: 454, pl. 11, figs 9, 10.
Dioryx compactus – Kobelt 1902: 337–338; Varga 1972: 136, figs 15, 16; Páll-Gergely et al. 2017: 10, fig. 4C.

##### Type locality.

“Bac-Kan”.

##### Material examined.

Tonkin, Bac-Kan, leg. Messager, MNHN-IM-2000-31796 (1 syntype).

##### Remarks.

Five shells in the Senckenberg Museum (Tonkin, Bac-Kan, Cho-Ra, leg. Messager, coll. Rolle ex coll. Bosch, SMF 192287) were labelled as syntypes, although they clearly belong to a different species than the single syntype in MNHN Paris, which agrees with the figures in Bavay and Dautzenberg (1900b). This single shell is identical to *Dioryx distortus*, but smaller. A comprehensive revision would probably reveal that the two names are synonyms.

The following remarks are based on the syntype from Paris: protoconch matte; R1 with irregular, very weak, widely spaced ribs and dense spiral striae of the same strength, although due to small space between spiral striae, they are much more conspicuous than the rare radial lines; R2 long, with very thin lighter and darker thicker stripes, the overall surface is smooth, glossy.

#### Dioryx dautzenbergi

Taxon classificationAnimaliaGastropodaCyclophoridae

Páll-Gergely, 2017

72F6B891-D3DD-503B-86D7-C528A8CEB50F

Alycaeus (Dioryx) major Bavay & Dautzenberg, 1900a: 118–119.
Alycaeus (Dioryx) major – Bavay and Dautzenberg 1900b: 452, pl. 11, figs 4–6.
Dioryx major – Kobelt 1902: 338–339.
Alycaeus major – Rees 1964: 63, pl. 5, fig. 26.
Dioryx dautzenbergi Páll-Gergely in Páll-Gergely et al., 2017: 10, fig. 4A. (replacement name for Alycaeus (Dioryx) major Bavay & Dautzenberg, 1900, non Alycaeus (Dioryx) granum var. major Godwin-Austen, 1893)

##### Type locality.

“Phi-Mi”.

##### Material examined.

Phi-Mi, Haut Tonkin, leg. Messager, MNHN-IM-2000-31788 (1 syntype): Tonkin, NHMW 41003 (2 shells).

##### Remarks.

Protoconch glossy; R1 with very weak growth lines and no spiral striae; R2 relatively short, smooth, very narrow lighter and thicker, darker stripes alternating.

#### Dioryx distortus

Taxon classificationAnimaliaGastropodaCyclophoridae

(Haines, 1855)

1D39AED0-21A5-57C4-AF0F-BDD4CEAD189F

Cyclostoma distortum Haines, 1855: 158, pl. 5, figs 5–8.
Alycaeus distortus – Reeve 1878: pl. 3, species 24.
Dioryx distortus – Kobelt and Möllendorff 1897: 146; Kobelt 1902: 337.

##### Type locality.

“Siam”.

##### Material examined.

Siam: coll. Haines, AMNH 62467 (1 syntype).

##### Remarks.

Protoconch matte, without spiral striation; the overall sculpture very weak; first whorl of R1 with spiral striation and widely spaced, irregular radial lines; later spiral lines disappear and radial lines become regular and dense; R2 very long, reaches ca. 150°, it is entirely smooth, only slimmer light and thicker darker stripes alternate.

#### Dioryx dongiensis

Taxon classificationAnimaliaGastropodaCyclophoridae

Varga, 1972

E081B1AE-D76A-5C33-88A2-B899F6AB1E85

Dioryx dongiensis Varga, 1972: 135–137, figs 21–23.
Dioryx cf. dongiensis – Egorov 2013: 34, fig. 61c.
Dioryx dongiensis – Páll-Gergely et al. 2017: 10.

##### Type locality.

“Vietnam, Tonkin, Cuc Phuong bei Dong, felsiger Regenwald”.

##### Material examined.

Vietnam: Tonkin: Cúc Phuong: Đang: sziklás esőerdő, leg. T. Pócs, 23.10.1962, HNHM 11909 (holotype).

##### Remarks.

The holotype was weathered, but the protoconch seemed to be without sculpture. R2 very long, smooth.

#### Dioryx feddenianus

Taxon classificationAnimaliaGastropodaCyclophoridae

(Theobald, 1870)

97C6A266-CE70-57B7-80F7-BAC8D06114F8

Alycaeus Feddenianus Theobald, 1870: 397, pl. 18, fig. 4.
Alycaeus feddenianus – Reeve 1878: pl. 2, species 18.
Dioryx feddenianus – Kobelt and Möllendorff 1897: 146; Kobelt 1902: 338; Gude 1921: 199, fig. 32.
Alycaeus (Dioryx) feddenianus Godwin-Austen 1914: 415, pl. 153, fig. 12.

##### Type locality.

“Shan States”.

##### Material examined.

Upper Salwin, NHMUK 1888.12.4.927–930 (4 shells).

##### Remarks.

Protoconch matte, without spiral striation; R1 with widely spaced spiral striae, and weaker, much denser radial lines; R2 exceeds 90°, it is smooth, with slimmer lighter and thicker darker stripes alternating.

#### Dioryx globuloides

Taxon classificationAnimaliaGastropodaCyclophoridae

Zilch, 1957

F0C2A791-CE29-5752-B0F7-8E6375CD400F

Alycaeus globulus Möllendorff, 1885: 162.
Dioryx globulus – Kobelt and Möllendorff 1897: 146; Kobelt 1902: 338; Yen 1939: 28, pl. 2, fig. 28.
Dioryx globuloides Zilch, 1957: 141, pl. 5, fig. 2 (replacement name for globulus Möllendorff, 1885, non Godwin-Austen, 1874).
Dioryx globuloides – Páll-Gergely et al. 2017: 10, fig. 4D.

##### Type locality.

“in regione Badung provinciae sinensis Hubei”.

##### Material examined.

Patung, Hupei: China, coll. Möllendorff, SMF 39221 (lectotype, designated by Zilch 1957); Same data, SMF 39222 (10 paralectotypes).

##### Remarks.

Protoconch rather matte, extremely finely granulated; R1 with irregular, fine, low wrinkles; R2 short, with darker, thick and lighter, narrow stripes alternating.

#### Dioryx globulosus

Taxon classificationAnimaliaGastropodaCyclophoridae

(Godwin-Austen, 1914)

58EE2BD6-AAC6-5793-BB4E-E5219E870B54

Alycaeus (Dioryx) globulosus Godwin-Austen, 1914: 368–369, pl. 157, figs 1, 1a.
Dioryx globulosus – Gude 1921: 200; Ramakrishna et al. 2010: 74; Tripathy et al. 2018: 789.

##### Type locality.

“Luyor, Tsanspu Valley”.

##### Material examined.

Luyor, Abor, 7,200 f., leg. Oakes, NHMUK 1903.7.1.3528 (7 syntypes in two different vials).

##### Remarks.

Protoconch glossy; R1 with irregular, weak ribs and very weak spiral striae; R2 relatively short, with very narrow lighter and darker thicker stripes; overall surface smooth.

#### Dioryx kobeltianus

Taxon classificationAnimaliaGastropodaCyclophoridae

(Möllendorff, 1875)

DD4F5E74-F287-52EA-BD05-7FA19F690F72

Alycaeus Kobeltianus Möllendorff, 1875: 121–122.
Alycaeus (Dioryx) kobeltianus – Schmacker and Boettger 1890: 121, pl. 2, fig. 3.
Dioryx kobeltianus – Kobelt and Möllendorff 1897: 146; Kobelt 1902: 338; Yen 1939: 29, pl. 2, fig. 30; Zilch 1957: 141, pl. 5, fig. 3; Varga 1972: 136, figs 17, 18; Páll-Gergely et al. 2017: 10, fig. 4E.

##### Type locality.

“Berge bei Kiukiang”.

##### Material examined.

Kiukiang, China, coll. Möllendorff 1874, SMF 39298 (syntype, labelled as holotype [the number of available shells was not stated in the original description]).

##### Remarks.

The original description does not mention the number of examined specimens. Thus, we consider the specimen labelled as holotype (SMF 39298) syntype.

Protoconch very finely granulated; R1 with fine, rather irregular growth lines and very fine spiral lines; R1 relatively short, smooth, with alternating dark, wider and light, slimmer stripes.

#### Dioryx labrirubidum

Taxon classificationAnimaliaGastropodaCyclophoridae

(Godwin-Austen, 1914)

F4C44327-52D7-51BE-AB61-5C4070D63BC1

Fig. 27

Alycaeus n. sp. – Nevill 1878: 292 (partim)
Alycaeus (Dioryx) labrirubidum Godwin-Austen, 1914: 430, pl. 155, figs 1, 1a.
Dioryx labrirubidum – Gude 1921: 200.

##### Type locality.

“Near Moulmein”.

##### Material examined.

Khargan, Attaran valley, coll. W. Theobald, NZSI M.8056 (syntype, labelled as holotype [the original description is based on multiple specimens]).

##### Remarks.

Protoconch smooth, without spiral striation, R1 with irregular, fine wrinkles; R2 ca. 0.25 whorl long, with irregularly arranged (probably teratological condition?) lighter, narrow, and thicker darker stripes.

**Figure 27.**
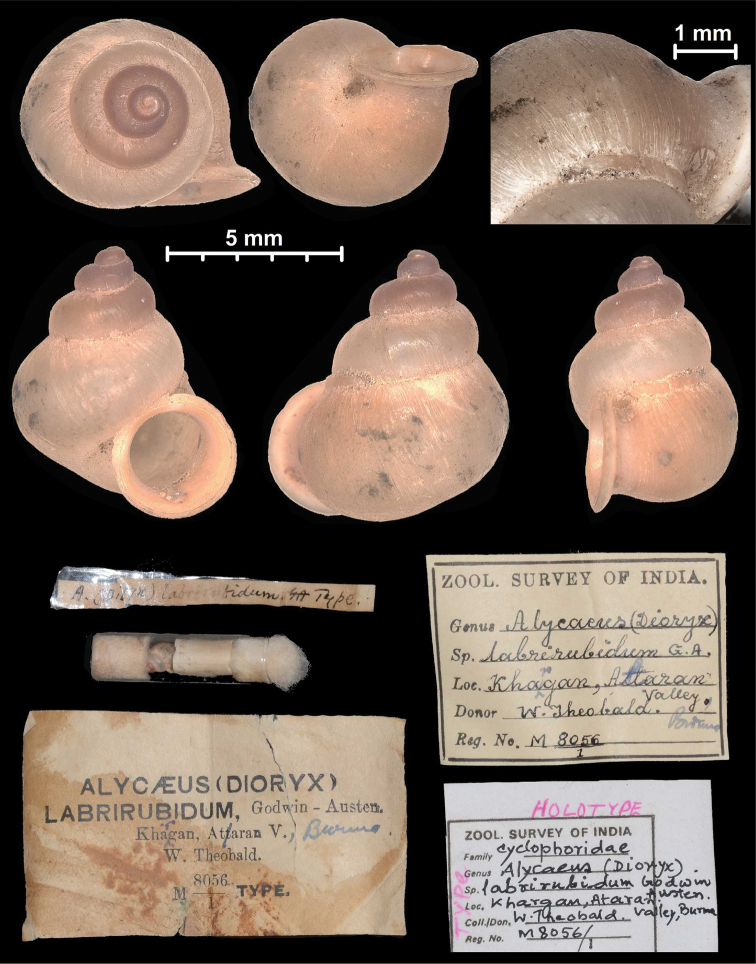
*Dioryx labrirubidum* (Godwin-Austen, 1914), syntype (NZSI M.8056). Photographs: Sheikh Sajan.

#### Dioryx menglunensis

Taxon classificationAnimaliaGastropodaCyclophoridae

Chen & Zhang, 1998

EBA4F895-323D-5DD5-A909-22DF48684B57

Fig. 28

Dioryx menglunensis Chen & Zhang, 1998: 349, 358, figs 4, 5 (erroneously labelled as Pupina menglunensis).
Dioryx menglunensis – Páll-Gergely et al. 2017: 10.

##### Type locality.

“Menglun, Mengla County (21°09'N, 101°02'E), Yunnan Province, China”.

##### Material examined.

Meng-Lun town, Meng-La County, Xi-Shuang-Ban-Na Dai Autonomous Prefecture, China, leg. Chen De-Niu, 1994.5.3, TM 046653 (holotype, deposited in IZCAS); TM 086956 (paratype) same as holotype.

##### Remarks.

Protoconch finely granulate, matte; R1 consisting with fine, irregular growth lines, and dense, fine, regular spiral striation; R2 long, reaches 180°, glossy, with lighter, slimmer, and darker, thicker stripes interchanging, and with fine, obscure spiral sculpture; constriction deep and relatively long.

**Figure 28.**
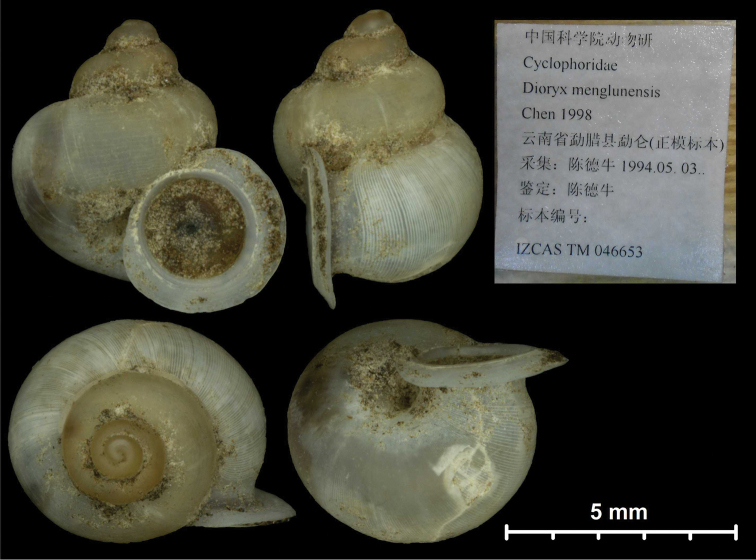
*Dioryx menglunensis* Chen & Zhang, 1998, holotype (IZCAS TM 046653). Photographs: Kaibaryer Meng.

#### Dioryx messageri

Taxon classificationAnimaliaGastropodaCyclophoridae

(Bavay & Dautzenberg, 1900)

7EEE193C-C87F-5D2E-A947-3705CF6508F1

Alycaeus (Dioryx) messageri Bavay & Dautzenberg, 1900a: 119.
Alycaeus (Dioryx) messageri – Bavay and Dautzenberg 1900b: 453, pl. 11, figs 7, 8.
Dioryx messageri – Kobelt 1902: 339; Páll-Gergely et al. 2017: 10, fig. 4B; Inkhavilay et al. 2019: 15, fig. 6A (fig. 6B is D. rosea).

##### Type locality.

“That-Khé”.

##### Material examined.

That-Khé, leg. Messager, MNHN-IM-2000-31785 (1 syntype); Tonkin, NHMW 41004 (2 shells).

##### Remarks.

Protoconch rather glossy, R1 with widely spaced, low, irregular ribs and dense, rather prominent spiral striae; R2 of ca. 90°, with alternating thinner/lighter and thicker/darker stripes.

#### Dioryx monadicus

Taxon classificationAnimaliaGastropodaCyclophoridae

(Heude, 1890)

27501CE2-1371-57BD-BB59-BDFBA1B85554

Alycoeus [sic] monadicus Heude, 1890: 130, 36, fig. 14.
Dioryx monadicus – Kobelt and Möllendorff 1897: 147; Kobelt 1902: 339; Páll-Gergely et al. 2017: 10.

##### Type locality.

“In montosis Tchen-k’eou”.

##### Material examined.

China: T’chen-keou, leg. Farges, ex coll. Mus. Heude, MCZ 167208 (1 syntype).

##### Description.

protoconch finely granulate; R1 with irregular, fine growth lines (no spiral striation visible); R2 smooth, with alternating white/narrow and darker/thicker stripes.

#### Dioryx pilula

Taxon classificationAnimaliaGastropodaCyclophoridae

(Gould, 1859)

7920030B-00AF-5A93-A7CD-55A4BA02E7D2

Alycaeus pilula Gould, 1859: 424–425.
Dioryx pilula and pilula var. minor – Kobelt and Möllendorff 1897: 147.
Alycaeus (Dioryx) pilula – Bavay and Dautzenberg 1900b: 456–457.
Dioryx pilula – Kobelt 1902: 339; Yen 1939: 28, pl. 2, fig. 29; Zilch 1957: 141; Varga 1972: 136, figs 19, 20; Páll-Gergely et al. 2017: 10.
Alcaeus [sic] pilulaJohnson 1964: 127.

##### Type locality.

“Hong Kong, China”.

##### Material examined.

Hong Kong, ex coll. Möllendorff, ex coll. Mus. Heude, MCZ 167223 (1 shell).

##### Remarks.

Protoconch extremely finely granulated, rather matte; R1 with irregular growth ridges and very fine spiral striation; R2 relatively short, with extremely fine alternating wider/darker and slimmer/lighter stripes.

Johnson (1964) did not find the type lot in the Smithsonian Museum. The sample we examined was collected at the type locality.

#### Dioryx pingoungensis

Taxon classificationAnimaliaGastropodaCyclophoridae

(Godwin-Austen, 1914)

1172244A-1BC4-5F79-912A-AA61DE953D25

Alycaeus (Dioryx) pingoungensis Godwin-Austen, 1914: 414–415, pl. 153, figs 13, 13a.
Dioryx pingoungensis – Gude 1921: 200–201.

##### Type locality.

“Pinguong, Shan Hills”.

##### Material examined.

Shan Hills, Pingoun, 2500 ft., leg. Ponsonby, NHMUK 1913.3.16.9 (1 syntype).

##### Remarks.

Protoconch matte, without notable sculpture; R1 smooth, with very low, irregular growth lines; R2 ca. 0.25 of whorl long, smooth, with alternating thinner/lighter and thicker/darker stripes.

#### Dioryx pocsi

Taxon classificationAnimaliaGastropodaCyclophoridae

Varga, 1972

2778E748-9C35-5537-9887-9338C31C54FA

Dioryx pocsi Varga, 1972: 135, figs 12–14.
Dioryx pocsi – Páll-Gergely et al. 2017: 10.

##### Type locality.

“Vietnam, Tonkin, Cuc Phuong bei Dong”.

##### Material examined.

Tonkin: Cúc Phuong bei Đang, leg. T. Pócs, 1963, HNHM 11964 (holotype).

##### Remarks.

Protoconch smooth, R2 long, entirely smooth, alternating narrow/lighter and thicker/darker stripes visible.

#### Dioryx requiescens

Taxon classificationAnimaliaGastropodaCyclophoridae

(Mabille, 1887)

665EE5A4-454D-58D9-A2A1-74A9FF345550

Alycaeus requiescens Mabille, 1887: 151, pl. 3, figs 11, 12.
Alycaeus (Alycaeus) requiescens – Kobelt 1902: 349.
Dioryx requiescens – Páll-Gergely et al. 2017: 10.

##### Type locality.

“Tonkin” (from the title).

##### Material examined.

Tonkin, leg. Balansa 1887, MNHN-IM-2000-31787 (8 syntypes).

##### Remarks.

Shell shape typical *Dioryx*; apex relatively elevated, matte; R1 with irregular, widely spaced, low ribs and very slightly stronger, dense spiral striation, which is the strongest on the first whorl of R1; R2 long, with dense alternating lighter/narrow and darker/thicker stripes; overall surface smooth.

#### Dioryx rosea

Taxon classificationAnimaliaGastropodaCyclophoridae

(Bavay & Dautzenberg, 1900)

EC00DD7C-3EA3-53A6-A939-242856778B3D

Alycaeus (Dioryx) messageri var. rosea – Bavay & Dautzenberg: 1900b: 453.
Dioryx messageri var. rosea – Kobelt 1902: 339.
Dioryx messageri – Inkhavilay et al. 2019: fig. 6B is D. rosea.

##### Type locality.

“That-Khé”.

##### Material examined.

RBINS MT.1005 (4 syntypes).

##### Remarks.

Protoconch rather matte, R1 consists with very weak, dense, inconspicuous growth lines, extremely weak spiral striation; R2 smooth, with alternating narrow/lighter and thicker/darker stripes.

This species differs from *D. messageri* in stable characters and they are found in the RBINS in multiple mixed samples. Therefore, we handle *D. rosea* as a separate species. A redescription will be presented in a separate paper.

#### Dioryx ruyangensis

Taxon classificationAnimaliaGastropodaCyclophoridae

Hu, Yin, & Chen, 2004

22F94D21-14E5-5D10-AA1E-8C91403BA35F

Fig. 29

Dioryx ruyangensis Hu, Yin, & Chen, 2004: 704–705.
Dioryx ruyangensis – Páll-Gergely et al. 2017: 10.

##### Type locality.

“Ruyang Town, Ruyuan County (24°7'N, 113°2"E), Guangdong Province, China”.

##### Remarks.

The holotype (IZCAS TM 132794) and a paratype (IZCAS TM 132795–132805) were photographed by one of us (Meng Kaibaryer). The shell height was 4.7 and 4.6 mm respectively, whereas it is given as 7.1 mm in the original description. Moreover, the examined specimens were much more corpulent than the one imaged in Hu et al. (2004), indicating that they belong to another species. Consequently, the types of this species are considered lost.

**Figure 29.**
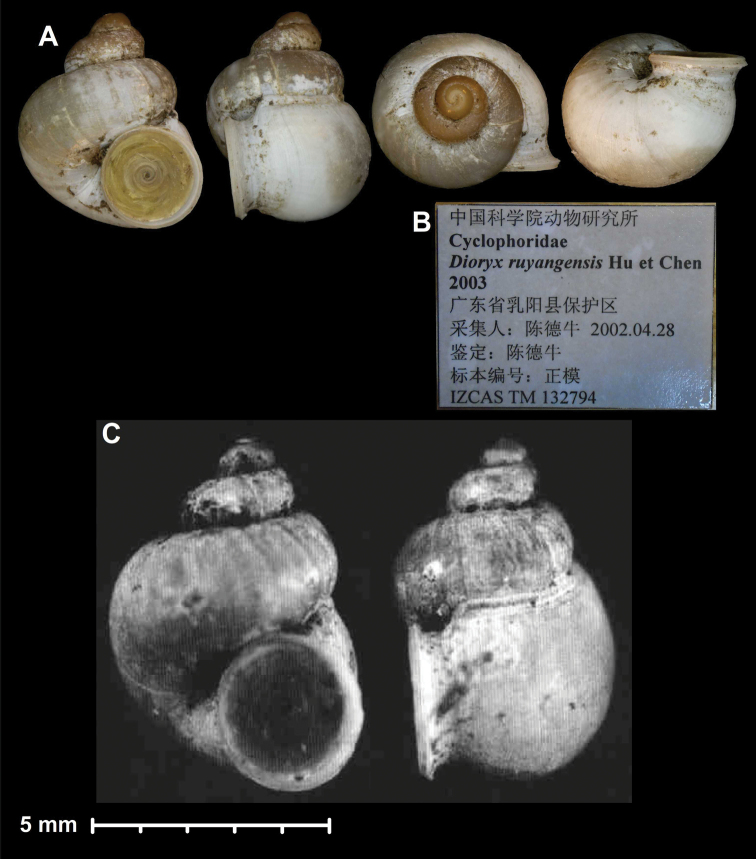
Shells of *Dioryx* Benson, 1859 species **A***Dioryx ruyangensis* Hu, Yin, & Chen, 2004, holotype (IZCAS TM 132794) **B** label of the holotype **C** figures from the original description of *Dioryx ruyangensis* Hu, Yin, & Chen, 2004. Photographs: Kaibaryer Meng (**A, B**).

#### Dioryx setchuanensis

Taxon classificationAnimaliaGastropodaCyclophoridae

(Heude, 1885)

4D86D6CC-8224-5BFD-A154-F402DBEC1D99

Alycoeus [sic] setchuanensis Heude, 1885: 97, pl. 24, figs 6, 6a–c.
Dioryx setchuanensis – Kobelt and Möllendorff 1897: 147; Kobelt 1902: 340; Páll-Gergely et al. 2017: 10.

##### Type locality.

“In ditione Tchen k’eou”.

##### Material examined.

China: Tchen-k’eou, coll. Farges, ex Mus. Heude, 1946, MCZ 167231 (4 syntypes).

##### Remarks.

Protoconch matte; R1 with inconspicuous, irregular growth lines and very fine, dense spiral striation (strength of spiral striation is variable); R2 smooth, with alternating lighter/slimmer and darker, slightly thicker stripes.

According to Johnson (1973) syntypes are also present in the USNM (inventory number: 472337).

#### Dioryx swinhoei

Taxon classificationAnimaliaGastropodaCyclophoridae

(H. Adams, 1866)

FF2351DC-EF98-5864-8740-FBF96D0D8D48

Alycaeus (Dioryx) swinhoei H. Adams, 1866: 318, pl. 33, figs 11, 11a.
Alycaeus swinhoei – Reeve 1878: pl. 3, species 21.
Dioryx swinhoei – Kobelt and Möllendorff 1897: 147; Kobelt 1902: 340; Hsieh et al. 2006: 88 + figures; Hwang 2014: 8, fig. 1F.

##### Type locality.

“Takow, Formosa”.

##### Material examined.

Formosa, NHMUK 1866.5.9.8 (lectotype, designated by Hwang 2014); Same data, NHMUK 1866.5.9.9 (1 paralectotype); Formosa: Sammaipo, leg. Hirase, 1906, 1378a (probably locality code of Hirase), coll. Jetschin ex coll. K.L. Pfeiffer, SMF 109780 (2 shells).

##### Remarks.

Both the lectotype and the paralectotype were strongly weathered. Protoconch without notable sculpture; R1 with some radial sculpture; R2 short, with alternating narrow/lighter and thicker/darker stripes, overall surface smooth. The specimens deposited in the Senckenberg Museum were not weathered, and the sculpture could be examined in more detail: protoconch rather glossy, R1 with very fine, irregular radial lines and weak spiral striation.

#### Dioryx tangmaiensis

Taxon classificationAnimaliaGastropodaCyclophoridae

Chen & Zhang, 2001

8017D0C2-431C-590C-AB6C-0569A5D514D4

Fig. 30

Dioryx tangmaiensis Chen & Zhang, 2001: 185–186, 189, figs 5, 6.
Dioryx tangmaiensis – Páll-Gergely et al. 2017: 10.

##### Type locality.

“Tongmai Town, (30°01'N, 95°E), Bomi County, Tibet Autonomous Region, China”.

##### Material examined.

Tong-Mai Town, Bo-Mi County, Tibet Autonomous Region, China, leg. Chen De-Niu & Gao Jia-Xiang, 1980.6.20, CASIZ TM 008902 (holotype, deposited in IZCAS); same data as holotype, leg. Gao Jia-Xiang, CASIZ TM 008903–008913 (paratypes).

##### Remarks.

Protoconch without notable sculpture; R1 with fine, irregular growth lines (some of them are almost represented as widely-spaced ribs) and very fine spiral striation; R2 ca. 0.25 whorl long, with alternating darker/wider and very lighter/narrow stripes.

**Figure 30.**
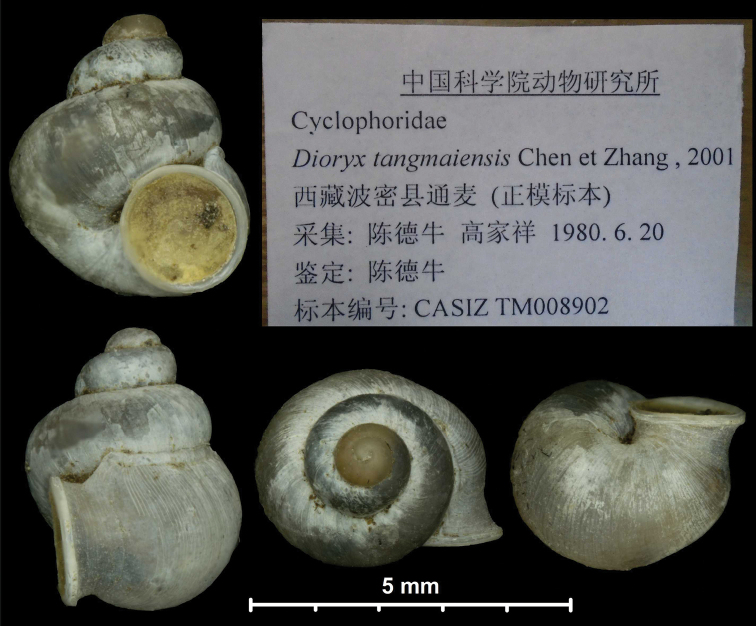
*Dioryx tangmaiensis* Chen & Zhang, 2001, holotype (CASIZ TM 008902). Photographs: Kaibaryer Meng.

#### Dioryx urceolus

Taxon classificationAnimaliaGastropodaCyclophoridae

(Godwin-Austen, 1914)

CD30110B-343D-5481-BA6A-1793EFDC2C03

Alycaeus (Dioryx) urceolus Godwin-Austen, 1914: 369, pl. 153, figs 9, 9a.
Dioryx urceolus – Gude 1921: 201; Ramakrishna et al. 2010: 74; Tripathy et al. 2018: 789.

##### Type locality.

“Abor Hills”.

##### Material examined.

Abor Hills, leg. Oakes, NHMUK 1903.7.1.3084 (1 syntype).

##### Remarks.

The whole shell was weathered so the original sculpture could not be examined. R2 relatively short.

#### Dioryx urnula

Taxon classificationAnimaliaGastropodaCyclophoridae

(Benson, 1853)

41C5E74A-C9F4-5961-A8A3-D3365AA95199

Alycaeus Urnula Benson, 1853: 284–285.
Alycaeus urnula – Reeve 1878: pl. 2, species 13.
Dioryx urnula – Kobelt and Möllendorff 1897: 147; Kobelt 1902: 340; Gude 1921: 201–202; Ramakrishna et al. 2010: 74; Tripathy et al. 2018: 789.
Alycaeus (Dioryx) urnula – Godwin-Austen 1914: 345–346, pl. 153, figs 1, 1a.
Alycaeus (Dioryx) urnula , Large variety – Godwin-Austen 1914: 346, pl. 153, fig. 2.

##### Type locality.

“ad Darjiling Himalayanum”.

##### Material examined.

Darjiling UMZC I.103780 (2 syntypes, photographs examined); Sikkim, coll. C. Bosch ex coll. Rolle, SMF 192286 (4 shells); Damsang, Daling District, NHMUK 1903.7.1.2589 (1 shell; “Large variety “).

##### Remarks.

Protoconch very finely granulated, R1 with hardly visible, oblique ribs, R2 normal length, smooth with alternating darker/wider and lighter/narrower stripes.

#### Dioryx urnula anghamiensis

Taxon classificationAnimaliaGastropodaCyclophoridae

(Godwin-Austen, 1914)

CC6D18CB-49B1-5E28-871B-51E697F8B19C

Alycaeus (Dioryx) urnula var. anghamiensis Godwin-Austen, 1914: 402, pl. 153, figs 6, 6a.
Dioryx urnula var. anghamiensis – Gude 1921: 202–203.

##### Type locality.

“Japvo Peak, Naga Hills, 9890 ft”.

##### Material examined.

Japvo Peak, Naga, 10,000 f., leg. Godwin-Austen, NHMUK 1903.7.1.2530 (4 syntypes, one of the separated by pink wool).

##### Remarks.

Protoconch smooth, R1 with irregular, very fine radial lines; R2 moderately long (ca. 90°), seemingly smooth, with alternating lighter/slimmer and darker/thicker stripes.

#### Dioryx urnula niosiensis

Taxon classificationAnimaliaGastropodaCyclophoridae

Páll-Gergely nom. nov.

0D2A37DC-CB4F-53FA-9031-8E91071B8EE9

Alycaeus (Dioryx) urnula var. daflaensis Godwin-Austen, 1914: 360, pl. 153, fig. 4 (non Alycaeus daflaensis Godwin-Austen, 1876).
Dioryx urnula var. daflaensis – Gude 1921: 203.

##### Type locality.

“Dafla Hills, Niosi Ridge, and Toruputu Peak”.

##### Etymology.

The replacement name *niosiensis* refers to the type locality (Niosi Ridge).

##### Remarks.

Alycaeus (Dioryx) urnula daflaensis Godwin-Austen, 1914 is a junior primary homonym of *Alycaeus daflaensis* Godwin-Austen, 1876. Therefore, a replacement name is proposed here.

#### Dioryx urnula rotundus

Taxon classificationAnimaliaGastropodaCyclophoridae

Páll-Gergely nom. nov.

1AEAE428-1569-55D2-8CC5-DCC342632DF3

Alycaeus (Dioryx) urnula var. globosus Godwin-Austen, 1914: 363, pl. 153, fig. 8 (non Alycaeus globosus H. Adams 1870).
Dioryx urnula var. globosa – Gude 1921: 202.

##### Type locality.

“Brahmakund, E. Assam”.

##### Material examined.

Brahmakund, E. Assam, leg. M. Ogle, NHMUK 1903.7.1.2532 (17 syntypes).

##### Etymology.

The replacement name *rotundus* (Latin for spherical, globular, round, circular) refers to the shell shape of this subspecies.

##### Remarks.

Protoconch matte; R1 with irregular, low riblets and somewhat weaker spiral striation; R2 ca. 90° in length, smooth, with alternating lighter/slimmer and darker/thicker stripes.

Alycaeus (Dioryx) urnula var. globosus Godwin-Austen, 1914 is a junior primary homonym of *Alycaeus globosus* H. Adams 1870. Here we propose *rotundus* as a replacement name for the junior homonym.

#### Dioryx urnula pisum

Taxon classificationAnimaliaGastropodaCyclophoridae

(Godwin-Austen, 1914)

7FD7C8BB-FB8D-592C-B369-69BDB6DB807C

Alycaeus (Dioryx) urnula var. pisum Godwin-Austen, 1914: 384, 402, pl. 153, figs 3, 3a.
Dioryx urnula var. pisum – Gude 1921: 203.

##### Type locality.

“Nongjinghi Trigonometrical Station, 4563 feet, Jaintia Hills”.

##### Material examined.

Nongjinghi, Jaintia, NHMUK 1903.7.1.2526 (11 syntypes).

##### Remarks.

The whole shell is nearly smooth; protoconch matte; R1 with irregular, very fine growth lines; R2 moderately long (ca. 90°), it has alternating slimmer/lighter and thicker/darker stripes.

#### Genus Metalycaeus

Taxon classificationAnimaliaGastropodaCyclophoridae

Pilsbry, 1900

584E5BE3-9FB1-5B90-9DD2-7F27B311041E

Alycaeus (Metalycaeus) Pilsbry, 1900: 382. (section of Alycaeus)
Raptomphalus Godwin-Austen, 1914: 366. syn. nov.
Metalycaeus – Bollinger 1918: 316; Páll-Gergely and Asami 2017: 4; Páll-Gergely et al. 2017: 73.
Chamalycaeus (Metalycaeus) – Thiele 1929: 108; Wenz 1938: 478; Egorov 2013: 37.
Chamalycaeus (Raptomphalus) – Thiele 1929: 108; Wenz 1938: 478; Egorov 2013: 37–38.

##### Type species.

**Figure 31.**
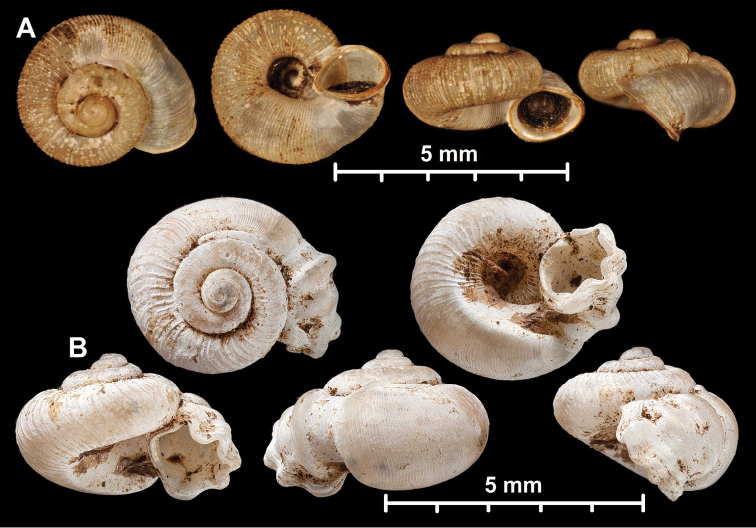
Type species of alycaeid genus-group taxa **A***Alycaeus melanopoma* Pilsbry, 1900 (synonym of *Chamalycaeus nipponensis* (Reinhardt, 1877)), lectotype (ANSP 78815; type species of *Metalycaeus*); **B***Metalycaeus magnificus* (Godwin-Austen, 1914), syntype (NHMUK 1903.7.1.3115; type species of *Raptomphalus*). Photographs: downloaded from the website of ANSP (**A**), Harold Taylor (**B**).

Alycaeus (Metalycaeus) melanopoma Pilsbry, 1900 (Fig. 31A) (junior synonym of *Chamalycaeus nipponensis* [Reinhardt, 1877], see Minato 1988), SD Thiele (1929: 108).

##### Diagnosis.

Shell small to very large (D: 3–10 mm), spire usually low, protoconch usually elevated, spirally striated (key character); spiral striation of protoconch very rarely absent (see under *M. laevis*); R1 usually reticulated with sometimes prominent radial ribs; spiral lines always present on the teleoconch; R2 rarely short, usually long to very long, sometimes entirely smooth, but typically with widely spaced, straight, sharp ribs; R3 usually well-developed, although can be reduced. Operculum thin or relatively thickened, sometimes with funnel-shaped outer surface caused by the modification of the multispiral outer laminae. Radula usual for the family (central tooth with five cusps, broad, central cusp pointed).

##### Differential diagnosis.

*Metalycaeus* is recognised based on the spirally striated protoconch, which distinguishes *Metalycaeus* from species assigned to all other alycaeid genera.

##### Distribution.

*Metalycaeus* is widely distributed from the southeastern Himalaya to the south of Honshu Island (Japan) and the northern islands of the Philippines, but it does not extend any further south than northern Laos and Vietnam (Fig. 9).

The *Metalycaeus* records in Páll-Gergely and Auffenberg (2019) from Borneo (*Chamalycaeus everetti*) and Sumatra (*Chamalycaeus sumatranus*) later proved to be *Chamalycaeus* species.

##### Remarks.

The type species of *Raptomphalus* (*Metalycaeus magnificus*) (Fig. 31B) and *M. oakesi* have a prominently keeled umbilicus which serve as the distinctive character for the genus *Raptomphalus*. A less prominently keeled umbilicus is observable in other species, such as *Chamalycaeus vulcani* and *Metalycaeus brahma*, which can be interpreted as intermediate forms between the non-keeled umbilicus of alycaeids and the keeled umbilicus of *Raptomphalus*. Moreover, a variety of *Metalycaeus brahma* has much less prominent umbilical keel than typical species in the genus. Therefore, the keeled umbilicus cannot serve as a distinctive character between genera. Consequently, *Raptomphalus* is a synonym of *Metalycaeus*.

For sake of simplicity, this genus is divided here into typical and atypical (or questionable) species. The first includes those having sharp, widely spaced R2 ribs, whereas the second includes species with R2 sculpture other than typical, including those which could not be examined.

#### Typical *Metalycaeus*

##### Metalycaeus beddomei

Taxon classificationAnimaliaGastropodaCyclophoridae

(Godwin-Austen, 1914)

7E1D7FAD-3595-50D1-9383-AAB355F8129C

Alycaeus beddomei Godwin-Austen, 1914: 386, pl. 149, figs 5, 5a.
Alycaeus beddomei – Gude 1921: 205–206; Ramakrishna and Mitra 2002: 21.
Alycaeus (Alycaeus) beddomei – Ramakrishna et al. 2010: 46.

###### Type locality.

“Naga Hills”.

###### Material examined.

Naga Hills, coll. Beddome, NHMUK 1912.4.16.294 (5 syntypes).

###### Remarks.

Protoconch elevated, spirally striated; R1 spirally striated; R2 long, with widely spaced, sharp ribs.

##### Metalycaeus bhutanensis

Taxon classificationAnimaliaGastropodaCyclophoridae

(Godwin-Austen, 1914)

A3C4E406-37C4-5410-97A2-F790BC018502

Alycaeus bhutanensis Godwin-Austen, 1914: 350, pl. 148, fig. 8.
Alycaeus (Chamalycaeus) bhutanensis – Gude 1921: 224.

###### Type locality.

“Bhutan Frontier, probably on Eastern, or Aka Hills, side”.

###### Material examined.

Bhutan, coll. Beddome, NHMUK 1912.4.16.290 (2 syntypes).

###### Remarks.

Spire low, protoconch elevated, spirally striated; R1 spirally striated; R2 ong, with widely spaced, sharp ribs.

##### Metalycaeus brahma

Taxon classificationAnimaliaGastropodaCyclophoridae

(Godwin-Austen, 1886)

D1C44C4A-EDD2-585D-AA2D-E67179253877

Fig. 32

Alycaeus brahma Godwin-Austen, 1886: 195–196, pl. 48, figs 3.
Alycaeus (Chamalycaeus) brahma – Kobelt 1902: 353.
Alycaeus commutatus Godwin-Austen, 1914: 351, pl. 148, fig. 7. syn. nov.
Alycaeus brahma – Godwin-Austen 1914, Vol. II: 363.
Alycaeus brahma var. – Godwin-Austen 1914, Vol. II: 363.
Alycaeus chanjukensis Godwin-Austen, 1914: 364–365, pl. 157, figs 5, 5a. syn. nov.
Alycaeus chanjukensis – Gude 1921: 207.
Alycaeus (Chamalycaeus) brahma – Gude 1921: 224–225.
Alycaeus (Raptomphalus) commutatus – Gude 1921: 286.
Alycaeus brahma – Ramakrishna and Mitra 2002: 22.
Alycaeus (Alycaeus) chanjukensis – Ramakrishna et al. 2010: 47.
Chamalycaeus (Chamalycaeus) brahma – Ramakrishna et al. 2010: 52; Tripathy et al. 2018: 789.
Alycaeus (Alycaeus) chanjukensis – Tripathy et al. 2018: 789.

###### Type locality.

“Brahmakund” (*brahma*); “Chanjuk La, Tsanspu Valley, 4300 ft., Lat. 29°25', Long. 95°20'” (*chanjukensis*); “Bhutan” (*commutatus*).

###### Material examined.

Brahmakund, E. Assam, leg. M. Ogle, NHMUK 1903.7.1.2610 (16 syntypes of *A. brahma*); Dihung valley, Singpho Hills, leg. M. Ogle, NHMUK 1903.7.1.2611 (1 specimen of “*Metalycaeus brahma* (Godwin-Austen, 1886) var.”); Chanjuk La, Tsanspu valley, Lat. 29°25', Long. 95°20’, coll. Oakes, NHMUK 1903.7.1.3583 (3 syntypes of *A. chanjukensis*); Bhutan, ex Beddome coll., NHMUK 1912.4.16.293 (2 syntypes of *A. commutatus*).

###### Remarks.

Protoconch elevated, spirally striated; R1 spirally striated; R2 very long, ribs on R2 sharp and widely spaced.

The variety “*M. brahma* var.” has a much less prominent keel inside the umbilicus, which supports our view regarding the invalid status of *Raptomphalus*.

*Alycaeus commutatus* and *Alycaeus chanjukensis* do not differ from *M. brahma* in any important shell characters. The shell and aperture shapes, sculpture, and ratios of shell regions (R1 and R2) are identical. Thus, those two species are considered as synonyms of *M. brahma*.

**Figure 32.**
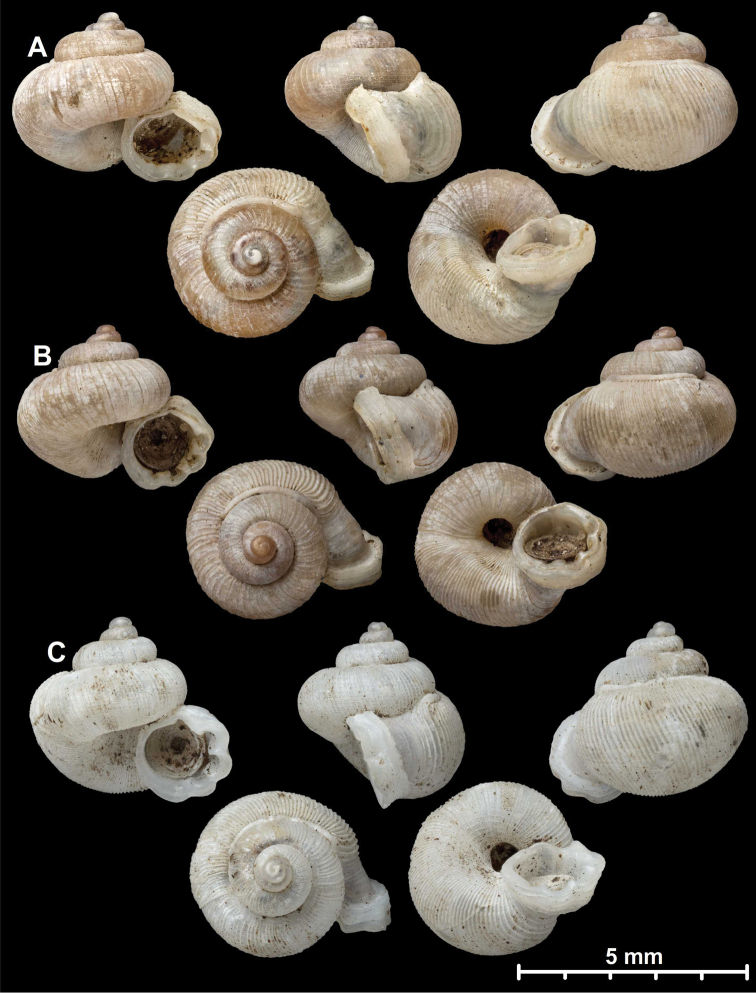
Shells of *Metalycaeus brahma* (Godwin-Austen, 1886) **A** syntype of *A. brahma* (NHMUK 1903.7.1.2610) **B** syntype of *A. commutatus* Godwin-Austen, 1914 (NHMUK 1912.4.16.293) **C** syntype of *A. chanjukensis* Godwin-Austen, 1914 (NHMUK 1903.7.1.3583). All photographs: Kevin Webb (NHM).

##### Metalycaeus burtii

Taxon classificationAnimaliaGastropodaCyclophoridae

(Godwin-Austen, 1874)

0387AB5D-E5C8-5DE5-B7F7-5B81338DBED8

Alycaeus Burtii Godwin-Austen, 1874: 149–150, pl. 3, fig. 9.
Alycaeus burtii – Reeve 1878: pl. 3, species 27; Godwin-Austen 1914: 352–353, pl. 144, figs 8, 8a.
Alycaeus (Alycaeus) burti [sic] – Kobelt 1902: 342; Ramakrishna et al. 2010: 47; Tripathy et al. 2018: 789.
Alycaeus burti [sic] – Gude 1921: 206–207.

###### Type locality.

“Foot of the Bhutan Himalaya at the debouchement of the Barowli River, Assam”.

###### Material examined.

Barowli Gorge, Tezpur Dist., Assam, NHMUK 1903.7.1.2492 (holotype [single specimen mentioned in the original description]).

###### Remarks.

Protoconch elevated, spirally striated; R1 irregularly ribbed, spirally striated; R2 relatively short, with sharp, straight, widely spaced ribs.

The original and correct spelling is *burtii*, and *burti* is an unjustified emendation.

##### Metalycaeus burtii yetayensis

Taxon classificationAnimaliaGastropodaCyclophoridae

(Godwin-Austen, 1914)

4EE3A776-D7E3-574C-9C40-665297AB19AD

Alycaeus burtii var. yetayensis Godwin-Austen, 1914: 353–354, pl. 149, fig. 7.
Alycaeus burtii var. yetaiensis [sic] – Gude 1921: 207.

###### Type locality.

“Yetay Ravine, No. 24 Peak, Dafla Hills”.

###### Material examined.

Yetay ravine, No. 24 Pk, Daflam No. 7 camp, NHMUK 1903.7.1.2535 (6 syntypes).

###### Remarks.

The traits examined for correct generic placement are identical with those of the nominotypical subspecies.

##### Metalycaeus caroli

Taxon classificationAnimaliaGastropodaCyclophoridae

(Semper, 1862)

F74CEEA3-184B-5F62-809E-383610371F19

Alycaeus caroli Semper, 1862: 148–149.
Alycaeus (Chamalycaeus) caroli – Kobelt 1902: 353–354.
Chamalycaeus (Chamalycaeus) caroli – Zilch 1957: 142, pl. 5, fig. 4.
Metalycaeus caroli – Páll-Gergely and Auffenberg 2019: 384, fig. 7A, B.

###### Type locality.

“ad Digallorin vicum insulæ Luzon”.

###### Material examined.

Philippines (N-Luzon), ‘ad Digallorin vicum insulæ Luzon’ (Digollorin Bay: 16°50'N, 122°26'E), coll. C. Semper, 18 August 1860, SMF 158415 (2 syntypes); Luzon, Philippines, V W. MacAndrew Collection, Acc. no. 1563, NHMUK 20150120 (2 shells).

###### Remarks.

Protoconch elevated, spirally striated; R1 with irregular ribs and spiral striae of ca. the same strength, or slightly even stronger than the ribs; R2 relatively short, with alternating darker/wider and slimmer/lighter stripes; its surface is nearly smooth.

##### Metalycaeus crenulatus

Taxon classificationAnimaliaGastropodaCyclophoridae

(Benson, 1859)

AF0EA8B1-D3BF-5159-8266-741D517167AE

Alycaeus crenulatus Benson, 1859: 180–181.
Alycaeus crenulatus – Reeve 1878: pl. 5, species 43; Godwin-Austen 1871: 90, pl. 3, fig. 4; Godwin-Austen 1914: 337–338, pl. 133, figs 1, 1a–c.
Alycaeus (Dicharax) crenulatus – Kobelt 1902: 367; Gude 1921: 242.
Chamalycaeus (Dicharax) crenulatus – Ramakrishna et al. 2010: 57; Tripathy et al. 2018: 789.

###### Type locality.

“in valle Rungun”.

###### Material examined.

Damsang, W. Bhutan, leg. Robert, NHMUK 1903.7.1.1254 (7 shells).

###### Remarks.

Protoconch elevated, spirally striated; R1 with rather regular ribs and weaker spiral striae; R2 short with widely spaced, high, sharp ribs.

##### Metalycaeus cyphogyrus

Taxon classificationAnimaliaGastropodaCyclophoridae

(Quadras & Möllendorff, 1895)

D6E55AEC-6699-507F-AD51-DAF0E0ADFD7B

Alycaeus cyphogyrus Quadras & Möllendorff, 1895: 144–145.
Alycaeus (Chamalycaeus) cyphogyrus – Kobelt 1902: 354.
Chamalycaeus (Chamalycaeus) cyphogyrus – Zilch 1957: 142, pl. 6, fig. 17.
Metalycaeus cyphogyrus – Páll-Gergely and Auffenberg 2019: 384, fig. 8A.

###### Type locality.

“In insula Catanduanes leg. J. Quadras, prope vicum Caramuan insulae Luzon”.

###### Material examined.

Philippinen: Caramuan (Luzon, Camarines), coll. Möllendorff, SMF 109473 (lectotype, designated by Zilch 1957); Philippinen: Catanduanes, SMF 109475 (8 paralectotypes); Catanduanes, Philippines, ‘S&F Ad. 31/10/07, da Costa Collection’, V. W. MacAndrew Collection, Acc. no. 1563, NHMUK 20150119 (3 shells).

###### Remarks.

Protoconch elevated, spirally striated; R1 with dense, weak ribs and spiral striae of the same strength; R2 moderately long, with relatively dense and low ribs.

##### Metalycaeus distinctus

Taxon classificationAnimaliaGastropodaCyclophoridae

(Godwin-Austen, 1893)

D8D5CB61-AE12-5B37-BAE0-C4B14C9512AF

Alycaeus ingrami var. – Godwin-Austen 1871: 91–92, pl. 4, fig. 3.
Alycaeus distinctus Godwin-Austen, 1893: 592.
Alycaeus distinctus – Godwin-Austen 1914: 363; Godwin-Austen 1914: 390, pl. 145, figs 3, 3a, b.
Alycaeus distinctus var. – Godwin-Austen 1914: 363, pl. 137, figs 2, 2a, 2b.
Alycaeus distinctus var. – Godwin-Austen 1914: 391, pl. 149, fig. 4. (2 varieties)
Alycaeus (Chamalycaeus) distinctus – Gude 1921: 226–227.
Chamalycaeus (Chamalycaeus) distinctus – Ramakrishna et al. 2010: 53.

###### Type locality.

“Neighbourhood of Asálú, N. Cachar Hills”.

###### Material examined.

Naga Hills, NHMUK 1903.7.1.2619 (10 syntypes); Sadia, Assam, NHMUK 1903.7.1.2620 (2 shells of “*Alycaeus distinctus* var.”); Jatinga Valley, N. Cachar, coll. Godwin-Austen, NHMUK 1903.7.1.2576 (19 + 6 shells) (“*Alycaeus distinctus* var.”).

###### Remarks.

Protoconch elevated, spirally striated; R1 finely, irregularly ribbed with equally strong spiral striae; R2 short, with sharp, straight, widely spaced ribs.

##### Metalycaeus godwinausteni

Taxon classificationAnimaliaGastropodaCyclophoridae

Páll-Gergely nom. nov.

8381F006-B133-52FB-8B95-8D475BB2F983

Alycaeus neglectus Godwin-Austen, 1914: 358, pl. 154, fig. 5. (non Alycaeus neglectus Heude, 1885)
Alycaeus (Dicharax) neglectus – Gude 1921: 261.
Chamalycaeus (Dicharax) neglectus – Ramakrishna et al. 2010: 64.

###### Type locality.

“Torúpútú Peak, Dafla Hills”.

###### Material examined.

Toruputu Peak, Dafla Hills, leg. Godwin-Austen, NHMUK 1903.7.1.2494 (2 syntypes).

###### Etymology.

We dedicate this species to H. H. Godwin-Austen (1834–1923), who described the majority of the alycaeid species in the Himalaya region.

###### Remarks.

Protoconch elevated, spirally striated; R1 rather glossy, but very fine reticulated (ribs and spiral striation or roughly equal strength); R2 short, with widely spaced, sharp ribs.

*Alycaeus neglectus* Heude, 1885 belongs to the genus *Metalycaeus* due to the spirally striated protoconch. This is the case even if it is treated here as a junior synonym of *Metalycaeus rathouisianus* (Heude, 1882). Thus, *A. neglectus* Godwin-Austen, 1914 is a primary as well as a secondary junior homonym of *Alycaeus neglectus* Heude, 1885 and thus, a replacement name (*godwinausteni*) must be given.

##### Metalycaeus heudei

Taxon classificationAnimaliaGastropodaCyclophoridae

(Bavay & Dautzenberg, 1900)

4019707A-3511-5C8F-9AD3-AA187F334F5D

Alycaeus (Charax) heudei Bavay & Dautzenberg, 1900a: 121–122.
Alycaeus (Charax) heudei – Bavay & Dautzenberg, 1900b: 458–459, pl. 11, figs 15–18.
Alycaeus (Dicharax) heudei – Kobelt 1902: 372.
Alycaeus paviei Bavay & Dautzenberg, 1912: 50–51, pl. 4, figs 5–8.
Alycaeus paviei var. minor Bavay & Dautzenberg, 1912: 51, pl. 4, fig. 9.
Chamalycaeus (Dicharax) compressicosta Zilch, 1957: 145–146, fig. 33.
Chamalycaeus (Dicharax) fractus Varga, 1974: 165–167, figs 1–5.
Alycaeus zhuangiyucuii Yang, Fan, Qiao & He, 2012: 32, fig. 2.
Metalycaeus heudei – Páll-Gergely et al. 2017: 74–84, figs 49C, D, 50–52, 53C, D; Inkhavilay et al. 2019: 16, fig. 6C.

###### Type locality.

“Haut-Tonkin”.

###### Material examined.

Haut Tonkin, leg. Messager, MNHN-IM-2000-27169 (1 syntype).

###### Remarks.

*Alycaeus paviei* Bavay & Dautzenberg, 1912, Alycaeus paviei var. minor Bavay & Dautzenberg, 1912, Chamalycaeus (Dicharax) compressicosta Zilch, 1957, Chamalycaeus (Dicharax) fractus Varga, 1974 and *Alycaeus zhuangiyucuii* Yang, Fan, Qiao & He, 2012 are synonyms of *Metalycaeus heudei* (see Páll-Gergely et al. 2017).

Protoconch elevated, spirally striated, R1 regularly ribbed, area between ribs with very fine spiral structure; R2 with sharp, widely spaced ribs.

##### Metalycaeus hirasei

Taxon classificationAnimaliaGastropodaCyclophoridae

(Pilsbry, 1900)

C4D263C0-2A5D-5109-B26D-A3B9DF036416

Alycaeus hirasei Pilsbry, 1900: 382.
Alycaeus (Metalycaeus) hirasei – Kobelt 1902: 378.
Chamalycaeus hirasei – Azuma 1982: 11, pl. 4, fig. 37; Minato 1988: 13–14.
Chamalycaeus (Metalycaeus) hirasei – Egorov 2013: 37, fig. 67c.
Metalycaeus hirasei – Páll-Gergely et al. 2017: 103, 104; Páll-Gergely and Asami 2017: 4.

###### Type locality.

“Kioto”.

###### Material examined.

Kioto, Japan, leg. Hirase, 1900, ANSP 78847 (lectotype designated by Baker, 1964, photographs examined).

###### Remarks.

There are clearly visible spiral striae on the protoconch and on R1.

##### Metalycaeus hungerfordianus

Taxon classificationAnimaliaGastropodaCyclophoridae

(Nevill, 1881)

9A6083AB-779E-5872-81DD-7362D12C35A3

Alycaeus hungerfordianus Nevill, 1881: 149–150.
Alycaeus (Chamalycaeus) hungerfordianus – Kobelt 1902: 356.
Chamalycaeus (Chamalycaeus) hungerfordianus – Zilch 1957: 142.
Chamalycaeus hungerfordianus – Hsieh et al. 2006: 86 + text figs; Hwang 2014: 7, fig. 1E.

###### Type locality.

“Tamsui”.

###### Material examined.

Formosa, leg. Hungerford, NHMUK 1891.3.17.790–791 (probable syntypes, see Hwang 2014).

###### Remarks.

Protoconch elevated, spirally striated; R1 very finely, irregularly ribbed, and striated with equally strong striae; R2 very short, consists of less than 10, sharp, widely spaced, rather irregular ribs which are slightly bent in the direction of their anterior neighbours.

##### Metalycaeus inflatus

Taxon classificationAnimaliaGastropodaCyclophoridae

(Godwin-Austen, 1874)

B6715413-F59A-5617-B2F2-31066BE12613

Alycaeus inflatus Godwin-Austen 1874: 146, pl. 3, fig. 1.
Alycaeus (Chamalycaeus) inflatus – Kobelt 1902: 356–357; Gude 1921: 227–228.
Alycaeus inflatus – Godwin-Austen 1914: 392–393, pl. 144, figs 1b–d.
Alycaeus inflatus var. – Godwin-Austen 1914: 394, pl. 144, figs 1, 1a.
Chamalycaeus (Chamalycaeus) inflatus – Ramakrishna et al. 2010: 53.

###### Type locality.

“Naga Hills under Japvo Peak”; “Yémi, Phúnngum, and Gaziphimi at the head of the Lanier River”.

###### Material examined.

Japvo Peak, 10000, Naga Hills, NHMUK 1903.7.1.2536 (3 syntypes in 2 vials).

###### Remarks.

Protoconch elevated, spirally striated; R1 with very weak, irregular ribs, and approximately as strong spiral lines; R2 relatively long, with sharp, straight, widely spaced ribs.

##### Metalycaeus kamakiaensis

Taxon classificationAnimaliaGastropodaCyclophoridae

(Godwin-Austen, 1914)

728E54D6-63F5-571B-8D18-BD929AFC6F77

Alycaeus kamakiaensis Godwin-Austen, 1914: 375–376, pl. 141, fig. 8.
Alycaeus kamakiaensis – Gude 1921: 209.
Alycaeus (Alycaeus) kamakiaensis – Ramakrishna et al. 2010: 48.

###### Type locality.

“Kamakia Temple Hill near Gowhatty, Assam”.

###### Material examined.

Kamakia Hill, Gowhatty, Assam, NHMUK 1903.7.1.2705 (1 syntype).

###### Remarks.

Protoconch elevated, spirally striated; R1 spirally striated; R3 moderately long, ribs are sharp and widely spaced.

##### Metalycaeus kengtungensis

Taxon classificationAnimaliaGastropodaCyclophoridae

(Godwin-Austen, 1914)

346A15A5-0655-535C-9DD7-7C4B05C526C7

Alycaeus kengtungensis Godwin-Austen, 1914: 409, pl. 139, figs 6, 6a.
Alycaeus (Raptomphalus) kengtungensis – Gude 1921: 287.

###### Type locality.

“Kengtung, Shan Frontier”.

###### Material examined.

Kengtung, Siam Frontier, leg. Woodthorpe, NHMUK 1903.7.1.3037 (holotype [single specimen mentioned in the original description]).

###### Remarks.

The whole shell is weathered; therefore, the sculpture could only be examined in very small regions of the shell. Protoconch elevated, some slight signs of spiral striation are visible; R1 regularly ribbed, spiral lines are not visible; R2 very long, the ribs are very widely spaced and sharp.

##### Metalycaeus laosensis

Taxon classificationAnimaliaGastropodaCyclophoridae

Páll-Gergely, 2017

14C9D465-7B10-54FE-A918-6BDB4784328A

Metalycaeus laosensis Páll-Gergely in Páll-Gergely et al., 2017: 86–87, fig. 47C.
Metalycaeus laosensis – Inkhavilay et al. 2019: 16, fig. 6D.

###### Type locality.

“Northern Laos, Phongsaly Province, old forest near stream approx. 1 km SW of a stream and Nam Ou (river) confluence, 493 m, 21°44.663'N, 102°10.999'E”.

###### Material examined.

Holotype (MNHN IM-2012-27172) and paratypes, see Páll-Gergely et al. (2017).

###### Remarks.

Protoconch elevated, spirally striated, R1 with irregular, low, widely spaced ribs and fine spiral striation; R2 short, regularly, densely ribbed, ribs rather straight in posterior part of region, ribs gradually curved towards aperture.

##### Metalycaeus latecostatus

Taxon classificationAnimaliaGastropodaCyclophoridae

(Möllendorff, 1882)

01B270F7-E744-5173-A787-DCE350500C1B

Alycaeus latecostatus – Möllendorff, 1882: 346, pl. 10, fig. 7.
Alycaeus (Orthalycaeus) latecostatus – Möllendorff 1886: 171.
Alycaeus (Chamalycaeus) latecostatus – Kobelt and Möllendorff 1897: 148; Kobelt 1902: 358.
Chamalycaeus latecostatus – Yen 1939: 29–30, pl. 2, fig. 34.
Chamalycaeus (Chamalycaeus) latecostatus – Zilch 1957: 143, pl. 5, fig. 11.
Metalycaeus latecostatus – Páll-Gergely et al. 2017: 88, fig. 47B.

###### Type locality.

“Bei Lien-tschou auf Tropfsteinfelsen”.

###### Material examined.

China: Lo-fou-schan (Kwangtung), leg. Möllendorff, coll. Kobelt, SMF 39215 (lectotype, designated by Yen 1939); Same data, SMF 39299 (2 paralectotypes); Same data, SMF 39216 (8 shells).

###### Remarks.

Protoconch elevated, spirally striated; R1 with very strong ribs with very weak spiral lines between them; R2 short, with widely spaced, sharp ribs, which are usually slightly bent in the direction of their anterior neighbours.

##### Metalycaeus lohitensis

Taxon classificationAnimaliaGastropodaCyclophoridae

(Godwin-Austen, 1914)

D5FE8F67-D3A0-52B2-B859-7C746CEF5A9C

Alycaeus lohitensis Godwin-Austen, 1914: 362–363, pl. 137, figs 1, 1a.
Alycaeus lohitensis – Gude 1921: 210.
Alycaeus (Alycaeus) lohitensis – Ramakrishna et al. 2010: 48; Tripathy et al. 2018: 789.

###### Type locality.

“Brahmakund, Eastern Assam”.

###### Material examined.

Brahmakund, Eastern Assam, leg. Ogle, NHMUK 1903.7.1.2493 (19 syntypes); Brahmakund, E. R. Sykes Collection, Acc. no. 1825, NHMUK 20150125 (1 shell) (matches the description and the figure in the orig. description).

###### Remarks.

Protoconch elevated, spirally striated; R1 with rather regular, widely spaced ribs with somewhat weaker spiral striae between the ribs; R2 long, with sharp, widely spaced ribs.

##### Metalycaeus luyorensis

Taxon classificationAnimaliaGastropodaCyclophoridae

(Godwin-Austen, 1914)

BC6C6671-0C86-5263-BB6F-0C4E10D5A5B4

Alycaeus luyorensis Godwin-Austen, 1914: 365–366, pl. 157, figs 6, 6a.
Alycaeus (Raptomphalus) luyorensis – Gude 1921: 288.
Chamalycaeus (Raptomphalus) luyorensis – Ramakrishna et al. 2010: 69.

###### Type locality.

“Luyor, Abor Hills, 7200 ft”.

###### Material examined.

Luyor, Abor, 7200, leg. Oakes, NHMUK 1903.7.1.3527 (2 yntypes).

###### Remarks.

Both shells were somewhat weathered. Protoconch elevated, spirally striated; R1 spirally striated; R2 relatively long, with dense, low ribs; they might be sharper in fresh shells.

##### Metalycaeus macgregori

Taxon classificationAnimaliaGastropodaCyclophoridae

(Godwin-Austen, 1914)

B776AC86-9F45-5EF7-B427-95E26219E1EF

Alycaeus macgregori Godwin-Austen, 1914: 356–357, pl. 141, figs 2, 2a, 2b.
Alycaeus (Chamalycaeus) macgregori – Gude 1921: 229.
Chamalycaeus (Chamalycaeus) macgregori – Ramakrishna et al. 2010: 54; Tripathy et al. 2018: 789.

###### Type locality.

“Dafla Hills”.

###### Material examined.

Dafla Hills, NHMUK 1903.7.1.2521 (holotype [fixed by original designation]); Shengorh, Dafla Hills, coll. Godwin-Austen, NZSI M.8058 (2 shells, labelled as co-type = paratypes).

###### Remarks.

Protoconch elevated, spirally striated; R1 finely ribbed and spirally striated; R2 relatively short with widely spaced ribs, but the fine structure of the ribs was not visible due to corrosion.

##### Metalycaeus muciferus

Taxon classificationAnimaliaGastropodaCyclophoridae

(Heude, 1885)

6D49DDFD-5AFB-5E42-97DB-32A82DBDCC8D

Fig. 33

Alycoeus [sic] muciferus Heude, 1885: 96, pl. 24, figs 1, 1a.
Alycaeus (Orthalycaeus) muciferus – Möllendorff 1886: 171.
Alycaeus helicodes Gredler, 1888: 365.
Alycaeus expansus Heude, 1890: 129, pl. 38, fig. 2. syn. nov.
Alycaeus (Chamalycaeus) muciferus – Kobelt and Möllendorff 1897: 149; Kobelt 1902: 359.
Metalycaeus muciferus – Páll-Gergely et al. 2017: 88–93, figs 58, 59, 60, 61 (helicodes Gredler is a synonym).
“*Chamalycaeus*” expansus – Páll-Gergely et al. 2017: 105, fig. 69L.

###### Type locality.

“in ditione Tchen-k’eou” (*Alycoeus muciferus*); Pe-shang, Prov. Hunan (*A. helicodes*); “Tchen K’eou” (*A. expansus*).

###### Material examined.

**Figure 33.**
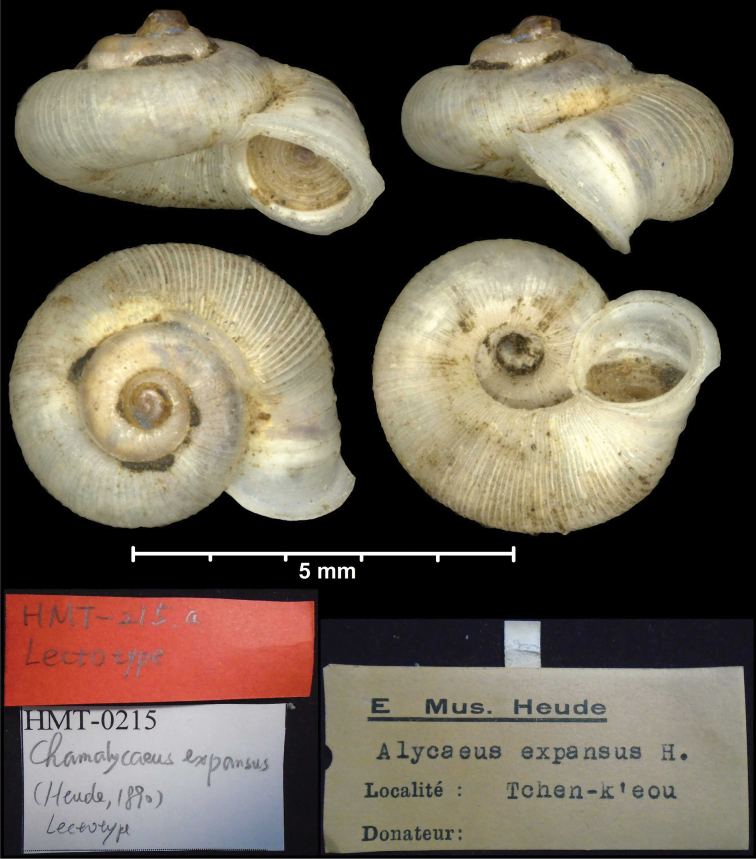
*Alycaeus expansus* Heude, 1890, syntype (HMT-215a; synonym of *Metalycaeus muciferus* (Heude, 1885)). Photographs: Kaibaryer Meng.

China, Tchen-k’eou, MCZ 167230 (7 syntypes of *A. muciferus*); Tchen-k’eou, China, Pe-shang, Hunan, leg. Fuchs, coll. Gredler, SMF 192225 (2 paralectotypes of *A. helicodes*, lectotype in the Franziskanergymnasium Bozen [Bolzano, Italy], designated by Zilch 1974); HMT-215a, syntype: labelled as lectotype of *A. expansus* deposited in IZCAS: Tchen-k’eou (Cheng-kou County, Chong-qing, China) (Fig. 33).

###### Remarks.

Protoconch conspicuously elevated from dorsal surface, its end with inconspicuous spiral striations; R1 with irregular, widely spaced ribs and fine spiral sculpture; R2 with dense, sharp, elevated ribs.

No type specimens of *Alycaeus expansus* were reported in American museums (Johnson 1973). We were not able to examine the types deposited in the Beijing Museum before the revision of Chinese Alycaeidae (Páll-Gergely et al. 2017), but could do so now. The general shell shape, colour, sculpture, and the lengths and proportions of R2 and R3 agree with those of *A. muciferus*, which was described from the same type locality. Consequently, we move *Alycaeus expansus* to the synonymy of *A. muciferus*.

##### Metalycaeus mundulus

Taxon classificationAnimaliaGastropodaCyclophoridae

(Godwin-Austen, 1914)

1446E8DD-A9F0-5CFC-88C5-3C5D2CA3A25D

Alycaeus mundulus Godwin-Austen, 1914: 357–358, pl. 149, fig. 8.
Alycaeus mundulus – Gude 1921: 212.
Alycaeus (Alycaeus) mundulus – Ramakrishna et al. 2010: 49; Tripathy et al. 2018: 789.

###### Type locality.

“Torúpútú, Dafla Hills”.

###### Material examined.

Toruputu, Dafla Hills, leg. Godwin-Austen, NHMUK 1906.1.1.955 (holotype [single specimen mentioned in the original description]).

###### Remarks.

The entire shell was weathered, but the following information could be obtained: protoconch strongly elevated, some spiral striation visible near suture (all other parts are strongly weathered); R1 with regular, strong ribs and spiral striae; R2 moderately long, rib structure could not be observed.

##### Metalycaeus nipponensis

Taxon classificationAnimaliaGastropodaCyclophoridae

(Reinhardt, 1877)

DBAF3D82-E6E1-599E-B088-F873F51AD93A

Fig. 31A

Alycaeus Nipponensis Reinhardt, 1877: 68.
Alycaeus nipponensis – Pilsbry 1900: 381.
Alycaeus melanopoma Pilsbry, 1900: 382.
Alycaeus (Chamalycaeus) nipponensis – Kobelt 1902: 360.
Alycaeus (Metalycaeus) melanopoma – Kobelt 1902: 378.
Chamalycaeus (Chamalycaeus) nipponensis – Zilch 1957: 144, pl. 5, fig. 16.
Chamalycaeus nipponensis – Azuma 1982: 11, pl. 4, fig. 35; Minato 1988: 13.
Chamalycaeus (Metalycaeus) nipponensis – Egorov 2013: 37, figs 67a, b.
Metalycaeus nipponensis – Páll-Gergely et al. 2017: 73, figs 46A–C; Páll-Gergely and Asami 2017: 4.

###### Type locality.

“Kioto”.

###### Material examined.

Hakone Mts, Japan, leg. Schmacker, ANSP 78815 (lectotype of *A. melanopoma*, designated by Baker 1964, photographs examined); Japan, Yedo, Doenitz l., coll. Reinhardt, SMF 185407 (labelled as ‘typus’); 東京都 (Tōkyō-to), 多摩市 (Tama-shi), 蓮光寺聖跡記念館 境内 (Renkō-ji Seiseki Kinenkan keidai) [Tama City, Tokyo Metropolitan Prefecture], NSMT-Mo 79102 (91 shells).

###### Remarks.

Protoconch elevated, its last 0.5 whorl with spiral lines; ribbing on R2 is quite variable; there are 14–22, usually elevated and distinct ribs. In some specimens the ribs are relatively irregular, and they seemingly join to each other near the tube. This is, however, rather due to the fact that the shells are weathered (despite the fact that they were collected alive), because the elevated ribs are destroyed and appear lower. R1 with clearly visible, fine spiral striation.

In the examined sample, 91 specimens had an operculum, and of those 53 of them had the outer black circle present. In case of 27 specimens the outer circle was entirely missing, and in 11 cases only small fragments of the rings remained.

##### Metalycaeus oakesi

Taxon classificationAnimaliaGastropodaCyclophoridae

(Godwin-Austen, 1914)

FBCABE51-5417-5222-B9C9-D888A2327069

Alycaeus oakesi Godwin-Austen, 1914: 366–367, pl. 157, figs 4, 4a.
Alycaeus (Raptomphalus) oakesi – Gude 1921: 289.
Chamalycaeus (Raptomphalus) oakesi – Ramakrishna et al. 2010: 69.

###### Type locality.

“Chanjuk La, in Tsanspu Valley 4300 ft., Lat. 29°25´, Long. 95°20´”.

###### Material examined.

Abor Hills, Lat. 29°25, Long. 95°20, NHMUK 1903.7.1.3578 (5 syntypes).

###### Remarks.

In the original description the subgeneric name *Raptomphalus* is not used. However, the species is introduced after Alycaeus (Raptomphalus) magnificus, and the characteristically keeled umbilicus is similar in both species.

All four shells were strongly weathered, but the characteristic elevated protoconch was recognisable. Moreover, some indication of spiral striation was visible on both the protoconch and R1, therefore the classification of *M. oakesi* in *Metalycaeus* is justified. R2 long, with widely spaced, sharp ribs.

##### Metalycaeus oharai

Taxon classificationAnimaliaGastropodaCyclophoridae

Páll-Gergely & Hunyadi, 2017

552F466C-74F3-5A1E-9C96-2CEBD35EC09E

Metalycaeus oharai Páll-Gergely & Hunyadi in Páll-Gergely et al., 2017: 93–96, figs 48C–F, 62A, B, 63C, D.

###### Type locality.

“China, Guangxi, Huanjiangmaonanzu Zizhixian, Dacai Xiang, Nongmaoshichang, 201 m, 24°45.566'N, 108°21.945'E”.

###### Material examined.

Holotype (HNHM 99712) and many other samples, see Páll-Gergely et al. (2017).

###### Remarks.

Protoconch elevated, finely spirally striated; R1 with sharp ribs and clearly visible, fine spiral lines resulting in a reticulated surface; R2 ribs ca. 2–3 × more densely arranged as in R1, ribs very sharp and high.

##### Metalycaeus physis

Taxon classificationAnimaliaGastropodaCyclophoridae

(Benson, 1859)

C4CA630A-81B5-5379-8556-8E0322A1F9A6

Alycaeus physis Benson, 1859: 179.
Alycaeus physis – Reeve 1878: pl. 6, species 51; Godwin-Austen 1914: 342, pl. 134, figs 1, 1a.
Alycaeus (Chamalycaeus) physis – Kobelt 1902: 361; Gude 1921: 231–232, fig. 34.
Alycaeus (Orthalycaeus) physis – Rees 1964: 64, pl. 5, fig. 27.
Chamalycaeus (Chamalycaeus) physis – Ramakrishna et al. 2010: 55.

###### Type locality.

“in valle Rungit (alt. 2000 ped.), prope Darjiling”.

###### Material examined.

Sikkim, UMZC I.102735 (1 syntype); Darjeeling, ‘Sow M/R 7/3/91, 626’, V W. MacAndrew Collection, Acc. no. 1563, NHMUK 20150124 (2 shells); Darjiling, NHMUK 1888.12.4.895–871 (3 shells).

###### Remarks.

Spire low, but protoconch elevated, spirally striated; R1 rather irregularly ribbed, with weak spiral lines; R2 long, with waved surface; there are light and dark stripes alternating.

##### Metalycaeus polygonoma

Taxon classificationAnimaliaGastropodaCyclophoridae

(W. T. Blanford, 1862)

9A78308B-B92B-50E9-9651-996E8F367495

Alycaeus polygonoma W. T. Blanford, 1862: 140–141.
Alycaeus polygonus [sic] – Reeve 1878: pl. 2, species 11.
Alycaeus (Dicharax) polygonoma – Kobelt 1902: 375; Gude 1921: 265–266.
Alycaeus polygonoma – Godwin-Austen 1914: 423, pl. 141, fig. 5.

###### Type locality.

“in montibus Arakanensibus”.

###### Material examined.

Tongoop Pass, Arakan Hills, NHMUK 1906.4.4.51 (2 syntypes in different vials).

###### Remarks.

Protoconch elevated, spirally striated; R1 with irregular, fine ribs and spiral lines of approximately the same strength; R2 long, with sharp, widely spaced ribs.

##### Metalycaeus prosectus

Taxon classificationAnimaliaGastropodaCyclophoridae

(Benson, 1857)

E175F738-BB97-5EE1-AEE3-2C0CB372FBA5

Alycaeus prosectus Benson, 1857: 203–204.
Alycaeus prosectus – Reeve 1878: pl. 6, species 49; Godwin-Austen 1914: 380–381, pl. 143, figs 1, 1a, 1b.
Alycaeus (Dicharax) prosectus – Kobelt 1902: 375–376; Gude 1921: 266–267.
Chamalycaeus (Dicharax) prosectus – Ramakrishna et al. 2010: 65.

###### Type locality.

“ad Teria Ghát”.

###### Material examined.

Khasi Hills, NHMUK 1906.4.4.63 (5 shells); UMZC I.102775 (4 syntypes); NHMUK 1888.12.4.873–875 (3 shells).

###### Remarks.

Protoconch elevated, spirally striated; R1 with irregular, low ribs and spiral striae of the same strength; R2 relatively long, with sharp, relatively widely spaced, straight ribs.

##### Metalycaeus quadrasi

Taxon classificationAnimaliaGastropodaCyclophoridae

(Möllendorff, 1895)

C118395E-6835-5CCB-A5C7-B124C1EF07A1

Alycaeus quadrasi Möllendorff in Quadras & Möllendorff, 1895: 83.
Alycaeus (Chamalycaeus) quadrasi – Kobelt 1902: 362.
Chamalycaeus (Chamalycaeus) quadrasi – Zilch 1957: 145, pl. 6, fig. 18.
Metalycaeus quadrasi – Páll-Gergely and Auffenberg 2019: 385, fig. 8B.

###### Type locality.

“prope vicum Buguey provinciae Cagayan”.

###### Material examined.

Philippinen, Buguey (Luzon, Cagayan), leg. Möllendorff, SMF 109515 (lectotype, designated by Zilch 1957); Same data, SMF 109516 (5 paralectotypes).

###### Remarks.

Protoconch elevated, spirally striated; R1 with extremely dense, fine ribs and spiral lines of approximately of the same strength; R2 relatively short, with widely spaced, sharp ribs.

##### Metalycaeus rathouisianus

Taxon classificationAnimaliaGastropodaCyclophoridae

(Heude, 1882)

5A497BF8-D0A5-5EF5-AD06-461C6BA4E177

Figs 34 , 35

Alycaeus rathouisianus Heude, 1882: 7, pl. 12, figs 12, 12a.
Alycaeus rathouisianus – Möllendorff 1882: 345.
Alycoeus [sic] neglectus Heude, 1885: 96, pl. 24, fig. 4. syn. nov.
Alycaeus (Orthalycaeus) rathouisianus – Möllendorff 1886: 169.
Alycaeus (Orthalycaeus) neglectus – Möllendorff 1886: 170.
Alycaeus (Chamalycaeus) neglectus – Kobelt and Möllendorff 1897: 149; Kobelt 1902: 360.
Alycaeus (Chamalycaeus) rathouisianus – Kobelt and Möllendorff 1897: 149; Kobelt 1902: 362.
Alycaeus dolomiticus Heude, 1890: 130, pl. 38, fig. 1.
Chamalycaeus rathouisianus – Yen 1939: 30, pl. 2, fig. 36.
Chamalycaeus (Chamalycaeus) rathouisianus – Zilch 1957: 145.
Metalycaeus rathouisianus – Páll-Gergely et al. 2017: 98–103, figs 55A–C, 63A, B, 65A, B.
Dicharax (?) neglectus – Páll-Gergely et al. 2017: 105–107, fig. 69K.

###### Type locality.

“E collibus juxta civitatem Song-kiang (松江), provinciæ Kiang-sou, ad montes districtus Tong-lieou, sed non ubique” (*Alycaeus rathouisianus*); “*ad rupestres colles civitatis Kien-té, provinciæ* Ngan-houé *legeram*” (*Alycaeus neglectus*); “Ad calcarios lapides juxtà civitatem *Tchen-kiang* (*Kiang-sou*)”.

###### Material examined.

**Figure 34.**
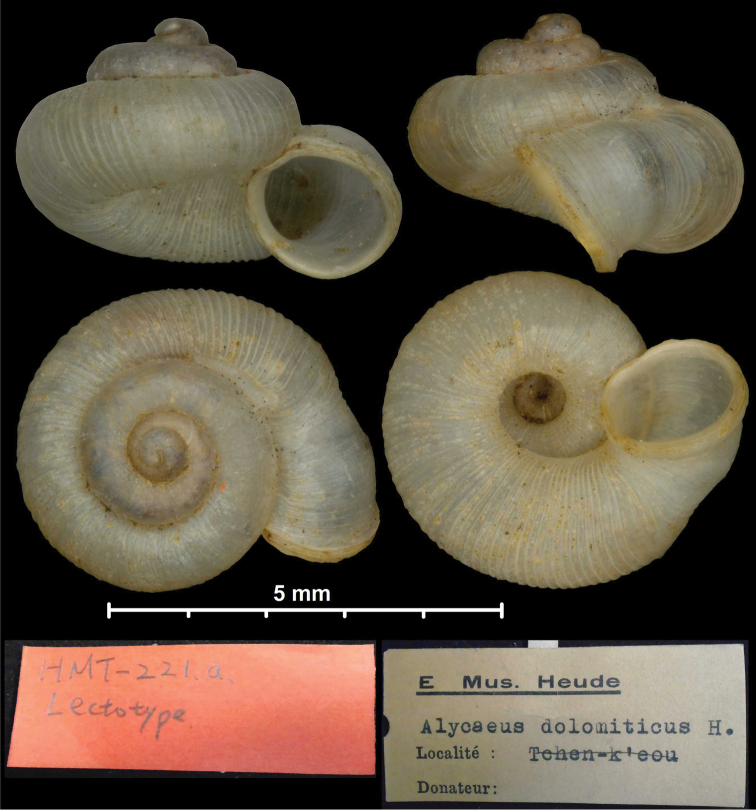
*Metalycaeus rathouisianus* (Heude, 1882), syntype: labelled as lectotype of *Alycaeus dolomiticus* (HMT-221a). Photographs: Kaibaryer Meng.

**Figure 35.**
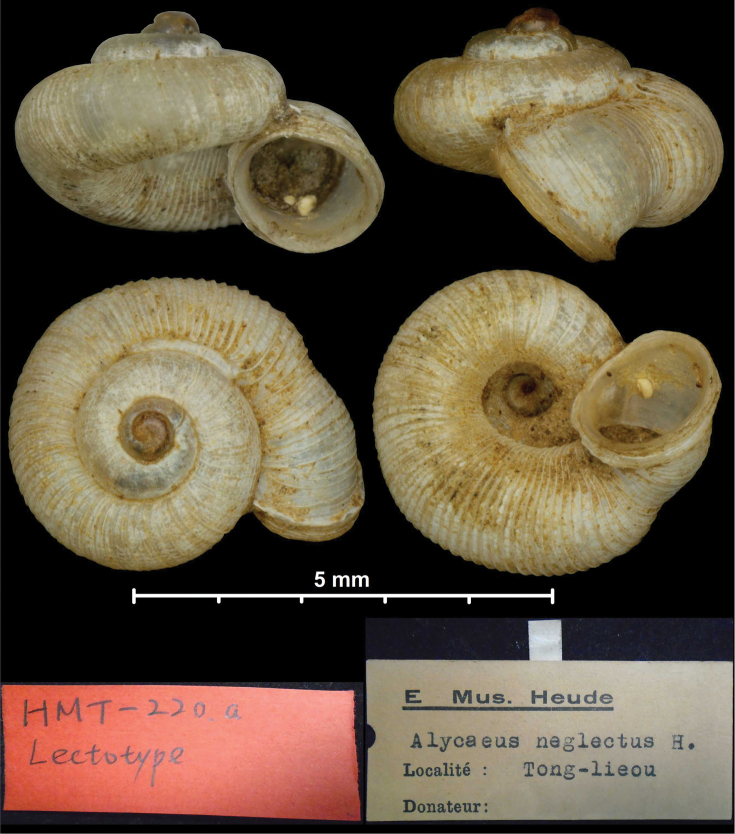
*Alycaeus neglectus* Heude, 1885, syntype (HMT-220a; synonym of *Metalycaeus rathouisianus* (Heude, 1882)). Photographs: Kaibaryer Meng.

China, Tsing-p’on, MCZ 167143 (23 syntypes of *A. rathouisianus*); Sung-kiang: China, coll. Möllendorff ex coll. Heude, SMF 39243 (5 syntypes of *A. rathouisianus*); same data, 39303 (3 syntypes of *A. rathouisianus*); China, Tchein-kiang (Kiang-sou), MCZ 167209 (29 syntypes of *A. dolomiticus*); HMT-221a (labelled as lectotype of *Alycaeus dolomiticus*, Fig. 34) deposited in IZCAS: locality information were deleted; HMT-221 (labelled as paralectotype of *Alycaeus dolomiticus*), same data with HMT-221; HMT-220a (1 syntype, labelled as lectotype of *Alycaeus neglectus*, Fig. 35), deposited in IZCAS: Tong-lieu (Dong-Liu town, Dong-Zhi County, Chi-Zhou City, An-Hui Province, China); Same data, HMT-220 (1 syntype, labelled as paralectotype of *Alycaeus neglectus*).

###### Remarks.

*Alycaeus dolomiticus* Heude, 1890 is a synonym of *Metalycaeus rathouisianus* (see Páll-Gergely et al. 2017).

According to Johnson (1973), paratypes of *A. neglectus* are present in the MCZ (inv. number: 167222) and the USNM (inventory number: 472336). In the revision of Chinese Alycaeidae (Páll-Gergely et al 2017) we examined the former sample, but the locality (“China, Tong-lieou” in Anhui Province) did not match the type locality given in the original description (“Kien-té” in Zhejiang Province). Consequently, we did not consider them as type specimens; moreover, on a hand-written label of the MCZ 167222 sample the following text was written: “not types, only holotype in Heude’s coll.”. We have now been able to examine the specimens in the Beijing museum, and found that the specimens labelled as types also have the same type locality (“Tong-lieu”). The Beijing specimens were also labelled as lectotype (HMT-221a), and paralectotypes (HMT-221). It appears that although the locality on the type specimen labels and that in the original description do not match, the shells labelled as types are indeed types of *A. neglectus*. Since we did not find traces of the lectotype designation, we consider all type specimens of *A. neglectus* syntypes.

Since we did not consider the specimens in the MCZ types in Páll-Gergely et al. (2017), we had to rely on the original description regarding the identity of *A. neglectus*, and based on the low protoconch in the figure of the original description, we tentatively classified it in *Dicharax*. However, this opinion must now be revised: the shells labelled as types in the Beijing Museum and in the MCZ are identical, and they are also identical to typical *Alycaeus rathouisianus* shells. Thus, *A. neglectus* is considered a synonym of *A. rathouisianus*.

##### Metalycaeus rotundatus

Taxon classificationAnimaliaGastropodaCyclophoridae

(Godwin-Austen, 1914)

6087B1CF-B2C5-51B9-967E-CE3971ABD08B

Alycaeus rotundatus Godwin-Austen, 1914: 359, pl. 154, fig. 6.
Alycaeus rotundatus – Gude 1921: 217.
Alycaeus (Alycaeus) rotundatus – Ramakrishna et al. 2010: 51.

###### Type locality.

“Dafla Hills”.

###### Material examined.

Dafla Hills. (?), NHMUK 1903.7.1.2543 (1 syntype).

###### Remarks.

Protoconch elevated, spirally striated; R1 irregularly wrinkled with spiral striation; R2 relatively short, with widely spaced, sharp ribs.

##### Metalycaeus rugosus

Taxon classificationAnimaliaGastropodaCyclophoridae

(Godwin-Austen, 1914)

3990EA5E-1934-5D1D-BA46-BA57A02046D2

Alycaeus rugosus Godwin-Austen, 1914: 359, pl. 141, figs 7, 7a.
Alycaeus (Dicharax) rugosus – Gude 1921: 268–269.
Chamalycaeus (Dicharax) rugosus – Ramakrishna et al. 2010: 66.

###### Type locality.

“Burroi Gorge, Dafla Hills”.

###### Material examined.

Burroi Gorge, Dafla, NHMUK 1903.7.1.2641 (3 syntypes, figured specimen indicated by pink wool).

###### Remarks.

All three shells were strongly weathered. Protoconch moderately elevated, with slight indication of spiral striae; R1 with regular, widely spaced ribs and very slight indication of spiral striae; R2 relatively short, with widely spaced, sharp ribs.

##### Metalycaeus satsumanus awaensis

Taxon classificationAnimaliaGastropodaCyclophoridae

(Pilsbry & Y. Hirase, 1904)

4B25B40B-E359-5AE7-A94E-2B14A24A0C74

Alycaeus awaensis Pilsbry & Y. Hirase, 1904a: 117.
Chamalycaeus awaensis – Azuma 1982: 11, pl. 3, fig. 34.
Chamalycaeus satsumanus awaensis – Minato 1988: 14.
Metalycaeus satsumanus awaensis – Páll-Gergely and Asami 2017: 4.

###### Type locality.

“Hiyama, Awa, Island of Shikoku”.

###### Material examined.

Hiyama, Awa, leg. Hirase, 1903, ANSP 84958 (lectotype, designated by Baker 1964, photographs examined).

###### Remarks.

Protoconch moderately elevated, very finely granulated, the last 0.5 whorl with fine spiral striae; R2 with 18–20 ribs; the ribs are widely spaced even near the tube; at the edge of the body whorl the gap between the ribs is ca. as wide as a rib itself; the first 0.75–1 whorl of R1 has clearly visible spiral striae.

##### Metalycaeus satsumanus laevicervix

Taxon classificationAnimaliaGastropodaCyclophoridae

(Pilsbry & Y. Hirase, 1904)

51FE7038-B691-5E5F-91F7-8BA11FBC7B61

Alycaeus laevicervix Pilsbry & Y. Hirase, 1904c: 618.
Chamalycaeus satsumanus laevicervix – Minato 1988: 14.
Metalycaeus satsumanus laevicervix – Páll-Gergely and Asami 2017: 4.

###### Type locality.

“Kuchinoerabushima, Ōsumi”.

###### Material examined.

Kuchinoerabushima, leg. Hirase, 1904, ANSP 87699 (1 syntype, photographs examined); label on the right: 大隅永良部嶋 (Osumi, Erabu-jima = Osumi, Erabu Island), label on the left: 大隅永良部島 is corrected by insertion as 大隅口之永良部島 (Osumi, Kuchinoerabu-jima = Osumi, Kuchinoerabu Island), Hirase coll., NSMT-Mo 2053 (3 shells).

###### Remarks.

Protoconch moderately elevated, rather roughly granulated, the granules are arranged into spiral lines on the last 0.25–0.5 of whorl; R2 with ca. 15 ribs.

##### Metalycaeus satsumanus

Taxon classificationAnimaliaGastropodaCyclophoridae

(Pilsbry, 1902)

54FA0DC3-6D30-555F-9F67-91FD28EC6D17

Alycaeus satsumana Pilsbry, 1902b: 548–549.
Chamalycaeus satsumanus – Azuma 1982: 10, pl. 3, fig. 31.
Chamalycaeus satsumanus satsumanus – Minato 1988: 14.
Metalycaeus satsumanus – Páll-Gergely et al. 2017: 103, 104; Páll-Gergely and Asami 2017: 4.

###### Type locality.

“Kagoshima, Satsuma, Kiusiu”.

###### Material examined.

Kagoshima, Satsuma, leg. Hirase, 1901, ANSP 81919 (lectotype, designated by Baker 1964, photographs examined); 鹿児島縣1鹿児島市原良田 (Kagoshima-ken, Kagoshima-shi, Harara, Ta[?] = Kagoshima Prefecture, Kagoshima City, Harara, Ta[?]), coll. Sakurai, NSMT-Mo 79103 (3 shells).

###### Remarks.

Protoconch elevated, with spiral lines on its last whorl; R2 with ca. 22 widely spaced, low, blunt, regular ribs; the distance between the ribs at the edge of the body whorl is at least 2–3 × wider than the width of a rib; R1 with conspicuous spiral striation.

*Alycaeus awaensis* Pilsbry & Y. Hirase, 1904 and *Alycaeus laevicervix* Pilsbry & Y. Hirase, 1904 are classified as a subspecies of *Chamalycaeus satsumanus* by Minato (1988), which we also follow here. *Sigmacharax tanegashimae* was additionally classified under *Chamalycaeus satsumanus* in the same study, but these species belong to different genera according to our revision.

##### Metalycaeus semperi

Taxon classificationAnimaliaGastropodaCyclophoridae

Páll-Gergely & Auffenberg, 2019

55E8E5DF-50EE-564A-8C1D-68FF30A3B471

Metalycaeus semperi Páll-Gergely & Auffenberg, 2019: 386, fig. 7C.

###### Type locality.

“Philippinen (Isabella): Luzon: Malunu”.

###### Material examined.

Holotype (SMF 349521) and paratypes (SMF 349522).

###### Remarks.

Protoconch elevated, finely spirally striated, consisting of ca. 1.5 whorls; R1 consisting of 1.75–2 whorls, very densely, finely ribbed and finely spirally striated, resulting in a fine, dense reticulated surface; R2 + R3 short, ca. 70–80° combined; R2 slightly shorter than R3; ribs on R2 more widely spaced and more elevated than those on R1, although still low; spiral striation also visible on R2 (Páll-Gergely and Auffenberg 2019).

##### Metalycaeus sinensis

Taxon classificationAnimaliaGastropodaCyclophoridae

(Heude, 1882)

99C4C0A4-C258-59FE-A6E9-823694F0FDEE

Alycaeus sinensis Heude, 1882: 7, pl. 12, figs 13, 13a.
Alycaeus sinensis – Möllendorff 1882: 345.
Alycaeus (Chamalycaeus) sinensis – Kobelt and Möllendorff 1897: 149; Kobelt 1902: 363.
Metalycaeus sinensis – Páll-Gergely et al. 2017: 103–104, fig 55.

###### Type locality.

“Ad radices saxorum inter folia decidua in districtu Tong-lieou (東流), provinciæ Ngan-houé”.

###### Material examined.

China, Tong-lieou, MCZ 167216 (lectotype, designated by Páll-Gergely et al. 2017).

###### Remarks.

Protoconch relatively low, with very fine matte surface and barely visible spiral lines; R1 with rather regular ribs and much weaker spiral lines between; ribs become more widely spaced towards the end of R1; R2 with relatively low, widely spaced ribs, however all available shells were weathered.

##### Metalycaeus stylifer

Taxon classificationAnimaliaGastropodaCyclophoridae

(Benson, 1857)

BBC2ED4F-F68C-5205-9CD0-3591F1F917F6

Alycaeus stylifer Benson, 1857: 204.
Alycaeus stylifer – Reeve 1878: pl. 6, species 46; Godwin-Austen 1914: 344, pl. 133, figs 2–2a–c.
Alycaeus (Dicharax) stylifer – Kobelt 1902: 376–377; Gude 1921: 269–270, fig. 35.
Chamalycaeus (Dicharax) stylifer – Ramakrishna et al. 2010: 66.

###### Type locality.

“ad Darjiling”.

###### Material examined.

Damsang, W. Bhutan, NHMUK 1903.7.1.1255 (1 shell); Darjeeling, UMZC I.102630 (possible holotype, photographs examined).

###### Remarks.

Protoconch elevated, spirally striated; R1 with irregular, weak ribs and spiral lines of comparable strength; R2 relatively short, with sharp, widely spaced ribs.

##### Metalycaeus subinflatus

Taxon classificationAnimaliaGastropodaCyclophoridae

(Godwin-Austen, 1914)

DCB01336-FFAD-5480-A31B-58CC9CB1667B

Alycaeus subinflatus Godwin-Austen, 1914: 400–401, pl. 154, figs 8, 8a.
Alycaeus (Chamalycaeus) subinflatus – Gude 1921: 235.
Chamalycaeus (Chamalycaeus) subinflatus – Ramakrishna et al. 2010: 56.

###### Type locality.

“Gaziphimi, Lahupa Naga Hills, N.E. Munipur”.

###### Material examined.

Gazifhimi, N.E. Manipur, leg. Godwin-Austen, NHMUK 1903.7.1.2489 (7 syntypes, one specimen indicated with pink wool, other shells in another glass vial).

###### Remarks.

Protoconch elevated, spirally striated; R1 with weak, irregular ribs and spiral striae of similar strength; R2 relatively long, with sharp, widely spaced ribs.

##### Metalycaeus suhajdai

Taxon classificationAnimaliaGastropodaCyclophoridae

Páll-Gergely nom. nov.

F90B9DF2-44C8-50F2-810C-BD059E8FC4F8

Alycaeus (Dioryx) varius Godwin-Austen, 1914: 402, pl. 157, figs 7, 7a. (non Alycaeus varius Pilsbry & Y. Hirase, 1905)
Dioryx varius – Gude 1921: 204; Ramakrishna et al. 2010: 75.

###### Type locality.

“Lhota Naga Hills”.

###### Material examined.

Lhota Naga, coll. Godwin-Austen, NHMUK 1903.7.1.2574 (1 syntype).

###### Etymology.

The specific name is dedicated to Szilárd Suhajda, the first Hungarian mountaineer to reach the peak of K2 (formerly known as Mount Godwin-Austen). Godwin-Austen was the first to fix the height and position of that mountain.

###### Remarks.

*Metalycaeus varius* (Godwin-Austen, 1914) and *Metalycaeus varius* (Pilsbry & Y. Hirase, 1905) are primary (and also secondary) homonyms. Therefore, the newer taxon needs a replacement name.

The syntype was strongly weathered, but the following observations could be made: protoconch moderately elevated, and although weathered, clearly spirally striated; R1 with strong, rather irregular ribs and weaker spiral striation; R2 very long, with elevated, sharp ribs near the tube (in other places the ribs are not visible due to corrosion).

The constriction is longer than in usual *Dioryx*, and the protoconch is spirally striated. Therefore, this species is transferred from *Dioryx* to *Metalycaeus*.

##### Metalycaeus teriaensis

Taxon classificationAnimaliaGastropodaCyclophoridae

(Godwin-Austen, 1914)

2482DCAE-DF0C-559C-B320-8D4A493C1F3D

Alycaeus teriaensis Godwin-Austen, 1914: 382, pl. 154, figs 10, 10a.
Alycaeus (Dicharax) teriaensis – Gude 1921: 272.
Chamalycaeus (Dicharax) teriaensis – Ramakrishna et al. 2010: 67.

###### Type locality.

“Teria Ghat, foot of the Khasi Hills”.

###### Material examined.

Teria Ghat, Khasi, NHMUK 1903.7.1.2750 (7 syntypes, one shell indicated with pink wool, other shells in another glass vial).

###### Remarks.

Protoconch elevated, spirally striated; R1 with irregular ribs and fine spiral striation; R2 relatively short, with widely spaced, regular, sharp ribs.

##### Metalycaeus tomotrema

Taxon classificationAnimaliaGastropodaCyclophoridae

(Möllendorff, 1887)

4493EAD7-1C5C-510B-A82D-07E9D1C108A2

Alycaeus tomotrema Möllendorff, 1887c: 292–305.
Alycaeus (Chamalycaeus) tomotrema – Kobelt 1902: 363–364.
Chamalycaeus (Chamalycaeus) tomotrema – Zilch 1957: 145, pl. 6, fig. 19.
Metalycaeus tomotrema – Páll-Gergely and Auffenberg 2019: 387, fig. 7D.

###### Type locality.

“as vicum Montalban provinciae Manila”.

###### Material examined.

Philippinen: Montalban bei Manila, leg. Möllendorff, SMF 109520 (lectotype, designated by Zilch 1957); Same data, SMF 109521 (2 paralectotypes).

###### Remarks.

Protoconch elevated, spirally striated; R1 very densely ribbed and spirally striated of approximately the same strength; R2 relatively short, with widely spaced, sharp ribs.

##### Metalycaeus yamneyensis

Taxon classificationAnimaliaGastropodaCyclophoridae

(Godwin-Austen, 1914)

9C3717D1-3C0C-5A88-892C-9FA7C507001E

Alycaeus yamnayensis (in the headings) and A. yamneyensis (in the text) Godwin-Austen, 1914: 368, pl. 156, fig. 2.
Alycaeus yamneyensis – Gude 1921: 222–223.
Alycaeus (Alycaeus) yamneyensis – Ramakrishna et al. 2010: 51; Tripathy et al. 2018: 789.

###### Type locality.

“Yamne Valley, Abor Hills”.

###### Material examined.

Yamney valley, Abor Hills, NHMUK 1903.7.1.3114 (3 shells, one of them separated with pink wool).

###### Remarks.

Protoconch elevated, spirally striated; R1 rather regularly ribbed and some spiral striation visible; R2 extremely long, with widely spaced, sharp ribs.

This species was spelled both “*yamnayensis*” and “*yamneyensis*” in the original description. The former is considered an incorrect original spelling, because the name is derived from the Yamne Valley.

##### Metalycaeus varius

Taxon classificationAnimaliaGastropodaCyclophoridae

(Pilsbry & Y. Hirase, 1905)

F7B65B3F-CB66-5D60-A3DD-D743BD598363

Alycaeus varius Pilsbry & Y. Hirase, 1905: 729.
Chamalycaeus varius – Hsieh et al. 2006: 87 + figures.

###### Type locality.

“Taihoku, Taiwan”.

###### Material examined.

Taihoku, Taiwan, ANSP 90244 (lectotype, designated by Baker 1964, photographs examined).

###### Remarks.

Protoconch elevated, spirally striated; R1 with widely spaced irregular ribs and some fine spiral striation; R2 with regular, elevated, sharp ribs.

##### Metalycaeus vinctus

Taxon classificationAnimaliaGastropodaCyclophoridae

(Pilsbry, 1902)

C617683A-F0E4-51B9-A846-70A5A60E9585

Alycaeus vinctus Pilsbry, 1902a: 53–54.
Chamalycaeus vinctus – Azuma 1982: 10, pl. 3, fig. 30; Minato 1988: 15, pl. 3, figs 5, 6.
Metalycaeus vinctus – Páll-Gergely and Asami 2017: 12–14, figs 1A; 2B, D, F; 3B, D, F; 4B, D, F; 6C, D, F; 7E, G.

###### Type locality.

“Tanegashima, Osumi”.

###### Material examined.

ANSP 83291 (lectotype, designated by Baker 1964, photographs examined); for other examined specimens see Páll-Gergely and Asami (2017).

###### Remarks.

Protoconch elevated, consists of 1.5 whorls, spirally striated except for the first 0.5 whorl which is smooth; R2 with ca. 18 ribs, which are relatively far from each other even close to the suture; the ribs are low, but all available shells were somewhat weathered.

##### Metalycaeus zayuensis

Taxon classificationAnimaliaGastropodaCyclophoridae

(Zhang, Chen & Zhou, 2008)

A08720BA-707D-5AA2-A011-28E7831C8206

Fig. 36

Chamalycaeus zayuensis Zhang, Chen & Zhou, 2008: 745, figs 1–4.
Metalycaeus (?) zayuensis – Páll-Gergely et al. 2017: 108.

###### Type locality.

“Tashan Town, Zayu County (28°04'N ,97°E), Tibet Autonomous Region, China”.

###### Material examined.

Probably lost, see remarks.

###### Remarks.

The specimens labelled as the types (Tashan Town, Zayu County, Nyingchi City, Tibet Autonomous Region, China, leg. Chen De-Niu, 1980.7.13, IZCAS TM_094538, holotype; IZCAS TM_094540, paratype) of *C. zayuensis* represent a species different from the one characterised in the original description. Namely, the real *C. zayuensis* has a smooth R2 with dense stripes, and finely ribbed R1, whereas the ones we examined have elevated, sharp, widely spaced R1 and R2 ribs. Furthermore, the ones in the Beijing Museum have sharp swelling on R3 with an incision at the middle, whereas the ones in the original description possess a relatively low, simple R3 swelling.

*Chamalycaeus zayuensis* was previously moved to the genus *Metalycaeus* based on the spiral striation (Zhouxing and Wechuan 2015; Páll-Gergely et al. 2017).

**Figure 36.**
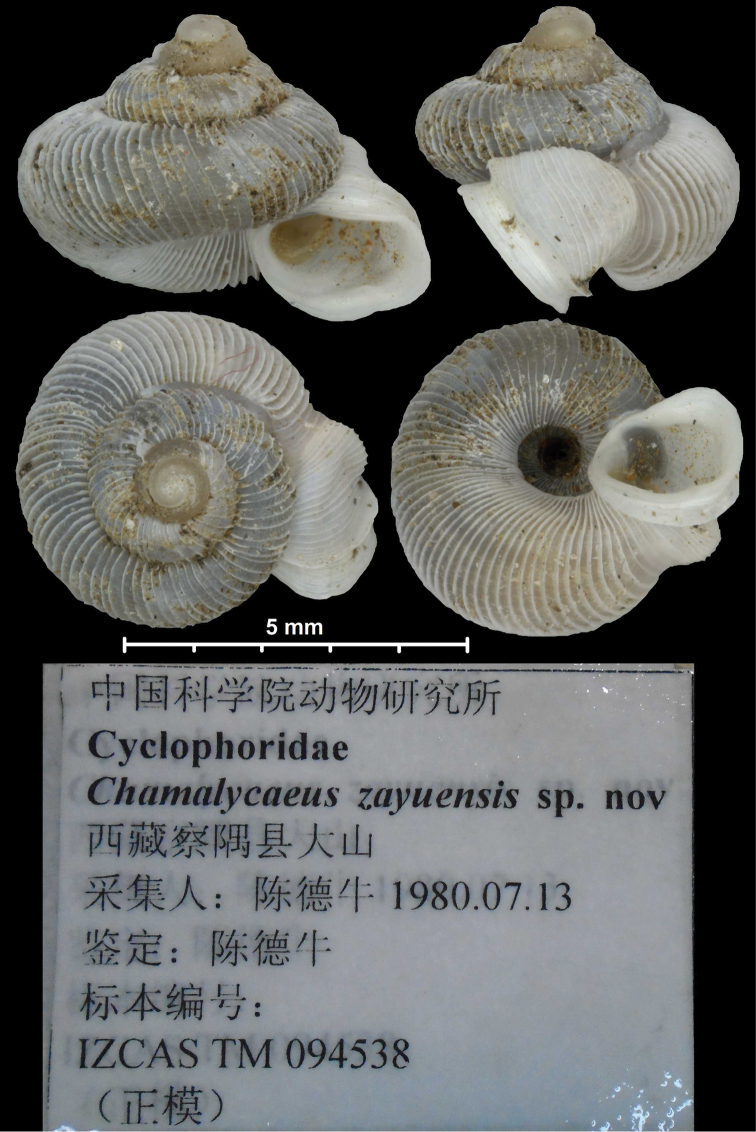
*Metalycaeus zayuensis* (Zhang, Chen & Zhou, 2008), holotype (IZCAS TM_094538). Photographs: Kaibaryer Meng.

#### Atypical (or questionable) *Metalycaeus*

##### Metalycaeus (?) aborensis

Taxon classificationAnimaliaGastropodaCyclophoridae

(Godwin-Austen, 1914)

6A7BC84A-C7FE-5080-B5F8-26FF1DA9B1C7

Alycaeus aborensis Godwin-Austen, 1914: 364, pl. 149, fig. 9.
Alycaeus (Chamalycaeus) aborensis – Gude 1921: 223.
Chamalycaeus (Chamalycaeus) aborensis – Ramakrishna et al. 2010: 52; Tripathy et al. 2018: 789.

###### Type locality.

“Bapu Peak, Abor Hills”.

###### Material examined.

Abor Hills, leg. Oakes, NHMUK 1903.7.1.3.102 (5 syntypes, one of them separated with pink wool).

###### Remarks.

Protoconch relatively low and spirally striated; R1 with rather regular, strong ribs and weaker spiral striation; R2 very long, there are no visible ribs which would elevate from the surface of the shell, alternate thicker/darker and slimmer/lighter lines, where the lighter ones represent microtunnels.

##### Metalycaeus (?) awalycaeoides

Taxon classificationAnimaliaGastropodaCyclophoridae

Páll-Gergely & Hunyadi, 2017

08F99FC3-E976-5216-B918-440EAAD25946

Metalycaeus (?) awalycaeoides Páll-Gergely & Hunyadi in Páll-Gergely et al., 2017: 74, figs 47A, 48A, B, 49A, B.

###### Type locality.

“China, Sichuan, Leshan Shi, Jinkouhe Xian, Jinhe Zhen, 610 m, 29°18.215'N, 103°6.787'E”.

###### Material examined.

Holotype (HNHM 99730).

###### Remarks.

Protoconch elevated and some remains of spiral striation visible near suture; R1 irregularly ribbed and weakly spirally striated; R2 relatively short, R2 ribs strongly developed, curved towards aperture, nearly reaching their neighbours.

##### Metalycaeus (?) dikrangensis

Taxon classificationAnimaliaGastropodaCyclophoridae

(Godwin-Austen, 1914)

3B45BD64-FBF0-583C-AD95-2106D5385B9A

Alycaeus dikrangensis Godwin-Austen, 1914: 355, pl. 148, figs 6, 6a.
Alycaeus dikrangensis – Gude 1921: 209.
Alycaeus (Alycaeus) dikrangensis – Ramakrishna et al. 2010: 48.

###### Type locality.

“Toruputu Peak, Dafla Hills”.

###### Material examined.

Toruputu Peak, Dafla Hills, leg. Godwin-Austen, NHMUK 1903.7.1.2533 (13 syntypes in 3 different vials).

###### Remarks.

Protoconch elevated, spirally striated; R1 regularly ribbed, spirally striated; R2 very long, alternate thicker/darker and slimmer/lighter stripes, the ribs are only very slightly elevated from the surface.

##### Metalycaeus (?) ibex

Taxon classificationAnimaliaGastropodaCyclophoridae

Páll-Gergely & Hunyadi, 2017

1B0D8BE8-8C20-5642-ABEB-8E98F709C0CB

Metalycaeus (?) ibex Páll-Gergely & Hunyadi in Páll-Gergely et al., 2017: 84, fig. 55E.

###### Type locality.

“Vietnam, Tonkin, Cam Duong”.

###### Material examined.

Vietnam, Tonkin, Cam Duong, leg. Messager, MNHN-IM-2012-27042 (holotype).

###### Remarks.

Sculpture of protoconch could not be examined due to strong corrosion; R1 rather regularly ribbed, with some additional, incomplete riblets between main ribs; spiral structure visible on some, not strongly weathered parts of R1; R2 very densely ribbed.

##### Metalycaeus (?) laevis

Taxon classificationAnimaliaGastropodaCyclophoridae

(Pilsbry & Y. Hirase, 1909)

56944E6D-C895-5471-A03B-9F2ED27D9F75

Alycaeus laevis Pilsbry & Y. Hirase, 1909a: 588.
Chamalycaeus laevis – Minato 1988: 15, pl. 2, figs 6–8.
Metalycaeus laevis – Páll-Gergely and Asami 2017: 4.

###### Type locality.

“Nakanoshima (Ōsumi)”.

###### Material examined.

Nakanoshima, Ōsumi, leg. Hirase, 1908, ANSP 95831 (lectotype, designated by Baker, 1964, photographs examined); Nakanoshima, Osumi (written in Japanese), coll. Hirase 268, NSMT-Mo 7579 (6 shells).

###### Remarks.

The NSMT sample we examined contained six shells with clearly visible protoconch sculpture. Four shells had spiral lines on the protoconch but in the case of two shells no spiral striae were visible. In all cases the protoconch was elevated. R2 with ca. 36 ribs, which are only just elevated from the surface of the shell. They are seemingly fully attached to their neighbours. There are no spiral lines visible on R1. The spiral striae stop at the protoconch-teleoconch border.

##### Metalycaeus (?) libonensis

Taxon classificationAnimaliaGastropodaCyclophoridae

(Chen, Li & Luo, 2003)

FF1A1C75-992C-5D3C-90AA-833E156CFD5A

Fig. 37

Chamalycaeus libonensis Chen et al. 2003: 619–620, figs 1–3.
Metalycaeus (?) libonensis – Páll-Gergely et al. 2017: 105.

###### Type locality.

“Feng Dong, Libo County (25°4'N, 107°8'E), Guizhou Province, China”.

###### Material examined.

Feng Dong, Libo County, Qian-Nan Prefecture, Guizhou Province, China, leg. Chen De-Niu, 2001.7.8, IZCAS TM 094538 (holotype).

###### Remarks.

Protoconch relatively elevated, some spiral striation is visible on the photographs; R1 with relatively widely-spaced ribs and fine spiral striation; R2 + R3 ca 90° combined R2 slightly shorter than R3; R2 ribs sharp but relatively low, similar to those on R1; R3 with a low but relatively sharp swelling.

**Figure 37.**
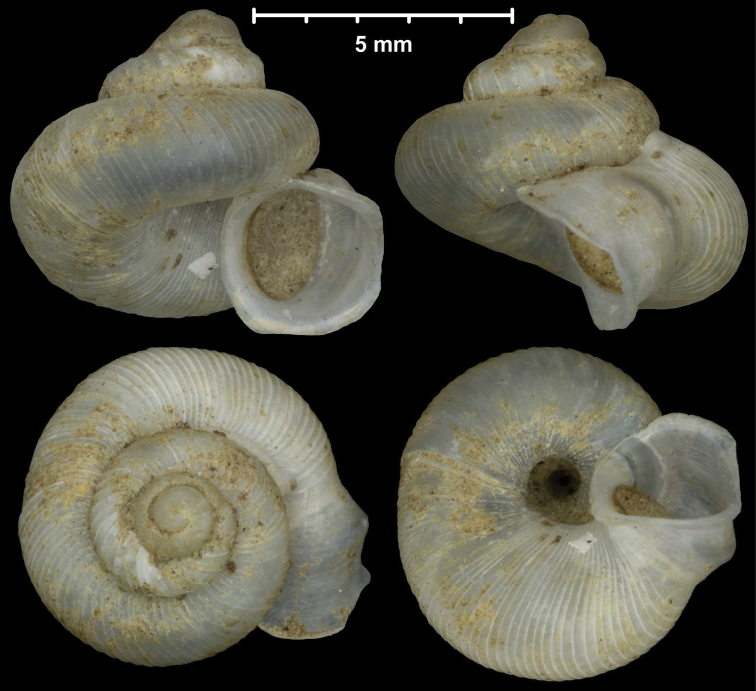
Metalycaeus (?) libonensis (Chen, Li & Luo, 2003), holotype. Photographs: Kaibaryer Meng.

##### Metalycaeus (?) magnificus

Taxon classificationAnimaliaGastropodaCyclophoridae

(Godwin-Austen, 1914)

89FBEA89-FF04-5F69-BB64-7CB3DB588C62

Fig. 31B

Alycaeus (Raptomphalus) magnificus Godwin-Austen, 1914: 366, pl. 156, figs 1, 1a, 1b.
Alycaeus (Raptomphalus) magnificus – Gude 1921: 288.
Chamalycaeus (Raptomphalus) magnificus – Ramakrishna et al. 2010: 69.

###### Type locality.

“Yamne valley, Abor Hills”.

###### Material examined.

Yamney Valley, Abor Hills, NHMUK 1903.7.1.3115 (1 syntype).

###### Remarks.

The entire shell weathered, but the following observations could be made. Protoconch rather elevated, some spiral striae are recognisable near the suture; R1 without visible spiral lines; R2 short, very densely ribbed, but the fine morphology of the ribs could not be seen.

##### Metalycaeus (?) minatoi

Taxon classificationAnimaliaGastropodaCyclophoridae

Páll-Gergely, 2017

A82DBFC0-9A27-5BF5-9B10-0C120810FF8B

Metalycaeus minatoi Páll-Gergely in Páll-Gergely & Asami, 2017: 4–12, figs 1B–D; 2A, C, E; 3A, C, E; 4A, C, E; 5A, B, E; 7A–D, F.

###### Type locality.

“20151214A (locality code), Hōman jinja (宝満神社), Kukinaga, Minamitane-chō, Kumage-gun, Tanegashima Island, Kagoshima Prefecture, Japan, 30°23.051´N, 130°56.108´E”.

###### Material examined.

Holotype (NSMT-Mo 78937) and paratypes, see Páll-Gergely and Asami (2017).

###### Remarks.

protoconch elevated, spiral lines mostly visible on last 0.25 whorl; R1 with rather irregular, widely spaced ribs and fine spiral lines; R2 short, very densely ribbed, alternating very narrower/lighter and wider/dark bands.

##### Metalycaeus (?) okuboi

Taxon classificationAnimaliaGastropodaCyclophoridae

Páll-Gergely & Hunyadi, 2017

E4B5E75A-E7D5-566D-BD39-287865FE8EE2

Metalycaeus (?) okuboi Páll-Gergely & Hunyadi in Páll-Gergely et al., 2017: 97–98, figs 53A, B, 62C, D, 64, 65C–F.

###### Type locality.

“China, Yunnan, Honghehanizuyizu Zizhizhou, Luxi Xian, Zhongshu Zhen, Alugudong”.

###### Material examined.

Holotype (HNHM 99713) and several other samples, see Páll-Gergely et al. (2017).

###### Remarks.

Protoconch moderately elevated, spiral lines visible on the last whorl of protoconch (although there was no clear distinction between protoconch and teleoconch); R1 irregularly ribbed, ribs low but relatively sharp at the end of R1; there are signs of very weak spiral lines between ribs on R1; R2 ribs not erected but situated horizontally. For more details see the original description.

##### Metalycaeus (?) panggianus

Taxon classificationAnimaliaGastropodaCyclophoridae

(Godwin-Austen, 1914)

DCF05423-28DF-58EB-81AA-C73041CB6401

Alycaeus panggiana Godwin-Austen, 1914: 367, pl. 156, figs 3, 3a.
Alycaeus panggianus – Gude 1921: 213–214.
Alycaeus (Alycaeus) panggianus – Ramakrishna et al. 2010: 50; Tripathy et al. 2018: 789.

###### Type locality.

“Sibbum, Abor Hills”.

###### Material examined.

Sibbum, Abor Hills, coll. Oakes, NHMUK 1903.7.1.3143 (holotype [single specimen mentioned in the original description]).

###### Remarks.

In the original description the subgeneric name *Raptomphalus* is not noted. However, the species is introduced after Alycaeus (Raptomphalus) magnificus, and the characteristically keeled umbilicus is similar to this species.

Protoconch elevated, spirally striated; R1 regularly ribbed, with recognisable spiral striae; R2 moderately long, with dense ribs, but their fine structure could not be examined due to the weathered condition of the holotype.

##### Metalycaeus (?) rubinus

Taxon classificationAnimaliaGastropodaCyclophoridae

(Godwin-Austen, 1893)

8B889BA8-A3DD-5C90-BF28-8AD081CF94CD

Alycaeus rubinus Godwin-Austen, 1893: 594.
Alycaeus rubinus – Godwin-Austen 1897: 3–4, pl. 63, figs 2, 2a; Godwin-Austen 1914: 412; Gude 1921: 217–218.
Alycaeus (Alycaeus) rubinus – Kobelt 1902: 350.

###### Type locality.

“Ruby Mines District, Upper Burmah”.

###### Material examined.

Ruby Mines District, Up. Burma, leg. Doherty, NHMUK 1903.7.1.2685 (2 syntypes).

###### Remarks.

Protoconch elevated, very finely spirally striated; R1 irregularly wrinkled, glossy, without spiral lines; R2 moderately long, with low, blunt, simple ribs.

##### Metalycaeus (?) sibbumensis

Taxon classificationAnimaliaGastropodaCyclophoridae

(Godwin-Austen, 1914)

F72FEB00-AFFB-5950-A5C4-D92E19595427

Alycaeus sibbumensis Godwin-Austen, 1914: 367, pl. 156, figs 4, 4a.
Alycaeus sibbumensis – Gude 1921: 219.
Alycaeus (Alycaeus) sibbumensis – Ramakrishna et al. 2010: 51; Tripathy et al. 2018: 789.

###### Type locality.

“Sibbum, Abor Hills”.

###### Material examined.

Sibbum, Abor Hills, leg. Oakes, NHMUK 1903.7.1.3142 (2 syntypes).

###### Remarks.

Protoconch elevated, spirally striated; R1 irregularly ribbed, with some signs of spiral striation; R2 very long, with alternating darker and lighter stripes forming a nearly smooth surface; the darker stripes probably represent the very low ribs.

##### Metalycaeus (?) toruputuensis

Taxon classificationAnimaliaGastropodaCyclophoridae

(Godwin-Austen, 1914)

8FCEFA02-9963-57ED-A2DC-F3FC58F5E96C

Fig. 38

Alycaeus Theobaldi – Godwin-Austen, 1876: 175–176, pl. 7, fig. 10. (operculum).
Alycaeus theobaldi – Nevill 1878: 290.
Alycaeus toruputuensis Godwin-Austen 1914: 359–360, pl. 149, figs 3, 3a, b, pl. 145, fig. 10 (operculum).
Alycaeus (Dicharax) toruputuensis – Gude 1921: 274.
Chamalycaeus (Dicharax) toruputuensis – Ramakrishna et al. 2010: 68.

###### Type locality.

“on the slopes of Torúpútú peak in the Dafla Hills” (in Godwin-Austen 1914).

###### Material examined.

Slope of Toruputu peak, Dafla Hills, Assam, coll. Godwin-Austen, NZSI M.8037 (holotype, “the specimen figured is the type shell”, “type in Indian Museum, Calcutta”: Godwin-Austen 1914: 360); Toruputu Peak, Dafla Hills, leg. Godwin-Austen, NHMUK 1903.7.1.2496 (6 paratypes in two different vials, one shell is indicated with pink wool).

###### Remarks.

Protoconch elevated, spirally striated; R1 with rather regular, strong ribs and much weaker spiral lines; R2 smooth, somewhat wavy, with thick darker and slimmer lighter stripes alternating.

**Figure 38.**
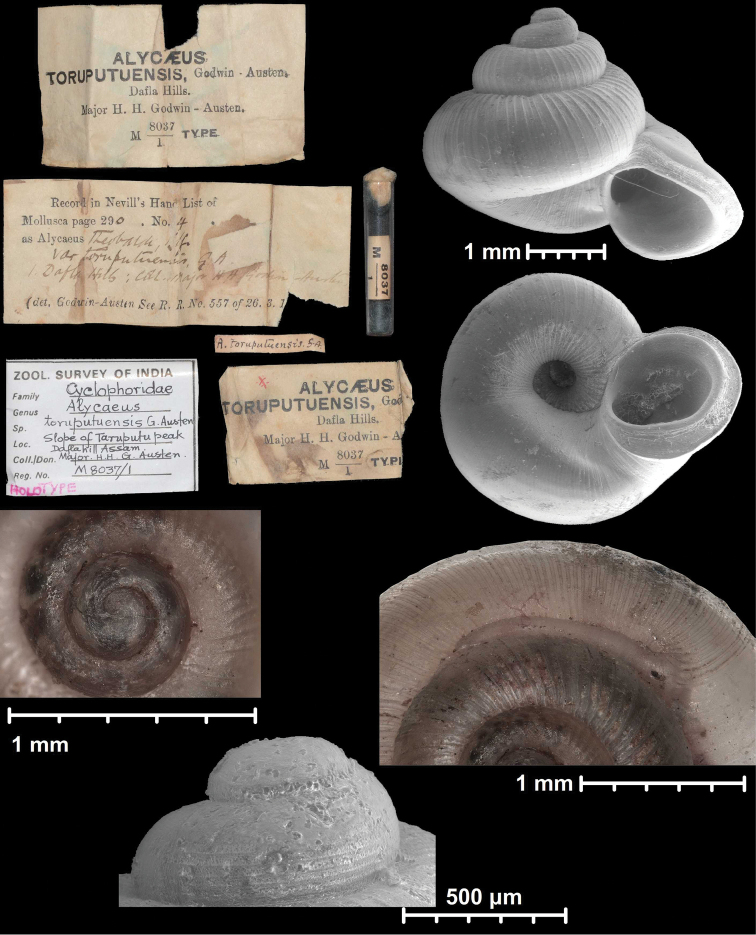
Metalycaeus (?) toruputuensis (Godwin-Austen, 1914), holotype (NZSI M.8037). All images: Sheikh Sajan.

##### Metalycaeus (?) vesica

Taxon classificationAnimaliaGastropodaCyclophoridae

(Godwin-Austen, 1914)

FBBEAF43-915D-5A06-A789-0C6BF52EE987

Alycaeus vesica Godwin-Austen 1914: 368, pl. 149, fig. 10.
Alycaeus vesica – Gude 1921: 220.

###### Type locality.

“Bapu Peak, Abor Hills”.

###### Material examined.

Abor Hills, Nr. Shimang, leg. Oakes, NHMUK 1903.7.1.3101 (2 syntypes in different vials).

###### Remarks.

Protoconch elevated, spirally striated; R1 irregularly ribbed and finely spirally striated; R2 very long, with widely spaced, but relatively low, slightly waved ribs.

#### Genus Pincerna

Taxon classificationAnimaliaGastropodaCyclophoridae

Preston, 1907

F4D5B910-3B74-5A36-A6B9-77F07BCD341B

Alycaeus (Pincerna) Preston, 1907: 206.
Alycaeus (Cycloryx) Godwin-Austen 1914: 334.
Chamalycaeus (Cycloryx) – Thiele 1929: 108; Wenz 1938: 478.
Alycaeus (Pincerna) – Thiele 1929: 108; Wenz 1938: 479; Egorov 2013: 33–34.
Chamalycaeus (Cycloryx) – Egorov 2013: 36.
Pincerna – Páll-Gergely 2017: 214.

##### Type species.

**Figure 39.**
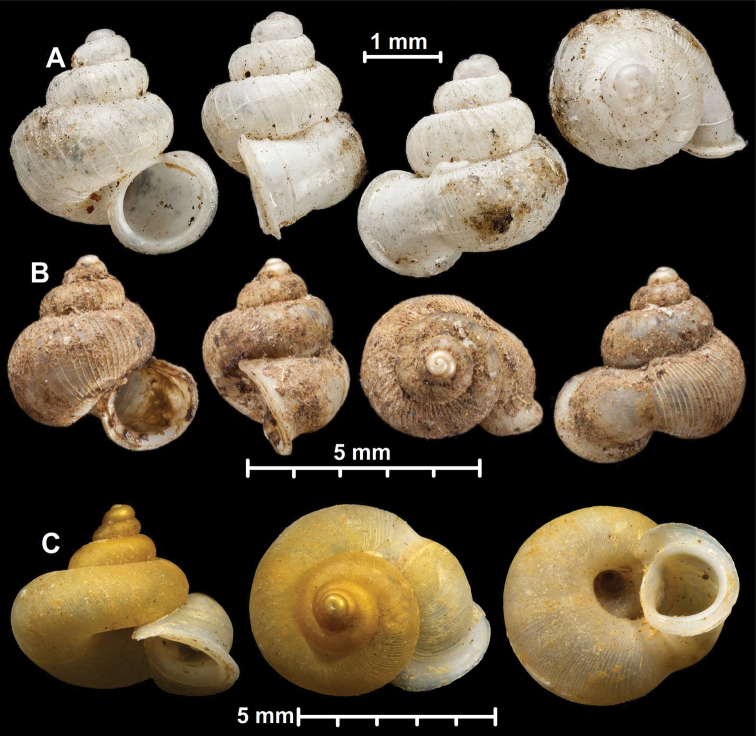
Type species of alycaeid genus-group taxa **A***Pincerna constricta* (Benson, 1851), syntype (NHMUK 1906.4.4.41; type species of *Cycloryx*) **B***Pincerna liratula* (Preston, 1907), syntype (NHMUK 1907.5.20.191; type species of *Pincerna*) **C***Stomacosmethis sarasinorum* (Bollinger, 1918), lectotype (NHMB 2411.a; type species of *Stomacosmethis*). Photographs: Harold Taylor (**A, B**); Eduard Stoeckli (**C**).

*Pincerna liratula* Preston, 1907 (Fig. 39B) (introduced as a subgenus of *Alycaeus*, but apparently used on genus level), by monotypy.

##### Diagnosis.

Shell very small to medium sized (D: 2.5–6 mm), globose triangular, usually higher than wide; protoconch smooth, without spiral striation; R1 usually with strong, widely-spaced ribs and weak spiral striation; R2 smooth to prominently ribbed (in those cases not different from R1), very short, pyriform (in typical *Cycloryx* species) or somewhat longer in *Pincerna*-like shells; umbilicus often closed by the reflected columellar extension in many species. Operculum thin, in some species with a low circular structure, or calcareous ridges radiating outwards of the nucleus on the outer surface. Radula of a single species is known, see Table 1 (central tooth with 5 cusps, broad, central cusp pointed).

##### Differential diagnosis.

See Remarks.

##### Distribution.

**Figure 40.**
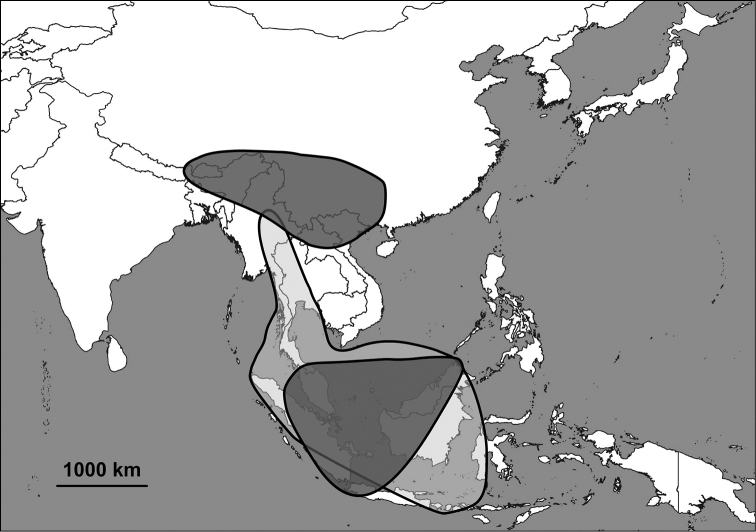
Distribution of *Pincerna* Preston, 1907 (dark shaded area) and *Stomacosmethis* Bollinger, 1918 (light shaded area).

The distribution of *Pincerna* seemingly consists of two disjunct geographic areas, namely the southeastern Himalaya to northern Vietnam and northern Laos, and Sumatra, the southern part of the Malay Peninsula, and northern Borneo (Fig. 40).

##### Remarks.

The relationship between *Alycaeus*, *Pincerna*, and *Cycloryx* is the most problematical point of the current revision. The genus *Cycloryx* Godwin-Austen, 1914 (type species: *Cyclostoma constrictum* Benson, 1851, OD) was erected as a subgenus of *Alycaeus* Gray, 1850, and was described on the basis of the ovately conoid shell shape, the regular ribbing on the upper whorls, and the extremely short, often clubbed or pear-shaped sutural tube (Godwin-Austen 1914). Godwin-Austen (1914) only included species from northeastern India and Burma (Rakhin = Arakan, and the Shan States) in this genus. However, the diagnosis of *Cycloryx* matches several species extralimital to the distributional range as defined by Godwin-Austen, namely those from northern Vietnam, Borneo, China’s Guizhou Province, the Malay Peninsula, and Sumatra. The most striking example is the Sumatran *Pincerna yanseni* and the northern Vietnamese *Alycaeus costulosus*, which both look so similar to Indian and Burmese species that it was challenging to find any differences between those two and the Indian and Burmese taxa. One of those “extralimital *Cycloryx*” is *Alycaeus liratulus* Preston, 1907, known from the Malay Peninsula and Sumatra, which has been placed in its own subgenus, *Pincerna* Preston, 1907. Originally, *Pincerna* was diagnosed on the basis of a “circular cup” on the outer surface of the operculum. The outer surface of operculum, however, has limited taxonomic value at the generic level in the Alycaeidae, especially since the outer rings have evolved in multiple alycaeid genera (*Dicharax*, *Metalycaeus*, *Stomacosmethis*; see this study and Páll-Gergely et al. 2017). Moreover, the most similar species to *P. liratula*, *P. thieroti*, lacks the circular cup on the outer surface of the operculum. Consequently, no important conchological characters distinguish *Cycloryx* and *Pincerna*, and thus, they have been synonymised (Páll-Gergely 2017). See also under Genus-level diversity (p. 13).

*Pincerna* is globular and sparsely ribbed, *Stomacosmethis* is triangular and densely ribbed. *Stomacosmethis balingensis* is globular, but densely ribbed, forming a connection between the two genera, but the radula morphology unambiguously indicates its position within *Stomacosmethis*. We retain the two genera separate due to the unique radular morphology of all known *Stomacosmethis* species. Furthermore, there are many species with typical features of both respective genera. See also under *Alycaeus* (p. 17).

#### Pincerna (?) anceyi

Taxon classificationAnimaliaGastropodaCyclophoridae

(Mabille, 1887)

F5B64AEC-C919-5CE7-B373-1AB238121753

Alycaeus anceyi Mabille 1887: 151–152, pl. 3, figs 14, 15.
Alycaeus (Alycaeus) anceyi – Kobelt 1902: 341.

##### Type locality.

“Tonkin” (from the title).

##### Material examined.

Tonkin, coll. Mabille, MNHN-IM-2000-31797 (3 syntypes).

##### Remarks.

Protoconch very finely granulated, matte; R1 nearly smooth, with irregular, very fine growth lines, and some fine ribbing near suture, spiral striation not visible; R2 short, darker thicker and somewhat narrower light stripes, but overall surface smooth.

The shell shape is similar to species from the genus *Pincerna*, but there are no strong ribs in the upper whorls of the shell, the tube is elongated, and not extremely short and piriform. The tube is shorter than usual for the genus *Alycaeus*, but the shell shape agrees with the other species classified within the genus.

#### Pincerna bembex

Taxon classificationAnimaliaGastropodaCyclophoridae

(Benson, 1859)

ECC0F7F6-6E2C-583C-AA49-C9227C8AE12F

Alycaeus bembex Benson, 1859: 178–179.
Alycaeus bembex – Reeve 1878: pl. 5, species 42; Godwin-Austen 1884: pl. 51, fig. 5.
Alycaeus (Alycaeus) bembex – Kobelt 1902: 342.
Alycaeus (Cycloryx) bembex – Godwin-Austen 1914: 346–347, pl. 147, figs 1, 1a; Gude 1921: 275–276.
Cycloryx bembex – Ramakrishna et al. 2010: 70.

##### Type locality.

“in valle Rungun”.

##### Material examined.

Darjiling, NHMUK 1906.4.4.44 (6 possible syntypes, one of them separated in a different vial and marked with a “T”).

##### Remarks.

Protoconch rather matte without any notable sculpture; R1 with widely spaced ribs and several, much finer radial lines between the larger ribs; R2 extremely short, with ca. 12 very narrow light stripes, and much thicker darker stripes.

#### Pincerna burrailensis

Taxon classificationAnimaliaGastropodaCyclophoridae

(Godwin-Austen, 1914)

8DE34F79-AD56-5A9A-8AEB-B5E7D09C97DE

Alycaeus (Cycloryx) burrailensis Godwin-Austen, 1914: 403, pl. 147, figs 6, 6a.
Alycaeus (Cycloryx) burrailensis – Gude 1921: 276.
Cycloryx burrailensis – Ramakrishna et al. 2010: 70.

##### Type locality.

“Japvo Peak, Naga Hills, 9890 ft”.

##### Material examined.

Japvo Peak, Naga Hills, 9890 f, leg. Godwin-Austen, Acc. no. 1830, NHMUK 1903.7.1.2591 (6 syntypes).

##### Remarks.

Protoconch matte, without spiral striation; R1 with regular, rather dense, sharp, elevated ribs and occasionally extremely fine spiral striation; R2 extremely short, with four or five ribs only, sculpture of R2 does not differ from that of R1.

#### Pincerna constricta

Taxon classificationAnimaliaGastropodaCyclophoridae

(Benson, 1851)

5F1BBAE8-5014-516E-B964-CE64BC3E39B3

Fig. 39A

Cyclostoma constrictum Benson, 1851: 184–195.
Alycaeus constrictus var. minor Benson, 1859: 181.
Alycaeus constrictus – Reeve 1878: pl. 5, species 41.
Alycaeus (Alycaeus) constrictus – Kobelt 1902: 343.
Alycaeus (Cycloryx) constrictus – Godwin-Austen 1914: 347–348, pl. 147, figs 4, 4a; Godwin-Austen 1914: 348–349, pl. 154, figs 1, 1a; Gude 1921: 277–278.
Alycaeus (Cycloryx) constrictus , var. minor – Godwin-Austen 1914: 348.
Cycloryx constrictus – Ramakrishna et al. 2010: 70.
Pincerna constricta – Páll-Gergely 2017: 218, fig. 1A.

##### Type locality.

“ad Darjiling Himalayæ Sikkimensis”.

##### Material examined.

UMZC I.103745 (1 syntype); Rungun Valley, Darjiling, NHMUK 1906.4.4.41 (5 shells).

##### Remarks.

Protoconch matte, without spiral lines; R1 with very sharp, widely spaced ribs, and extremely fine spiral lines; R2 very short, with alternating darker/wider and much narrower/lighter stripes (altogether ca. 12), overall surface smooth.

#### Pincerna costata

Taxon classificationAnimaliaGastropodaCyclophoridae

(Godwin-Austen, 1914)

3C924D68-866E-5301-8E47-594B59EA0F30

Alycaeus (Cycloryx) costatus Godwin-Austen, 1914: 360–361, pl. 154, figs 2, 2a.
Alycaeus (Cycloryx) costatus – Gude 1921: 278.
Cycloryx costatus – Ramakrishna et al. 2010: 71.
Pincerna costata – Páll-Gergely 2017: 214.

##### Type locality.

“Dikrang Valley, Dafla Hills”.

##### Material examined.

Toruputu Peak, Dafla Hills, Dikrang valley, Dafla, leg. Godwin-Austen, NHMUK 1903.7.1.2596 (18 syntypes).

##### Remarks.

Protoconch matte, no spiral lines visible; R1 with regular, widely spaced, sharp ribs and extremely fine spiral striation; R2 very short, with ca. five ribs which are similar to those of in R2 but denser.

#### Pincerna costulosa

Taxon classificationAnimaliaGastropodaCyclophoridae

(Bavay & Dautzenberg, 1912)

7C36A7C8-790D-57B8-B698-1E693A7457CA

Alycaeus costulosus Bavay & Dautzenberg, 1912: 49–50, pl. 4, figs 1–4.
Pincerna costulosa – Z.-Y. Chen & M. Wu 2020: 45, figs 1, 4C, D

##### Type locality.

“Phong-Tho, Tonkin”.

##### Material examined.

Tonkin, Phong Tho, leg. Messager, MNHN-IM-2000-31786 (1 syntype); Tonkin, Phong Tho, probably leg. Messager, coll. Staadt, 1969, MNHN-2012-27043 (4 shells, 2 of them typical *costulosus*, 2 other shells small *vanbuensis*).

##### Remarks.

Protoconch matte, no spiral lines visible; R1 strongly, regularly ribbed with hardly visible, extremely fine spiral striation; R2 extremely short, with ca. seven narrow, light stripes, the overall surface is smooth.

Some Indian species originally included in *Cycloryx* (e.g., P. graphica var. dihingensis, *P. thompsoni*, *P. burrailensis*) are so similar to *P. costulosa* that it was challenging to find any notable differences. *Pincerna costulosa* must evidently be classified in *Pincerna* in the face of the large geographic gap between it and the Indian/Burmese species.

#### Pincerna (?) crenilabris

Taxon classificationAnimaliaGastropodaCyclophoridae

(Möllendorff, 1897)

F3E4FEA4-4C34-58D1-813D-18A7B45EC596

Alycaeus (Orthalycaeus) crenilabris Möllendorff, 1897b: 93.
Alycaeus (Alycaeus) crenilabris – Kobelt 1902: 343; Zilch 1957: 146–147, pl. 6, fig. 25.
Alycaeus crenilabris – van Benthem Jutting 1948: 572–573, fig. 28.

##### Type locality.

“Java” (from the title).

##### Material examined.

W-Java, Djampang, 2000’, leg. H. Fruhstorfer, 1893, coll. O. Boettger, SMF 57197 (syntype, labelled as holotype [number of available shells was not mentioned in the original description]); Java, Djampong (?), Res.: Preauger-Regentschappen, leg. ??? bauer (label not readable), coll. Oberwimmer, NHMW 111538 (3 shells).

##### Remarks.

The spire is high, but the protoconch is relatively low, it is rather matte, without spiral lines; R1 with some spiral lines, especially at the end of the region; There are widely spaced, strong ribs on R1 which are bunt at the beginning, but sharp at the end of the region; R2 short, with elevated, sharp, widely spaced ribs with some weak spiral striation between the ribs.

This species fits in the genus *Chamalycaeus* as well as into *Pincerna*. It is tentatively placed here due to the elevated spire. Further studies should focus on its systematic relationships.

#### Pincerna (?) crenilabris korintjiensis

Taxon classificationAnimaliaGastropodaCyclophoridae

Páll-Gergely nom. nov.

46DFCC6E-6583-55A2-8556-B9E92FCCFC13

Alycaeus crenilabris latecostatus van Benthem Jutting, 1959: 80. (non Alycaeus latecostatus Möllendorff, 1882)

##### Type locality.

“Kajo Aro Estate, Mt. Korintji, among fallen leaves, 1450 m alt.”.

##### Etymology.

The replacement name *korintjiensis* refers to the type locality (Mt. Korintji).

##### Remarks.

The holotype and the paratype were not examined by us, but according to the original description the differences between this and the nominotypical subspecies are only minor (more distantly ribbed than the nominotypical subspecies).

*Alycaeus crenilabris latecostatus* van Benthem Jutting, 1959 is a junior primary homonym of *Alycaeus latecostatus* Möllendorff, 1882 (treated as *Metalycaeus* here). Thus, a replacement name (*korintjiensis*) is proposed to replace the junior homonym.

#### Pincerna (?) crenilabris juttingae

Taxon classificationAnimaliaGastropodaCyclophoridae

Páll-Gergely nom. nov.

71975146-4CB3-5B1C-9FBE-B427B6C976A3

Alycaeus crenilabris laevis van Benthem Jutting, 1959: 79–80. (non Alycaeus laevis Pilsbry & Y. Hirase, 1909)

##### Type locality.

“Brastagi, 1750 m alt., among dead leaves”.

##### Etymology.

This subspecies is dedicated to and named after Woutera Sophie Suzanna van Benthem Jutting (1899–1991), who described this taxon under the name *Alycaeus crenilabris laevis*.

##### Remarks.

The holotype and the three paratypes were not examined by us, but according to the original description, this and the nominotypical subspecies differ only in the sculpture of R3 and the formation of the peristome.

*Alycaeus crenilabris laevis* van Benthem Jutting, 1959 is a primary homonym of *Alycaeus laevis* Pilsbry & Y. Hirase, 1909, therefore a replacement name (*juttingae*) is proposed here.

#### Pincerna difficilis

Taxon classificationAnimaliaGastropodaCyclophoridae

(Godwin-Austen, 1914)

6262B508-ACA5-5F19-8BDC-1CF77E602B2E

Alycaeus (Cycloryx) difficilis Godwin-Austen, 1914: 415–416, pl. 155, figs 2, 2a.
Alycaeus (Cycloryx) difficilis – Gude 1921: 278–279.

##### Type locality.

“Shan Hills”.

##### Material examined.

Shan States, leg. Fedden, NHMUK 1906.5.5.22 (2 syntypes).

##### Remarks.

Protoconch matte, no spiral lines visible; R1 with widely spaced, strong, sharp, regular ribs and much weaker riblets between the main ribs; R2 extremely short, the few (ca. five) ribs are somewhat elevated from the surface.

#### Pincerna elegans

Taxon classificationAnimaliaGastropodaCyclophoridae

(Godwin-Austen, 1914)

079F6611-2FAB-5BA8-AD1E-2A8ACDFEA6F9

Alycaeus (Cycloryx) elegans Godwin-Austen, 1914: 361, pl. 147, fig. 9.
Alycaeus (Cycloryx) elegans – Gude 1921: 279.
Cycloryx elegans – Ramakrishna et al. 2010: 71.
Pincerna elegans – Páll-Gergely 2017: 214.

##### Type locality.

“Shengorh Peak, Dafla Hills”.

##### Material examined.

Shengorh Pk, Dafla Hills, coll. Godwin-Austen, NHMUK 1903.7.1.2594 (1 syntype).

##### Remarks.

Protoconch matte, without spiral striation; R1 with relatively dense, regular, sharp ribs and extremely fine, pitted spiral striation between the ribs; R2 with ca. eight ribs, which are similar to those of R1.

#### Pincerna globosa

Taxon classificationAnimaliaGastropodaCyclophoridae

(H. Adams, 1871)

9D1E07B3-918A-54E8-9763-3FA38974C42A

Alycaeus globosus H. Adams, 1871: 794.
Alycaeus globosus – Godwin-Austen 1889: 346–347, pl. 37, figs 3, 3a; E. A. Smith 1895: 116.
Alycaeus (Alycaeus) globosus – Kobelt 1902: 345.

##### Type locality.

“Busan, near Sarawak, Borneo”.

##### Material examined.

Busan Hills, Borneo, “typical” NHMUK 1889.12.7.30–31 (4 shells, possible syntypes); Borneo, ex coll. Fulton, London, NHMW 21003 (2 shells).

##### Remarks.

Protoconch relatively low in face of the elevated spire, glossy, R1 regularly ribbed with widely spaced, sharp ribs and extremely fine spiral striation; R2 extremely short, with alternating light/very narrow and darker/wider stripes; there are five or six light stripes, and they are very slightly elevated from the surface.

#### Pincerna globosa kinabaluana

Taxon classificationAnimaliaGastropodaCyclophoridae

(E. A. Smith, 1895)

844DC18F-FAFB-58A7-A8C2-8E13994B68E7

Alycaeus globosus var. kina-baluana E. A. Smith, 1895: 116.

##### Type locality.

“Kina-Balu, N. Borneo”.

##### Material examined.

Kina Balu, NHMUK 1893.6.7.104–107 (4 shells, possible syntypes).

##### Remarks.

Similar to the nominotypical form but has a wider peristome and the end of R1 is almost smooth.

#### Pincerna globosa muluana

Taxon classificationAnimaliaGastropodaCyclophoridae

(E. A. Smith, 1895)

3944BE93-2A95-5733-903E-ABDCDE4C7C9A

Alycaeus globosus var. muluana E. A. Smith, 1895: 116.

##### Type locality.

“Mulu Mountain”.

##### Material examined.

Mulu Mt., N. Borneo, leg. A. Everett, NHMUK 1892.2.5.11–14 (4 syntypes).

##### Remarks.

As in the nominotypical form in the mentioned traits.

#### Pincerna globosa pygmaea

Taxon classificationAnimaliaGastropodaCyclophoridae

(E. A. Smith, 1895)

6C2A4F01-A8B7-502B-AF66-9789CB4632A6

Alycaeus globosus var. pygmaea E. A. Smith, 1895: 116.

##### Type locality.

“Mulu Mountain”.

##### Material examined.

Mulu Mt., N. Borneo, NHMUK 1892.7.20.33 and 1893.6.8.10 (2 syntypes).

##### Remarks.

Protoconch matte, R1 with strong, regular, widely spaced, sharp ribs without spiral striations; R2 short with ribs that are similar to the ones on R1 in morphology and density.

Smith (1895) advised considering all forms of *Alycaeus globosus* under one species because there is “such a general resemblance throughout the series”. Although this is true, the size difference between *pygmaea* and *muluana* are extreme. If they were truly collected from the same site, then they should be treated as distinct species. However, the accuracy of the localities is unknown.

#### Pincerna globosa rabongensis

Taxon classificationAnimaliaGastropodaCyclophoridae

(E. A. Smith, 1895)

431353A1-8CF7-5DDF-8637-2CE194BEAFAC

Alycaeus globosus var. rabongensis E. A. Smith, 1895: 116.

##### Type locality.

“Mount Rabong, Sarawak”.

##### Material examined.

Mt. Rabong, NHMUK 1893.6.7.89–92 (4 syntypes); Borneo, Mt. Rabong, NHMW 40997 (2 shells).

##### Remarks.

Protoconch without spiral striae; R1 with regular, elevated, sharp ribs and weak spiral striae; R2 very short, with 6–8 darker/thicker stripes and lighter/narrower ones which are relatively well elevated from the surface.

#### Pincerna granum

Taxon classificationAnimaliaGastropodaCyclophoridae

(Godwin-Austen, 1893)

274C7F99-DB25-5B72-928B-108D76385581

Alycaeus (Dioryx) granum Godwin-Austen, 1893: 593–594.
Alycaeus (Dioryx) granum – Godwin-Austen 1897: 4–5, pl. 63, fig. 6.
Alycaeus (Alycaeus) granum – Kobelt 1902: 345.
Alycaeus (Cycloryx) granum – Godwin-Austen 1914: 364; Gude 1921: 279–280.
Cycloryx granum – Ramakrishna et al. 2010: 71.

##### Type locality.

“Margarita, foot of Eastern Naga Hills”.

##### Material examined.

Margarita, near Sadia, Assam, coll. Godwin-Austen, NHMUK 1903.7.1.2511 (1 syntype).

##### Remarks.

Protoconch matte, R1 with widely spaced, regular, sharp ribs and extremely fine spiral striation; R2 very short, with ca. 3 ribs, which are denser than those on R1.

#### Pincerna graphiaria

Taxon classificationAnimaliaGastropodaCyclophoridae

(Godwin-Austen, 1914)

964CACF8-10E8-5B9B-AC97-919F82DC24AF

Alycaeus (Cycloryx) graphiarius Godwin-Austen, 1914: 416, pl. 146, figs 7, 7a.
Alycaeus (Cycloryx) graphiarius – Gude 1921: 280.

##### Type locality.

“Burma and Shan States (the Irravady and Salween Valleys)” (from the chapter title on page 405).

##### Material examined.

Shan States, leg. Fedden, coll. Blanford, NHMUK 1906.4.4.21 (1 syntype).

##### Remarks.

Protoconch matte, no spiral lines visible; R1 with extremely widely spaced, sharp ribs and extremely fine spiral striation; R2 very short, with few ribs similar to those on R1 in morphology, but denser.

#### Pincerna graphica

Taxon classificationAnimaliaGastropodaCyclophoridae

(W. T. Blanford, 1862)

2309DE69-13B4-5BAC-886D-89D120A96B77

Alycaeus graphicus W. T. Blanford, 1862: 137–138.
Alycaeus graphicus – Reeve 1878: pl. 4, species 34.
Alycaeus graphicus var. – Theobald 1870: 398, pl. 18, fig. 3.
Alycaeus (Dioryx) graphicus var. minor Godwin-Austen, 1874: 149, pl. 3, fig. 4.
Alycaeus (Alycaeus) graphicus – Kobelt 1902: 345.
Alycaeus (Cycloryx) graphicus – Godwin-Austen 1914: 361, 419–420, pl. 146, figs 1, 1a, 1b; Gude 1921: 280–281.
Alycaeus (Cycloryx) graphicus var. minor – Godwin-Austen 1914: 403, pl. 144, figs 9, 9a.
Cycloryx graphicus – Ramakrishna et al. 2010: 72.

##### Type locality.

“in montibus Arakanensibus provinciam Burmanam Pegu ad Arakan secernentibus”.

##### Material examined.

Moditoung, Tongoop Pass, Arakan, NHMUK 1906.4.4.42 (4 syntypes).

##### Remarks.

Protoconch glossy, no spiral lines visible; R2 with widely spaced, regular, sharp ribs and without spiral striation; R2 extremely short, consisting of six or seven ribs that are similar to the ones on R1, but are denser.

#### Pincerna graphica dihingensis

Taxon classificationAnimaliaGastropodaCyclophoridae

(Godwin-Austen, 1914)

DD45C973-EAED-5B12-A875-0FD757FA006E

Alycaeus (Cycloryx) graphicus var. dihingensis Godwin-Austen, 1914: 363–364, 404, pl. 146, figs 6, 6a.
Alycaeus (Cycloryx) graphicus Var. dihingensis – Gude 1921: 281–282.

##### Type locality.

“Sadia, Assam, 350 ft”.

##### Material examined.

Sadia, Assam, NHMUK 1903.7.1.2516 (2 syntypes).

##### Remarks.

Protoconch matte, no spiral lines visible; R1 with regular, widely spaced, sharp ribs and extremely fine spiral striation; R2 extremely short, with ca. five narrow, light stripes, which are somewhat elevated from the surface.

#### Pincerna graphica variabilis

Taxon classificationAnimaliaGastropodaCyclophoridae

(Godwin-Austen, 1914)

FB30FECA-AC42-589F-8563-2FF9E06569D9

Alycaeus (Cycloryx) graphicus var. variabilis Godwin-Austen, 1914: 403–404, pl. 146, fig. 4.

##### Type locality.

“Lhota Naga”.

##### Material examined.

Lhota Naga, NHMUK 1903.7.1.2607 (17 syntypes).

##### Remarks.

Protoconch matte, without spiral striation; R1 with rather low, regular, widely spaced ribs without spiral striation; R2 very short, with ca. six ribs which are similar to those on R1 in morphology but are more densely situated.

#### Pincerna khunhoensis

Taxon classificationAnimaliaGastropodaCyclophoridae

(Godwin-Austen, 1914)

B6266C31-A46D-550C-AE7A-41A9466E2ABD

Alycaeus (Cycloryx) khunhoensis Godwin-Austen, 1914: 403, pl. 147, fig. 8.
Alycaeus (Cycloryx) khunhoensis – Gude 1921: 282.
Cycloryx khunhoensis – Ramakrishna et al. 2010: 72.
Pincerna khunhoensis – Páll-Gergely 2017: 214.

##### Type locality.

“Khunho Peak, Naga Hills”.

##### Material examined.

Khunho Peak, Naga Hills, coll. Godwin-Austen, NHMUK 1903.7.1.2520 (1 syntype).

##### Remarks.

Protoconch matte, without spiral striation; R1 with widely spaced, sharp, regular ribs that have extremely fine spiral striation; R2 very short, with ca. five ribs similar to those of R1 in density and height.

#### Pincerna liratula

Taxon classificationAnimaliaGastropodaCyclophoridae

(Preston, 1907)

0133F64F-F424-5C3B-B9F9-7E39AEB0AFEB

Fig. 39B

Alycaeus (Pincerna) liratula Preston, 1907: 206.
Alycaeus (Alycaeus) liratulus – Laidlaw 1928: 6, 35.
Alycaeus liratulus – van Benthem Jutting 1959: 77; Foon and Liew 2017: 54–56, figs 7C, 23, 31B.
Pincerna liratula – Egorov 2013: 34, figs 59a, b; Páll-Gergely 2017: 214, fig. 1C; Páll-Gergely et al. 2017: 10, fig. 3D.

##### Type locality.

“Ke-lan-tan”.

##### Material examined.

Ke-lan-tan, Malay Penins., ex coll. Preston, 1909, ANSP 99391 (1 syntype, photographs examined); Ke-lan-tan, Purchased of Mr. H.B. Preston, NHMUK 1907.5.20.191–192 (2 syntypes).

##### Remarks.

Protoconch relatively low, no spiral lines visible; R1 with widely spaced, relatively low ribs, with finer spiral striation; R2 moderately short, with strong, irregular ribs and weak spiral striation; outer surface of the operculum rather glossy, multispiral, with an elevated ring on the edge of the nucleus.

#### Pincerna major

Taxon classificationAnimaliaGastropodaCyclophoridae

(Godwin-Austen, 1893)

91451A61-73A6-5AE3-83EF-15FB34AF3715

Alycaeus otiphorus var. – Godwin-Austen 1871: 93, pl. 5, fig. 6.
Alycaeus (Dioryx) granum var. major Godwin-Austen, 1893: 594.
Alycaeus (Cycloryx) mangutensis Godwin-Austen, 1914: 377–378, pl. 146, figs 5, 5a.
Alycaeus (Cycloryx) mangutensis – Gude 1921: 282.
Cycloryx mangutensis – Ramakrishna et al. 2010: 72.

##### Type locality.

“Mangut Valley, Jaintia Hills”.

##### Material examined.

Mangat valley, Jiantia Hills, leg. Godwin-Austen, NHMUK 1903.7.1.2518 (8 syntypes).

##### Remarks.

Protoconch matte, R1 with regular, widely spaced, rather low ribs; no spiral striation observed; R2 very short, with ca. four ribs which are similar to the ones on R1 in terms of density and morphology.

This species was described as *A. mangutensis* by Godwin-Austen in 1914, and that name was later used as a valid taxon name (Gude 1921; Ramakrishna et al. 2010). However, the name Alycaeus (Dioryx) granum var. major Godwin-Austen, 1893 for the same taxon was validly introduced, and is available. Thus, the species must be called *Pincerna major* (Godwin-Austen, 1893), and the name *Alycaeus mangutensis* Godwin-Austen, 1914 is a junior objective synonym.

#### Pincerna (?) maolanensis

Taxon classificationAnimaliaGastropodaCyclophoridae

(Luo, Zhang & Zhuo, 2009)

12DE86DF-E72F-5CB0-99FE-C4B10CA50829

Fig. 41

Dioryx maolanensis Luo, Zhang & Zhuo, 2009: 862–864, figs 1–6.
Pincerna maolanensis – Páll-Gergely et al. 2017: 10.

##### Type locality.

“Maolan Town, Libo County (25°03'N, 108°00'E), Guizhou Province, China”.

##### Material examined.

Mao-Lan Town, Libo County, Qian-Nan Prefecture, Guizhou Province, China, leg. Luo Tai-Chang, 2001.7.9., IZCAS TM 047081 (holotype).

##### Remarks.

Protoconch without spiral striae; R1 with strong, widely-spaced ribs and very fine spiral striation; R2 + R3 nearly 0.5 whorl together, approximately the same length; R2 long, without elevated ribs.

**Figure 41.**
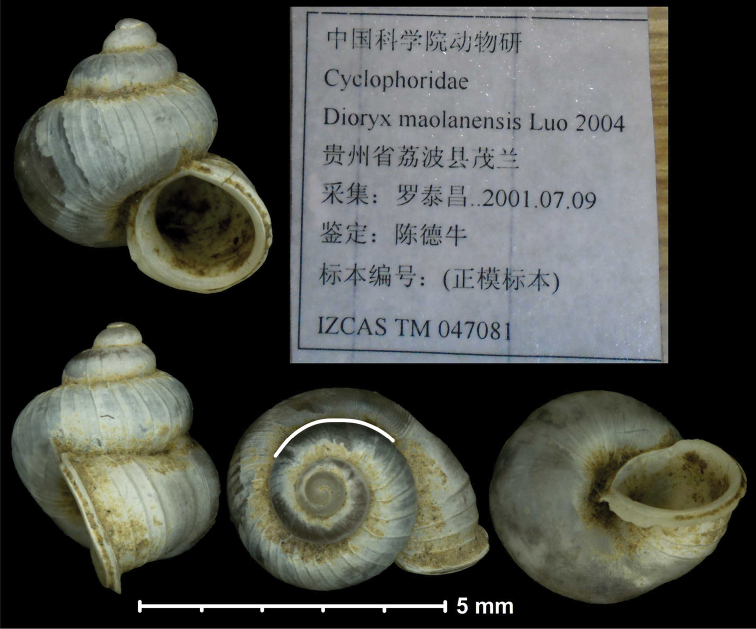
Pincerna (?) maolanensis (Luo, Zhang & Zhuo, 2009), holotype (IZCAS TM 047081). White line indicates the length of the sutural tube. Photographs: Kaibaryer Meng.

#### Pincerna margarita

Taxon classificationAnimaliaGastropodaCyclophoridae

(Theobald, 1874)

DDD27FF0-497F-555F-9E8A-46E388CC0551

Alycaeus margarita Theobald in Hanley & Theobald, 1874: 39, pl. 95, fig. 10.
Alycaeus margaritus – Reeve 1878: pl. 1, species 5.
Alycaeus (Alycaeus) margarita – Kobelt 1902: 347.
Alycaeus (Cycloryx) margarita – Godwin-Austen 1914: 416.
Alycaeus margarita – Gude 1921: 211–212.

##### Type locality.

“Shan Provinces”.

##### Material examined.

Upper Salwin, NHMUK 1888.12.4.892–893 (2 syntypes).

##### Remarks.

Protoconch matte, without spiral striation; R1 with strongly elevated and sharp, regular, widely spaced ribs; R2 extremely short, with ca. five ribs which are denser than those on R1, but similar in terms of morphology.

#### Pincerna mouhoti

Taxon classificationAnimaliaGastropodaCyclophoridae

(L. Pfeiffer, 1862)

3287A303-236A-5033-8A02-81A3F9A9BDA2

Alycaeus mouhoti L. Pfeiffer, 1862: 275, pl. 36, figs 1, 2.
Alycaeus mouhoti – Reeve 1878: pl. 3, species 19; Páll-Gergely et al. 2017: 10; Inkhavilay et al. 2019: 12, figs 4A, B.
Alycaeus (Alycaeus) mouhoti – Kobelt 1902: 347.

##### Type locality.

“Lao Mountains, Camboja”.

##### Material examined.

Lao Mountains, leg. Mouhot, NHMUK 20170120 (3 syntypes); Lao Mountains, NHMUK 1903.07.01.2715 (1 shell).

##### Remarks.

Apex matte, no spiral lines visible; R1 with rather regular, low ribs and extremely fine spiral striation (mostly visible near the suture); R2 moderately long, smooth, narrow lighter and thicker darker stripes alternating.

The tube length is variable within this species (based on Chinese and Vietnamese samples, B. Páll-Gergely, unpublished information); in some specimens its length can reach to a 0.5 whorl, whereas in others it is shorter than a 0.25 whorl.

#### Pincerna multicostulata

Taxon classificationAnimaliaGastropodaCyclophoridae

(Godwin-Austen, 1914)

52EAA7C0-6006-5A78-9FA8-8A4A76B5763F

Alycaeus (Cycloryx) multicostulatus Godwin-Austen, 1914: 404, pl. 147, fig. 7.
Alycaeus (Cycloryx) multicostulatus – Gude 1921: 282–283.
Cycloryx multicostulatus – Ramakrishna et al. 2010: 73.

##### Type locality.

“Head of the Lanier River, Lahupa Naga Hills, N.E. Manipur”.

##### Material examined.

Munipur, head of Lanier Rr, leg. Godwin-Austen, NHMUK 1903.7.1.2557 (1 syntype).

##### Remarks.

Protoconch glossy; R1 with dense, relatively low, but sharp ribs and extremely fine spiral striation; R2 very short, with ca. five ribs similar to those of R1 in terms of morphology and density.

#### Pincerna otiphorus

Taxon classificationAnimaliaGastropodaCyclophoridae

(Benson, 1859)

94DBC630-AAED-5885-9222-14D8CD76961C

Alycaeus otiphorus Benson, 1859: 178–179.
Alycaeus otiphorus – Reeve 1878: pl. 4, species 30.
Alycaeus (Alycaeus) otiphorus – Kobelt 1902: 347.
Alycaeus (Cycloryx) otiphorus – Godwin-Austen 1914: 349, pl. 147, figs 2, 2a, 2b; Gude 1921: 283.
Cycloryx otiphorus – Ramakrishna et al. 2010: 73.

##### Type locality.

“ad Pankabari (1000 ped. alt.) et in valle Rungun (4000 ped.) prope Darjiling Himalayanum”.

##### Material examined.

No locality data, UMZC I.102555 (1 syntype); Darjiling, “compared with typical sp in Museum Cambridge”, NHMUK 1903.7.1.2565 (1 shell).

##### Remarks.

The whole shell was weathered, but the smooth protoconch and the remains of regular ribs on R1 are visible; R2 very short, probably smooth.

#### Pincerna paucicostata

Taxon classificationAnimaliaGastropodaCyclophoridae

(Godwin-Austen, 1914)

050910C7-B59C-5346-8283-741D5BD063A8

Alycaeus paucicostatus Godwin-Austen, 1914: 361, pl. 147, figs 5, 5a.
Alycaeus (Cycloryx) paucicostatus – Gude 1921: 284.
Cycloryx paucicostatus – Ramakrishna et al. 2010: 73.

##### Type locality.

“Torúpútú Peak, Dafla Hills”.

##### Material examined.

Toruputu Pk, Dafla Hills, NHMUK 1903.7.1.2595 (2 syntypes).

##### Remarks.

Protoconch glossy, without spiral lines; R1 with very sharp, elevated ribs and extremely fine, but well visible spiral striation; the density of ribs decreases in an anterior direction; R2 very short, with ca. five ribs, similar to those of the end of R1 but are slightly denser.

#### Pincerna summa

Taxon classificationAnimaliaGastropodaCyclophoridae

(Godwin-Austen, 1914)

29E2B8C5-E830-5D8C-9175-BC0E6E2225D4

Alycaeus (Cycloryx) summus Godwin-Austen, 1914: 349–350, pl. 147, figs 3, 3a.
Alycaeus (Cycloryx) summus – Gude 1921: 284–285.

##### Type locality.

“Rechila Peak, Western Bhutan”.

##### Material examined.

Rechila Pk, W. Bhutan, leg. Godwin-Austen, NHMUK 1903.7.1.2573 (12 syntypes).

##### Remarks.

Protoconch matte, without spiral striation; R1 with dense, weak, irregular ribs and only slightly weaker spiral striation; R2 very short, with ca. five narrow, light stripes, the surface of R2 nearly smooth.

#### Pincerna tenella

Taxon classificationAnimaliaGastropodaCyclophoridae

(Godwin-Austen, 1914)

E1682747-E41F-5D98-AD43-3B856AD823BD

Alycaeus (Cycloryx) tenellus Godwin-Austen, 1914: 417, pl. 155, figs 3, 4, 4a.
Alycaeus (Cycloryx) tenellus – Gude 1921: 285.

##### Type locality.

“Shan States”.

##### Material examined.

Shan Hills, on same slide as N. 22, leg. Fedden, NHMUK 1906.5.5.87 (holotype [single specimen mentioned in the original description]).

##### Remarks.

The holotype was strongly weathered. Protoconch without any visible sculpture, R1 and R2 with widely spaced ribs; between the main elevated ribs there are some additional lower ribs.

#### Pincerna thieroti

Taxon classificationAnimaliaGastropodaCyclophoridae

(de Morgan, 1885)

DBB2A9F4-6310-5091-873A-6D18241AD408

Alycaeus thieroti de Morgan, 1885b: 403–404, pl. 8, fig. 4.
Alycaeus (Orthalycaeus) thieroti – Möllendorff 1891: 342.
Alycaeus (Alycaeus) thieroti – Kobelt 1902: 352.
Alycaeus thieroti – Venmans 1956: 82–83, fig. 3 (radula, see Results); Foon and Liew 2017: 69–72, figs 7D, 29, 31C.
Pincerna thieroti – Páll-Gergely 2017: 214.

##### Type locality.

“G. Lano”.

##### Material examined.

Mont Lano, Perak, coll. de Morgan, MNHN-IM-2000-31799 (1 syntype); West Malaysia: Selangor: Serendah Forest Reserve, Bukit Takun (400 m), alive on low shrubs, leg. Sow-Yan Chan, Hiong Eng Ng & Leo Nguang 27.10.1996, ex coll. Chan, 1997, SMF 311321 (5 shells; similar to the ones in the original description).

##### Remarks.

Protoconch very finely granulated, rather glossy; R1 with rather irregular, low, widely spaced ribs and very prominent spiral sculpture, which is visible on the whole shell except for the protoconch; R2 very short, consists of darker and thicker, and narrow and light stripes, which are very slightly elevated from the surface.

#### Pincerna thompsoni

Taxon classificationAnimaliaGastropodaCyclophoridae

(Godwin-Austen, 1914)

75954E0C-CDA4-50BC-BA99-E1C392490D2E

Alycaeus (Cycloryx) thompsoni Godwin-Austen, 1914: 404, pl. 146, figs 3, 3a.
Alycaeus (Cycloryx) thompsoni – Gude 1921: 285.
Cycloryx thompsoni – Ramakrishna et al. 2010: 74.

##### Type locality.

“Munipur”.

##### Material examined.

Munipur, coll. Godwin-Austen, NHMUK 1903.7.1.2550 (1 syntype).

##### Remarks.

Entire shell somewhat weathered; protoconch matte; R1 regularly, densely ribbed, spiral striation not visible, but additional, lower radial lines present between the ribs; R2 very short, ca. five ribs, similar to those on R1 in density.

This species may be conspecific with P. graphicus var. dihingensis because there is only a slight difference in the density of ribbing (coarser and more oblique in *thompsoni*). *Pincerna thompsoni* has a completely closed umbilicus, but due to the lack of large shell material we are unable to determine whether this represents individual variability or a stable character. Alternatively, the closed umbilicus may be a feature of more mature (more developed) specimens.

#### Pincerna (?) vallis

Taxon classificationAnimaliaGastropodaCyclophoridae

Z.-Y. Chen & M. Wu, 2020

D8B3E62C-E378-51A2-975A-425355781F39

Pincerna vallis Z.-Y. Chen & M. Wu, 2020: 42, figs 1, 4A, B, 5A.

##### Type locality.

“China, Hubei, Wufeng Tujiazu Autonomous County, Chaibuxi National Forest Park, 30.216N, 110.199E, 1220 m a.s.l.”.

##### Material examined.

Photographs of the holotype (HBUMM 10017-spec. 1) and the paratype (HBUMM 10017-spec. 2), were examined.

##### Remarks.

Protoconch finely granulate, without spiral striation; R1 with regularly spaced strong ribs, R2 very short, consisting of ca. 15 lighter stripes, R3 finely ribbed close to the peristome. No spiral striation mentioned in original description, but some spiral lines visible on R1.

#### Pincerna (?) vanbuensis

Taxon classificationAnimaliaGastropodaCyclophoridae

(Bavay & Dautzenberg, 1900)

24694156-78BC-5999-851D-B00F0EAE98FF

Alycaeus (Dioryx) vanbuensis Bavay & Dautzenberg, 1900a: 120.
Alycaeus (Dioryx) vanbuensis – Bavay & Dautzenberg, 1900b: 455–456, pl. 11, figs 19–21.
Dioryx vanbuensis – Kobelt 1902: 340; Varga 1972: 136, figs 24, 25.
Alycaeus vanbuensis – Páll-Gergely et al. 2017: 10, fig. 3C; Inkhavilay et al. 2019: 13, fig. 4C–E.

##### Type locality.

“Van-bu”.

##### Material examined.

Tonkin, Van-Bu, leg. Dr. R. Bavay, MNHN-IM-2000-31798 (1 syntype).

##### Remarks.

Shell shape globular; protoconch matte, R1 with regular, low, but clearly visible ribs and extremely fine spiral striation; R2 relatively long (ca. 90°), light slimmer and darker thicker stripes alternate.

#### Pincerna yanseni

Taxon classificationAnimaliaGastropodaCyclophoridae

Páll-Gergely, 2017

401E8D2B-3C21-5A20-AA65-1B1D6CD49384

Pincerna yanseni Páll-Gergely, 2017: 214–219, figs 1B, 2, 3.

##### Type locality.

“Indonesia, Sumatera Barat (West Sumatra), Solok Selatan (South Solok Regency), Koto Parik Gadang Diateh subdistrict, Goa [=cave] Pinti Kayu, near Sungai [=river] Dareh, approximate GPS position: 1.3027°S, 101.1164°E”.

##### Material examined.

Holotype (MZB.Gst. 18.970), and several paratypes: see Páll-Gergely (2017).

##### Remarks.

Protoconch glossy, first 1.75 whorls of R1 with low, relatively dense, regular ribs and weak, dense spiral striation; this sculpture gradually changes to a more sparsely ribbed region, which spans ca. 0.5 a whorl; R2 and tube very short, with eight or nine ribs very narrow, only slightly elevated from the surface; for description of the fine structure of the microtunnels, see original description.

#### Genus Stomacosmethis

Taxon classificationAnimaliaGastropodaCyclophoridae

Bollinger, 1918

901659C1-213D-5E4D-B860-FAE4787A0191

Alycaeus (Stomacosmethis) Bollinger, 1918: 316.
Alycaeus (Stomacosmethis) – Egorov 2013: 34.

##### Type species.

Alycaeus (Stomacosmethis) sarasinorum Bollinger, 1918 (Fig. 39C), SD Egorov (2013: 34).

##### Diagnosis.

Shell small to very large (D: 3–13 mm), usually brightly coloured yellow or orange; shell shape triangular or depressed triangular; protoconch smooth without spiral striation; R1 usually finely reticulated, without prominent ribs; R2 very short, usually roughly, weakly wrinkled, R3 long, with blunt swelling. Operculum thin, outer surface smooth or with outer, elevated, trumpet-like projection. The outer operculum surface can also be finely granulated, flaky or have short calcareous spikes (Foon and Liew 2017). Central radular tooth elongated, usually with a single central cusp only, or central cusps with one or two small cusp at each side.

##### Differential diagnosis.

Most species classified in this genus were previously described in *Alycaeus*. However, *Alycaeus* species are characterised by a very long R2, whereas the R2 is extremely short is *Stomacosmethis*. *Pincerna* species possess a globular shell with more widely spaced ribs, and they are usually not colourful. The distinction of *Stomacosmethis* and *Pincerna* requires further examination. See also under *Pincerna* (p. 147).

##### Distribution.

This genus is distributed in the tropical forests of the Malay Archipelago (Malay Peninsula, Borneo, Java, Sumatra, Sulawesi).

##### Remarks.

This genus was originally diagnosed based on the calcareous, pipe, tongue or cup-shaped structure on the outer side of the operculum. Since the circular opercular structures have evolved multiple times, this genus must be re-diagnosed. The type species belongs to a clearly delineable group of the Alycaeidae having triangular, colourful shells, dense, fine ribs and short tube, which is now called *Stomacosmethis*. See also under *S. balingensis*, which is exceptional in terms of shell shape.

#### Stomacosmethis altispirus

Taxon classificationAnimaliaGastropodaCyclophoridae

(Möllendorff, 1902)

385CA130-89AC-5457-9C2C-A8E6A0EEBDD5

Alycaeus perakensis altispirus Möllendorff, 1902: 144–145.
Alycaeus (Alycaeus) perakensis altispirus – Zilch 1957: 147, pl. 6, fig. 32.
Alycaeus altispirus – Foon and Liew 2017: 21–23, figs 7A, B, 10, 31A.

##### Type locality.

“Kelantan, Ostküste der Halbinsel Malacca” (from the title).

##### Material examined.

Malakka, Kelantan, coll. Möllendorff, SMF 109738 (lectotype, designated by Zilch 1957).

##### Remarks.

Protoconch very finely granulated, without spiral lines; R1 with irregular, low ribs and very weak spiral striation in-between, approximately same strength; R2 very short, with darker thick and fewer than ten lighter narrower stripes very slightly elevated from the surface.

This taxon was described as a subspecies of *A. perakensis*. We agree with Foon and Liew (2017) that it should be handled as a species due to the characteristic flat whorls and expanded peristome.

#### Stomacosmethis balingensis

Taxon classificationAnimaliaGastropodaCyclophoridae

(Tomlin, 1948)

92CA0759-75AA-5E0D-9364-6B535431B48B

Alycaeus balingensis Tomlin, 1948: 224–225, pl. 1, fig. 2 (incorrectly cited as fig. 3).
Alycaeus balingensis – Foon and Liew 2017: 21–23, figs 7A, B, 10, 31A.

##### Type locality.

“Bukit Baling, Kedah, rather common”.

##### Material examined.

Bukit Baling, Kedah, leg. Tweedie, Dec. 1938, NHMUK 1948.10.2.4 (1 syntype).

##### Remarks.

Protoconch matte, without spiral striation; R1 with regular, dense riblets which become sparser anteriorly; R2 extremely short, with low, blunt ribs; R3 with widely spaced, regular, strong but low ribs.

The globular shell shape suggests that this species belongs to *Pincerna*. However, the dense ribbing would be unusual in *Pincerna*, and the radular morphology is identical with that of *S. perakensis*.

#### Stomacosmethis calopoma

Taxon classificationAnimaliaGastropodaCyclophoridae

(E. von Martens, 1908)

D36413FC-D06F-5524-B53B-865BDF07E770

Alycaeus calopoma E. von Martens, 1908: 279, pl. 5, fig. 16.
Alycaeus callopoma (sic) – Tielecke 1940: 345.

##### Type locality.

“Gunung Sekerat, Sekuran, an Kalkfelsen”.

##### Material examined.

Gunung Sekurat, Sangana, Fanso, leg. M. Schmidt, ZMB/MOLL 109931.

##### Remarks.

We received photographs of the operculum of the type specimen but were unable to examine the shell. This species is classified in *Stomacosmethis* based on the illustrations in the original description.

#### Stomacosmethis carinatus

Taxon classificationAnimaliaGastropodaCyclophoridae

(Maassen, 2006)

5069C3AF-F9C8-5D8B-B7A1-2F59FDE3B410

Alycaeus carinata Maassen, 2006: 137–138, figs 10–13.
Alycaeus carinata – Kittel et al. 2012: 59, fig. 9B; Foon and Liew 2017: 24–25, figs 11, 31Q.

##### Type locality.

“Malaysia, Pahang, Bukit Sagu, NW. of Kuantan”.

##### Material examined.

Malaysia, Pahang, Bukit Tengkek (nw of Kuantan), Ptani, October 1998, ex coll. Hemmen, coll. PGB (2 paratypes).

##### Remarks.

Protoconch elevated (although the spire is low), matte, no spiral lines visible; R1 very roughly wrinkled with weak spiral striation; R2 extremely short, with alternating darker/thicker and lighter/narrower stripes; lighter stripes elevated from surface, overall surface roughly wrinkled.

#### Stomacosmethis christae

Taxon classificationAnimaliaGastropodaCyclophoridae

(Maassen, 2006)

0E9D60C7-1B38-500A-84CC-837AB745CFF3

Alycaeus christae Maassen, 2006: 136, figs 1–5.
Alycaeus christae – Kittel et al. 2012: 59, fig. 9A.

##### Type locality.

“Thailand, Krabi Province, at km 117.6 of road # 4 (Krabi – Phang Nga)”, 1.5 km on unpaved road, 08°09.574'N, 098°51.761'E”.

##### Material examined.

Malaysia, Krabi Prov., at km 117.6 off rd. #4 (Krabi to Phang Nga), 1.5 km on unpaved road, 14.09.2000, ex coll. Hemmen, coll. PGB (1 paratype; note that Malaysia is erroneous on the label of this specimen).

##### Remarks.

Protoconch finely granulated without spiral lines; R1 regularly and finely ribbed with even finer spiral striations; R2 short, with low sharp ribs (cross sectional view is triangular).

#### Stomacosmethis congener

Taxon classificationAnimaliaGastropodaCyclophoridae

(E. A. Smith, 1895)

0A0701BB-FA2E-5DC2-873B-016A58C27C19

Fig. 42A

Alycaeus (Orthalycaeus) congener E. A. Smith, 1895: pl. 3, fig. 26.
Alycaeus (Alycaeus) congener – Kobelt 1902: 342.

##### Type locality.

“Mulu and Barit Mountains, Sarawak”.

##### Material examined.

Barriet Mt, N. Borneo, NHMUK 1892.7.20.30–32 (3 syntypes); Molu, N. Borneo, coll. Dr. A. Oberwimmer, NHMW 71640/O/12201 (1 shell).

##### Remarks.

Protoconch matte, without spiral lines; R1 with rather irregular ribs and much finer spiral striae; R2 very short, with alternating wider/darker and narrower/lighter stripes; lighter stripes somewhat elevate from the surface.

**Figure 42.**
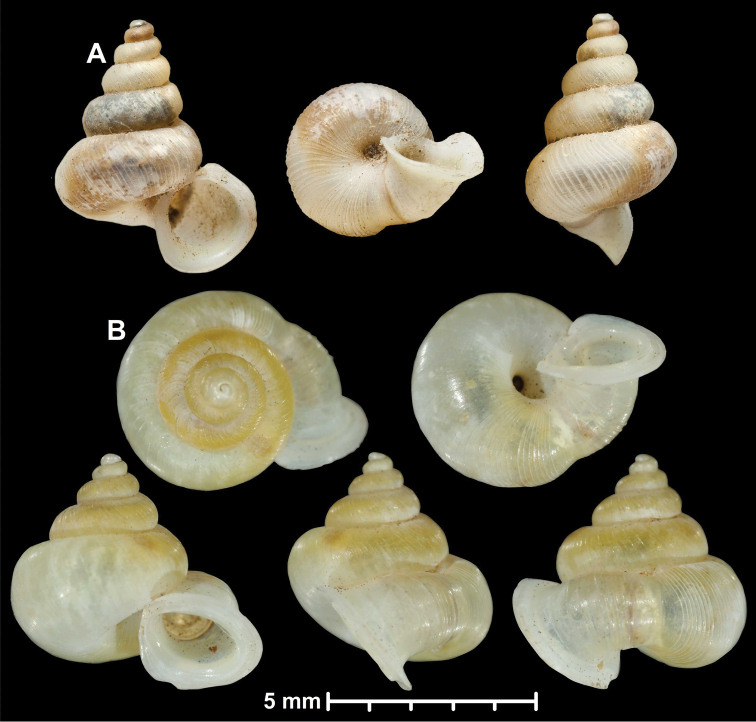
Shells of *Stomacosmethis* Bollinger, 1918 species **A***Stomacosmethis congener* (E. A. Smith, 1895), syntype (NHMUK 1892.7.20.30–32) **B***Stomacosmethis dohrni* (O. Boettger, 1893), lectotype (SMF 109278). Figures: Barna Páll-Gergely, courtesy Ronald Janssen (**B**) and Harold Taylor (**A**).

#### Stomacosmethis dohrni

Taxon classificationAnimaliaGastropodaCyclophoridae

(O. Boettger, 1893)

B080F238-1B1D-5084-9435-A2F7802CB0B7

Fig. 42B

Alycaeus hochstetteri (partim) – E. von Martens, 1867: 152.
Alycaeus dohrni – Boettger 1893: 195–196.
Alycaeus (Alycaeus) dohrni – Kobelt 1902: 343; Zilch 1957: 146–147, pl. 6, fig. 28.

##### Type locality.

“Borneo”.

##### Material examined.

Borneo, leg. H. Dohrn 1889, coll. O. Boettger, SMF 109278 (lectotype, designated by Zilch 1957); Same data, SMF 109279 (paralectotype).

##### Remarks.

Protoconch very finely granulated, without spiral striae; R1 with irregular, rather strong ribs with very slight indication of spiral striation; R2 very short, with alternating darker/thicker and lighter/slimmer stripes; lighter stripes are somewhat elevated from the surface, the entire area is finely wrinkled.

#### Stomacosmethis fultoni

Taxon classificationAnimaliaGastropodaCyclophoridae

(Möllendorff, 1896)

8CA91F61-C068-5FB8-8FA1-DD619FA65108

Fig. 43A

Alycaeus (Orthalycaeus) fultoni Möllendorff in E. A. Smith, 1895: 117–118, pl. 3, fig. 28.
Alycaeus (Alycaeus) fultoni – Kobelt 1902: 343–344; Zilch 1957: 146–147, pl. 6, fig. 29.

##### Type locality.

“prope Gomanton insulæ Borneo”.

##### Material examined.

N-Borneo, Gomanton, coll. Möllendorff, SMF 109280 (lectotype, designated by Zilch 1957); Same data, SMF 109281 (2 paralectotypes); Borneo, ex coll. Fulton, NHMW 21002 (2 shells).

##### Remarks.

Protoconch without spiral lines, rather matte; R1 with irregular ribs and very weak spiral lines; R2 very short, with darker/wider and lighter/narrower stripes; lighter stripes are elevated from the surface, the overall surface is seemingly irregularly wrinkled.

**Figure 43.**
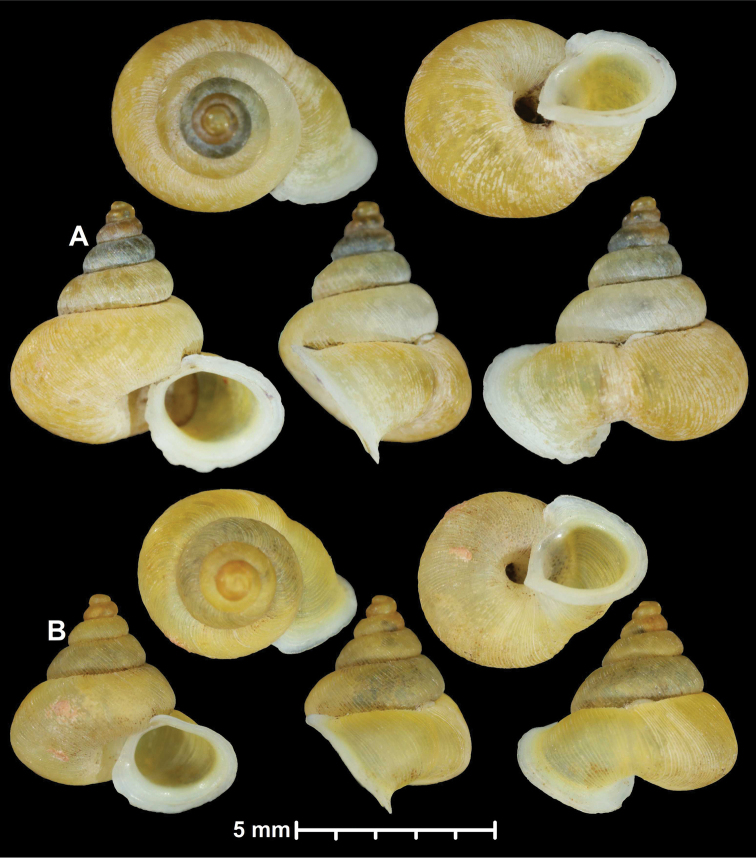
Shells of *Stomacosmethis* Bollinger, 1918 species **A***Stomacosmethis fultoni* (Möllendorff, 1896), lectotype (SMF 109280) **B***Stomacosmethis fultoni degenerans* (Fulton, 1901), lectotype (SMF 109282). All figures: Barna Páll-Gergely, courtesy Ronald Janssen.

#### Stomacosmethis fultoni degenerans

Taxon classificationAnimaliaGastropodaCyclophoridae

(Fulton, 1901)

D1338DB3-4C60-514C-877F-9121E8EFEFFB

Fig. 43B)

Alycaeus fultoni var. degenerans Fulton, 1901: 242.
Alycaeus (Alycaeus) fultoni degenerans – Zilch 1957: 147, pl. 6, fig. 30.

##### Type locality.

“Gomanton, N. Borneo”.

##### Material examined.

N-Borneo, Gomanton, leg. Fulton, coll. Möllendorff, SMF 109280 (lectotype, designated by Zilch 1957); Same data, SMF 109281 (2 paralectotypes).

##### Remarks.

Protoconch without spiral lines, rather matte; R1 with rather regular, strong, sharp ribs and very weak spiral lines; R2 very short, with alternating darker/wider and lighter/narrower stripes; lighter stripes are very slightly elevated from the surface.

#### Stomacosmethis galbanus

Taxon classificationAnimaliaGastropodaCyclophoridae

(Godwin-Austen, 1889)

8F9F3109-02CD-5FC3-9557-1CF69BF53187

Alycaeus galbanus Godwin-Austen, 1889: 346, pl. 37, figs 1, 1a.
Alycaeus (Alycaeus) galbanus – Kobelt 1902: 344.

##### Type locality.

“Niah Hills”.

##### Material examined.

Type specimens not found in the NHM. Niah-Berge, Borneo, coll. Rušnov ex coll. W. Blume, NHMW 71770/R/20 (2 shells).

##### Remarks.

Protoconch without spiral lines, matte; R1 with irregular, rather strong ribs and very weak spiral lines; R2 very short, with alternating darker/wider and lighter/narrower stripes; lighter stripes are elevated from the surface, the overall surface is seemingly irregularly wrinkled.

#### Stomacosmethis hosei

Taxon classificationAnimaliaGastropodaCyclophoridae

(Godwin-Austen, 1889)

D79E2F3B-EBF9-5859-B4FF-BD01FEBD5487

Alycaeus hosei Godwin-Austen, 1889: 347, pl. 37, fig. 2.
Alycaeus hosei – E. A. Smith 1895: 117.
Alycaeus (Alycaeus) hosei – Kobelt 1902: 346.

##### Type locality.

“Busan Hills”.

##### Material examined.

Borneo, ex coll. Fulton, NHMW 21004 (3 shells).

##### Remarks.

Protoconch without spiral lines; R1 with rather regular, weak ribs and some indication of weak spiral striae; R2 very short, with darker wider and lighter slimmer stripes alternating; lighter stripes are slightly elevated from the surface.

#### Stomacosmethis jagori

Taxon classificationAnimaliaGastropodaCyclophoridae

(E. von Martens, 1860)

618A9F76-EDBB-5934-BF8D-EF084494A29A

Alycaeus jagori E. von Martens, 1860: 208.
Alycaeus Hochstetteri L. Pfeiffer, 1861: 215, pl. 3, figs 1–4.
Alycaeus jagori – L. Pfeiffer 1861: 215, pl. 3, figs 5–7; E. von Martens 1867: 152–153; Sarasin P. & Sarasin F. 1899: 61–62, pl. 4, figs 46, 46a, pl. 5, fig. 66, pl. 8, fig. 91; Tielecke 1940: 345; Benthem Jutting 1948: 568–570, fig. 25.
Alycaeus fugori [sic] – Reeve 1878: pl. 6, species 50.
Alycaeus jagori var. minor E. von Martens, 1867: 152.
Alycaeus hochstetteri (partim) – E. von Martens 1867: 152.
Alycaeus (Alycaeus) jagori – Kobelt 1902: 346; Bollinger 1918: 314; Zilch 1957: 147.
Alycaeus (Alycaeus) hochstetteri – Kobelt 1902: 345–346; Bollinger 1918: 314, pl. 11, fig. 7.

##### Type locality.

“Jagor aus Java” (*A. jagori*); “in montibus Nungnang insulae Javae” (*A. hochstetteri*); “südl. Celebes, bei Maros unweit Makassar” (A. jagori var. minor).

##### Material examined.

Java. Jagor, coll. Möllendorff ex coll. Martens, SMF 109304 (1 syntype of *A. jagori*, photographs examined); Java. coll. Rušnov ex coll. W. Blume, NHMW/71770/R/21 (18 shells).

##### Remarks.

Benthem-Jutting (1948) claimed that *Alycaeus hochstetteri* is a synonym of *A. jagori*. Neither she, nor we had a chance to examine the type specimens of *A. hochstetteri*, but we follow Benthem-Jutting’s view, which is based on that of Pfeiffer (1861). Hendriks et al. (2019) studied the phylogeography of *S. jagori* from Borneo. Given that the species is described from Java, the identity of the Bornean samples needs to be revised.

Protoconch glossy, without spiral lines; R1 with irregular, low ribs, very weak spiral striation in-between; R2 very short, with alternating darker/thicker and lighter/narrower stripes somewhat elevated from the surface; overall surface of R2 irregularly wrinkled.

#### Stomacosmethis kapayanensis alticola

Taxon classificationAnimaliaGastropodaCyclophoridae

(Foon & Liew, 2017)

1BEAF3C4-4F98-5562-8D91-640BDC61616E

Alycaeus alticola Foon & Liew, 2017: 16–19, figs 7J, 8, 31G.

##### Type locality.

“PHG 77 Bukit Mengapur, Pahang (3°44'42"N, 102°50'16"E)”.

##### Remarks.

According to the original description this taxon differs from *A. charasensis*, *A. costacrassa*, *A. selangoriensis*, and *A. kapayanensis* in the formation of the body whorl and in slight differences in the shell sculpture. However, the differences in shell shape are not obviously visible on the photographs in Foon and Liew (2017). Instead, only very slight differences (if any) are to be seen, which we feel do not represent distinctive character traits on a species level. Therefore, we treat this taxon as a subspecies of *S. kapayanensis*.

#### Stomacosmethis kapayanensis charasensis

Taxon classificationAnimaliaGastropodaCyclophoridae

(Foon & Liew, 2017)

762699AE-28AD-533D-9B6D-247A21BEA32D

Alycaeus charasensis Foon & Liew, 2017: 25–27, Figs 7K, 12, 31H.

##### Type locality.

“PHG 73 Bukit Charas, Pahang (3°54'35"N, 103°08'48"E)”.

##### Remarks.

This taxon differs in minor traits (extent of the body whorl and strength of the sculpture) from *S. kapayanensis* and some other “species” described by Foon and Liew, therefore it is treated as a subspecies of *S. kapayanensis*.

#### Stomacosmethis kapayanensis costacrassa

Taxon classificationAnimaliaGastropodaCyclophoridae

(Foon & Liew, 2017)

22670DCD-EC33-5FD0-ADA9-541FF6302FE8

Alycaeus costacrassa Foon & Liew, 2017: 33–35, figs 7M, 15, 31N.

##### Type locality.

“Mykarst-065 Batu Balong, Pahang (3°42'41"N, 101°51'25"E)”.

##### Remarks.

This taxon differs from *Alycaeus kapayanensis selangoriensis* and *Stomacosmethis kapayanensis kapayanensis* in characters of the operculum and shell sculpture, formation of the peristome and the body whorl. We believe however, that such characters are of minor taxonomic importance, and therefore, *Alycaeus costacrassa* is here considered a subspecies of *S. kapayanensis*.

#### Stomacosmethis kapayanensis ikanensis

Taxon classificationAnimaliaGastropodaCyclophoridae

(Foon & Liew, 2017)

BD95AB61-0075-5B9F-971F-6F42DEBE204A

Alycaeus ikanensis Foon & Liew, 2017: 41–43, figs 18, 31L.

##### Type locality.

“KTN 06 Gua Ikan, Kelantan (5°21'14"N, 102°01'33"E)”.

##### Remarks.

*Alycaeus ikanensis* is similar to the other taxa described by Foon and Liew, which are treated here as subspecies of *Stomacosmethis kapayanensis*. The original description states that *A. ikanensis* is most similar to *A. costacrassa* and differs from that species in its smaller and more slender shell. Again, these traits are enough for subspecific distinction, and slight differences in size and shell shape are insufficient for differences at the species level.

#### Stomacosmethis kapayanensis

Taxon classificationAnimaliaGastropodaCyclophoridae

(de Morgan, 1885)

1DC21AA3-1F5D-5D63-ACA0-1C03C8B57F2D

Alycaeus kapayanensis de Morgan, 1885b: 403, pl. 8, fig. 5.
Alycaeus (Orthalycaeus) kapayensis (sic) – Möllendorff 1891: 342.
Alycaeus (Alycaeus) kapayanensis – Kobelt 1902: 346.
Alycaeus kapayanensis – Foon and Liew 2017: 46–49, figs 7O, 20, 31M.

##### Type locality.

“rochers calcaires du G. Lano”.

##### Material examined.

G. Laon, Perak, MNHN-IM-2000-31792 (1 syntype).

##### Remarks.

Protoconch extremely finely granulated, matte; R1 rather irregularly, finely ribbed with weaker spiral striation; R2 short, with alternating darker/thicker and lighter/slimmer stripes, which are somewhat elevated from the surface, the whole area is rather irregularly sculptured.

Foon and Liew (2017) described six new species from Peninsular Malaysia, which are similar to *S. kapayanensis* in all the important characters, and show only minor differences, such as fine sculpture (e.g., strength of radial vs. spiral striation), operculum thickness, and shell size and shape. Here we maintain those species (*alticola*, *charasensis*, *costacrassa*, *ikanensis*, *kurauensis*, *selangoriensis* and *virgogravida*) as subspecies of *S. kapayanensis*.

#### Stomacosmethis kapayanensis kurauensis

Taxon classificationAnimaliaGastropodaCyclophoridae

(Foon & Liew, 2017)

A1F6400D-174E-5D61-A83C-B2DC997657AF

Alycaeus kurauensis Foon & Liew, 2017: 52–54, figs 7R, 22, 31P.

##### Type locality.

“PRK 59 Bukit Batu Kurau, Perak (4°55'45"N, 100°49'02"E)”.

##### Remarks.

According to the original description, *Alycaeus kurauensis* differs from *Stomacosmethis kapayanensis* in “having a wider ultimate whorl, thicker operculum with smooth exterior and more widely spaced radial ribs.” These traits, in our opinion, are sufficient for distinction on subspecific level, but do not justify the distinctness at the species level. Therefore, this taxon is handled as a subspecies of *S. kapayanensis*.

#### Stomacosmethis kapayanensis selangoriensis

Taxon classificationAnimaliaGastropodaCyclophoridae

(Foon & Liew, 2017)

D60A0AB4-4CB4-5AA4-9EBA-FD2DE18D0A53

Alycaeus selangoriensis Foon & Liew, 2017: 64–66, figs 7U, 27, 31O.
Alycaeus kapayanensis – Venmans 1956: 83–84, figs 4, 5 (radula, see Results on radula, p. 12).

##### Type locality.

“SGR 01 Batu Caves, Selangor (3°14'17"N, 101°41'02"E)”.

##### Remarks.

This taxon differs from *S. kapayanensis* in the larger shell, more expanded body whorl, thicker operculum, and the formation of the upper palatal section of the peristome. These differences seem to justify differentiation at the subspecific level only.

#### Stomacosmethis kapayanensis virgogravida

Taxon classificationAnimaliaGastropodaCyclophoridae

(Foon & Liew, 2017)

C1403BD2-7741-5B1C-983D-CB9E114E646B

Alycaeus virgogravida Foon & Liew, 2017: 72–75, figs 30, 31S.

##### Type locality.

“Limestone hill at east side of Pulau Dayang Bunting, off Langkawi Island, Kedah (6°12'26"N, 99°47'04"E)”.

##### Remarks.

This taxon differs from *S. kapayanensis* and its subspecies in only minor traits (shell and operculum sculpture, formation of whorls). Although described as a species in its own right, we suggest using it as a subspecies of *S. kapayanensis*.

#### Stomacosmethis kelantanensis clementsi

Taxon classificationAnimaliaGastropodaCyclophoridae

(Foon & Liew, 2017)

E7476CEA-302F-5899-877D-7316C069CFDE

Alycaeus clementsi Foon & Liew, 2017: 28–30, figs 7L, 13, 31J

##### Type locality.

“Gua Kelam, PRS 64 Wang Ulu, Perlis (6°38'41"N, 100°12'09"E)”.

##### Remarks.

*Alycaeus clementsi* is very similar to *Stomacosmethis kelantanensis* in general shells shape and sculpture. The differences mentioned in the original description (shell size, peristome thickness, outer surface of the operculum, animal colour) are sufficient to distinguish them at the subspecific level only.

#### Stomacosmethis kelantanensis expansus

Taxon classificationAnimaliaGastropodaCyclophoridae

(Foon & Liew, 2017)

CAF4A79D-CD17-5038-A60A-A078B1C1ED83

Alycaeus expansus Foon & Liew, 2017: 25–37, figs 7N, 16, 31I.

##### Remarks.

*Alycaeus expansus* Foon & Liew is most similar to *A. clementsi* and *Stomacosmethis kelantanensis*. Foon and Liew (2017) do not explain the differences between *A. expansus* and the other two taxa, but mention that the characteristic features of this species are the obtuse ultimate whorl, the strongly expanded peristome, the thick operculum and the animal colouration. Based on the photographs in the original description it is difficult to understand what the authors meant by “obtuse ultimate whorl”, because the general shell and body whorl shape do not differ conspicuously from the other two taxa; it was supposed to mean the more rounded-looking ultimate whorl, especially for shells from the type locality (Junn Kitt Foon, pers. comm. 2020 June). Contrary to the original description, the peristome of *S. kelantanensis* is even more expanded than that of *A. expansus*. The animal colouration and the operculum thickness are characters which should not be used as distinguishing characters at the species level, especially because thickness of shell or operculum is highly dependent on the environment. Therefore, this taxon is treated as a subspecies of *S. kelantanensis*.

*Alycaeus expansus* Foon & Liew, 2017 is a primary homonym of *Alycaeus expansus* Heude, 1890. To our knowledge, the older name has not been used in this combination since 1899, thus no replacement name for *Alycaeus expansus* Foon & Liew, 2017 is necessary.

#### Stomacosmethis kelantanensis

Taxon classificationAnimaliaGastropodaCyclophoridae

(Sykes, 1902)

63537C59-1324-5827-8065-C427D80F71C0

Alycaeus kelantanense Sykes, 1902: 62, pl. 3, figs 13, 14.
Alycaeus (Orthalycaeus) kelantanensis – Möllendorff 1902: 145–146.
Alycaeus kelantanensis – Foon and Liew 2017: 49–52, figs 7P, Q, 21, 31K.

##### Type locality.

“Kelantan, Malay Peninsula” (from the title).

##### Material examined.

Kelantan, NHMUK 20160061 (1 syntype); Kelantan, coll. Möllendorff, SMF 158410 (6 shells); Malaysia, Kelantan, rd. #8 Gua Musang, Kuala Lipis, left side off km19, ex coll. Hemmen, coll. PGB (4 shells; they are similar to the syntype but narrower).

##### Remarks.

Protoconch spirally striated at its very beginning and this sculpture turns to a rather finely granulated surface until the end of the protoconch (in case of *Metalycaeus* species the first 0.5–1.0 whorl is usually without spiral lines); R1 rather irregularly ribbed with somewhat weaker spiral striation; R2 very short, with ca. seven low ribs. The SMF 158410 sample contains three shells with prominent spiral structure and another three shells with only very slight indication of spiral lines.

#### Stomacosmethis kuekenthali

Taxon classificationAnimaliaGastropodaCyclophoridae

(P. Sarasin & F. Sarasin, 1899)

9245C1E2-8595-5B85-A2BE-42524A6DCC8F

Fig. 44

Alycaeus kuekenthali P. Sarasin & F. Sarasin, 1899: 62–63, pl. 4, figs 47, 47a, pl. 5, figs 67a, 68, pl. 8, fig. 92.
Alycaeus (Chamalycaeus) kuekenthali – Kobelt 1902: 357–358.

##### Type locality.

“Kalkgrotten von Barabatuwa, nördlich von Maros, Süd-Celebes”.

##### Material examined.

Selatan Barabatuwa, Insel Sulawesi (=Celebes), in lime cave (Kalkgrotten), leg. Sarasin, Fritz + Paul, 1893–1896, NMB 02266a (lectotype, designated herein, photographs examined); S. Celebes, E.R. Sykes coll. 1954, Acc. 1825, NHMUK (1 shell, agrees with the original description and figure).

##### Remarks.

Protoconch matte; R1 with irregular, low wrinkles and extremely fine spiral striation on the first 1–1.5 whorls; R2 very short, with alternating lighter/slimmer, and darker/thicker stripes, the lighter stripes slightly elevated from the surface; operculum with a very strongly elevated, expanded, trumpet-like structure.

Lothar Forcart selected a specimen (NMB 02266a) and labelled it as lectotype, but never published this action (Ambros Hänggi, pers. comm. 2020 June). Thus, here we designate the specimen selected by him as the lectotype.

**Figure 44.**
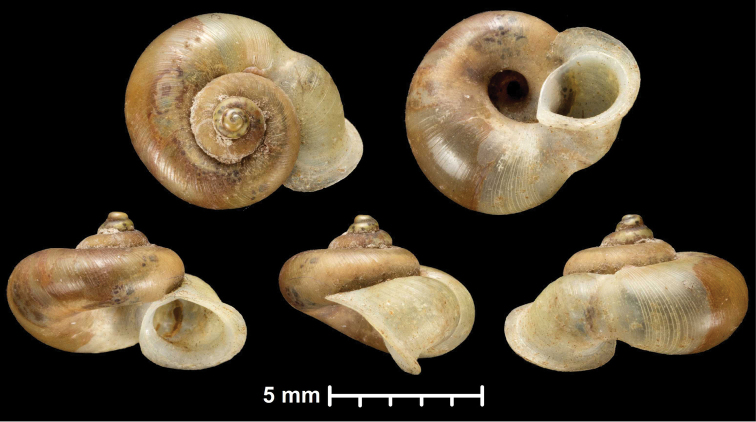
*Stomacosmethis kuekenthali* (P. Sarasin & F. Sarasin, 1899) (NHMUK 20160313). Photographs: Harold Taylor (NHM).

#### Stomacosmethis matchacheepiorum

Taxon classificationAnimaliaGastropodaCyclophoridae

(Dumrongrojwattana & Maassen, 2008)

93919383-01C7-56CB-8700-E20CF9829AF3

Alycaeus matchacheepiorum Dumrongrojwattana & Maassen, 2008: 3–4, figs 7–12.

##### Type locality.

“Thailand, Khao Pratun Cave, an isolated limestone hill in Royong Province at 13°07'19"N, 101°36'03"E, 55 meters elevation”.

##### Remarks.

We had no opportunity to examine shells of *Alycaeus matchacheepiorum*. However, the original description is sufficient for correct generic placement. The protoconch is smooth, R2 very short and the entire teleoconch is finely ribbed.

#### Stomacosmethis perakensis

Taxon classificationAnimaliaGastropodaCyclophoridae

(Crosse, 1879)

D7ADB90B-3558-5F80-B633-76EEFCB75DAD

Alycaeus perakensis Crosse, 1879: 206–208, pl. 12, fig. 7.
Alycaeus (Orthalycaeus) perakensis – Möllendorff 1891: 342.
Alycaeus (Alycaeus) perakensis – Kobelt 1902: 348.
Alycaeus perakensis – Tielecke 1940: 345; Foon and Liew 2017: 56–59, figs 7S, 24, 31V.

##### Type locality.

“Buket Pondong, Perak”.

##### Material examined.

Perak, Buket Pondong, MNHN-IM-2000-31793 (5 syntypes); Perak, NHMW 41007 (3 shells).

##### Remarks.

Protoconch glossy, without spiral lines; R1 with irregular, rather low ribs and very weak spiral striation in-between, but at the end of the region the ribs are more elevated, sharp; R2 very short, with alternating darker/thicker and lighter/narrower stripes which are somewhat elevated from the surface.

*Stomacosmethis roebeleni* may be a subspecies of *A. perakensis* although we refrain from treating it as such without further evaluation.

#### Stomacosmethis porcilliferus

Taxon classificationAnimaliaGastropodaCyclophoridae

(Bollinger, 1918)

104ED283-8882-523A-ACE2-7E0DF1180FE3

Alycaeus (Stomacosmethis) porcilliferus Bollinger, 1918: 317–318, pl. 11, figs 6, 9.

##### Type locality.

“Am Gumung-Sekerat, nahe Tandjong Kutei, O.-Borneo”.

##### Material examined.

O. Borneo, Tandjong, Kutei, don.: Prof. C. Schmidt, 1902, NHMB 2412-a (syntype [the number of available shells were not mentioned in the original description], labelled as holotype and also, lectotype, photographs examined).

##### Remarks.

Protoconch matte, no notable sculpture visible; R1 with rather regular, low ribs and very fine spiral striation; R2 short, with ca. 11 white, low, regular ribs. No operculum was found.

#### Stomacosmethis praetextus

Taxon classificationAnimaliaGastropodaCyclophoridae

(van Benthem Jutting, 1959)

3897DEA5-0CF0-5E1C-861F-557A3BD604E1

Fig. 45A

Alycaeus praetextus van Benthem Jutting, 1959: 78–79, pl. 1, fig. 1.

##### Type locality.

“Batu Sangkarnear Pajakombo, Padang Highlands”.

##### Material examined.

Sumatra: Batew Sangkar, Pajakumbok, leg. Dr. Meyer, 13.12.1955, SMF 186532 (4 paratypes).

##### Remarks.

Protoconch very finely granulated, matte; R1 with rather irregular ribs and somewhat weaker spiral striation; R2 very short, with alternating thicker/darker and narrower/lighter stripes, which are slightly elevated from the surface.

**Figure 45.**
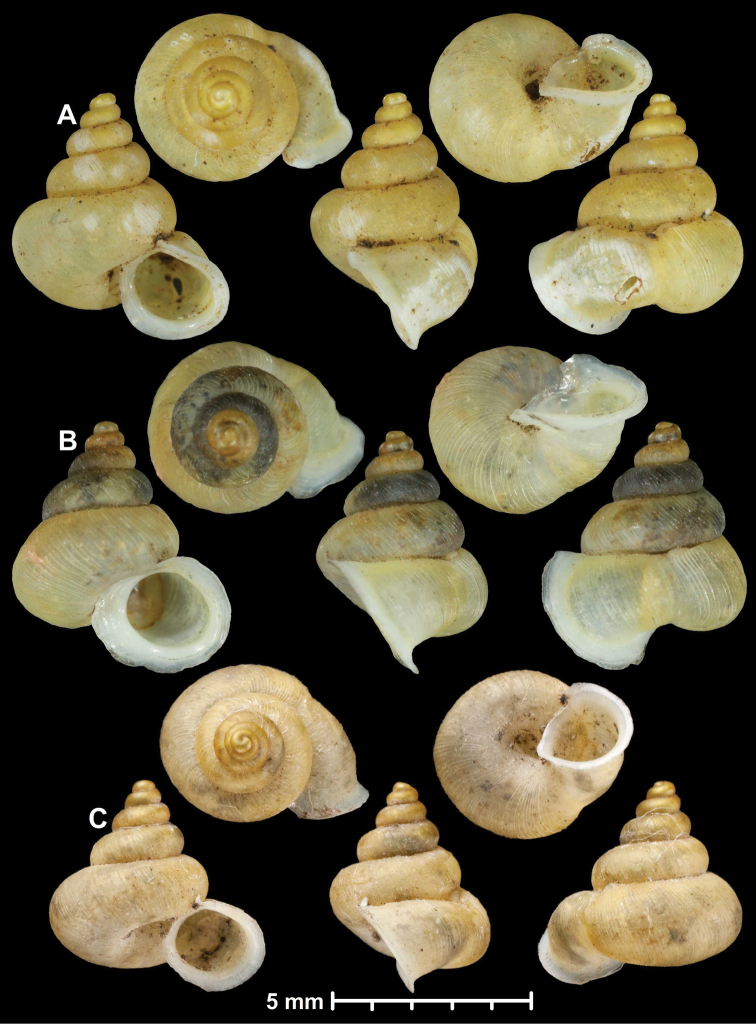
Shells of *Stomacosmethis* Bollinger, 1918 species **A***S. praetextus* (van Benthem Jutting, 1959), paratype (SMF 186532) **B***S. rimatus* (O. Boettger, 1893), lectotype (SMF 109315) **C***Stomacosmethis sadongensis* (E. A. Smith, 1895), syntype (NHMUK 1893.6.7.73–76). Photographs: Barna Páll-Gergely, courtesy Ronald Janssen (**A, B**), Harold Taylor (**C**).

#### Stomacosmethis regalis

Taxon classificationAnimaliaGastropodaCyclophoridae

(Foon & Liew, 2017)

9B79A843-6C9C-5517-9683-8C7C47EDB6BE

Alycaeus regalis Foon & Liew, 2017: 59–62, figs 7T, 25, 31T.

##### Type locality.

“PHG 02 Gunung Senyum, Pahang (3°41'50"N, 102°26'04"E)”.

##### Remarks.

Seems to be a “good” species based on the original description, morphologically distinct from all other congeners. The short tube and triangular, coloured (bright yellow) shell fits with the relevant characters for this genus.

#### Stomacosmethis rimatus

Taxon classificationAnimaliaGastropodaCyclophoridae

(O. Boettger, 1893)

F6C26C39-7D34-599F-9DD3-1327D4A3FEDB

Fig. 45B

Alycaeus rimatus Boettger, 1893: 196–197.
Alycaeus (Alycaeus) rimatus – Kobelt 1902: 350; Zilch 1957: 147, pl. 6, fig. 31.

##### Type locality.

“Brunei, W.-Borneo”.

##### Material examined.

N-Borneo, Brunei, leg. O. Staudinger, coll. O. Boettger, SMF 109315 (lectotype, designated by Zilch 1957); Same data, SMF 109316 (2 paralectotypes).

##### Remarks.

Protoconch very finely granulated, matte; R1 with irregular, strong ribs and much weaker spiral striation; R2 very short, with alternating darker/thicker and lighter/slimmer stripes, which are slightly elevated from the surface.

#### Stomacosmethis roebeleni

Taxon classificationAnimaliaGastropodaCyclophoridae

(Möllendorff, 1894)

B2510A99-9483-5883-8995-7ADEE863E775

Alycaeus roebeleni Möllendorff, 1894: 154, pl. 16, figs 20, 21.
Alycaeus (Alycaeus) roebeleni – Kobelt 1902: 350; Zilch 1957: 147, pl. 6, fig. 27.
Alycaeus roebeleni – Foon and Liew 2017: 62–64, figs 26, 31W.

##### Type locality.

“Samui Islands, Gulf of Siam” (from the title).

##### Material examined.

Golf von Siam, Koh-Samui, coll. Möllendorff, SMF 109317 (lectotype, designated by Zilch 1957); Siam, Samui, coll. Möllendorff, NHMW 40508 (13 shells, probably syntypes).

##### Remarks.

Protoconch elevated, no spiral lines visible; R1 with rather irregular, fine ribs, and somewhat weaker, dense spiral striae; R2 short, with roughly ten thicker/darker stripes, and much narrower/lighter stripes in-between, which are elevated from the surface.

#### Stomacosmethis sadongensis

Taxon classificationAnimaliaGastropodaCyclophoridae

(E. A. Smith, 1895)

30CA21A1-4705-59A7-9C68-28B6484D73D4

Fig. 45C

Alycaeus (Orthalycaeus) sadongensis E. A. Smith, 1895: 117, pl. 3, fig. 27.
Alycaeus (Alycaeus) sadongensis – Kobelt 1902: 350–351.

##### Type locality.

“Sadong, Sarawak”.

##### Material examined.

Sadong, Sarawak, NHMUK 1893.6.7.73–76 (4 syntypes).

##### Remarks.

Protoconch elevated, matte, without spiral striation; R1 with dense, regular, rather low riblets and very weak spiral striae; R2 very short, with 6–7 darker/thicker stripes and very narrow/light stripes in-between, the ribs are very slightly elevated from the surface.

#### Stomacosmethis sarasinorum

Taxon classificationAnimaliaGastropodaCyclophoridae

(Bollinger, 1918)

CE0D4D19-FD7D-58F0-9B68-C8EB3E49F06A

Fig. 39C

Alycaeus (Stomacosmethis) sarasinorum Bollinger, 1918: 316–317, pl. 11, figs 4, 5, 8.
Alycaeus (Stomacosmethis) sarasinorum – Egorov 2013: 34, figs 60a, b.

##### Type locality.

“Malawa-Quelle: nördl. des Bowonglangi in S.-Celebes”.

##### Material examined.

Malawa-Quelle, Celebes, don.: Dr. P. u. F. Sarasin, 1918, NHMB 2411.a (lectotype [designated in Egorov 2013]; photographs examined).

##### Remarks.

Protoconch matte, without notable sculpture; R1 with fine, regular, low ribs and weaker spiral striation; R2 extremely short, with slightly elevated, white ribs. No operculum was found.

#### Stomacosmethis senyumensis

Taxon classificationAnimaliaGastropodaCyclophoridae

(Foon & Liew, 2017)

9B22FEB4-07B2-5FFE-B2D6-21BE2AB28B7E

Alycaeus senyumensis Foon & Liew, 2017: 67–69, figs 7V, W, 28, 31R.

##### Type locality.

“PHG 02 Gunung Senyum, Pahang (3°41'50"N, 102°26'04"E)”.

##### Remarks.

Seems to be a distinct species based on original description: short R2 and triangular orange-coloured shell confirm its position within this genus.

#### Stomacosmethis somnueki

Taxon classificationAnimaliaGastropodaCyclophoridae

(Panha & Patamakanthin, 2001)

A711EEB0-D1F1-5E23-8C87-014EAF3EF237

Alycaeus somnueki Panha & Patamakanthin, 2001: 189–190, pls 1–5.
Alycaeus huberi Thach, 2018: 19, figs 94, 95.
Alycaeus somnueki – Páll-Gergely et al. 2020: 37 (treats Alycaeus huberi [fig. 1 therein] as a synonym).
Alycaeus huberi – Thach 2020: 20, fig. 899 (synonymy rejected).

##### Type locality.

“Ao Luk limestone areas, Krabi Province, (...) Thailand”.

##### Material examined.

MNHN-IM-2000-34058 (holotype of *A. huberi*).

##### Remarks.

No specimens of *Stomacosmethis somnueki* were examined by us, but the original description and the published figures are sufficient for generic placement. Protoconch smooth, R2 short, with rather regular, low ribs.

#### Stomacosmethis spratti

Taxon classificationAnimaliaGastropodaCyclophoridae

(Godwin-Austen, 1888)

04524D6A-6143-5E03-BD21-4D242FB157FD

Alycaeus (Cycloryx) spratti Godwin-Austen, 1888: 245.
Alycaeus (Chamalycaeus) spratti – Kobelt 1902: 363.
Alycaeus (Cycloryx) spratti – Godwin-Austen 1914: 417, pl. 151, figs 10, 10a; Gude 1921: 284.

##### Type locality.

“Pinguong, Shan Hills, 2500 feet”.

##### Material examined.

Burmah, Shan Hills, 2500 ft, leg. Ponsonby, NHMUK 1887.6.23.24–26 (3 syntypes).

##### Remarks.

Protoconch rather matte, without spiral lines; R1 with dense, low, rather regular ribs and very weak spiral striation; R2 extremely short, with alternating darker/thicker and lighter/narrower stripes, which are elevated from the surface.

#### Stomacosmethis wilhelminae

Taxon classificationAnimaliaGastropodaCyclophoridae

(Maassen, 2006)

0F747A64-8ACD-5D5F-832D-F198318F39C4

Alycaeus wilhelminae Maassen, 2006: 138, figs 14–18.

##### Type locality.

“Indonesia, Sumatra, Aceh Besar, westcoast near Pasi, 10 km S. of Lhong, on limestone slope”.

##### Remarks.

We had no opportunity to examine the holotype, but the original description and the photographs published therein provide sufficient information regarding the generic status of this species. The protoconch of the weathered holotype is smooth and R2 is short and regularly ribbed. Maassen (2006) questioned the generic status of *A. wilhelminae* based on its closed umbilicus; further investigation should focus on the value of this character. However, at the moment we see no need to classify it in a genus other than *Stomacosmethis*.

### Nomina nuda

**“*Alycaeus scepticus* Theobald, 1863”**

**Remarks.**

The name *Alycaeus scepticus* appeared in Theobald (1863: 377), but it was not made available. Blanford (1965: 101) mentioned that it turned out to be a variety of *A. ingrami*. Thus, Gude (1921) treated this name as a synonym of that species.

**“*Alycaeus anapetes* Panha & Burch, 1998”**

**Remarks.**

The name *Alycaeus anapetes* Panha & Burch, 1998 appeared in the comparisons section under *Alycaeus somnueki* (see Panha and Patamakanthin 2001: 189). However, this taxon has never been formally described.

**“Alycaeus (Charax) longituba var. latestriata Kobelt & Möllendorff, 1897”**

**Remarks.**

This name was mentioned by Kobelt and Möllendorff (1897: 150) but has never been made available.

**“*Alycaeus broti* Aldrich, 1889”**

**Remarks.**

See under *Chamalycaeus everetti* (Godwin-Austen, 1889).

**“Alycaeus (Orthalycaeus) jagori var. trigonostoma”**

**Remarks.**

Kobelt (1902) mentioned that the taxon Alycaeus (Orthalycaeus) jagori var. trigonostoma was listed by Kobelt and Möllendorff (1897, Nachrichtsblatt der Deutschen Malakozoologischen Gesellschaft 29) on page 150, but we did not find that taxon listed there. This name has never made available.

### Manuscript names

**“*Alycaeus pomatiaeformis* Ancey”**

**Remarks.**

A sample in the RBINS (Borneo, coll. Ancey, 28.VI.08) was labelled in this manner. The shell had a very wide peristome, and may be a new species.

***Chamalycaeus satsumanus kiiensis* Kuroda**

**Remarks.**

Museum samples from the Sakurai and Inuba collections in the NSMT bear this name, but was never been formally published (Hanshin Shell Club 1986).

## Concluding remarks

After examining specimens of virtually all alycaeid taxa and the relevant literature, here we propose a novel classification of 320 accepted species and 43 subspecies into seven genera. The following conclusions can be made:

(1) While the genera *Dicharax*, *Dioryx*, and *Metalycaeus* have unique character states which serve as diagnostic traits, the other four genera are defined based on a combination of shell characters. Distinctness between *Pincerna* and *Alycaeus* and between *Pincerna* and *Stomacosmethis* remain to be verified.

(2) The most important achievements of this study are the separation of *Stomacosmethis* from *Alycaeus*, and the identification of *Dicharax* and *Metalycaeus* species. We also found a common anatomical trait of the Alycaeidae (bursa copulatrix originates from the lateral side of the ovarium) besides the unique sutural tube-microtunnel system, which is probably a device for gas exchange.

(3) The species-level diversity (i.e., number of described species) is largely dependent on whether the splitting or lumping approaches were employed by the taxonomists who classified specimens from particular areas, which resulted in peculiar geographic differences in species diversity.

(4) Various lines of evidence suggest the presence of three subgroups within the Alycaeidae (*Alycaeus*-*Dioryx*, *Chamalycaeus*-*Dicharax*-*Metalycaeus*, *Pincerna*-*Stomacosmethis*), which can be tested by future studies.

### Relationships between genera

Morphological traits (shell, genital system, radula) presented here and unpublished molecular phylogenetic pattern suggest the presence of three groups within Alycaeidae. Firstly, the genera *Alycaeus* and *Dioryx* form a distinct group based on the blunt central cusp of radula and the position of the bursa copulatrix that starts posterior to the middle section of the ovarium. Their similarly rather large shell sizes (at least for many *Dioryx* species and all *Alycaeus* species) and overlapping distribution suggest these two genera might be closely related. Secondly, the genera *Chamalycaeus*, *Dicharax*, and *Metalycaeus* generally possess the depressed shell and have a plesiomorphic radula type with a central, round tooth that has pointed cusps. The bursa copulatrix does not extend beyond the ovarium, which can be a synapomorphic characteristic for this group. Thirdly, *Pincerna* and *Stomacosmethis* are both characterised by having a very short tube and the elevated spire and inhabit a largely overlapping geographical area. However, the genital traits of the single known *Pincerna* and *Stomacosmethis* species are strikingly different, and the radula of *Stomacosmethis* with an elongated central tooth and few cusps is unique among Alycaeidae. Thus, dependable grouping of the recognised alycaeid genera is not yet possible because the morphology of radula and genital system, and DNA sequences have been available only in a limited number of alycaeid species.

Molecular phylogeny could be a powerful tool to obtain testable hypotheses of evolutionary relationships between species and/or higher taxa. In our attempts, direct PCR amplification of DNA extracted from Alycaeidae snails was not sufficiently successful as that from other families (Junn Kitt Foon, pers. comm.), although few alycaeid taxa have been included in preceding studies (Webster et al. 2012; Hendricks et al. 2019). Conventionally used primers may need to be specifically modified for several key alycaeids to resolve the generic phylogeny of this family. Our analysis of generic relationships including the outcome of molecular phylogeny without *Dioryx* suggests that the genus *Alycaeus* is distant from *Stomacosmethis* and *Pincerna*, which are closely related and may not be mutually monophyletic. *Chamalycaeus*, *Metalycaeus* and *Dicharax* are also close relatives of one another within the family Alycaeidae (Páll-Gergely et al. in prep.).

### Biogeography and fossil records

**Figure 46.**
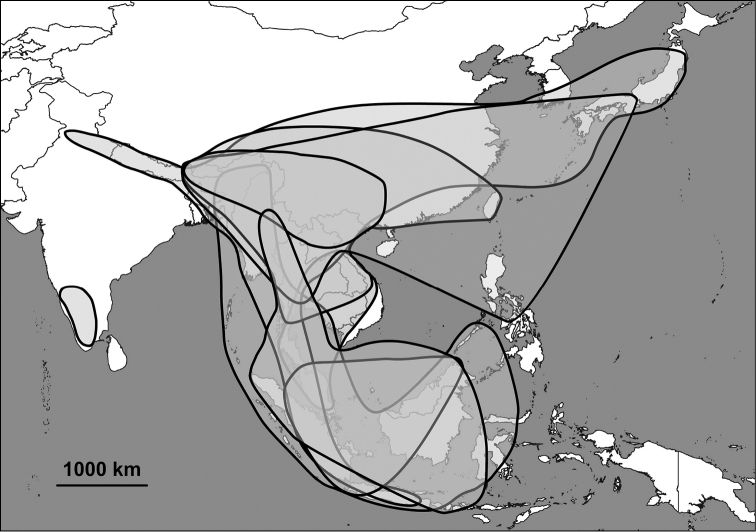
Distribution of all alycaeid genera showing regions with relatively high generic diversity.

The Alycaeidae is a characteristic family of the Oriental Biogeographic Region, with a few genera expanding their distributions to the Sino-Japanese/Palaearctic realms (see Holt et al. 2013) (Fig. 46). Isolated occurrence of alycaeids in the Western Ghats supports a hypothesis that the fauna of that region is mostly of Southeast Asian origin (Raheem et al. 2014, Fred Naggs, pers. comm.).

There are three areas that can be considered as biodiversity centres for this family, namely the southeastern Himalaya, northern Vietnam/southern China, and peninsular Malaysia/Sumatra (Table 5), each inhabited by five genera. The diversity in terms of the number of genera decreases towards the periphery of these areas, e.g., southwestern Himalaya (1 genus), southern India (1 genus), Japan (2 genera), and the Philippines (2 genera). The current classification suggests that alycaeids expanded their distributions to Japan and the Philippines in two independent events (Páll-Gergely and Auffenberg 2019). Budha et al. (2015) (and also Kuznetsov 1996 and Kuznetsov and Schileyko 1997) reported several alycaeid species from Nepal, but these were not examined by us. Identification of alycaeids is only possible by careful comparison with the type material, which was not done in these studies. Thus, we did not include their identification data in Nepal.

**Table 5.** Distribution of genera in the three regions with high diversity. Double asterisks indicate relatively high taxon diversity of that given genus.

	Peninsular Malaysia and Sumatra	Northern Vietnam, northern Laos and southern China	Southeastern Himalaya
* Alycaeus *	*	*	
* Chamalycaeus *	*		*
* Dicharax *	*	**	**
* Dioryx *		**	*
* Metalycaeus *		*	*
* Pincerna *	**	*	**
* Stomacosmethis *	**		

Although Japan is not rich in the number of genera, unique features of conchology are found in a few of the Japanese species, such as the extremely expanded aperture of *D. expanstoma*, detached initial whorls of *D. miyazakii*, and uniquely shaped peristome with weak parietal callus in *D. okamurai*.

The genus *Pincerna* has a conspicuously disjunct distribution. One set of species inhabits the Himalaya, northern Thailand, northern Laos, northern Vietnam, and southern China, whereas the other inhabits peninsular Malaysia, Sumatra, and Borneo.

The only fossil alycaeid is 23–21 million years old Dicharax (?) sonlaensis (see Raheem and Schneider 2018) from northern Vietnam, which fits the range of morphological variation of an extant *Dicharax* species of the same region. In recent years several terrestrial caenogastropods were described from 99 million year-old Burmese ambers, and there is more to come. All cyclophoroidean families of Southeast Asia (Cyclophoridae, Diplommatinidae, Pupinidae) have been found from mid-Cretaceous Burmese amber (Hirano et al. 2019; Neubauer et al. 2019) with the exception of the Alycaeidae. The absence of alycaeids in Burmese amber may suggest that this is a younger group than the other related families, which is in agreement with the molecular dating of Hirano et al. (2019). Nevertheless, special attention should be paid on alycaeid-like fossil shells since they would not be recognised as alycaeids without the sutural tube and the R2 region that is differently ribbed than the other parts of the body whorl.

## Supplementary materials

Supplementary material 1 Figure S1. Tukik, Central Aceh, specimen 1 (HNHM 104858) Data type image file https://binary.pensoft.net/file/468692This dataset is made available under the Open Database License (http://opendatacommons.org/licenses/odbl/1.0/). The Open Database License (ODbL) is a license agreement intended to allow users to freely share, modify, and use this Dataset while maintaining this same freedom for others, provided that the original source and author(s) are credited.Barna Páll-Gergely
