# Supplementary figures and images for: Genus-level revision of the Alycaeidae (Gastropoda, Cyclophoroidea), with an annotated species catalogue
Source: Zookeys. 2020 Oct 29;981:1–220. doi: 10.3897/zookeys.981.53583 (PMC7644702; doi:10.3897/zookeys.981.53583)

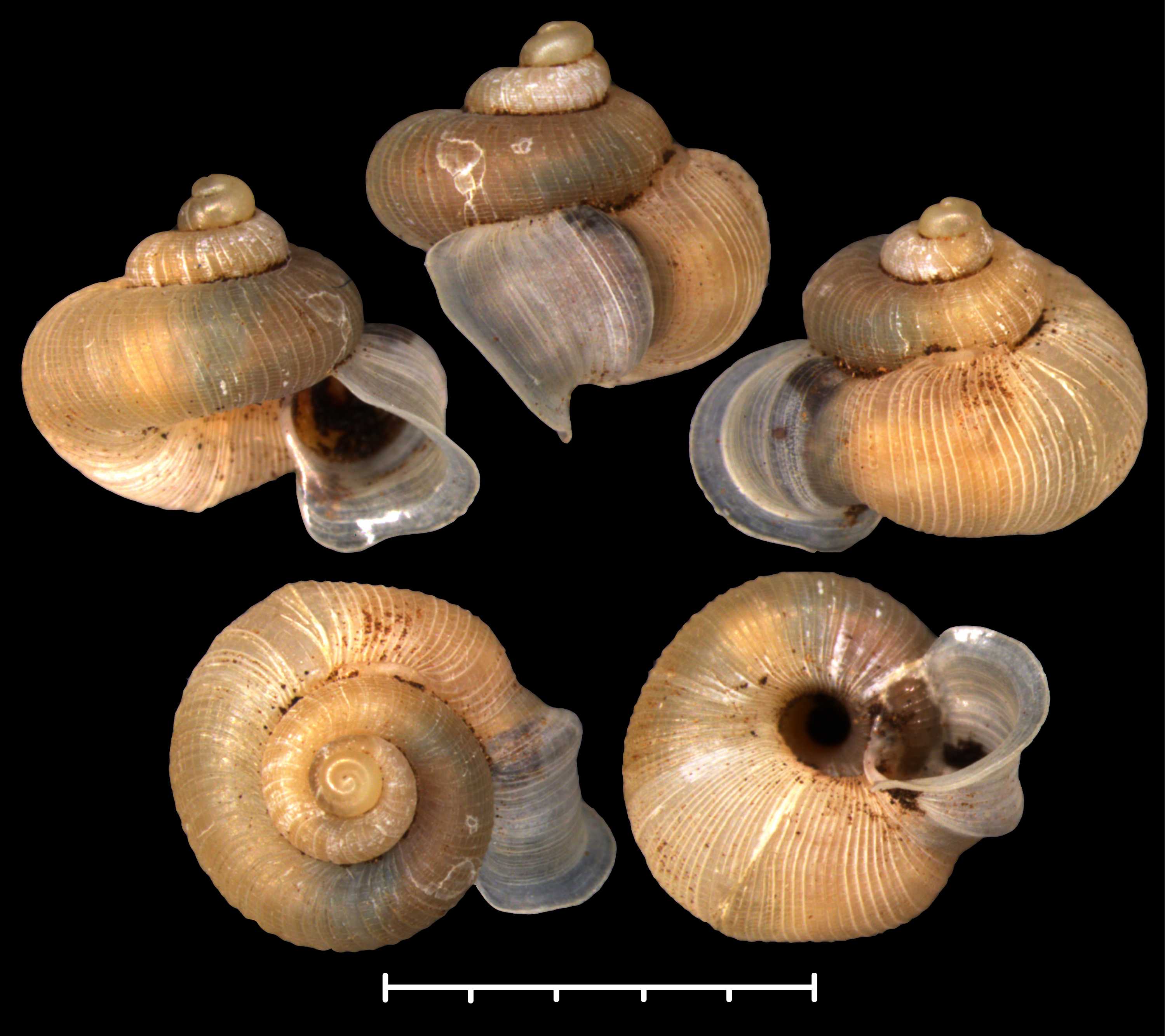

Supplement: Supplementary material 1 — Figure S1. Tukik, Central Aceh, specimen 1 (HNHM 104858) [file zookeys-981-001-s001.jpg]
